# Difluoromethanesulfonyl hypervalent iodonium ylides for electrophilic difluoromethylthiolation reactions under copper catalysis

**DOI:** 10.1098/rsos.160102

**Published:** 2016-05-25

**Authors:** Sadayuki Arimori, Okiya Matsubara, Masahiro Takada, Motoo Shiro, Norio Shibata

**Affiliations:** 1 Department of Frontier Materials, Nagoya Institute of Technology, Gokiso, Showa-ku, Nagoya 466-8555, Japan; 2 Rigaku Corporation, 3-9-12 Matsubara-cho, Akishima, Tokyo 196-8666, Japan

**Keywords:** difluoromethylthiolation, hypervalent iodonium ylide, carbene, sulfur, fluorine

## Abstract

Difluoromethanesulfonyl hypervalent iodonium ylides 2 were developed as electrophilic difluoromethylthiolation reagents for a wide range of nucleophiles. Enamines, indoles, β-keto esters, silyl enol ethers and pyrroles were effectively reacted with 2 affording desired difluoromethylthio (SCF_2_H)-substituted compounds in good to high yields under copper catalysis. The reaction of allyl alcohols with 2 under the same conditions provided difluoromethylsulfinyl (S(O)CF_2_H) products in good yields. The difluoromethylthiolation of enamines is particularly effective with wide generality, thus the enamine method was nicely extended to the synthesis of a series of difluoromethythiolated cyclic and acyclic β-keto esters, 1,3-diketones, pyrazole and pyrimidine derivatives by a consecutive, two-step one-pot reaction using 2.

## 1. Introduction

**Figure 1.**
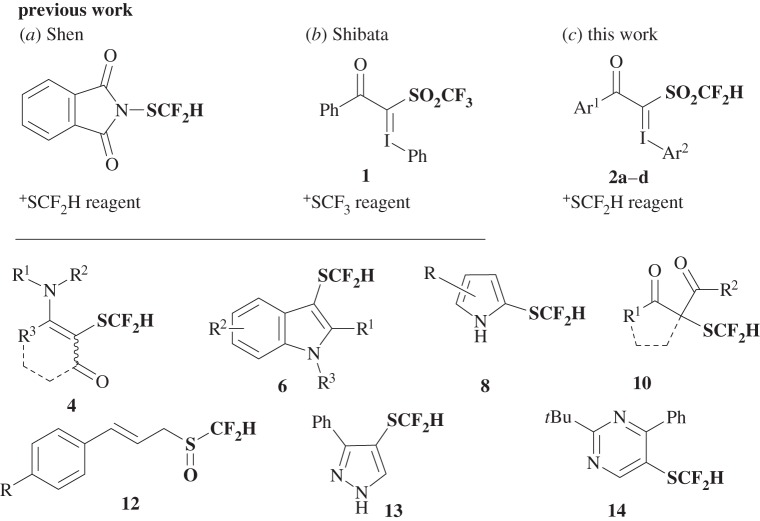
Previous studies on (*a*) ^+^SCF_2_H reagent, (*b*) ^+^SCF_3_ reagent and (*c*) hypervalent iodonium ylides as ^+^SCF_2_H reagents.

Fluorine (F) and sulfur (S) atoms have been individually recognized over the past couple of decades to be important structural elements with biological activities in drugs [1–10]. These facts, together with the recent successful observation on the market that the trifluoromethyl (CF_3_) group is frequently found in pharmaceuticals and agrochemicals [11–15], have led medicinal chemists to explore the use of the trifluoromethylthio (SCF_3_) group as a strategic functional component to assist in drug discovery [16–38]. In recent years, more than a dozen attractive synthetic methods for introduction of the SCF_3_ group into target compounds have been successively reported [16–38]. In this context, the difluoromethylthio group (SCF_2_H) has emerged as a next potential subject in this field. While SCF_3_ is entirely lipophilic, the SCF_2_H group has the potential to be a weak hydrogen-bonding donor, which results in a suitable hydrophilic/hydrophobic balance of SCF_2_H-substituted molecules [39–42]. Thus, incorporation of SCF_2_H into biologically active molecules should permit the efficient design of novel, viable drug candidates. There are several synthetic approaches available for SCF_2_H-substituted compounds [43–59], such as nucleophilic reaction of appropriate thiolates to difluoromethyl carbine [43–53] and electrophilic or radical difluoromethylation of thiolates [54–56]. These methods rely upon the construction of a bond between S and CF_2_H, and therefore have some limitations, although recently Goossen *et al*. provided a solution via copper-mediated difluoromethylation of organothiocyanates [57,58]. Impressively, Shen and co-workers [60] reported Sandmeyer-type direct diifluoromethylthiolation using an *N*-heterocyclic carbene difluoromethylthiolated silver complex to provide aryl SCF_2_H compounds. The method is an ideal approach for introducing SCF_2_H, but the substrate scope is limited to diazonium salts. The same group reported the first shelf-stable electrophilic difluoromethylthiolation reagent, *N*-difluoromethylthiophtalimide or the Shen reagent (figure 1*a*) [61].

The Shen reagent is efficient, but new reagents and more methods to expand the accessibility to a wide variety of SCF_2_H compounds are continuously required. Incidentally, we reported in 2013 that trifluoromethanesulfonyl (SO_2_CF_3_) hypervalent iodonium ylide **1** is an efficient reagent for the electrophilic trifluoromethylthiolation reaction (figure 1*b*) [62]. Despite its carbon-SO_2_CF_3_ structure, a reactive SCF_3_ species is unexpectedly, but effectively released from **1** via C–S bond cleavage under copper catalysis allowing it to be transferred into a wide variety of nucleophilic substrates including enamines, indoles, β-keto esters, pyrroles [63], allylsilanes, silyl enol ethers [64], allyl alcohols and boronic acids [65]. Inspired by this powerful reactivity and wide substrate generality and linked to the mechanistic uniqueness of iodonium ylide reagent **1**, we describe herein an investigation of novel shelf-stable electrophilic difluoromethylthiolation reagents **2** and their reactivity towards a variety of nucleophiles (figure 1*c*). Difluoromethanesulfonyl (SO_2_CF_2_H) hypervalent iodonium ylides **2** were found to be useful for electrophilic difluoromethylthiolation of a variety of nucleophiles including enamines **3**, indoles **5**, pyrroles **7** and β-keto esters **9** to provide corresponding SCF_2_H products **4**, **6**, **8** and **10**. The reaction of allyl alcohols **11** with **2** under the same conditions provided difluoromethylsulfinyl (S(O)CF_2_H) products **12**, instead of SCF_2_H products, in good yields. These methods can be applied to the synthesis of a series of difluoromethylthiolated cyclic and acyclic β-keto esters, 1,3-diketones **10**, pyrazole **13** and pyrimidine **14** by a consecutive, two-step one-pot reaction using **2** under an enamine strategy. The reactivity and reaction mechanism of **2** are discussed.

## 2. Results and discussion

**Scheme 1.**
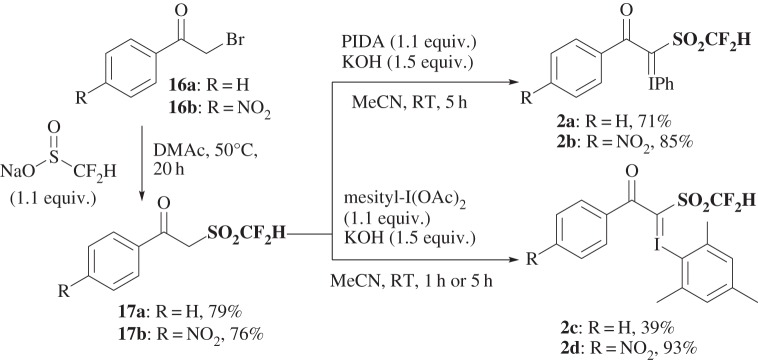
Preparation of difluoromethylthiolation reagents **2**.

Preparation of difluoromethanesulfonyl hypervalent iodonium ylides **2** is shown in scheme 1. 2-Bromoacetophenone (**16a**) was treated with sodium difluoromethanesulfinate [66] in dimethyl-\break acetamide (DMAc) at 50°C for 20 h to give 2-difluoromethanesulfonylacetophenone (**17a**) in 79% yield. The reaction of **17a** with phenyliodonium diacetate (PIDA) in the presence of potassium hydroxide provided **2a** in 71% yield. Other reagents **2b**, **2c** and **2d** were prepared using a method similar to that for **2a** (scheme 1). All the reagents are crystals and stable enough for practical use, except for **2c**. They can be maintained in a refrigerator (0°C). The most stable reagent is **2d** and ^19^F-NMR-based stability of the reagents can be ranked as **2d** > **2b** > **2a** >>> **2c** (electronic supplementary material, table S1).

**Scheme 2.**
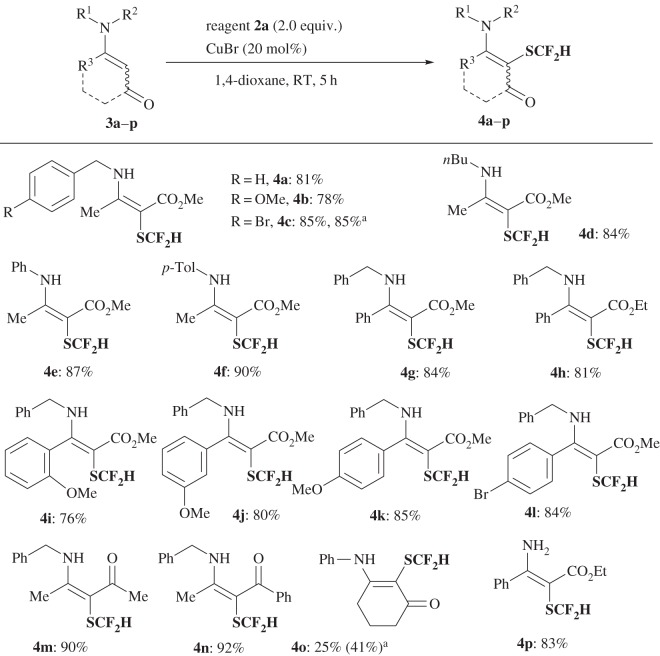
Difluoromethylthiolation of enamines **3**. Superscript ‘a’ denotes reagent **2d** was used instead of **2a**.

We began our study on difluoromethylthiolation with **2a** using β-enamino ester **3a**. After screening the reaction conditions (electronic supplementary material, table S2), a catalytic amount of Cu(I)Br (20 mol%) in 1,4-dioxane at room temperature was found to be the best set of conditions, and the difluoromethylthiolation reaction proceeded well providing α-SCF_2_H-β-enamino ester **4a** in 94% yield (run 4, electronic supplementary material, table S2). Substrate generality for the difluoromethylthiolation of β-enamino esters **3** by **2a** was investigated (scheme 2). As shown in scheme 2, a wide range of β-enamino esters **3** were found to be suitable substrates, furnishing the corresponding SCF_2_H-enamines **4** in high yields independent of the substitution on the nitrogen atom (benzyl, alkyl and aryl), the size of the ester group (OMe, OEt) or the enamine skeleton (methyl or aryl enamines). The reactions of enamino ketones **3m**,**n** were also efficient under the same conditions to furnish α-SCF_2_H-β-enamino ketones **4m**,**n** in 90% and 92% yield, respectively, independent of the existence of enolizable ketone. The use of cyclic enamino ketone **3o** was also attempted and the desired product **4o** was obtained in 25% yield, which improved slightly to 41% after using reagent **2d**. In addition, *N*-unprotected β-enamino ester **3p** was applied under the same conditions to give a high yield of **4p** (83%). The structure of **4** was confirmed by ^19^F NMR, ^1^H NMR, ^13^C NMR, IR and mass spectra. The X-ray crystallographic structure of **4c** was analysed (CCDC 1446329; electronic supplementary material, figure S1).

**Scheme 3.**
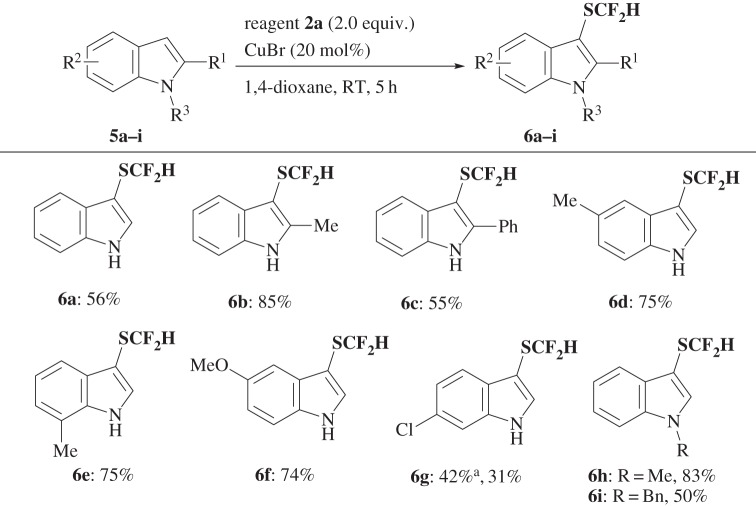
Difluoromethylthiolation of indoles **5**. Superscript ‘a’ denotes reagent **2d** was used instead of **2a**.

**Scheme 4.**
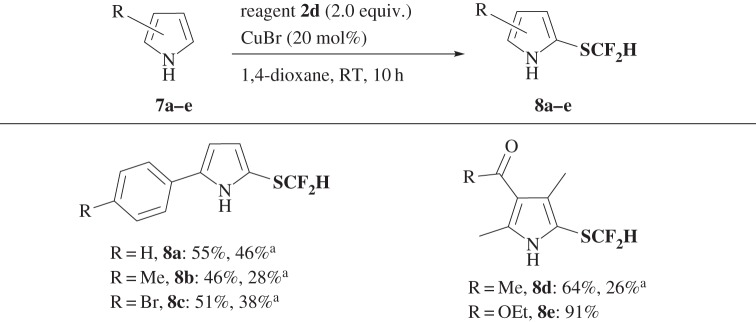
Difluoromethylthiolation of pyrroles **7**. Superscript ‘a’ denotes reagent **2a** was used.

**Scheme 5.**
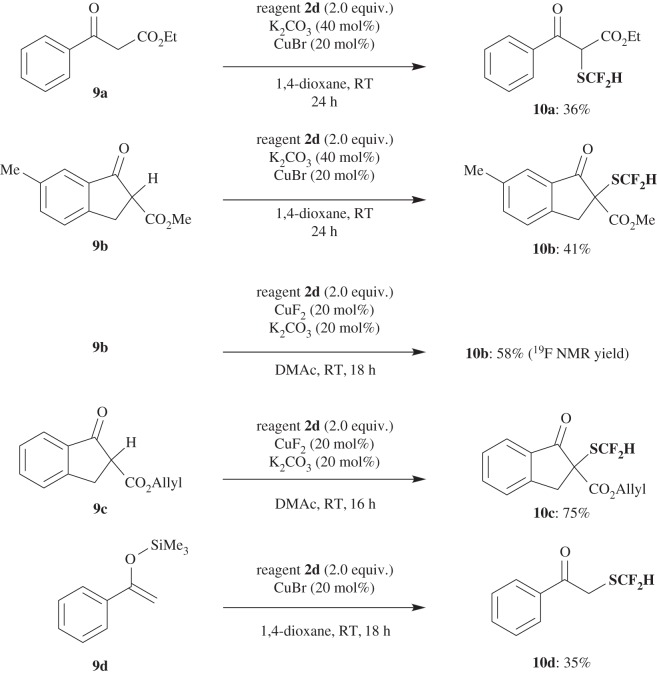
Difluoromethylthiolation of β-keto esters **9a–c** and silyl enol ether **9d**.

Reagent **2a** was found to have wide applicability as a difluoromethylthiolation reagent not only for enamines, but also for a variety of nucleophiles, such as indoles **5** (scheme 3), pyrroles **7** (scheme 4), β-keto esters **9a–c** (scheme 5) and silyl enol ether **9d** (scheme 5) under the same or slightly modified conditions to provide corresponding SCF_2_H products **6**, **8** and **10** in good yields. When yields were not satisfactory, they could be improved by using regent **2d** instead of **2a**, in particular, for the difluoromethylthiolation of pyrroles **7** (for optimized reaction conditions, see electronic supplementary material, table S3) and β-keto esters **9** (scheme 5).

**Scheme 6.**
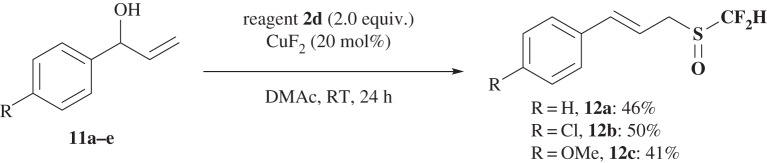
Reaction of allyl alcohols **11** with **2a**.

The reaction of allyl alcohol **11a** with **2a** under the optimized conditions of CuF_2_ in DMAc (see electronic supplementary material, table S4) gave a difluoromethylsulfinyl, S(O)CF_2_H compound **12a** in moderate yield (46%) via a [2,3]-sigmatropic rearrangement, instead of an SCF_2_H compound. Both the electron-donating (OMe) and electron-deficient (Cl) groups were applicable in the reaction (scheme 6).

**Scheme 7.**
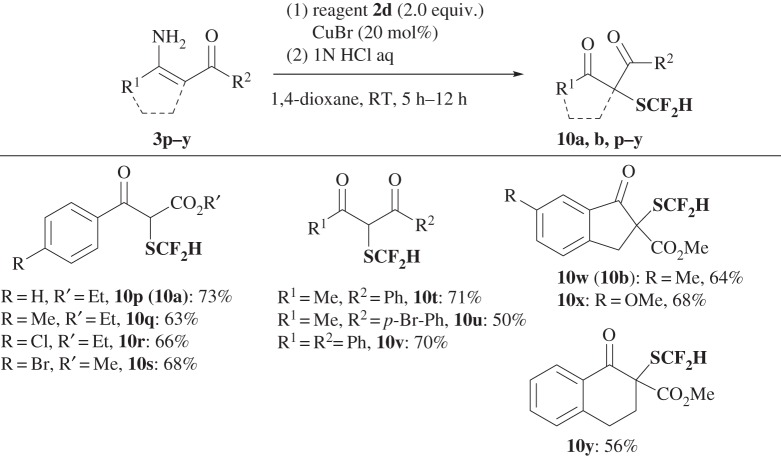
Enamine method: two-step, one-pot synthesis of difluoromethylthiolated β-keto esters and 1,3-diketones **10** from NH_2_-enamines **3p–y**.

The difluoromethylthiolation reaction by **2** is particularly useful for the reaction of enamines. It should be noted that the difluoromethylthiolation of enamines, i.e. the enamine method, can be expanded to the synthesis of α-SCF_2_H-β-keto esters and α-SCF_2_H-1,3-diketones by a one-pot combination of reactions that involves difluoromethylation of unprotected, NH_2_-enamine esters and enamine ketones **3p**–**y** with **2a** and subsequent hydrolysis (scheme 7). A series of acyclic and cyclic α-SCF_2_H-β-ketoesters and α-SCF_2_H-1,3-diketones with a variety of substituents were obtained in high yields (**10a, b, p**–**y**: 63–73%). This enamine method has the advantage of higher yields relative to the direct reaction with β-keto esters. More importantly, acyclic α-SCF_2_H, β-keto esters and ketones are not prepared using the Shen reagent [61], presumably due to the lower reactivity of acyclic substrates than cyclic ones (**9a–c**; scheme 5).

**Scheme 8.**
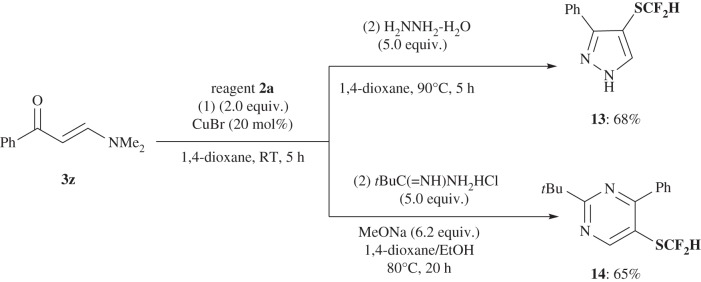
Enamine method: two-step, one-pot synthesis of difluoromethylthiolated pyrazole **13** and pyrimidine **14** from enamine **3z**.

The enamine method was further extended to allow the synthesis of biologically attractive SCF_2_H-substituent heterocycles of pyrazole and pyrimidine by a similar two-step, one-pot, consecutive reaction procedure (scheme 8). First, enamine ketone **3z** was treated with **2a** in the presence of CuBr in dioxane at room temperature for 5 h. The addition of hydrazine monohydrate (5.0 equiv.) followed by heating and cyclohydration produced 4-difluoromethylthiolated pyrazole **13** in 68% yield. Similarly, the difluoromethylation of **3z** with **2a** followed by treatment with *tert*-butylcarbamidine hydrochloride (5.0 equiv.) and sodium methoxide (6.2 equiv.) under heated conditions gave 5-difluoromethylthiolated pyrimidine **14** in 65% yield.

**Scheme 9.**
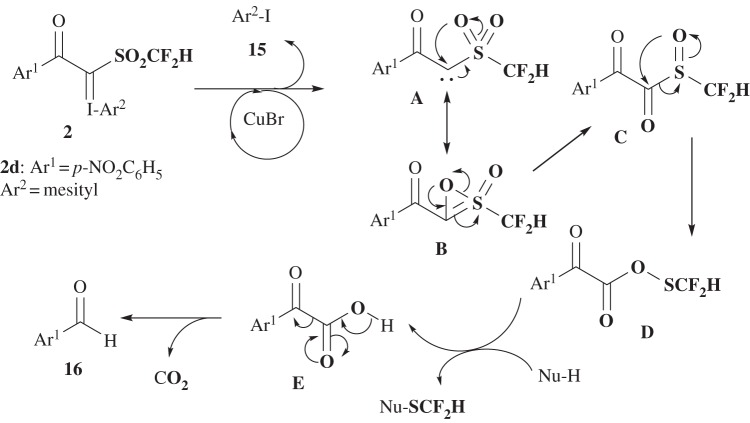
The proposed reaction mechanism.

A proposed reaction mechanism of difluoromethylthiolation by reagent **2** is postulated in scheme 9. This mechanism is principally the same as a previous consecutive reaction mechanism [62,63] by SCF_3_-reagent **1** involving successive (i) copper-catalyzed carbene-generation **A**, (ii) oxathiirene-2-oxide formation **B**, (iii) rearrangement to sulfoxide **C**, and (iv) collapse to thioperoxoate **D**. Hence, the SCF_2_H thioperoxoate **D** is likely to be an actual species for electrophilic difluoromethylthiolation of nucleophiles via decarboxylation of **E**. Detection of the residues, Ar^2^-I **15** (Ar^2^ = mesityl) and Ar^1^CHO **16** (Ar^1^ = *p*-NO_2_Ph) after the reaction with reagent **2d**, together with the previous mechanistic investigation using SCF_3_-reagent **1** [62,63], strongly support the reaction mechanism shown in scheme 9 (also see electronic supplementary material, scheme S3).

The roles of copper catalysts, CuF_2_ and CuBr, depending on the substrates are not known. The Lewis acid centre of CuF_2_ is harder than the Lewis acid centre of CuBr; since Cu(II) is harder than Cu(I), F^−^ is harder than Br^−^. The reaction mechanism in scheme 9 includes sulfur atoms with different oxidation states with different softness and hardness (from soft to hard: S, S(O) and SO_2_) [67], thus the catalyst of CuF_2_ or CuBr might also activate the different stage of each transition state. The more clear explanation should be required based on the detailed study such as molecular calculations.

## 3. Conclusion

In conclusion, the preparation and application of novel electrophilic difluoromethylthiolation reagents **2a**–**d** have been developed. Reagents **2** were found to be useful for the difluoromethylthiolation of a wide range of enamines, indoles, pyrroles and β-keto esters. Allylic alcohols were also reacted with **2** to provide allylic S(O)CF_2_H compounds via sigmatropic rearrangement. The difluoromethylthiolation of enamines (enamine method) can be widely extended to the synthesis of a variety of SCF_2_H-β-keto esters, 1,3-diketones, pyrazole and pyrimidine under a two-step one-pot procedure. High yields are obtained with a wide substrate scope and the reactions proceed at room temperature. This should be compared with Shen's recent papers on SCF_2_H transfer, which need elevated temperatures and prolonged reaction times [61] or stoichiometric amounts of a silver complex. Besides, the access to the SCF_2_H-β-keto esters and 1,3-diketones is more general by our reagents than the Shen reagent [61]. Because the fluorine often induces some expectation and something interesting [9,10,68–72], our new SCF_2_H reagents would be efficient tools for the development of novel drugs and functional materials. Further investigation of reagent **2** for the difluoromethylthiolation of other substrates, such as heteroatom nucleophiles (N-, S- or P-nucleophiles), is underway.

## Supplementary Material

160410RSopen_SI: PDF file, experimental details

## Data accessibility

The datasets supporting this article have been uploaded as part of the electronic supplementary material.
