# Supplementary material for: Difluoromethanesulfonyl hypervalent iodonium ylides for electrophilic difluoromethylthiolation reactions under copper catalysis
Source: R Soc Open Sci. 2016 May 25;3(5):160102. doi: 10.1098/rsos.160102 (PMC4892452; doi:10.1098/rsos.160102)
Supplement: 160410RSopen_SI: PDF file, experimental details [file rsos160102supp1.pdf]

Supporting Information

Difluoromethanesulfonyl Hypervalent Iodonium Ylides for Electrophilic Difluoromethylthiolation Reactions under Copper Catalysis

Sadayuki Arimori †, Okiya Matsubara †, Masahiro Takada †, Motoo Shiro ‡ and Norio Shibata † *

† Department of Frontier Materials, Nagoya Institute of Technology, Gokiso, Showa-ku, Nagoya 466-8555, Japan.
‡ Rigaku Corporation, 3-9-12 Matsubara-cho, Akishima, Tokyo 196-8666, Japan.

Tel: (+81) 52-735-7543, Fax: (+81) 52-735-5442
E-mail: nozshiba@nitech.ac.jp

Table of Content

1. General Information ……………………………………………………………2
2. Preparation of Reagent **2a-d** and Starting Materials ……………………………...2
3. Experimental Details ………………………………………………………...6
4. Optimization of Conditions ……………………………………………………12
5. Analytical Data ………………………………………………………………...14
6. X-ray structure of **4c** ……………………………………………………...35
7. References and Notes……………………………………………………………..35
8. ^{19}F, ^{1}H, ^{13}C NMR Spectra of Corresponding Compounds…………………………..36

1. General Information

All reactions were performed in oven-dried glassware under a positive pressure of nitrogen. Solvents were transferred via syringe and were introduced into the reaction vessels through a rubber septum. All solvents were purified by standard method. All of the reactions were monitored by thin-layer chromatography (TLC) carried out on 0.25 mm Merck silica gel (60-F254). The TLC plates were visualized with UV light or $KMnO_4$ in water/heat. All of the reaction products were purified by Column chromatography. Column chromatography was carried out on a column packed with silica gel 60N spherical neutral size 63-210 mm. The ^{19}F NMR (282 MHz) and ^{1}H NMR (300 MHz) in $CDCl_3$, $(CD_3)_2CO$ or $(CD_3)_2SO$ were recorded on a Varian Mercury 300. ^{13}C NMR (125 MHz) spectra for solution in $CDCl_3$, CD_3CN or $(CD_3)_2SO$ were recorded on a BRUKER Advance 500. Chemical shifts (δ) are expressed in ppm downfield from internal TMS (δ = 0.00) or C_6F_6 [δ = -162.2 ($CDCl_3$) or -163.5 ($(CD_3)_2CO$)] as an internal standard. Coupling constants (*J*) are in Hertz (Hz). The following abbreviations were used to explain the multiplicities: s = singlet, d = doublet, t = triplet, q = quartet, m = multiplet, br = broad. Mass spectra were recorded on a SHIMADZU GCMS-QP5050A (EI-MS) and SHIMAZU LCMS-2020 (ESI-MS and APCI-MS). Infrared spectra were recorded on JASCO FT/IR-200 or a JASCO FT/IR-4100 spectrometer.

2. Preparation of Reagent 2a-d and Starting Materials

Synthesis of Reagent 2a-d

Scheme S1. Synthesis of reagent **2a-d**

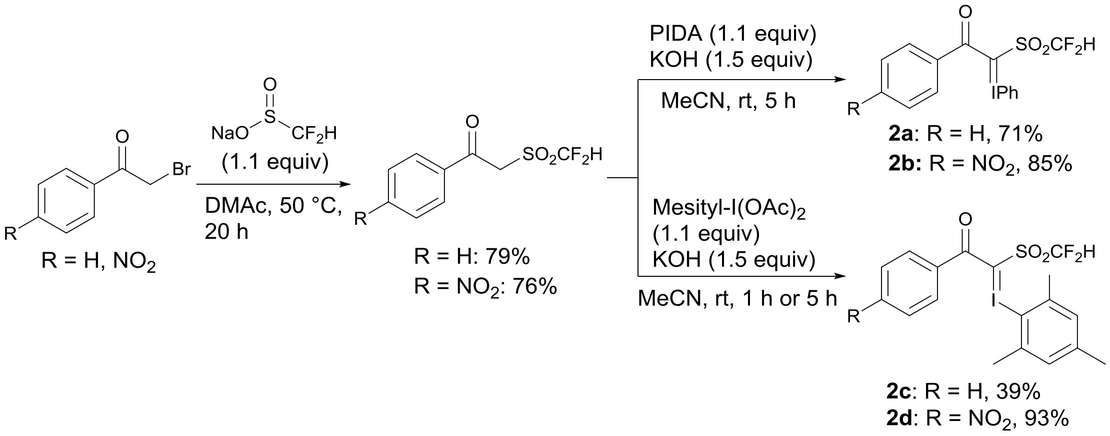

Synthesis of 1-phenyl-2-[(difluoromethyl)sulfonyl]ethanone:

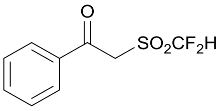

A mixture of 2-bromoacetophenone (4.38 g, 22.0 mmol) and sodium difluoromethanesulfinate (3.34 g, 24.2 mmol) in DMAc (100 mL) was stirred at 50 ºC for 20 h. Then water (50 mL) was added to the mixture at room temperature. The resulting mixture was extracted with Et_2O and organic layer was washed with water 2 times, brine for once then dried over magnesium sulfate. The solvent was removed by rotary evaporation to give a crude product then the crude product was purified by frash column chromatography on silica gel (eluent: ethyl acetate/hexane =1/4, Rf = 0.2 - 0.3). A light yellow solid (1-phenyl-2-[(difluoromethyl)sulfonyl]ethanone) was afforded (4.1 g , 79%). Mp: 106-107 ºC.

^{1}H NMR (300 MHz, $CDCl_3$): δ ppm 7.95 (d, *J* = 7.5 Hz, 2H), 7.71-7.68 (m, 1H), 7.58-7.53 (m, 2H), 6.67 (t, *J* = 54.0 Hz, 1H), 4.83 (s, 2H). ^{19}F NMR (282 MHz, $CDCl_3$): δ ppm -125.35 (d, *J* = 53.3 Hz, 2F). ^{13}C NMR (125 MHz, $CDCl_3$): δ ppm 187.65, 135.22, 134.77, 129.19, 128.92, 114.32 (t, *J* = 284 Hz), 56.87. IR (KBr): ν = 3036, 2958, 2915, 1681, 1452, 1336, 1154, 1108 cm^{-1}. MS (ESI): 257 (M+Na). HRMS (ESI) $C_9H_8F_2NaO_3S$ (M+Na) for Calcd: 257.0060, Found: 257.0059.

Synthesis of reagent 2a:

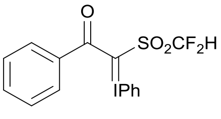

To a mixture of 1-phenyl-2-[(difluoromethyl)sulfonyl]ethanone (3.22 g 13.7 mmol) and KOH (1.16 g, 20.6 mmol) in MeCN (100 mL), PIDA (4.87 g, 15.1 mmol) was added. After stirring for 5 h at room temperature, cold water (200 mL) was added to the mixture and the reaction mixture was stirred continuously for 0.5 h. The precipitate was filtered and washed with water and Et_2O then dried under vacuum. A white solid (reagent **2a**) was afforded (4.26 g, 71%). Mp: 72-73ºC (decomposed).
^{1}H NMR (300 MHz, $(CD_3)_2SO$): δ ppm 7.85 (d, *J* = 7.8 Hz, 2H), 7.62-7.57 (m, 1H), 7.53-7.47 (m, 2H), 7.39-7.29 (m, 5H), 6.70 (t, *J* = 53.1 Hz, 1H). ^{9}F NMR (282 MHz, $CDCl_3$): δ ppm -120.86 (d, *J* = 52.4 Hz, 2F). ^{13}C NMR (125 MHz, $CDCl_3$): δ ppm 186.53, 137.47, 134.56, 132.58, 131.87, 130.37, 130.24, 127.98, 127.60, 114.54 (t, *J* = 281 Hz), 94.37. IR (KBr): ν = 3056, 1685, 1523, 1444, 1325, 1279, 1154, 1092 cm^{-1}. MS (ESI): 475 (M+K). HRMS (EI) $C_{15}H_{11}F_2INaO_3S$ (M+Na) for Calcd: 458.9339, Found: 458.9340.

Synthesis of reagent 2c:

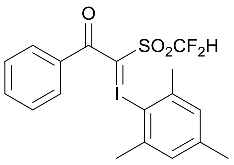

To a mixture of 1-phenyl-2-[(difluoromethyl)sulfonyl]ethanone (0.50 g 2.1 mmol) and KOH (0.18 g, 3.2 mmol) in MeCN (10 mL), iodomesitylene diacetate (0.85 g, 2.3 mmol) was added. After stirring at room temperature for 1 h, cold water (20 mL) was added to the mixture and the reaction mixture was stirred continuously for 0.1 h. The precipitate was filtered and washed with cold water and Et_2O then dried under vacuum. A white solid (reagent **2c**) was afforded (0.40 g, 39%). Mp: 58-59ºC (decomposed).
^{1}H NMR (300 MHz, $CDCl_3$): δ ppm 7.49 (d, *J* = 6.2 Hz, 2H), 7.40-7.33 (m, 3H), 7.06 (s, 2H), 5.92 (t, *J* = 55.2 Hz, 1H), 2.72 (s, 6H), 2.37 (s, 3H). ^{19}F NMR (282 MHz, $CDCl_3$): δ ppm -120.9 (d, *J* = 54.4 Hz, 2F). ^{13}C NMR (125 MHz, CD_3CN): δ ppm 187.24, 144.34, 143.35, 140.10, 130.64, 130.40, 128.79, 128.41, 123.06, 115.44 (t, *J* = 278 Hz), 73.46, 26.58, 20.92. IR (KBr): ν = 2970, 2943, 2916, 1688, 1600, 1448, 1363, 1170, 1127, 1004 cm^{-1}. MS (ESI): 479 (M+H). HRMS (EI) $C_{18}H_{17}F_2INaO_3S$ (M+Na) for Calcd: 500.9809, Found: 500.9816.

Synthesis of 1-(4-nitrophenyl)-2-[(difluoromethyl)sulfonyl]ethanone:

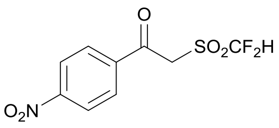

A mixture of 2-bromo-4’-nitoroacetophenone (2.00 g, 8.2 mmol) and sodium difluoromethanesulfinate (1.25 g, 9.1 mmol) in DMAc (70 mL) was stirred at 50 ºC for 20 h. Then water (50 mL) was added to the mixture at room temperature. The resulting mixture was extracted with Et_2O and organic layer was washed with water 2 times, brine for once then dried over magnesium sulfate. The solvent was removed by rotary evaporation to give a crude product then the crude product was purified by frash column chromatography on silica gel (eluent: ethyl acetate/hexane =1/1, Rf = 0.5 - 0.6). A yellow solid (1-(4-nitrophenyl)-2-[(difluoromethyl)sulfonyl]ethanone) was afforded (1.7 g , 76%). Mp: 83-84 ºC.

^{1}H NMR (300 MHz, $CDCl_3$): δ ppm 8.41 (d, *J* = 7.5 Hz, 2H), 8.15 (d, *J* = 7.2, 2H), 6.55 (t, *J* = 53.1 Hz, 1H), 4.85 (s, 2H). ^{19}F NMR (282 MHz, $CDCl_3$): δ ppm -124.0 (d, *J* = 53.6 Hz, 2F). ^{13}C NMR (125 MHz, $CDCl_3$): δ ppm 186.15, 151.29, 139.05, 130.05, 124.33, 114.44 (t, *J* = 285 Hz), 56.81. IR (KBr): ν = 3110, 2970, 1584, 1511, 1329, 1275, 1143, 1092 cm^{-1}. MS (ESI): 117 ($M-NO_2-SO_2CF_2H$). HRMS (EI) $C_9H_7F_2NO_5S$ for Calcd: 279.0013, Found: 279.0013.

Synthesis of reagent 2b:

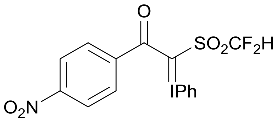

To a mixture of 1-(4-nitrophenyl)-2-[(difluoromethyl)sulfonyl]ethanone (0.50 g 1.8 mmol) and KOH (0.15 g, 2.7 mmol) in MeCN (10 mL), PIDA (0.64 g, 2.0 mmol) was added. After stirring at room temperature for 5 h, water (50 mL) was added to the mixture and the reaction mixture was stirred continuously for 0.5 h. The precipitate was filtered and washed with water and Et_2O then dried under vacuum. A white solid (reagent **2b**) was afforded (0.73g, 85%). Mp: 101-102 ºC (decomposed).

^{1}H NMR (300 MHz, $(CD_3)_2SO$): δ ppm 8.19 (d, *J* = 6.6 Hz, 2H), 7.96 (d, *J* = 7.5 Hz, 2H), 7.64-7.49 (m, 5H), 6.70 (t, *J* = 54.0 Hz, 1H). ^{19}F NMR (282 MHz, $(CD_3)_2CO$): δ ppm -121.6 (d, *J* = 51.3 Hz, 2F). ^{13}C NMR (125 MHz, $(CD_3)_2SO$): δ ppm 183.87, 147.40, 146.27, 133.20, 133.20, 131.16, 128.23, 122.75, 117.97, 114.28 (t, *J* = 280 Hz), 76.50. IR (KBr): ν = 3063, 2970, 1696, 1592, 1507, 1333, 1271, 1139, 1085 cm^{-1}. MS (ESI): 504 (M+Na). HRMS (EI) $C_{15}H_{10}F_2INNaO_5S$ (M+Na) for Calcd: 503.9190, Found: 503.9191.

Synthesis of reagent 2d:

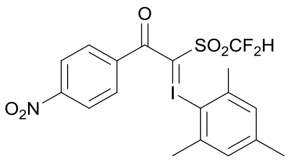

To a mixture of 1-(4-nitrophenyl)-2-[(difluoromethyl)sulfonyl]ethanone (0.50 g 1.8 mmol) and KOH (0.15 g, 2.7 mmol) in MeCN (10 mL), iodomesitylene diacetate (0.72 g, 2.0 mmol) was added. After stirring at room temperature for 5 h, water (50 mL) was added to the mixture and the reaction mixture was stirred continuously for 0.5 h. The precipitate was filtered and washed with water and Et_2O then dried under vacuum. A white solid (reagent **2d**) was afforded (0.87g, 93%). Mp: 113-114 ºC (decomposed).

^1H NMR (300 MHz, $(CD_3)_2SO$): δ ppm 8.16 (d, J = 8.4 Hz, 2H), 7.46 (d, J = 8.1 Hz, 2H), 7.12 (s, 2H), 6.50 (t, J = 53.4 Hz, 1H), 2.59 (s, 6H), 2.30 (s, 3H). ^{19}F NMR (282 MHz, $CDCl_3$): δ ppm -120.2 (d, J = 55.5 Hz, 2F). ^{13}C NMR (125 MHz, $(CD_3)_2SO$): δ ppm 183.43, 147.28, 146.42, 141.77, 141.63, 128.97, 128.46, 124.14, 122.57, 114.25 (t, J = 280 Hz), 74.65, 26.01, 20.48. IR (KBr): ν = 2970, 2370, 1584, 1511, 1329, 1275, 1143, 1092 cm^{-1}. MS (ESI): 555 (M+MeOH). HRMS (EI) $C_{18}H_{16}F_2INNaO_5S$ (M+Na) for Calcd: 545.9660, Found: 545.9658.

Preparation of Starting Materials

Sodium difluoromethanesulfinate was prepared by the reported procedure. [1]

All starting materials were known compounds. Enamine **3**, [2-4] **3z**, indole **5**, pyrrole **7** [5] and allyl alcohol **11** [6] were commercially available or synthesized by the reported procedure.

3. Experimental Details

(A) General procedure for difluoromethylthiolation of enamines 3, indoles 5 and pyrroles 7.

To a mixture of enamine **3**, indole **5** or pyrrole **7** (0.2 mmol) and Cu(I)Br (0.04 mmol) in 1,4-dioxane (2.5 mL), reagent **2a** (0.4 mmol) or **2d** (0.4 mmol) was added at room temperature under N_2. The resulting mixture was stirred at room temperature for 5 h and then filtered through a short plug of celite. The filtrate was concentrated under reduced pressure and purified through flash column chromatography on silica gel (ethyl acetate/hexane) to afforded the target product.

(B) General procedure for difluoromethylthiolation of *β*-keto esters 9

To a mixture of *β*-keto ester **9** (0.2 mmol) Cu(I)Br (0.04 mmol) and K_2CO_3 (0.08 mmol) in 1,4-dioxane (2.5 mL), reagent **2d** (0.4 mmol) was added at room temperature under N_2. The resulting mixture was stirred at room temperature for 24 h and then filtered through a short plug of celite. The filtrate was concentrated under reduced pressure and purified through flash column chromatography on silica gel (ethyl acetate/hexane) to afforded the target product.

(C) General procedure for reaction of ally alcohols 11 with reagent 2.

To a mixture of ally alcohol (0.20 mmol) and CuF_2 (0.04 mmol) in DMAc (2.5 mL), reagent **2a** (0.4 mmol) was added at room temperature under N_2. After stirring at room temperature for 24 h, water (2.0 mL) was added to the mixture. The resulting mixture was extracted with ethyl acetate, the organic phase was washed with water 2 times and saturated sodium bicarbonate solution over 5 times to remove the starting material and brine once then dried over magnesium sulfate. The solvent was removed by rotary evaporation and purified through flash column chromatography on silica gel (ethyl acetate/hexane) to afforded the target product.

(D) General procedure for Enamine method: Two step, one-pot synthesis of difluoromethylthiolatiolated *β*-keto esters and 1,3-diketones 10p-y.

To a mixture of enamine **3p-y** (0.2 mmol) and Cu(I)Br (0.04 mmol) in 1,4-dioxane (2.5 mL), reagent **2a** (0.4 mmol) or **2d** (0.4 mmol) was added at room temperature under N_2. After stirring at room temperature for 5 h, 1N-HCl aq (1.0 mL) was added to the reaction mixture. The resulting mixture was continuously stirred for 12 h (for 24 h when **3q** or **3r** was a starting material), then extracted with ethyl acetate, the organic phase was washed with water 2 times and brine once then dried over magnesium sulfate. The solvent was removed by rotary evaporation and purified through flash column chromatography on silica gel (ethyl acetate/hexane) to afforded the target product.

(E) General procedure for reaction of *β*-keto esters with reagent 2.

To a mixture of *β*-keto esters (0.2 mmol), CuF_2 (0.04 mmol) and Potassium carbonate (0.04 mmol) in DMAc (2.5 mL), reagent **2d** (0.4 mmol) was added at room temperature under N_2. After stirring at room temperature for 18 h or 16 h. The resulting mixture was then extracted with ethyl acetate, the organic phase was washed with water and brine once then dried over magnesium sulfate. The solvent was removed by rotary

evaporation and purified through flash column chromatography on silica gel (DCM/hexane) to afforded the target product.

(F) General procedure for reaction of silyl enol ether 9d with reagent 2.

To a mixture of silyl enol ether (0.20 mmol) and CuBr (0.04 mmol) in 1,4-dioxane (2.5 mL), reagent **2d** (0.4 mmol) was added at room temperature under N_2. After stirring at room temperature for 18 h, 1 N HClaq (1.0 mL) was added to the mixture and stirring at room temperature for 30 min. The resulting mixture was extracted with ethyl acetate, the organic phase was washed with water 2 times and brine once then dried over magnesium sulfate. The solvent was removed by rotary evaporation and purified through flash column chromatography on silica gel (DCM/hexane) to afforded the target product.

Table S1. Stability of Reagent 2a-d

run	reagent	residual rate (%)[b]			
		1 h	5 h	10 h	24 h
1	**2a**	91	66	42	0
2	**2b**	95	97	92	52
3	**2c**	94	2	0	0
4	**2d**	99	100	100	100

[a] Conditions: **2** (0.1 mmol), rt, under air atmosphere. [b] Residual rate were determined by ^{19}F NMR spectroscopy with PhF as an internal standard.

Reagent **2a-d** (0.1 mmol, respectively) was put into a glass tube at room temperature in air atmosphere. After 1 h, 5 h, 10 h and 24 h respectively, $CDCl_3$ and PhF (0.2 mmol: an internal standard) were added to the resulting reagent. The residual rate of reagent was immediately analyzed by ^{19}F NMR spectroscopy.

Scheme S2. Reaction of 3m with Reagent 2a under Different conditions

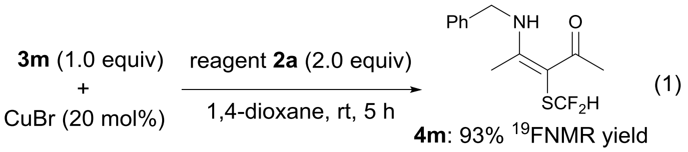

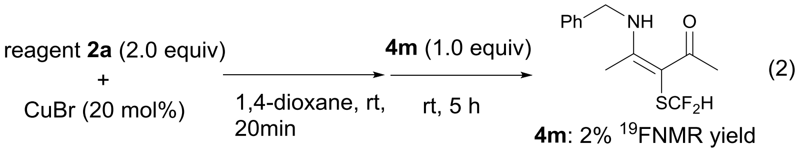

(1): To a mixture of enamine **3m** (0.1 mmol) and Cu(I)Br (2.9 mg, 0.02 mmol) in 1,4-dioxane (1.3 mL), reagent **2a** (174.5 mg, 0.2 mmol) was added at room temperature under N_2. The resulting mixture was stirred at room temperature for 5 h and then filtered through a short plug of celite. The filtrate was concentrated under reduced pressure then analyzed by ^{19}F NMR spectroscopy with PhF (18.4 μL, 0.2 mmol) as an internal standard.

(2): The mixture of Cu(I)Br (2.9 mg, 0.02 mmol), **2a** (174.5 mg, 0.2 mmol) and 1,4-dioxane (1.3 mL) was stirred at room temperature for 20 min under N_2. **3m** was added to the resulting mixture and then stirred for 5 h. The reaction mixture was filtered through a short plug of celite. The filtrate was concentrated under reduced pressure then analyzed by ^{19}F NMR spectroscopy with PhF (18.4 μL, 0.2 mmol) as an internal standard.

Scheme S3. GCMS Analysis of Difluoromethylthiolation of 3m with 2d

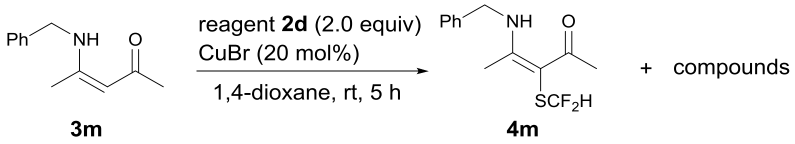

To a mixture of enamine **3m** (18.9 mg, 0.1 mmol) and Cu(I)Br (2.8 mg, 0.02 mmol) in 1,4-dioxane (1.3 mL), reagent **2d** (104.7 mg, 0.2 mmol) was added at room temperature under N_2. The resulting mixture was stirred at room temperature for 5 h and then filtered through a short plug of celite. The filtrate 0.1 mL was added into CH_2Cl_2 1.5 mL then the resulting mixture 5 μL was analyzed by GCMS.

GCMS analytical data of the reaction mixture of 3m, CuBr and reagent 2d.

All peak data (Peak 1-3)

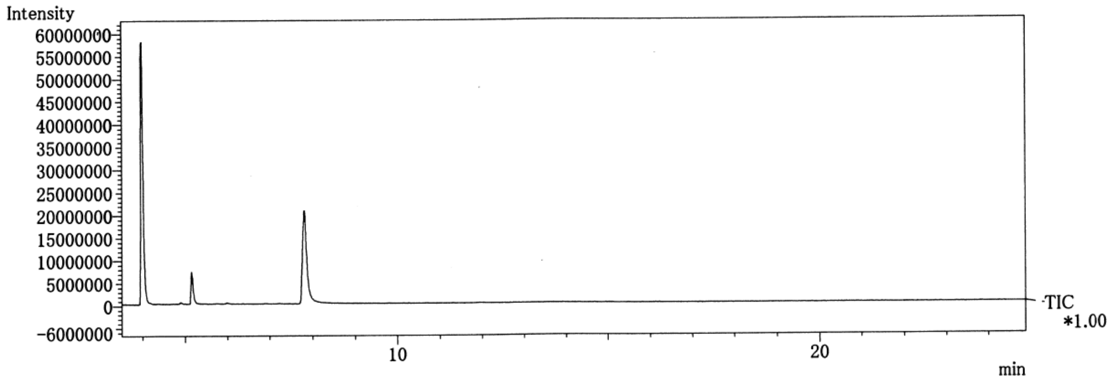

Peak 1 data (RT=3.98 min)

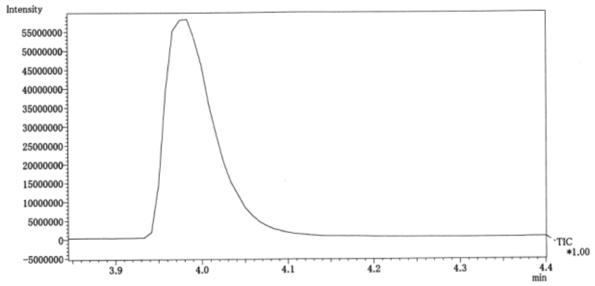

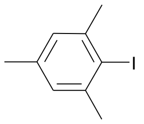

Chemical Formula: $C_9H_{11}I$
Exact Mass: 245.9905

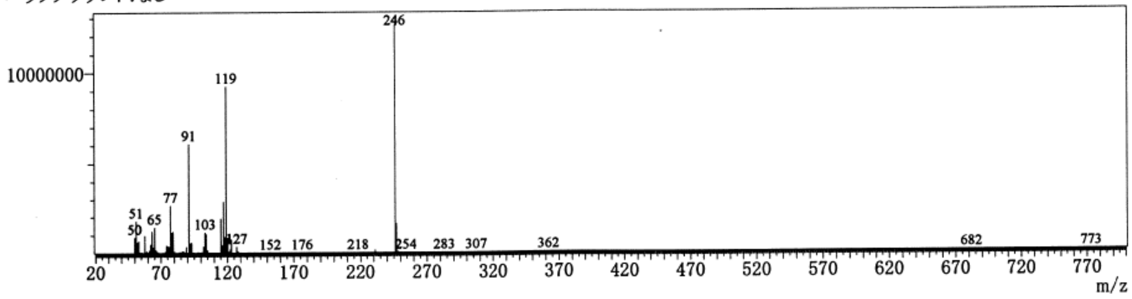

Peak 2 data (RT=5.15 min)

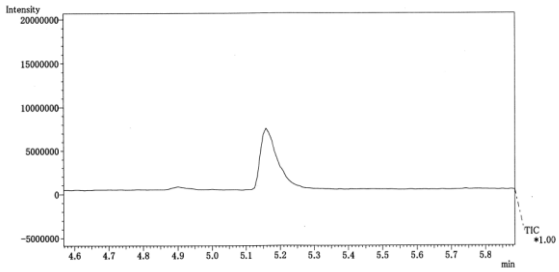

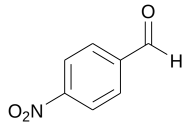

Chemical Formula: $C_7H_5NO_3$
Exact Mass: 151.0269

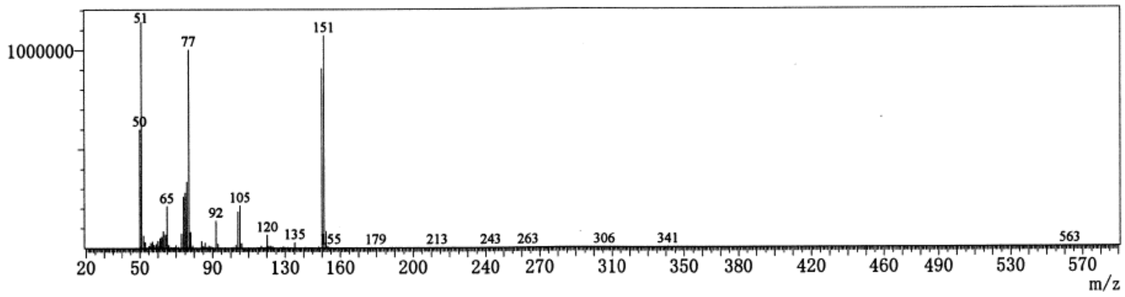

Peak 3 data (RT=7.80 min)

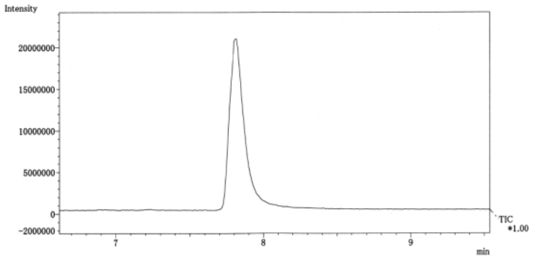

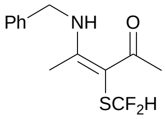

4m
Chemical Formula: $C_{13}H_{15}F_2NOS$
Exact Mass: 271.0842

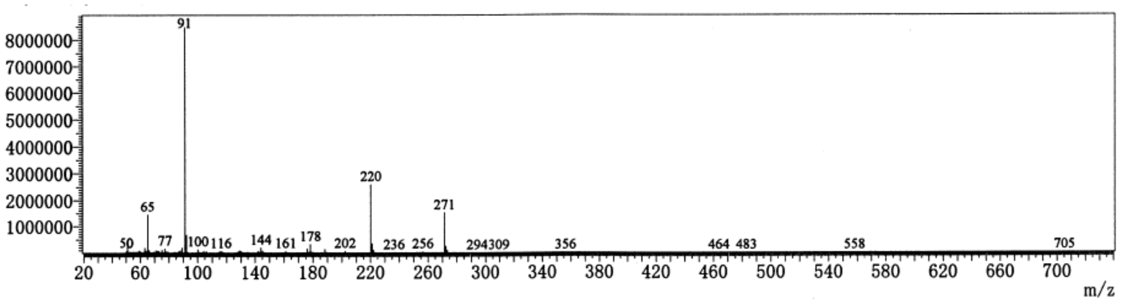

GCMS analytical data of authentic sample Mesityl-I and *p*-NO_2PhC(O)H under same measurement condition.

All peak data

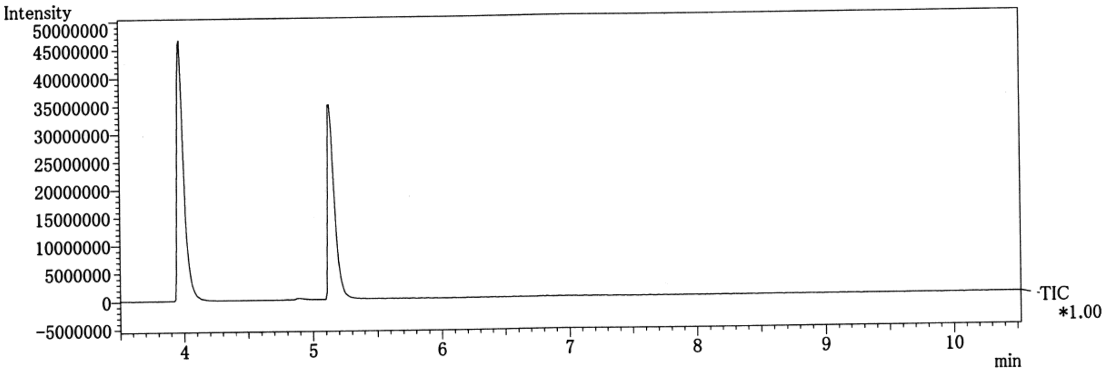

Mesityl-I peak (RT=3.97 min)

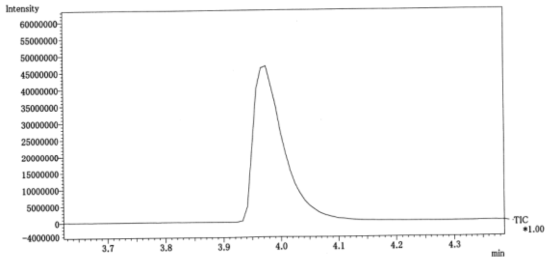

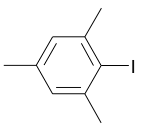

Chemical Formula: $C_9H_{11}I$
Exact Mass: 245.9905

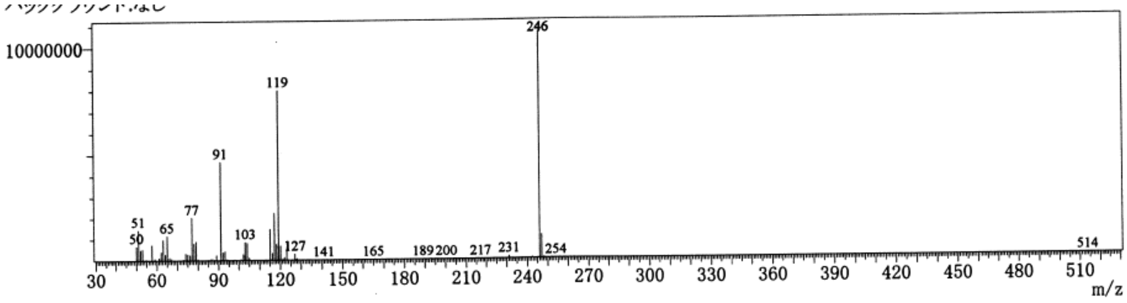

p-NO_2PhC(O)H peak (RT=5.13 min)

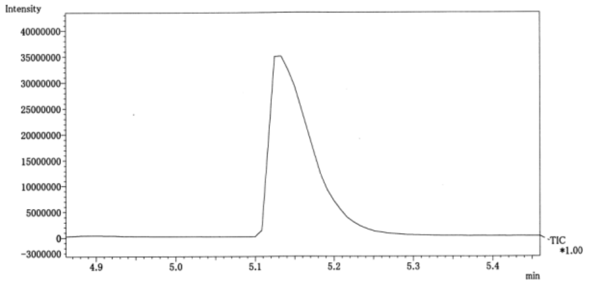

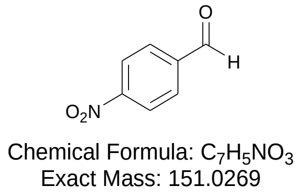

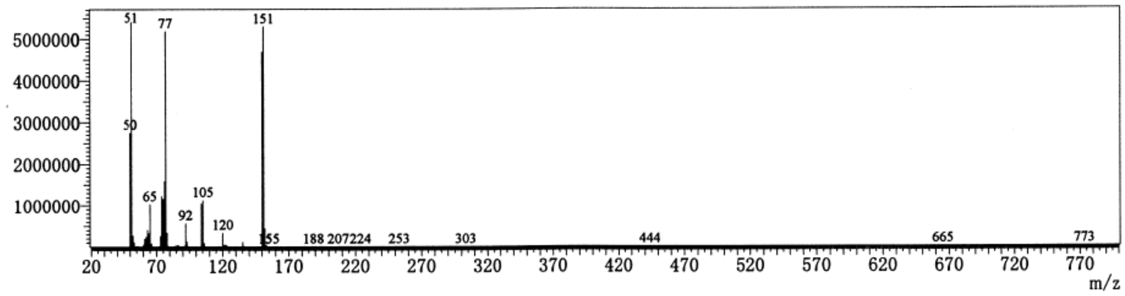

Instrument conditions GCMS (SHIMADZU GCMS-QP5050A)

(GC)

Colum: HYDRODEX-B-TBDAc 25 m×0.25 mm, film thickness: 0.25 μm

Carrier gas: helium

The gradient of temperature: 50~230 °C (40°C/min)

Flow rate: 2 mL/min

(MS)

Temperature of interface: 230 °C

Scan (m/z): 50 ~ 800

4. Optimization of Conditions

Table S2. Optimization of the Difluoromethylthiolation of Enamine 3a

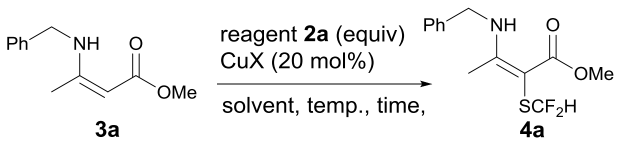

run	**2a** (equiv)	CuX	temp. (°C)	time (h)	solvent	yield (%)[b]
1	2.0	CuF_2	0→rt	5	DMAc	50
2	2.0	CuCl	rt	5	1,4-dioxane	82
3	2.0	$CuCl_2$	rt	5	1,4-dioxane	71
4	2.0	CuBr	rt	5	1,4-dioxane	**94**
5	2.0	CuI	rt	5	1,4-dioxane	82
6	2.0	CuF_2	rt	5	1,4-dioxane	78
7	2.0	CuOAc	rt	5	1,4-dioxane	73
8	2.0	$Cu(OAc)_2$	rt	5	1,4-dioxane	81
9	2.0	CuBr	rt	5	THF	74
10	2.0	CuBr	rt	5	MeCN	38
11	2.0	CuBr	rt	5	DMF	48
12	2.0	CuBr	rt	5	DMAc	42
13	2.0	CuBr	rt	5	DMSO	16
14	2.0	CuBr	rt	5	PhMe	60
15	2.0	CuBr	rt	5	EtOH	77
16	1.1	CuBr	rt	5	1,4-dioxane	58
17	2.0	-	rt	5	1,4-dioxane	0
18	2.0	CuBr	rt	1	1,4-dioxane	89
19	2.0	CuBr	rt	10	1,4-dioxane	91
20	2.0	CuBr	rt	24	1,4-dioxane	84
22	2.0	CuBr	50	5	1,4-dioxane	78
23[c]	2.0	CuBr	rt	5	1,4-dioxane	94

[a] Reaction conditions: **3a** (0.1 mmol), **2a** (0.2 mmol) and CuX (20 mol%) in solvent (1.25 mL) under N_2 atmosphere. [b] ^{19}F NMR yields with PhF as an internal standard. [c] 100 mol% of CuBr was used.

Table S3. Optimization of the Difluoromethylthiolation of Pyrrole 7a

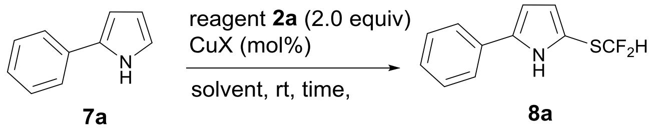

run	CuX (mol%)	solvent	time (h)	yield (%)[b]
1	CuCl (20)	1,4-dioxane	24	51
2	CuCl (50)	1,4-dioxane	24	46
3	CuCl (100)	1,4-dioxane	24	42

4	-	1,4-dioxane	24	0
5[c]	CuCl (20)	1,4-dioxane	24	38
6	CuBr (20)	1,4-dioxane	24	**58**
7	CuF_2 (20)	DMAc	24	27
8	CuBr (20)	1,4-dioxane	5	**58**
9[d]	CuBr (20)	1,4-dioxane	5	46
10[e]	CuBr (20)	1,4-dioxane	5	47
11[f]	CuBr (20)	1,4-dioxane	5	**57**

[a] Reaction conditions: **7a** (0.1 mmol), **2a** (0.2 mmol) in solvent (1.25 mL) under N_2 atmosphere. [b] ^{19}F NMR yields with PhF as an internal standard. [c] $PhNMe_2$ (20 mol%) was added. [d] **2b** (0.2 mmol) was used instead of **2a**. [e] **2c** (0.2 mmol) was used instead of **2a**. [f] **2d** (0.2 mmol) was used instead of **2a**.

Table S4. Optimization of the Reaction Conditions of Allyl Alcohol 11a

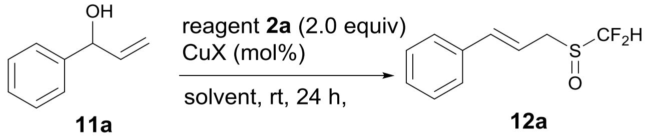

run	CuX	solvent	yield (%)[b]
1	CuBr	1,4-dioxane	27
2	CuF_2	DMAc	**50**

[a] Reaction conditions: **11a** (0.1 mmol), **2a** (0.2 mmol) in solvent (1.25 mL) under N_2 atmosphere. [b] ^{19}F NMR yields with PhF as an internal standard.

5. Analytical Data

(*E*)-Methyl 3-(benzylamino)-2-[(difluoromethyl)thio]but-2-enoate (4a)

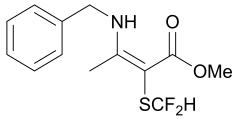

4a was prepared according to the **General procedure (A).**

Yellow oil (46.8 mg, 81%). Eluent: ethyl acetate/hexane =1/7, Rf = 0.4.

^1H NMR (300 MHz, $CDCl_3$): δ ppm 10.61 (brs, 1H), 7.37-7.33 (m, 5H), 6.53 (t, *J* = 58.5 Hz, 1H), 4.53 (d, *J* = 5.7 Hz, 2H), 3.74 (s, 3H), 2.39 (s, 3H). ^{19}F NMR (282 MHz,

$CDCl_3$): δ ppm -96.85 (d, *J* = 58.3 Hz, 2F). ^{13}C NMR (125 MHz, $CDCl_3$): δ ppm 171.23, 170.94, 137.03, 128.96, 127.76, 126.85, 121.56 (t, *J* = 274 Hz), 76.29 (t, *J* = 3.75 Hz), 51.51, 48.25, 17.89. IR (neat): ν = 3263, 2947, 1638, 1580, 1441, 1255, 1065, 1027 cm^{-1}. MS (EI): 287 (M). HRMS (ESI) $C_{13}H_{15}F_2NNaO_2S$ (M+Na) for Calcd: 310.0689, Found: 310.0695.

(*E*)-Methyl 3-[(4-methoxybenzyl) amino]-2-[(difluoromethyl)thio]but-2-enoate (4b)

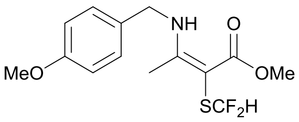

4b was prepared according to the **General procedure (A).**
Yellow solid (49.6 mg, 78%). Mp: 48 ~ 49 ºC. Eluent: ethyl acetate/hexane =1/7, Rf = 0.5.
^1H NMR (300 MHz, $CDCl_3$): δ ppm 10.52 (brs, 1H), 7.19 (d, *J* = 8.1 Hz, 2H), 6.89 (d, *J* = 8.4 Hz, 2H), 6.53 (t, *J* = 58.2 Hz, 1H), 4.45 (d, *J* = 5.4 Hz, 2H), 3.81 (s, 3H), 3.72 (s, 3H), 2.40 (s, 3H). ^{19}F NMR (282 MHz, $CDCl_3$): δ ppm -96.85 (d, *J* = 58.4 Hz, 2F). ^{13}C NMR (125 MHz, $CDCl_3$): δ ppm 171.22, 170.76, 159.17, 128.93, 128.31, 121.60 (t, *J* = 274 Hz), 114.35, 76.04 (t, *J* = 3.75 Hz), 55.27, 51.48, 47.84, 17.92. IR (KBr): ν = 3129, 2951, 2057, 1882, 1580, 1514, 1444, 1242, 1062 cm^{-1}. MS (ESI): 335 ($M+NH_4$). HRMS (ESI) $C_{14}H_{17}F_2NNaO_3S$ (M+Na) for Calcd: 340.0795, Found: 340.0791.

(*E*)-Methyl 3-[(4-bromobenzyl)amino]-2-[(difluoromethyl)thio]but-2-enoate (4c)

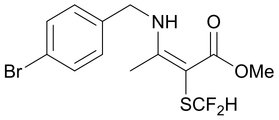

4c was prepared according to the **General procedure (A).**
Pale yellow solid (62.0 mg, 85%: using **2a**, 62.1 mg, 85%: using **2d**). Mp: 77-78 ºC. Eluent: ethyl acetate/hexane =1/7, Rf = 0.3.
^1H NMR (300 MHz, $CDCl_3$): δ ppm 10.60 (brs, 1H), 7.49 (d, *J* = 8.1 Hz, 2H), 7.14 (d, *J* = 7.8 Hz, 2H), 6.53 (t, *J* = 58.2 Hz, 1H), 4.48 (d, *J* = 5.4 Hz, 2H), 3.74 (s, 3H), 2.37 (s, 3H). ^{19}F NMR (282 MHz, $CDCl_3$): δ ppm -96.86 (d, *J* = 58.7 Hz, 2F). ^{13}C NMR (125 MHz, $CDCl_3$): δ ppm 171.26, 170.83, 136.15, 132.11, 128.53, 121.71, 121.41 (t, *J* = 275 Hz), 76.79 (t, *J* = 3.75 Hz), 51.60, 47.62, 17.90. IR (KBr): ν = 3198, 3125, 2954, 1638, 1573, 1487, 1444, 1317, 1274, 1069 cm^{-1}. MS (ESI): 365 (M+H). HRMS (ESI) $C_{13}H_{14}BrF_2NNaO_2S$ (M+Na) for Calcd: 387.9794, Found: 387.9787.

(*E*)-Methyl 3-(butylamino)-2-[(difluoromethyl)thio]but-2-enoate (4d)

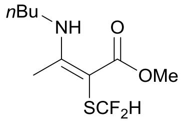

4d was prepared according to the **General procedure (A).**

Yellow oil (42.8 mg, 84%). Eluent: ethyl acetate/hexane =1/7, Rf = 0.6.

^{1}H NMR (300 MHz, $CDCl_3$): δ ppm 10.26 (brs, 1H), 6.51 (t, *J* = 58.8 Hz, 1H), 3.73 (s, 3H), 3.31 (d, *J* = 6.0 Hz, 2H), 2.37 (s, 3H), 1.64-1.59 (m, 2H), 1.47-1.42 (m, 2H), 0.96 (t, *J* = 7.2 Hz, 3H). ^{19}F NMR (282 MHz, $CDCl_3$): δ ppm -97.9 (d, *J* = 57.5 Hz, 2F). ^{13}C NMR (125 MHz, $CDCl_3$): δ ppm 171.38, 170.78, 121.73 (t, *J* = 274 Hz), 74.89 (t, *J* = 3.75 Hz), 51.41, 44.30, 31.75, 20.00, 17.74, 13.69. IR (neat): ν = 3129, 2958, 2877, 1638, 1588, 1448, 1248, 1065 cm^{-1}. MS (ESI): 292 (M+K). HRMS (ESI) $C_{10}H_{17}F_2NNaO_2S$ (M+Na) for Calcd: 276.0846, Found: 276.0852.

(*E*)-Methyl 3-(phenylamino)-2-[(difluoromethyl)thio]but-2-enoate (4e)

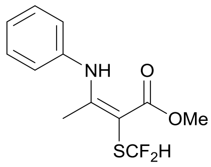

4e was prepared according to the **General procedure (A).**

Yellow solid (47.6 mg, 87%). Mp: 46-47 ºC. Eluent: ethyl acetate/hexane =1/7, Rf = 0.5.

^{1}H NMR (300 MHz, $CDCl_3$): δ ppm 10.84 (brs, 1H), 7.39-7.36 (m, 2H), 7.29-7.26 (m, 1H), 7.12 (d, *J* = 6.9 Hz, 2H), 6.61 (t, *J* = 58.5 Hz, 1H), 3.79 (s, 3H), 2.34 (s, 3H). ^{19}F NMR (282 MHz, $CDCl_3$): δ ppm -96.24 (d, *J* = 58.4 Hz, 2F). ^{13}C NMR (125 MHz, $CDCl_3$): δ ppm 171.08, 169.16, 138.41, 129.27, 126.69, 15.77, 121.50 (t, *J* = 274 Hz), 76.68 (t, *J* = 3.75 Hz), 51.76, 19.51. IR (KBr): ν = 3156, 2954, 1638, 1569, 1437, 1309, 1248, 1065, 1011 cm^{-1}. MS (ESI): 305 (M+MeOH). HRMS (ESI) $C_{12}H_{13}F_2NNaO_2S$ (M+Na) for Calcd: 296.0533, Found: 296.0535.

(*E*)-Methyl 3-(*p*-tolylamino)-2-[(difluoromethyl)thio]but-2-enoate (4f)

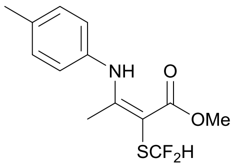

4f was prepared according to the **General procedure (A).**

Pale yellow solid (51.6 mg, 90%). Mp: 58-59 ºC. Eluent: ethyl acetate/hexane =1/7, Rf = 0.5.

^{1}H NMR (300 MHz, $CDCl_3$): δ ppm 11.75 (brs, 1H), 7.18 (d, *J* = 7.5 Hz, 2H), 7.00 (d, *J* = 7.5 Hz, 2H), 6.60 (t, *J* = 58.2 Hz, 1H), 3.79 (s, 3H), 2.36 (s, 3H), 2.31 (s, 3H). ^{19}F NMR (282 MHz, $CDCl_3$): δ ppm -96.26 (d, *J* = 58.4 Hz, 2F). ^{13}C NMR (125 MHz, $CDCl_3$): δ ppm 171.11, 169.47, 136.66, 135.79, 129.83, 125.71, 121.58 (t, *J* = 275 Hz), 78.13 (t, *J* = 3.75 Hz), 51.70, 20.95, 19.45. IR (KBr): ν = 3168, 3098, 2950, 1627, 1565, 1433, 1309, 1255, 1015 cm^{-1}. MS (ESI): 310 (M+Na). HRMS (ESI) $C_{13}H_{15}F_2NNaO_2S$ (M+Na) for Calcd: 310.0689, Found: 310.0695.

(*E*)-Methyl 3-(benzylamino)-3-phenyl-2-[(difluoromethyl)thio]acrylate (4g)

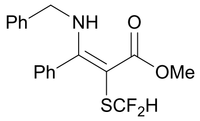

4g was prepared according to the **General procedure (A).**

Colorlss oil (58.7 mg, 84%). Eluent: ethyl acetate/hexane =1/7, Rf = 0.4.

^{1}H NMR (300 MHz, $CDCl_3$): δ ppm 10.44 (brs, 1H), 7.42 (brs, 3H), 7.31-7.28 (m, 2H), 7.12 (brs, 5H), 6.42 (t, *J* = 58.2 Hz, 1H), 4.13 (d, *J* = 5.7 Hz, 2H), 3.80 (s, 3H). ^{19}F NMR (282 MHz, $CDCl_3$): δ ppm -97.26 (d, *J* = 58.4 Hz, 2F). ^{13}C NMR (125 MHz, $CDCl_3$): δ ppm 172.19, 171.24, 137.56, 134.28, 129.02, 128.73, 128.37, 127.72, 127.61, 126.99, 121.21 (t, *J* = 275 Hz), 78.01 (t, *J* = 3.75 Hz), 51.70, 49.44. IR (neat): ν = 3233, 3033, 2945, 1646, 1565, 1444, 1263, 1158, 1065 cm^{-1}. MS (ESI): 372 (M+Na). HRMS (ESI) $C_{18}H_{17}F_2NNaO_2S$ (M+Na) for Calcd: 372.0846, Found: 372.0850.

(*E*)-Ethyl 3-(benzylamino)-3-phenyl-2-[(difluoromethyl)thio]acrylate (4h)

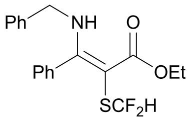

4h was prepared according to the **General procedure (A).**

Colorless oil (58.7 mg, 81%). Eluent: ethyl acetate/hexane =1/7, Rf = 0.5.

^{1}H NMR (300 MHz, $CDCl_3$): δ ppm 10.43 (brs, 1H), 7.42 (brs, 3H), 7.30-7.28 (m, 3H), 7.12 (brs, 4H), 6.43 (t, *J* = 58.2 Hz, 1H), 4.26 (q, *J* = 6.9 Hz, 2H), 4.12 (d, *J* = 5.7 Hz, 2H), 1.34 (t *J* = 6.9 Hz, 3H). ^{19}F NMR (282 MHz, $CDCl_3$): δ ppm -97.15 (d, *J* = 58.3 Hz, 2F). ^{13}C NMR (125 MHz, $CDCl_3$): δ ppm 171.97, 170.82, 137.67, 134.44, 128.96, 128.72, 128.36, 127.74, 127.58, 127.02, 121.33 (t, *J* = 274 Hz), 78.40 (t, *J* = 3.75 Hz),

60.36, 49.42, 14.42. IR (neat): ν = 3229, 2981, 1642, 1565, 1433, 1255, 1158, 1062 cm^{-1}. MS (ESI): 386 (M+Na). HRMS (ESI) $C_{19}H_{19}F_2NNaO_2S$ (M+Na) for Calcd: 386.1002, Found: 386.1016.

(*E*)-Methyl 3-(benzylamino)-3-(2-methoxyphenyl)-2-[(difluoromethyl)thio]acrylate (4i)

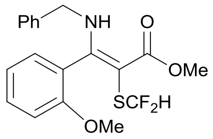

4i was prepared according to the **General procedure (A).**

White solid (57.5 mg, 76%). Mp: 70-71 ºC. Eluent: ethyl acetate/hexane =1/7, Rf = 0.3. ^{1}H NMR (300 MHz, $CDCl_3$): δ ppm 10.40 (brs, 1H), 7.41 (brs, 1H), 7.29-7.26 (m, 3H), 7.12 (d, *J* = 6.3 Hz, 2H), 7.02 (brs, 2H), 6.93 (d, *J* = 6.3 Hz, 1H), 6.45 (t, *J* = 58.5 Hz, 1H), 4.14 (s, 2H), 3.78 (s, 3H), 3.74 (s, 3H). ^{19}F NMR (282 MHz, $CDCl_3$): δ ppm -96.6 (dd, *J* = 58.4, 242 Hz, 1F), -96.6 (dd, *J* = 58.4, 242 Hz, 1F). ^{13}C NMR (125 MHz, $CDCl_3$): δ ppm 171.25, 169.67, 155.50, 137.50, 130.75, 128.96, 128.58, 127.50, 127.27, 123.31, 122.73 (t, *J* = 273 Hz), 120.46, 110.69, 78.41 (t, *J* = 3.75 Hz), 55.22, 51.60, 49.40. IR (KBr): ν = 3233, 2947, 1642, 1565, 1499, 1437, 1263, 1158, 1058 cm^{-1}. MS (ESI): 380 (M+H). HRMS (ESI) $C_{20}H_{21}F_2NNaO_3S$ (M+Na) for Calcd: 402.0951, Found: 402.0949.

(*E*)-Methyl 3-(benzylamino)-3-(3-methoxyphenyl)-2-[(difluoromethyl)thio]acrylate (4j)

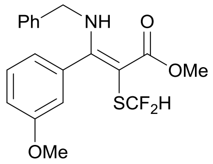

4j was prepared according to the **General procedure (A).**

Pale yellow oil (60.9 mg, 80%). Eluent: ethyl acetate/hexane =1/7, Rf = 0.4.
^{1}H NMR (300 MHz, $CDCl_3$): δ ppm 10.41 (brs, 1H), 7.33-7.26 (m, 4H), 7.13-7.11 (m, 2H), 6.95 (d, *J* = 6.6 Hz, 1H), 6.72 (d, *J* = 8.1, 1H), 6.64 (s, 1H), 6.45 (t, *J* = 58.5 Hz, 1H), 4.14 (s, 2H), 3.80 (s, 3H), 3.74 (s, 3H). ^{19}F NMR (282 MHz, $CDCl_3$): δ ppm -95.6 (dd, *J* = 58.4, 13.8 Hz, 2F). ^{13}C NMR (125 MHz, $CDCl_3$): δ ppm 171.86, 171.15, 159.39, 137.68, 135.40, 129.55, 128.68, 127.54, 126.95, 121.23 (t, *J* = 275 Hz), 119.77, 114.82, 113.07, 77.84 (t, *J* = 3.75 Hz), 55.14, 51.65, 49.35. IR (neat): ν = 3233, 2951, 1646, 1565, 1460, 1437, 1267, 1224, 1042 cm^{-1}. MS (ESI): 380 (M+H). HRMS (ESI)

$C_{20}H_{21}F_2NNaO_3S$ (M+Na) for Calcd: 402.0951, Found: 402.0960

(*E*)-Methyl 3-(benzylamino)-3-(4-methoxyphenyl)-2-[(difluoromethyl)thio]acrylate (4k)

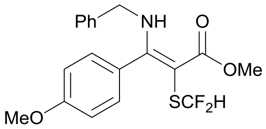

4k was prepared according to the **General procedure (A).**

White solid (64.5 mg, 85%). Mp: 57-58 ºC. Eluent: ethyl acetate/hexane =1/7, Rf = 0.4. ^{1}H NMR (300 MHz, $CDCl_3$): δ ppm 10.44 (brs, 1H), 7.33-7.26 (m, 3H), 7.13-7.04 (m, 4H), 6.93 (d, *J* = 8.4 Hz, 2H), 6.43 (t, *J* = 58.2 Hz, 1H), 4.16 (d, *J* = 5.7 Hz, 2H), 3.84 (s, 3H), 3.79 (s, 3H). ^{19}F NMR (282 MHz, $CDCl_3$): δ ppm -97.28 (d, *J* = 58.4 Hz, 2F). ^{13}C NMR (125 MHz, $CDCl_3$): δ ppm 172.30, 171.28, 159.92, 137.71, 129.24, 128.72, 127.55, 126.96, 126.46, 121.28 (t, *J* = 275 Hz), 113.71, 78.19 (t, *J* = 3.75 Hz), 55.19, 51.67, 49.41. IR (KBr): ν = 3233, 2951, 2843, 1642, 1565, 1441, 1252, 1162, 1062 cm^{-1}. MS (ESI): 380 (M+H). HRMS (ESI) $C_{19}H_{19}F_2NNaO_3S$ (M+Na) for Calcd: 402.0951, Found: 402.0961

(*E*)-Methyl 3-(benzylamino)-3-(4-bromophenyl)-2-[(difluoromethyl)thio]acrylate (4l)

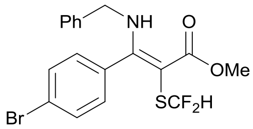

4l was prepared according to the **General procedure (A).**

White solid (71.7 mg, 84%). Mp: 72-73 ºC. Eluent: ethyl acetate/hexane =1/7, Rf = 0.3. ^{1}H NMR (300 MHz, $CDCl_3$): δ ppm 10.42 (brs, 1H), 7.55 (d, J = 8.4 Hz, 2H), 7.32-7.26 (m, 3H), 7.09 (d, J = 6.0 Hz, 2H), 7.00 (d, J = 8.4 Hz, 2H), 6.43 (t, *J* = 58.8 Hz, 1H), 4.13 (d, *J* = 5.7 Hz, 2H), 3.80 (s, 3H). ^{19}F NMR (282 MHz, $CDCl_3$): δ ppm -97.40 (d, *J* = 57.2 Hz, 2F). ^{13}C NMR (125 MHz, $CDCl_3$): δ ppm 171.06, 171.01, 137.37, 133.06, 131.65, 129.53, 128.80, 127.70, 126.87, 123.35, 120.82 (t, *J* = 275 Hz), 78.25 (t, *J* = 3.75 Hz), 51.78, 49.41. IR (KBr): ν = 3237, 3033, 2951, 1646, 1565, 1441, 1263, 1154, 1065 cm^{-1}. MS (ESI): 450 (M+Na). HRMS (ESI) $C_{18}H_{16}BrF_2NNaO_2S$ (M+Na) for Calcd: 449.9951, Found: 449.9948

(*E*)-4-(Benzylamino)-3-[(difluoromethyl)thio]pent-3-en-2-one (4m)

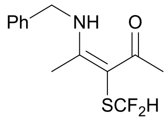

4m was prepared according to the **General procedure (A).**

Yellow oil (48.7 mg, 90%). Eluent: ethyl acetate/hexane =1/7, Rf = 0.3.

^{1}H NMR (300 MHz, $CDCl_3$): δ ppm 12.86 (brs, 1H), 7.37-7.30 (m 5H), 6.50 (t, *J* = 56.7 Hz, 1H), 4.55 (d, *J* = 5.7 Hz, 2H), 2.43 (s, 3H), 2.37 (s, 3H). ^{19}F NMR (282 MHz, $CDCl_3$): δ ppm -95.54 (dd, *J* = 57.5, 10.9 Hz, 2F). ^{13}C NMR (125 MHz, $CDCl_3$): δ ppm 199.83, 171.75, 136.56, 128.99, 127.81, 126.87, 121.40 (t, *J* = 274 Hz), 88.33 (t, *J* = 3.75 Hz), 48.28, 29.45, 18.02. IR (neat): ν = 3033, 2958, 1577, 1452, 1356, 1263, 1031 cm^{-1}. MS (ESI): 272 (M+H). HRMS (ESI) $C_{13}H_{16}F_2NOS$ (M+H) for Calcd: 272.0921, Found: 272.0925.

(*E*)-3-(Benzylamino)-1-phenyl-2-[(difluoromethyl)thio]but-2-en-1-one (4n)

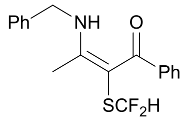

4n was prepared according to the **General procedure (A).**

Yellow solid (61.5 mg, 92%). Mp: 59-60 ºC. Eluent: ethyl acetate/hexane =1/7, Rf = 0.3.

^{1}H NMR (300 MHz, $CDCl_3$): δ ppm 13.01 (brs, 1H), 7.40-7.34 (m, 10H), 6.25 (t, *J* = 56.7 Hz, 1H), 4.64 (d, *J* = 5.4 Hz, 2H), 2.49 (s, 3H). ^{19}F NMR (282 MHz, $CDCl_3$): δ ppm -96.68 (dd, *J* = 57.5, 13.8 Hz, 2F). ^{13}C NMR (125 MHz, $CDCl_3$): δ ppm 197.41, 173.63, 142.46, 136.15, 129.03, 128.77, 127.91, 127.44, 127.30, 126.99, 121.00 (t, *J* = 274 Hz), 87.54 (t, *J* = 3.75 Hz), 48.51, 18.41. IR (KBr): ν = 3029, 2945, 1577, 1553, 1456, 1317, 1294, 1131, 1054 cm^{-1}. MS (ESI): 351 ($M+NH_4$). HRMS (ESI) $C_{18}H_{17}F_2NNaOS$ (M+Na) for Calcd: 356.0897, Found: 356.0895.

3-(Phenylamino)-2-[(difluoromethyl)thio]cyclohex-2-enone (4o)

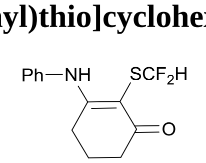

4o was prepared according to the **General procedure (A).**

Brown oil (13.2 mg, 25%). Eluent: ethyl acetate/hexane =1/1, Rf = 0.4.

^{1}H NMR (300 MHz, $CDCl_3$): δ ppm 8.33 (brs, 1H), 7.43-7.33 (m, 3H), 7.16 (d, *J* = 7.2

Hz, 2H), 6.75 (t, *J* =58.8 Hz, 1H), 2.63-2.53 (m, 4H), 1.97 (t, *J* = 5.7 Hz, 2H). ^{19}F NMR (282 MHz, $CDCl_3$): δ ppm -93.55 (d, *J* = 59.5 Hz, 2F). ^{13}C NMR (125 MHz, $CDCl_3$): δ ppm 192.84, 169.14, 136.98, 129.54, 127.32, 125.90, 120.27 (t, *J* = 275 Hz), 94.38 (t, *J* = 2.5 Hz), 37.15, 28.02, 21.01. IR (neat): ν = 3319, 3245, 2951, 1634, 1553, 1390, 1189, 1065 cm^{-1}. MS (ESI): 292 (M+Na). HRMS (ESI) $C_{13}H_{13}F_2NNaOS$ (M+Na) for Calcd: 292.0584, Found: 292.0597.

(*E*)-Ethyl 3-amino-3-phenyl-2-[(difluoromethyl)thio]acrylate (4p)

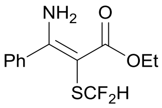

4pwas prepared according to the **General procedure (A).**

White solid (44.8 mg, 83%). Mp: 61-62 ºC. Eluent: ethyl acetate/hexane = 1/9, Rf = 0.3. ^{1}H NMR (300 MHz, $CDCl_3$): δ ppm 9.35 (brs, 1H), 7.44-7.41 (m, 2H), 7.39 -7.32 (m, 3H), 6.50 (t, *J* = 58.5 Hz, 1H), 5.38 (brs, 1H), 4.28 (q, *J* = 7.2 Hz, 2H), 1.35 (t *J* = 6.9 Hz, 3H). ^{19}F NMR (282 MHz, $CDCl_3$): δ ppm -97.08 (d, *J* = 58.4 Hz, 2F). ^{13}C NMR (125 MHz, $CDCl_3$): δ ppm 170.21, 170.12, 137.80, 129.51, 128.22, 127.87, 121.22 (t, *J* = 274 Hz), 79.38 (t, *J* = 3.75 Hz), 60.56, 14.39. IR (KBr): ν = 3408, 3296, 2997, 2954, 1650, 1596, 1479, 1317, 1263, 1027 cm^{-1}. MS (ESI): 296 (M+Na). HRMS (ESI) $C_{12}H_{13}F_2NNaO_2S$ (M+Na) for Calcd: 296.0533, Found: 296.0524.

3-[(Difluoromethy)thio]-1*H*-indole (6a)

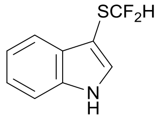

6a was prepared according to the **General procedure (A).**

Orange oil (22.3 mg, 56%). Eluent: ethyl acetate/hexane =1/4, Rf = 0.5.

^{1}H NMR (300 MHz, $CDCl_3$): δ ppm 8.46 (brs, 1H), 7.80 (s, *J* =7.8 Hz, 1H), 7.48 (d, *J* = 2.7 Hz, 1H), 7.42 (d, *J* = 6.6 Hz, 1H), 7.31-7.24 (m, 2H), 6.88 (t, *J* = 57.0 Hz, 1H). ^{19}F NMR (282 MHz, $CDCl_3$): δ ppm -92.6 (d, *J* = 57.2 Hz, 2F). MS (ESI): 200 (M+H). HRMS (ESI) for $C_9H_6F_2NS$ (M-H) Calcd: 198.0189, Found: 198.0185. The product was identified by comparison of the spectral data with the report data. Reference: Zhu D, Gu, Lu, Shen Q. 2015 N-Difluoromethylthiophthalimide: A Shelf-Stable, Electrophilic Reagent for Difluoromethylthiolation. *J. Am. Chem. Soc.* **137**, 10547-10553. (doi:10.1021/jacs.5b03170)

3-[(Difluoromethy)thio]-2-methyl-1*H*-indole (6b)

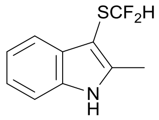

6b was prepared according to the **General procedure (A).**

White solid (36.3 mg, 85%). Mp: 59-60ºC. Eluent: ethyl acetate/hexane =1/4, Rf = 0.4.

^{1}H NMR (300 MHz, $CDCl_3$): δ ppm 8.23 (brs, 1H), 7.71-7.68 (m, 1H), 7.34-7.31 (m, 1H), 7.23-7.19 (m, 2H), 6.63 (t, *J* = 57.3 Hz, 1H), 2.57 (s, 3H). ^{19}F NMR (282 MHz, $CDCl_3$): δ ppm -92.5 (d, *J* = 57.5 Hz, 2F). ^{13}C NMR (125 MHz, $CDCl_3$): δ ppm 142.61, 135.05, 130.79, 122.37, 121.20 (t, *J* = 275 Hz), 121.01, 118.61, 110.65, 93.46 (t, *J* = 3.75 Hz), 12.13. IR (KBr): ν = 3377, 3060, 2966, 1542, 1456, 1406, 1309, 1069, 1023 cm^{-1}. MS (ESI): 231 (M+NH_4). HRMS (ESI) $C_{10}H_8F_2NS$ (M-H) for Calcd: 212.0346, Found: 212.0347.

3-[(Difluoromethy)thio]-2-phenyl-1*H*-indole (6c)

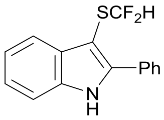

6c was prepared according to the **General procedure (A).**

Yellow solid (30.3 mg, 55%). Mp: 84-85 ºC. Eluent: ethyl acetate/hexane =1/4, Rf = 0.5.

^{1}H NMR (300 MHz, $CDCl_3$): δ ppm 8.56 (brs, 1H), 7.85-7.79 (m, 3H), 7.54-7.42 (m, 4H), 7.32-7.28 (m, 2H), 6.71 (t, *J* = 57.3 Hz, 1H). ^{19}F NMR (282 MHz, $CDCl_3$): δ ppm -92.1 (d, *J* = 57.5 Hz, 2F). ^{13}C NMR (125 MHz, $CDCl_3$): δ ppm 143.26, 135.39, 131.69, 130.97, 129.00, 128.78, 128.63, 123.62, 122.50, 121.42 (t, *J* = 275 Hz), 119.71, 93.72 (t, *J* = 3.75 Hz). IR (KBr): ν = 3419, 2958, 1456, 1398, 1313, 1227, 1058, 1027 cm^{-1}. MS (ESI): 298 (M+Na). HRMS (ESI) $C_{15}H_{10}F_2NS$ (M-H) for Calcd: 274.0502, Found: 274.0490.

3-[(Difluoromethy)thio]-5-methyl-1*H*-indole (6d)

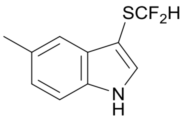

6d was prepared according to the **General procedure (A).**

Yellow solid (32.4 mg, 75%). Mp: 43-44 ºC. Eluent: ethyl acetate/hexane =1/4, Rf = 0.5.

^{1}H NMR (300 MHz, $CDCl_3$): δ ppm 8.39 (brs, 1H), 7.57 (s, 1H), 7.43 (d, J = 2.7 Hz, 1H), 7.31 (d, *J* = 8.1 Hz, 1H), 7.1 (dd, *J* = 8.1, 1.5 Hz, 1H), 6.67 (t, *J* = 57.9 Hz, 1H), 2.49 (s, 3H). ^{19}F NMR (282 MHz, $CDCl_3$): δ ppm -92.8 (d, *J* = 57.5 Hz, 2F). ^{13}C NMR (125 MHz, $CDCl_3$): δ ppm 134.32, 131.85, 130.81, 129.86, 124.87, 121.08 (t, *J* = 276 Hz), 118.80, 111.24, 95.99 (t, *J* = 3.77 Hz), 21.48. IR (KBr): ν = 3403, 3141, 2916, 2370, 1677, 1483, 1456, 1100, 1054, 1035 cm^{-1}. MS (ESI): 236 (M+Na). HRMS (ESI) $C_{10}H_8F_2NS$ (M-H) for Calcd: 212.0346, Found: 212.0355

3-[(Difluoromethy)thio]-7-methyl-1*H*-indole (6e)

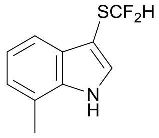

6e was prepared according to the **General procedure (A).**

Yellow solid (32.4 mg, 75%). Mp: 45-46 ºC. Eluent: ethyl acetate/hexane =1/4, Rf = 0.5.

^{1}H NMR (300 MHz, $CDCl_3$): δ ppm 8.40 (brs, 1H), 7.64 (d, *J* = 7.8 Hz, 1H), 7.48 (d, *J* = 2.7 Hz, 1H), 7.18 (t, *J* = 7.5 Hz, 1H), 7.08 (d, *J* = 7.2 Hz, 1H), 6.68 (t, *J* = 57.6 Hz, 1H), 2.51 (s, 3H). ^{19}F NMR (282 MHz, $CDCl_3$): δ ppm -92.6 (d, *J* = 57.2 Hz, 2F). ^{13}C NMR (125 MHz, $CDCl_3$): δ ppm 135.64, 131.49, 129.25, 123.74, 123.19, 121.46, 121.00 (t, *J* = 274 Hz), 120.78, 97.09 (t, *J* = 3.75 Hz), 16.33. IR (KBr): ν = 3404, 2974, 2370, 1495, 1414, 1286, 1058 cm^{-1}. MS (ESI): 245 (M+MeOH). HRMS (ESI) $C_{10}H_8F_2NS$ (M-H) for Calcd: 212.0346, Found: 212.0340.

3-[(Difluoromethy)thio]-5-methoxy-1*H*-indole (6f)

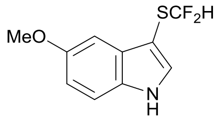

6f was prepared according to the **General procedure (A).**

White solid (33.9 mg, 74%). Mp: 95-96 ºC. Eluent: ethyl acetate/hexane =1/4, Rf = 0.3.

^{1}H NMR (300 MHz, $CDCl_3$): δ ppm 8.40 (brs, 1H), 7.44 (d, *J* = 2.7 Hz, 1H), 7.31 (d, *J* = 8.7 Hz, 1H), 7.21 (d, *J* = 2.1 Hz, 1H), 6.93 (dd, *J* = 8.7, 2.4 Hz, 1H), 6.67 (t, *J* = 57.6 Hz, 1H), 3.90 (s, 3H). ^{19}F NMR (282 MHz, $CDCl_3$): δ ppm -92.5 (d, *J* = 57.2 Hz, 2F). ^{13}C NMR (125 MHz, $CDCl_3$): δ ppm 155.36, 132.32, 130.91, 130.46, 121.08 (t, *J* = 274 Hz), 113.80, 112.44, 100.44, 96.07 (t, *J* = 3.75 Hz). 55.78. IR (KBr): ν = 3381, 3152, 3005, 2831, 1584, 1483, 1286, 1204, 1058, 1023 cm^{-1}. MS (ESI): 230 (M+H). HRMS (ESI) $C_{10}H_8F_2NOS$ (M-H) for Calcd: 228.0295, Found: 228.0307.

3-[(Difluorometh y)thio]-6-chloro-1*H*-indole (6g)

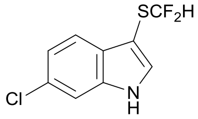

6g was prepared according to the **General procedure (A).**

Brown solid (19.6 mg, 42%: using **1d**, 14.5 mg, 31%: using **1a**). Mp: 45-46 ºC. Eluent: ethyl acetate/hexane =1/4, Rf = 0.5.

^{1}H NMR (300 MHz, $CDCl_3$): δ ppm 8.48 (brs, 1H), 7.69 (d, *J* = 8.1 Hz, 1H), 7.47-7.41 (m, 2H), 7.22 (d, *J* = 8.4 Hz, 1H), 6.67 (t, *J* = 57.3 Hz, 1H). ^{19}F NMR (282 MHz, $CDCl_3$): δ ppm -92.5 (d, *J* = 57.5 Hz, 2F). ^{13}C NMR (125 MHz, $CDCl_3$): 136.37, 132.38, 129.29, 128.39, 122.15, 120.60 (t, *J* = 275 Hz), 120.40, 111.52, 97.04 (t, *J* = 3.75 Hz). IR (KBr): ν = 3427, 3144, 2924, 1615, 1503, 1441, 1313, 1227, 1065, 1027 cm^{-1}. MS (ESI): 251 ($M+NH_4$). HRMS (ESI) $C_9H_5ClF_2NS$ (M-H) for Calcd: 231.9799, Found: 231.9797.

3-[(Difluorometh y)thio]-1-methyl-1*H*-indole (6h)

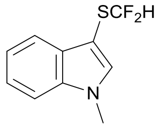

6h was prepared according to the **General procedure (A).**

White solid (35.4 mg, 83%). Mp: 45-46 ºC. Eluent: ethyl acetate/hexane =1/9, Rf = 0.5.

^{1}H NMR (300 MHz, $CDCl_3$): δ ppm 7.78 (d, *J* = 7.5 Hz, 1H), 7.38-7.31 (m, 2H), 7.29-7.25 (m, 2H), 6.65 (t, *J* = 57.6 Hz, 1H), 3.83 (s, 3H). ^{19}F NMR (282 MHz, $CDCl_3$): δ ppm -92.6 (d, *J* = 57.5 Hz, 2F). ^{13}C NMR (125 MHz, $CDCl_3$): 137.18, 136.09, 130.38, 122.72, 121.03 (t, *J* = 275 Hz), 120.88, 119.37, 109.75, 94.12 (t, *J* = 3.75 Hz), 33.18. IR (KBr): ν = 3121, 3056, 2958, 1514, 1460, 1336, 1313, 1243, 1065, 1023 cm^{-1}. MS (ESI): 252 (M+K). HRMS (EI) $C_{10}H_9F_2NS$ for Calcd: 213.0424, Found: 213.0431.

3-[(Difluorometh y)thio]-1-benzyl-1*H*-indole (6i)

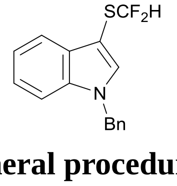

6i was prepared according to the **General procedure (A).**

Purple solid (28.9 mg, 50%). Mp: 54-55 ºC. Eluent: ethyl acetate/hexane =1/9, Rf = 0.6.

^{1}H NMR (300 MHz, $CDCl_3$): δ ppm 7.81-7.78 (m, 1H), 7.39 (s, 1H), 7.34-7.31 (m, 3H),

7.28-7.24(m, 3H), 7.14 (d, *J* = 6.9 Hz, 2H), 6.67 (t, *J* = 57.6 Hz, 1H), 5.34 (s, 2H). ^{19}F NMR (282 MHz, $CDCl_3$): δ ppm -92.7 (d, *J* = 57.2 Hz, 2F). ^{13}C NMR (125 MHz, $CDCl_3$): 136.78, 136.24, 135.46, 130.60, 128.94, 128.02, 126.97, 122.93, 121.11, 121.02 (t, *J* = 274 Hz), 119.53, 110.28, 95.13 (t, *J* = 3.75 Hz), 50.51. IR (KBr): ν = 3113, 3063, 3029, 1604, 1495, 1452, 1309, 1065, 1035 cm^{-1}. MS (ESI): 290 (M+H). HRMS (EI) $C_{16}H_{13}F_2NS$ for Calcd: 289.0737, Found: 289.0756.

2-[(Difluoromethy)thio]-5-phenyl-1*H*-pyrrole (8a)

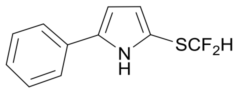

8a was prepared according to the **General procedure (A).**

Purple solid (24.6 mg, 55%: using **2d**, 20.6 mg, 46%: using **2a**). Mp: 30-31ºC. Eluent: ethyl acetate/hexane =1/9, Rf = 0.6.

^{1}H NMR (300 MHz, $CDCl_3$): δ ppm 8.54 (brs, 1H), 7.49 (d, *J* = 7.2 Hz, 2H), 7.40 (t, *J* = 7.2 Hz, 2H), 7.30-7.26 (m, 1H), 6.67 (t, *J* = 57.3 Hz, 1H), 6.61 (s, 1H), 6.55 (s, 1H). ^{19}F NMR (282 MHz, $CDCl_3$): δ ppm -92.8 (d, *J* = 57.5 Hz, 2F). ^{13}C NMR (125 MHz, $CDCl_3$): δ ppm 137.02, 131.50, 129.01, 127.35, 124.20, 121.58, 120.32 (t, *J* = 276 Hz), 109.82 (t, *J* = 3.75 Hz), 107.99. IR (KBr): ν = 3419, 3129, 2974, 1725, 1607, 1495, 1456, 1286, 1042 cm^{-1}. MS (ESI): 257 (M+MeOH). HRMS (ESI) $C_{11}H_8F_2NS$ (M-H) for Calcd: 224.0346, Found: 224.0340.

2-[(Difluoromethy)thio]-5-(*p*-tolyl)-1*H*-pyrrole (8b)

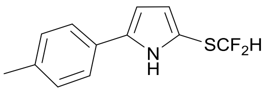

8b was prepared according to the **General procedure (A).**

Purple solid (22.1 mg, 46%: using **2d**, 13.4 mg, 28%: using **2a**). Mp: 45-46 ºC. Eluent: ethyl acetate/hexane =1/9, Rf = 0.6.

^{1}H NMR (300 MHz, $CDCl_3$): δ ppm 8.53 (brs, 1H), 7.38 (d, *J* = 7.5 Hz, 2H), 7.20 (t, *J* = 7.2 Hz, 2H), 6.66 (t, *J* = 57.9 Hz, 1H), 6.60 (s, 1H), 6.50 (s, 1H), 2.37 (s, 3H). ^{19}F NMR (282 MHz, $CDCl_3$): δ ppm -92.9 (d, *J* = 57.2 Hz, 2F). ^{13}C NMR (125 MHz, $CDCl_3$): δ ppm 137.25, 137.21, 129.68, 128.78, 124.16, 121.54, 120.40 (t, *J* = 276 Hz), 109.31 (t, *J* = 3.75 Hz), 107.52, 21.27. IR (KBr): ν = 3404, 2924, 1708, 1507, 1286, 1166, 1077, 1038 cm^{-1}. MS (ESI): 262 (M+Na). HRMS (ESI) $C_{12}H_{10}F_2NS$ (M-H) for Calcd: 238.0502, Found: 238.053.

2-[(Difluoromethy)thio]-5-(4-bromophenyl)-1*H*-pyrrole (8c)

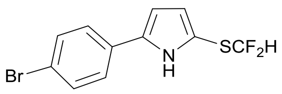

8c was prepared according to the **General procedure (A).**

Purple solid (34.0 mg, 51%: using **2d**, 25.3 mg, 38%: using **2a**). Mp: 55 ºC. Eluent: ethyl acetate/hexane =1/9, Rf = 0.5.

^{1}H NMR (300 MHz, $CDCl_3$): δ ppm 8.54 (brs, 1H), 7.51 (d, *J* = 8.4 Hz, 2H), 7.35 (d, *J* = 8.4 Hz, 2H), 6.67 (t, *J* = 57.3 Hz, 1H), 6.61 (t, *J* = 3.0 Hz, 1H), 6.53 (t, *J* = 3.0 Hz, 1H). ^{19}F NMR (282 MHz, $CDCl_3$): δ ppm -92.8 (d, *J* = 57.5 Hz, 2F). ^{13}C NMR (125 MHz, $CDCl_3$): δ ppm 135.83, 132.12, 130.42, 125.68, 121.72, 121.06, 120.12 (t, *J* = 275 Hz), 110.46 (t, *J* = 3.75 Hz), 108.43. IR (neat): ν = 3403, 2312, 1487, 1421, 1286, 1166, 1085, 1131 cm^{-1}. MS (ESI): 304 (M+H). HRMS (ESI) $C_{11}H_7BrF_2NS$ (M-H) for Calcd: 301.9451, Found: 301.9455.

3-Acetyl-5-[(difluoromethyl)thio]-2,4-dimethyl-1*H*-pyrrole (8d)

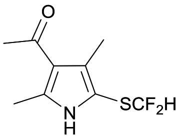

8d was prepared according to the **General procedure (A).**

White solid (28.1 mg, 64%: using **2d**, 11.4 mg, 26%: using **2a**). Mp: 134-135ºC. Eluent: ethyl acetate/hexane =1/4, Rf = 0.3.

^{1}H NMR (300 MHz, $CDCl_3$): δ ppm 8.43 (brs, 1H), 6.56 (t, *J* = 57.3 Hz, 1H), 2.53 (s, 3H), 2.45 (s, 3H), 2.38 (s, 3H). ^{19}F NMR (282 MHz, $CDCl_3$): δ ppm -92.8 (d, *J* = 57.5 Hz, 2F). ^{13}C NMR (125 MHz, $CDCl_3$): δ ppm 194.96, 138.68, 130.81, 122.71, 119.87 (t, *J* = 276 Hz), 106.08 (t, *J* = 3.75 Hz), 30.96, 15.23, 13.03. IR (KBr): ν = 3175, 3106, 3048, 2370, 2312, 1619, 1471, 1054 cm^{-1}. MS (ESI): 242 (M+Na). HRMS (ESI) $C_9H_{10}F_2NOS$ (M-H) for Calcd: 218.0451, Found: 218.0438

Ethyl 5-[(difluoromethyl)thio]-2,4-dimethyl-1*H*-pyrrole-3-carboxylate (8e)

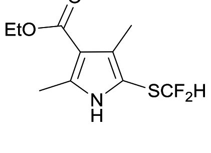

8e was prepared according to the **General procedure (A).**

White solid (45.4 mg, 91%). Mp: 114-115 ºC. Eluent: ethyl acetate/hexane =1/9, Rf = 0.4.

^{1}H NMR (300 MHz, $CDCl_3$): δ ppm 8.22 (brs, 1H), 6.55 (t, *J* = 57.3 Hz, 1H), 4.28 (q, *J*

= 7.2 Hz, 2H), 2.51 (s, 3H), 2.34 (s, 3H), 1.35 (t, *J* = 7.2 Hz, 3H). ^{19}F NMR (282 MHz, $CDCl_3$): δ ppm -92.9 (d, *J* = 56.4 Hz, 2F). MS (ESI): 281 (M+MeOH). The product was identified by comparison of the spectral data with the report data. Reference: Zhu D, Gu, Lu, Shen Q. 2015 N-Difluoromethylthiophthalimide: A Shelf-Stable, Electrophilic Reagent for Difluoromethylthiolation. *J. Am. Chem. Soc.* **137**, 10547-10553. (doi:10.1021/jacs.5b03170)

Ethyl 3-oxo-3-phenyl-2-[(difluoromethyl)thio]propanoate (10a) (10p)

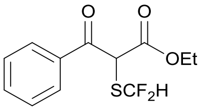

10a (10p) was prepared according to the **General procedure (B) and (D).**
Brown oil (**10a**: 19.8 mg, 36%, **10p**: 40.2 mg, 73%). Eluent: ethyl acetate/hexane =1/5, Rf = 0.3.
^{1}H NMR (300 MHz, $CDCl_3$): δ ppm 8.00 (d, *J* = 7.2 Hz, 2H), 7.64 (d, *J* = 6.9 Hz, 1H), 7.54-7.43 (m, 2H), 7.07 (t, *J* = 56.1 Hz, 0.8H) (ketone), 6.58 (t, *J* = 57.3 Hz, 0.1H) (enol), 5.51 (s, 0.7H) (ketone), 4.39 (q, *J* = 6.9 Hz, 0.5H) (enol), 4.24 (q, *J* = 7.2 Hz, 1.5H) (ketone), 1.40 (t, *J* = 7.2 Hz, 0.7 H) (enol), 1.22 (t, *J* = 7.2 Hz, 2.4H) (ketone). ^{19}F NMR (282 MHz, $CDCl_3$): δ ppm -94.26 (dd, *J* = 55.5, 245 Hz, 1F) (ketone), -92.65 (dd, *J* = 55.5, 245 Hz, 1F) (ketone), -96.32 (d, *J* = 57.2 Hz, 2F) (enol). 75% of ketone, 25% of enol form. ^{13}C NMR (125 MHz, $CDCl_3$): δ ppm 189.62 (ketone), 182.66 (enol), 173.26 (enol), 166.76 (ketone), 134.39 (ketone), 134.14 (enol or ketone), 134.12 (enol or ketone), 130.71 (enol or ketone), 129.07 (enol), 128.98 (enol or ketone), 128.97 (enol or ketone), 127.79 (ketone), 120.30 (t, *J* = 276 Hz) (enol), 119.43 (t, *J* = 274 Hz) (ketone), 87.02 (t, *J* = 3.75 Hz) (enol), 63.03 (ketone), 62.38 (enol), 51.33 (t, *J* = 2.50 Hz) (ketone), 14.10 (enol), 13.76 (ketone). IR (neat): ν = 2985, 1742, 1685, 1561, 1448, 1263, 1193, 1139, 1073, 1031 cm^{-1}. MS (ESI): 297 (M+Na) HRMS (ESI) $C_{12}H_{12}F_2NaO_3S$ (M+Na) for Calcd: 297.0373, Found: 297.0376.

Ethyl 3-oxo-4-tolyl-2-[(difluoromethyl)thio]propanoate (10q)

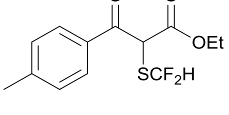

10q was prepared according to the **General procedure (D).**

Yellow oil (36.3 mg, 63%). Eluent: ethyl acetate/hexane =1/7, Rf = 0.5.

^{1}H NMR (300 MHz, $CDCl_3$): δ ppm 7.90 (d, *J* = 8.1 Hz, 1.7H) (ketone), 7.56 (d, *J* = 8.1 Hz, 0.3H) (enol), 7.32-7.24 (m, 2H), 7.06 (t, *J* = 56.7 Hz, 0.7H) (ketone), 6.59 (t, *J* = 57.6 Hz, 0.1H) (enol), 5.48 (s, 0.7H) (ketone), 4.38 (q, *J* = 6.9 Hz, 0.3H) (enol), 4.25 (q, *J* = 6.9 Hz, 1.7H) (ketone), 2.44 (s, 2.6H) (ketone), 2.41 (s, 0.5H) (enol), 1.40 (t, *J* = 7.2 Hz, 0.5 H) (enol), 1.22 (t, *J* = 6.9 Hz, 2.5H) (ketone). ^{19}F NMR (282 MHz, $CDCl_3$): δ ppm -92.62 (dd, *J* = 55.5, 244 Hz, 1F) (ketone), -94.30 (dd, *J* = 55.5, 244 Hz, 1F) (ketone), -96.19 (d, *J* = 57.5 Hz, 2F) (enol). 83% of ketone, 17% of enol form. ^{13}C NMR (125 MHz, $CDCl_3$): δ ppm 189.15 (ketone), 182.69 (enol), 173.34 (enol), 166.89 (ketone), 145.61 (ketone), 141.24 (enol), 131.61 (ketone), 131.26 (enol), 129.65 (ketone), 129.17 (enol), 129.11 (ketone), 128.47 (enol), 120.46 (t, *J* = 276 Hz) (enol), 119.49 (t, *J* = 273 Hz) (ketone), 86.53 (t, *J* = 3.75 Hz) (enol), 62.95 (ketone), 62.29 (enol), 51.31 (t, *J* = 2.50 Hz) (ketone), 21.76 (ketone), 21.50 (enol), 14.10 (enol), 13.77 (ketone). IR (neat): ν = 2958, 1742, 1685, 1607, 1267, 1185, 1073, 1031 cm^{-1}. MS (ESI): 287 (M-H). HRMS (ESI) $C_{13}H_{14}F_2NaO_3S$ (M+Na) for Calcd: 311.0529, Found: 311.0530.

Ethyl 3-oxo-(4-chlorophenyl)-2-[(difluoromethyl)thio]propanoate (10r)

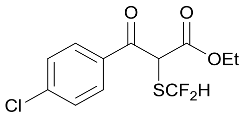

10r was prepared according to the **General procedure (D).**

Brown oil (40.8 mg, 66%). Eluent: ethyl acetate/hexane =1/7, Rf =0.6.

^{1}H NMR (300 MHz, $CDCl_3$): δ ppm 7.95 (d, *J* = 8.4 Hz, 1.1H) (ketone), 7.61 (d, *J* = 8.4 Hz, 0.9H) (enol), 7.49 (d, *J* = 8.4 Hz, 1.2H) (ketone), 7.41 (d, *J* = 8.4 Hz, 0.8H) (enol), 7.05 (t, *J* = 55.8 Hz, 0.5H) (ketone), 6.59 (t, *J* = 57.6 Hz, 0.2H) (enol), 5.45 (s, 0.5H) (ketone), 4.39 (q, *J* = 7.2 Hz, 0.8H) (enol), 4.26 (q, *J* = 7.1 Hz, 1.2H) (ketone), 1.40 (t, *J* = 6.9 Hz, 1.1 H) (enol), 1.23 (t, *J* = 6.9 Hz, 1.9H) (ketone). ^{19}F NMR (282 MHz, $CDCl_3$): δ ppm -92.71 (dd, (dd, *J* = 55.5, 244 Hz, 1F) (ketone), -94.00 (dd, *J* = 55.5, 244 Hz, 1F) (ketone), -96.21 (d, *J* = 57.2 Hz, 2F) (enol). 54% of ketone, 46% of enol form. ^{13}C NMR (125 MHz, $CDCl_3$): δ ppm 189.59 (ketone), 181.26 (enol), 173.13 (enol), 166.49 (ketone), 141.06 (enol or ketone), 136.90 (enol or ketone), 132.47 (enol or ketone), 132.41 (enol or ketone), 130.64 (enol or ketone), 130.37 (enol or ketone), 129.33 (enol or ketone), 128.13 (enol or ketone), 120.08 (t, *J* = 276 Hz) (enol), 119.31 (t, *J* = 274 Hz) (ketone), 87.23 (t, *J* = 3.75 Hz) (enol), 63.18 (ketone), 62.52 (enol), 51.21 (t,

J = 2.50 Hz) (ketone), 14.08 (enol), 13.78 (ketone). IR (neat): ν = 2985, 1739, 1688, 1592, 1487, 1263, 1092, 1031 cm^{-1}. MS (ESI): 307 (M-H). HRMS (ESI) $C_{12}H_{11}ClF_2NaO_3S$ (M+Na) for Calcd: 330.9983, Found: 330.9986.

Methyl 3-oxo-(4-bromophenyl)-2-((difluoromethyl)thio)propanoate (10s)

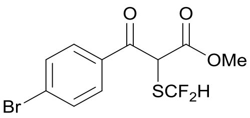

10s was prepared according to the **General procedure (D).**

Colorless oil (45.8 mg, 68%). Eluent: ethyl acetate/hexane = =1/9, Rf = 0.4.

^{1}H NMR (300 MHz, $CDCl_3$): δ ppm 7.87 (d, *J* = 8.4 Hz, 1H) (ketone), 7.66 (d, *J* = 8.4 Hz, 1H) (enol), 7.55 (brs, 2H), 7.04 (t, *J* = 55.8 Hz, 0.5H) (ketone), 6.59 (t, *J* = 57.6 Hz, 0.4H) (enol), 5.48 (s, 0.5H) (ketone), 3.93 (s, 1.4H) (enol), 3.79 (s, 1.6H) (ketone). ^{19}F NMR (282 MHz, $CDCl_3$): δ ppm -92.87 (dd, (dd, *J* = 55.3, 243 Hz, 1F) (ketone), -94.00 (dd, *J* = 55.3, 243 Hz, 1F) (ketone), -96.34 (d, *J* = 57.5 Hz, 2F) (enol). 51% of ketone, 49% of enol form. ^{13}C NMR (125 MHz, $CDCl_3$): δ ppm 188.73 (ketone), 181.49 (enol), 173.52 (enol), 166.97 (ketone), 132.80 (enol), 132.71 (enol), 132.38 (ketone), 132.14 (enol), 131.12 (ketone), 130.80 (ketone), 130.42 (ketone), 129.98 (enol), 119.93 (t, *J* = 276 Hz) (enol), 119.22 (t, *J* = 273 Hz) (ketone), 86.99 (t, *J* = 3.75 Hz) (enol), 53.89 (ketone), 53.25 (enol), 50.78 (t, *J* = 2.50 Hz) (ketone). IR (neat): ν = 2958, 1746, 1685 1584, 1483, 1437, 1263, 1073 cm^{-1}. MS (ESI): 337 (M-H). HRMS (ESI) $C_{11}H_9BrF_2NaO_3S$ (M+Na) for Calcd: 360.9322 Found: 360.9319

1-Phenyl-2-[(difluoromethyl)thio]-1,3-butanedion (10t)

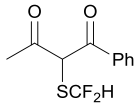

10t was prepared according to the **General procedure (D).**

Orange oil (34.9 mg, 71%). Eluent: ethyl acetate/hexane =1/5, Rf = 0.6.

^{1}H NMR (300 MHz, $CDCl_3$): δ ppm 7.63 (d, *J* = 7.5 Hz, 2H), 7.49-7.43 (m, 3H), 6.37 (t, *J* = 56.4 Hz, 1H), 2.55 (s, 3H). ^{19}F NMR (282 MHz, $CDCl_3$): δ ppm -95.26 (d, *J* = 56.4 Hz, 2F). 100% of enol form. ^{13}C NMR (125 MHz, $CDCl_3$): δ ppm 201.72, 194.01, 135.94, 131.03, 128.71, 127.79, 119.94 (t, *J* = 276 Hz), 94.99 (t, *J* = 3.75 Hz), 25.60. IR (KBr): ν = 3063, 2966, 1542, 1398, 1073, 1035 cm^{-1}. MS (ESI): 267 (M+Na). HRMS (ESI) $C_{11}H_{10}F_2NaO_2S$ (M+Na) for Calcd: 267.0267, Found: 267.0276

1-(*p*-Bromophenyl)-2-[(difluoromethyl)thio]-1,3-butanedion (10u)

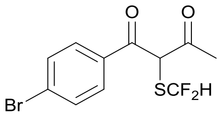

10u was prepared according to the **General procedure (D).**

Yellow oil (32.5 mg, 50%). Eluent: ethyl acetate/hexane =1/9, Rf = 0.4.

^{1}H NMR (300 MHz, $CDCl_3$): δ ppm 7.59-7.51 (m, 4H), 6.39 (t, *J* = 55.8 Hz, 1H), 2.55 (s, 3H). ^{19}F NMR (282 MHz, $CDCl_3$): δ ppm -95.26 (d, *J* = 56.4 Hz, 2F). 100% of enol form. ^{13}C NMR (125 MHz, $CDCl_3$): δ ppm 201.59, 193.06, 135.94, 131.09, 130.42, 125.72, 119.65 (t, *J* = 276 Hz), 94.75 (t, *J* = 2.5 Hz), 25.49. IR (neat): ν = 2962, 1592, 1398, 1069, 1035, 1011 cm^{-1}. MS (ESI): 321 (M-H). HRMS (ESI) $C_{11}H_9BrF_2NaO_2S$ (M+Na) for Calcd: 344.9372, Found: 344.9377

1,3-Diphenyl-2-[(difluoromethyl)thio]-1,3-propanedion (10v)

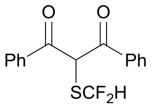

10v was prepared according to the **General procedure (D).**

Colorless oil (42.9 mg, 70%). Eluent: ethyl acetate/hexane =1/4, Rf = 0.6.

^{1}H NMR (300 MHz, $CDCl_3$): δ ppm 8.00 (d, *J* = 6.9 Hz, 3H), 7.76-7.44 (m, 7H), 7.03 (t, *J* = 55.5 Hz, 0.9H) (ketone), 6.28 (s, 1H), (ketone), 6.14 (t, *J* = 56.7 Hz, 0.2H) (enol), 3.71 (s, 0.2 H) (enol). ^{19}F NMR (282 MHz, $CDCl_3$): δ ppm -92.88 (d, *J* = 56.4 Hz, 2F) (ketone), -96.32 (d, *J* = 56.4 Hz, 2F) (enol). 84% of ketone, 16% of enol form. ^{13}C NMR (125 MHz, $CDCl_3$): δ ppm 196.20 (enol), 190.97 (ketone), 136.25 (enol), 134.32 (ketone), 131.40 (enol), 129.19 (enol), 129.15 (ketone), 129.01 (ketone), 127.86 (ketone), 119.52 (t, *J* = 273 Hz) (ketone), 93.65 (t, *J* = 3.75 Hz) (enol), 55.46 (ketone). IR (neat): ν = 3063, 2927, 2324, 1700, 1673, 1596, 1448, 1282, 1185, 1073 cm^{-1}. MS (ESI): 329 (M+Na). HRMS (ESI) $C_{16}H_{12}F_2NaO_2S$ (M+Na) for Calcd: 329.0424, Found: 329.0424.

Methyl 2-[(difluoromethyl)thio]-6-methyl-1-oxo-2,3-dihydro-1*H*-indane-2-calboxyl ate (10b) (10w)

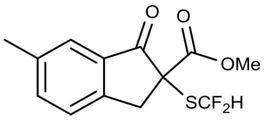

10b (10w) was prepared according to the **General procedure (B), (D) and (E).**

Yello solid (**10b**: 23.5 mg, 41%, 58% (Synthesized by general procedure E and yield was calculated by ^{19}F NMR with PhF as an internal standard.), **10w**: 36.6 mg, 64%). Eluent: DCM/hexane = 4/1, Rf = 0.7

^{1}H NMR (300 MHz, $CDCl_3$): δ ppm 7.64 (s, 1H), 7.51 (t, *J* = 55.5 Hz, 1H), 7.50 (brs, 1H), 7.36 (d, *J* = 7.5 Hz, 1H), 3.98 (d, *J* = 17.7 Hz, 1H), 3.81 (s, 3H), 3.22 (d, 18.0 Hz, 1H), 2.43 (s, 3H) ^{19}F NMR (282 MHz, $CDCl_3$): δ ppm -92.06 (dd, *J* = 55.3, 250 Hz, 1F), -93.48 (dd, *J* = 55.5, 250 Hz, 1F). MS (ESI):309 (M+Na). HRMS (ESI) $C_{13}H_{13}F_2NaO_3S$ (M+Na) for Calcd: 309.0373, Found: 309.0370. The product was identified by comparison of the spectral data with the report data. Reference: Zhu D, Gu, Lu, Shen Q. 2015 N-Difluoromethylthiophthalimide: A Shelf-Stable, Electrophilic Reagent for Difluoromethylthiolation. *J. Am. Chem. Soc.* **137**, 10547-10553. (doi:10.1021/jacs.5b03170)

Allyl 2-[(difluoromethyl)thio]-6-methyl-1-oxo-2,3-dihydro-1*H*-indane-2-calboxylate (**10c**)

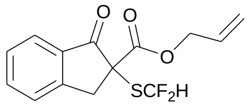

10c was prepared according to the **General procedure (E).**

Yellow oil (44.7 mg, 75%). Eluent: DCM/hexane = 4/1, Rf = 0.7.

^{1}H NMR (300 MHz, $CDCl_3$): δ ppm 7.85 (d, *J* = 7.5 Hz, 1H), 7.519 (t, *J* = 55.7 Hz, 1H) 7.716 ~ 7.666 (m, 1H), 7.469 (t, *J* = 6.8 Hz, 2H), 5.948 ~ 5.817 (m, 1H), 5.345 ~ 5.240 (m, 2H), 4.703 (d, *J* = 5.4 Hz, 2H), 4.046 (d, 17.7 Hz, 1H), 3.27 (d, *J* = 18 Hz, 1H). ^{19}F NMR (282 MHz, $CDCl_3$): δ ppm 91.9 (dd, *J* = 55.6 Hz, 250 Hz, 1F), 93.3 Hz (dd, *J* = 58.8 Hz, 252 Hz). ^{13}C NMR (125 MHz, $CDCl_3$): δ ppm 196.66, 167.98, 150.49, 136.29, 132.97, 130.71, 128.61, 126.25, 125.84, 120.26 (t, *J* = 269 Hz, 1C), 119.48, 67.55, 58.46, 39.44. IR (neat): ν = 3083, 2951, 1746, 1719, 1607, 1464, 1433, 1275, 1239, 1185, 1065, 1042 cm^{-1}. MS (ESI): 321 (M+Na).

1-phenyl-2-[(difluoromethyl)thio]-Ethanone (10d)

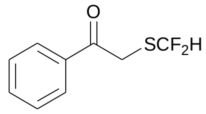

10d was prepared according to the **General procedure (F).**

Pale yellow solid (13.7 mg, 35%). Mp: 33 ~ 34 ºC. Eluent:l DCM/hexane =3/1, Rf = 0.6.

^{1}H NMR (300 MHz, $CDCl_3$): δ ppm 7.99 ~ 7.96 (m, 2H), 7.66 ~ 7.60 (m, 1H), 7.53 ~ 7.48 (m, 2H), 6.95 (t, *J* = 56.4 Hz, 1H), 4.330 (s, 2H). ^{19}F NMR (282 MHz, $CDCl_3$): δ ppm -94.52 (d, *J* = 56.4 Hz, 2F). ^{13}C NMR (125 MHz, $CDCl_3$): δ ppm193.19, 128.48, 128.88, 119.6 (t, *J* = 273 Hz), 134.97, 133.99, 34.42 (t, *J* = 3.1 Hz). IR (KBr): ν = 3334, 2947, 2920, 1673, 1596, 1580, 1448, 1390, 1329, 1313, 1204, 1085, 1023, 1004 cm^{-1}. MS (ESI): 225 (M+Na). HRMS (ESI) $C_9H_8F_2OS$ (M+Na) for Calcd: 225.0162, Found: 225.0157.

Methyl 2-[(difluoromethyl)thio]-6-methoxy-1-oxo-2,3-dihydro-1*H*-indene-2-carboxylate (10x)

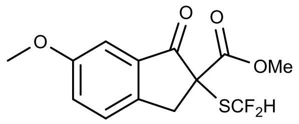

10x was prepared according to the **General procedure (D).**
White solid (41.3 mg, 68%). Mp: 65 ~ 66 ºC Eluent: DCM/hexane = 1/4, Rf = 0.3.
^{1}H NMR (300 MHz, $CDCl_3$): δ ppm 7.50 (t, *J* = 55.7 Hz, 1H), 7.36 (d, *J* = 8.4 Hz, 1H), 7.29 (d, *J* = 10.5 Hz, 2H), 3.95 (d, *J* = 17.7 Hz, 1H), 3.86 (s, 3H), 3.82 (s, 3H), 3.20 (d, *J* = 17.7 Hz, 1H). ^{19}F NMR (282 MHz, $CDCl_3$): δ ppm -92.07 (dd, *J* = 55.3, 250 Hz, 1F), -93.49 (dd, *J* = 56.4, 250, 1F). ^{13}C NMR (125 MHz, $CDCl_3$): δ ppm 196.73, 168.82, 160.21, 143.43, 134.23, 126.95, 125.92, 106.55, 120.22 (t, *J* = 269 Hz), 59.18, 55.70, 53.98, 38.93. IR (KBr): ν = 3071, 3024, 2962, 2842, 1746, 1707, 1494, 1301, 1259, 1069, 1038 cm^{-1}. MS (ESI): 325 (M+Na). HRMS (ESI) $C_{13}H_{13}F_2NaO_4S$ (M+Na) for Calcd: 325.0322, Found: 325.0320.

Methyl 2-[(difluoromethyl)thio]-1-oxo-1,2,3,4-tetrahydronaphthalene-2-carboxylate (10y)

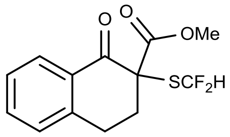

10y was prepared according to the **General procedure (D).**
Pale yellow solid (32.1 mg, 56%). Mp: 50 ºC Eluent: ethyl acetate/hexane = 1/4, Rf = 0.4.
^{1}H NMR (300 MHz, $CDCl_3$): δ ppm 8.08 (d, *J* = 7.5 Hz, 1H), 7.56 (d, *J* = 7.8 Hz, 1H), 7.36 (t, *J* = 49.2 Hz, 1H), 7.38 (t, *J* = 2.6 Hz, 1H), 7.26 – 7.25 (m, 1H), 3.81 (s, 3H), 3.13 – 3.01 (m, 2H), 2.98 – 2.91 (m, 1H), 2.43 – 2.34 (m, 1H). ^{19}F NMR (282 MHz,

CDCl$_3$): δ ppm -91.88 (dd, *J* = 57.8, 254 Hz, 1F), -94.48 (dd, *J* = 55.5, 254 Hz, 1F). ^{13}C NMR (125 MHz, CDCl$_3$): δ ppm 190.15, 169.17, 142.24, 134.48, 130.19, 128.73 (d, *J* = 2.5 Hz), 122.89, 120.75 (t, *J* = 239 Hz,), 120.73, 61.27 (d, *J* = 1.3 Hz), 53.76, 32.47, 25.82 IR (KBr): ν = 3361, 3071, 2969, 1726, 1688, 1599, 1436, 1297, 1251, 1069, 1034 cm^{-1}. MS (ESI): 309 (M+Na). HRMS (ESI) $C_{13}H_{13}F_2NaO_3S$ (M+Na) for Calcd: 309.0373, Found: 309.0381.

(*E*)-{3-[(Difluoromethyl)-sulfinyl]prop-1-enyl}benzene (12a)

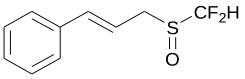

12a was prepared according to the **General procedure (C).**

White solid (20.0 mg, 46%). Mp: 58ºC. Eluent: ethyl acetate/hexane =1/4, Rf = 0.4.

^{1}H NMR (300 MHz, CDCl$_3$): δ ppm 7.43-7.32 (m, 5H), 6.80 (d, *J* =15.9 Hz, 1H), 6.20 (t, *J* = 54.0 Hz, 1H), 6.20-6.14 (m, 1H), 4.07 (d, *J* = 7.8 Hz, 2H). ^{19}F NMR (282 MHz, CDCl$_3$): δ ppm -123.28 (d, *J* = 52.5 Hz, 2F). ^{13}C NMR (125 MHz, CDCl$_3$): δ ppm 141.03, 135.13, 129.06, 128.78, 126.87, 114.83 (t, *J* = 285 Hz), 111.04, 52.94. IR (KBr): ν = 3082, 2989, 1755, 1491, 1452, 1410, 1340, 1154, 1096 cm^{-1}. MS (EI): 117 (M-S(O)CF$_2$H). HRMS (EI) C_9H_9 (M-S(O)CF$_2$H) for Calcd: 117.0704, Found:117.0682.

1-Chloro-4-{(*E*)-{3-[(difluoromethyl)-sulfinyl]prop-1-enyl}benzene (12b)

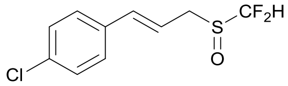

12b was prepared according to the **General procedure (C).**

White solid (25.1 mg, 50%). Mp: 61-62 ºC. Eluent: ethyl acetate/hexane =1/4, Rf = 0.5.

^{1}H NMR (300 MHz, CDCl$_3$): δ ppm 7.39-7.29 (m, 4H), 6.75 (d, *J* =15.9 Hz, 1H), 6.20 (t, *J* = 52.8 Hz, 1H), 6.17-6.09 (m, 1H), 4.06 (d, *J* = 6.6 Hz, 2H). ^{19}F NMR (282 MHz, CDCl$_3$): δ ppm -122.91 (d, *J* = 52.4 Hz, 2F). ^{13}C NMR (125 MHz, CDCl$_3$): δ ppm 139.73, 134.84, 133.61, 128.99, 128.06, 114.96 (t, *J* = 285 Hz), 111.71, 52.71. IR (KBr): ν = 2993, 2935, 2370, 1491, 1340, 1306, 1119, 1092 cm^{-1}. MS (EI): 151 (M-S(O)CF$_2$H). HRMS (EI) C_9H_9 (M-S(O)CF$_2$H) for Calcd: 151.0315, Found:151.0300.

4-{(*E*)-{3-[(difluoromethyl)-sulfinyl]prop-1-enyl}-1-methoxy-benzene (12c)

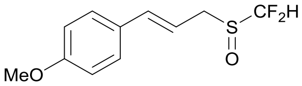

12c was prepared according to the **General procedure (C).**

Yellow solid (20.1 mg, 41%). Mp: 57-58ºC. Eluent: ethyl acetate/hexane =1/9, Rf = 0.2. ^{1}H NMR (300 MHz, $CDCl_3$): δ ppm 7.36 (d, *J* = 8.4 Hz, 2H), 6.88 (d, *J* =8.4 Hz, 2H), 6.21 (t, *J* = 52.5 Hz, 1H), 6.06-5.98 (m, 1H), 4.04 (d, *J* = 6.9 Hz, 2H), 3.82 (s, 3H). ^{19}F NMR (282 MHz, $CDCl_3$): δ ppm -123.56 (d, *J* = 52.4 Hz, 2F). ^{13}C NMR (125 MHz, $CDCl_3$): δ ppm 160.27, 140.53, 128.24, 127.94, 114.74 (t, *J* = 284 Hz), 114.14, 108.34, 55.32, 53.09. IR (KBr): ν = 2997, 2966, 2916, 2843, 1607, 1515, 1255, 1170, 1100 cm^{-1}. MS (EI): 147 (M-S(O)CF_2H). HRMS (EI) C_9H_9 (M-S(O)CF_2H) for Calcd: 147.0810, Found: 147.0784.

4-(Difluoromethyl)thio-3-phenyl-1*H*-pyrazole (13)

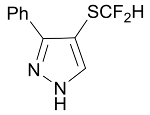

To a mixture of enamine **3z** (35.2 mg, 0.2 mmol, 1.0 equiv) and Cu(I)Br (5.7 mg, 0.04 mmol, 20 mol%) in 1,4-dioxane (2.5 ml), reagent **2a** (174.5 mg, 0.4 mmol, 2.0 equiv) was added at room temperature. After stirring at room temperature for 5 h, hydrazine monohydrate (48.6 μL, 1.0 mmol, 5.0 equiv) was added to the reaction mixture. The resulting mixture was continuously stirred at 90 °C for 5 h, then 2 mL of water was added and extracted with ethyl acetate, the organic phase was washed with water 2 times and brine once then dried over magnesium sulfate. The solvent was removed by rotary evaporation and purified through flash column chromatography on silica gel (eluent: ethyl acetate/hexane =1/4, Rf = 0.3-0.4) to afforded the target product **13**.

Yellow solid (30.8 mg, 68%). Mp: 61-62ºC. ^{1}H NMR (300 MHz, $CDCl_3$): δ ppm 7.77-7.74 (m, 2H), 7.70 (s, 1H), 7.45-7.43 (m, 3H), 6.60 (t, *J* = 57.3 Hz, 1H). ^{19}F NMR (282 MHz, $CDCl_3$): δ ppm -93.5 (d, *J* = 57.2 Hz, 2F). ^{13}C NMR (125 MHz, $CDCl_3$): 149.79, 142.63, 129.30, 129.21, 128.69, 128.13, 119.92 (t, *J* = 275 Hz), 98.27. IR (KBr): ν = 3168, 3101, 3063, 3016, 2951, 2873, 1317, 1069, 1031 cm^{-1}. MS (EI): 249 (M+Na). HRMS (ESI) $C_{10}H_7F_2N_2S$ (M-H) for Calcd: 225.0298, Found: 225.0297.

5-(Difluoromethyl)thio-4-phenyl-2-(*tert*-butyl)-pyrimidine (14)

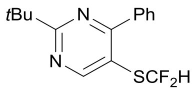

To a mixture of enamine **3z** (35.2 mg, 0.2 mmol, 1.0 equiv) and Cu(I)Br (5.7 mg, 0.04 mmol, 20 mol%) in 1,4-dioxane (2.5 ml), reagent **2a** (174.5 mg, 0.4 mmol, 2.0 equiv) was added at room temperature. After stirring at room temperature for 5 h, *tert*-butylcarbamidine hydrochloride (136.6 mg, 1.0 mmol, 5.0 equiv), sodium

methoxide (67.0 mg, 1.24 mmol, 6.2 equiv) and EtOH (2.0 ml) were added to the reaction mixture. The resulting mixture was continuously stirred at 80 °C for 20 h, then 2 mL of water was added and extracted with ethyl acetate, the organic phase was washed with water 2 times and brine once then dried over magnesium sulfate. The solvent was removed by rotary evaporation and purified through flash column chromatography on silica gel (eluent: ethyl acetate/hexane =1/9, Rf = 0.6) to afforded the target product **14**.

Colorless oil (38.3 mg, 65%). ^{1}H NMR (300 MHz, $CDCl_3$): δ ppm 8.90 (s, 1H), 7.77 (brs, 2H), 7.50 (brs, 3H), 6.60 (t, *J* = 56.1 Hz, 1H), 1.46 (s, 9H) ^{19}F NMR (282 MHz, $CDCl_3$): δ ppm -91.8 (d, *J* = 56.4 Hz, 2F). ^{13}C NMR (125 MHz, $CDCl_3$): 178.06, 168.11, 163.30, 137.26, 130.00, 129.90, 128.10, 119.45 (t, *J* = 276 Hz), 116.15 (t, *J* = 2.5 Hz), 39.59, 29.50. IR (neat): ν = 3063, 3029, 2958, 1557, 1519, 1421, 1321, 1185, 1061, 1035 cm^{-1}. MS (EI): 294. HRMS (EI) $C_{15}H_{16}F_2N_2S$ for Calcd: 294.1002, Found: 294.0997.

Methyl 6-methoxy-1-amino-1*H*-indene-2-carboxylate (3x)

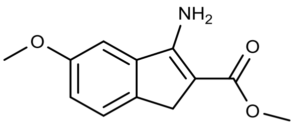

3x was prepared according to the reported procedure.

White solid (491.7 mg, 99%). Mp = 127 ~ 128 °C. Eluent: ethyl acetate/hexane = 1/2, Rf = 0.4. ^{1}H NMR (300 MHz, $CDCl_3$): δ ppm 7.36 (d, *J* = 7.5 Hz, 1H), 6.96 (d, *J* = 7.8 Hz, 1H), 6.91 (s, 1H), 5.93 (brs, 2H), 3.85 (s, 3H), 3.80 (s, 3H), 3.49 (s, 2H) ^{13}C NMR (125 MHz, $CDCl_3$): δ ppm 168.12, 158.76, 156.06, 139.16, 136.30, 125.30, 115.26, 103.81, 99.08, 55.49, 50.50, 33.73. IR (KBr): ν = 3431, 3327, 3229, 3160, 3001, 2951, 2889, 2835, 1646, 1631, 1542, 1444, 1336, 1313, 1248, 1204, 1100, 1027 .MS (ESI): 220 (M+H). HRMS (ESI) $C_{12}H_{13}NNaO_3$ (M+Na) for Calcd: 242.0793, Found: 242.0775

6. X-ray structure of 4c

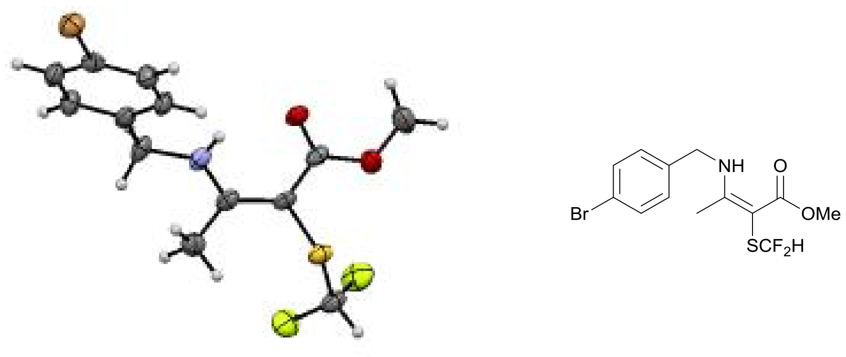

Figure S1. X-ray structure of **4c**

7. References and Notes

1. He Z, Tan P, Ni C, Hu *J*. 2015 Fluoroalkylative Aryl Migration of Con*J*ugated N-Arylsulfonylated Amides Using Easily Accessible Sodium Di- and Monofluoroalkanesulfinates, *Org*. *Lett*. **17**, 1838-1841. (doi:10.1021/acs.orglett.5b00308)
2. Zhang ZH, Yin L, Wang YM. 2006 A General and Efficient Method for the Preparation of β-Enamino Ketones and Esters Catalyzed by Indium Tribromide, *Adv*. *Synth*. *Catal*. **348**, 184-190. (doi:10.1002/adsc.200505268)
3. Zhao M, Wang F, Li X. 2012 Cross-Dehydrogenative Coupling between Enamino Esters and Ketones: Synthesis of Tetrasubstituted Pyrroles, *Org*. *Lett*. **14**, 1412-1415.(doi: 10.1021/ol300147t)
4. Yang YD, Azuma A, Tokunaga E, Yamasaki M, Shiro M, Shibata N. 2013 Trifluoromethanesulfonyl Hypervalent Iodonium Ylide for Copper-Catalyzed Trifluoromethylthiolation of Enamines, Indoles, and β-Keto Esters, *J*. *Am*. *Chem*. *Soc*., **135**, 8782-8785. **(**doi:10.1021/*J*a402455f)
5. Roman H, Johannes EB, Frank G. 2015 Transition-Metal-Free Trifluoromethylthiolation of N-Heteroarenes *Chem*. *-Eur*. *J*. **21**. 8047-8051. (doi: 10.1002/chem.201500957)
6. Stephan L, Winfriend M, *J*an W B. Udo N. 2002 Auxiliary controlled enantioselective synthesis of 3-aryl-prolines, *Tetrahedron* **58**, 1317-1334. (doi:10.1016/S0040-4020(01)01233-9)

8. ^{19}F, ^{1}H, ^{13}C NMR Spectra of Corresponding Compounds

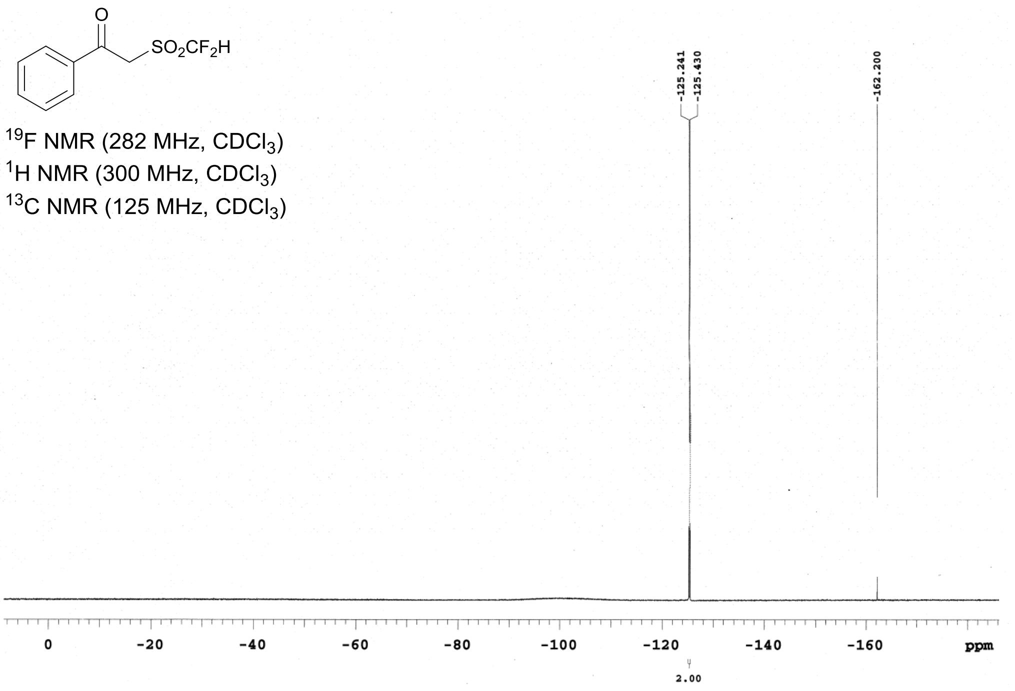

^{19}F NMR (282 MHz, $CDCl_3$)
^{1}H NMR (300 MHz, $CDCl_3$)
^{13}C NMR (125 MHz, $CDCl_3$)

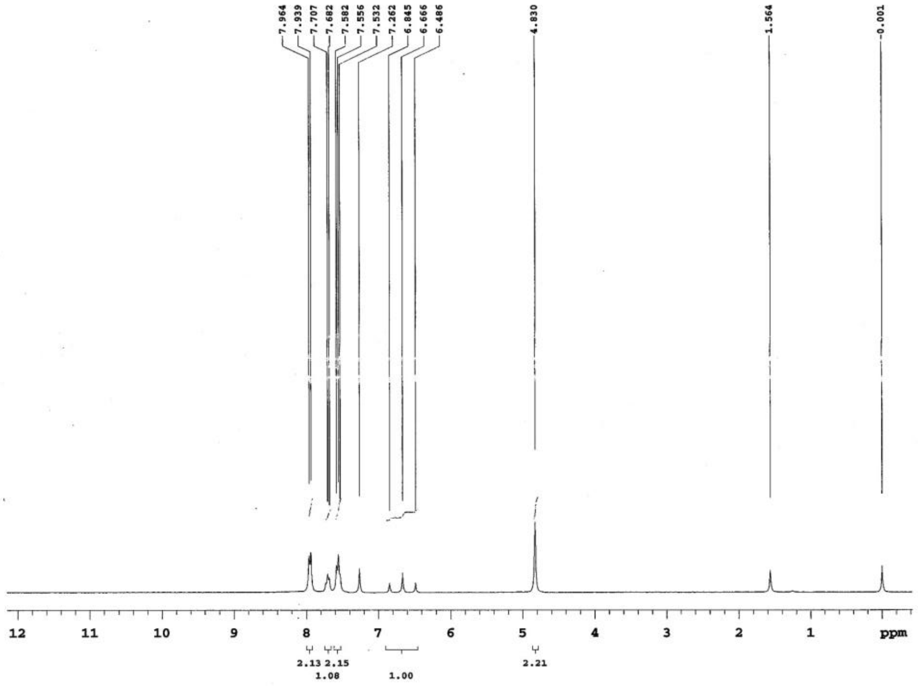
7.964
7.939
7.707
7.682
7.582
7.556
7.532
7.262
6.845
6.666
6.486
4.830
1.564
0.001
2.13
2.15
1.08
1.00
2.21
ppm

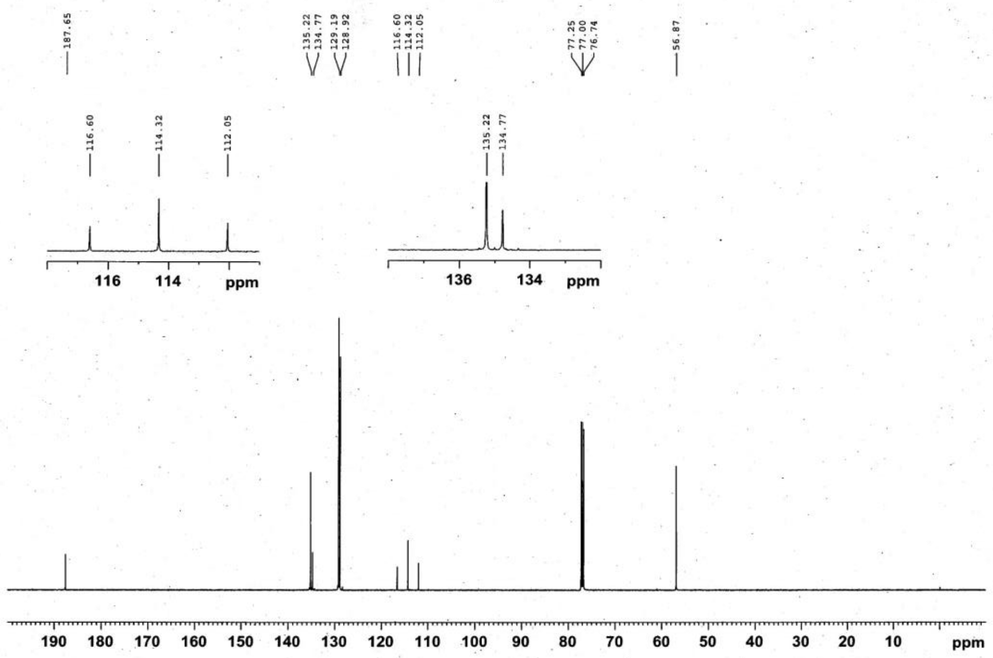
187.65
135.22
134.77
129.19
128.92
116.60
114.32
112.05
77.25
77.00
76.74
56.87
ppm

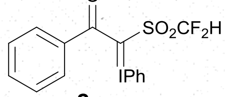

2a

^{19}F NMR (282 MHz, $CDCl_3$)
^{1}H NMR (300 MHz, $(CD_3)_2SO$)
^{13}C NMR (125 MHz, $CDCl_3$)

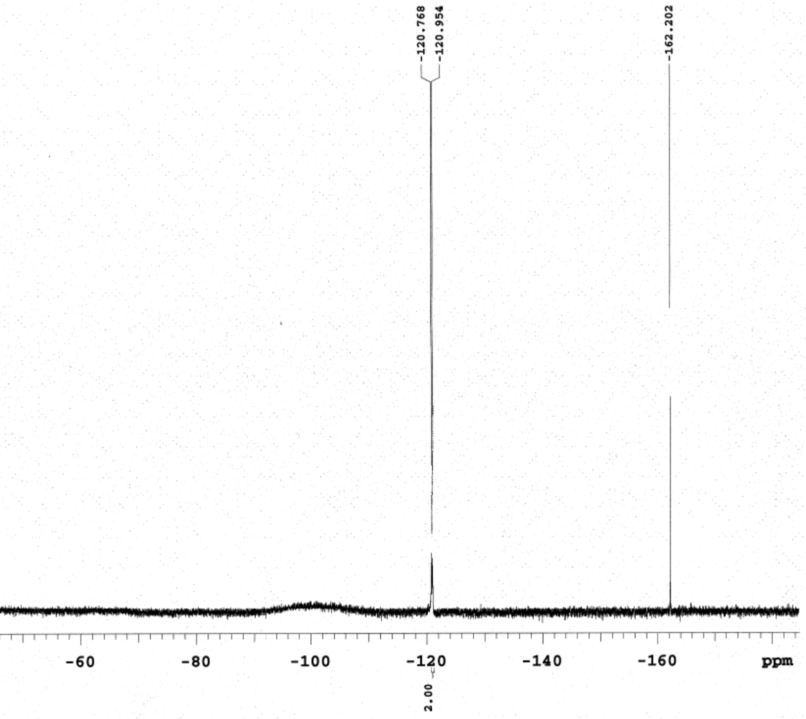

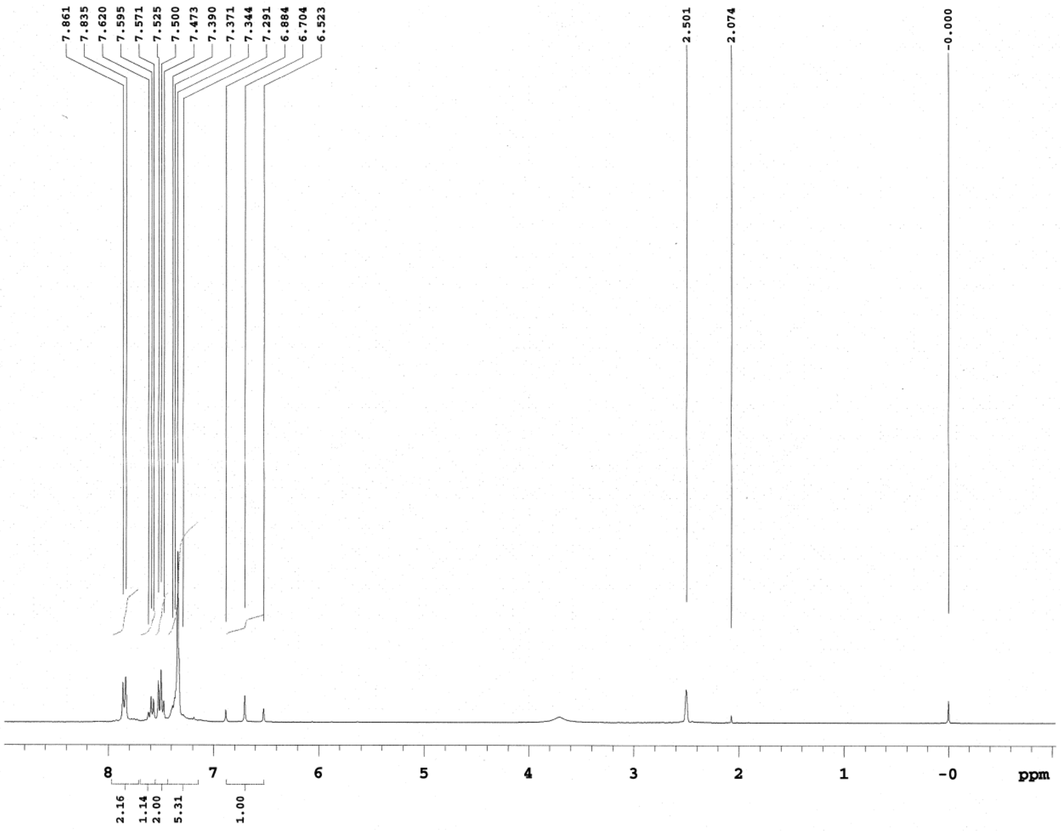

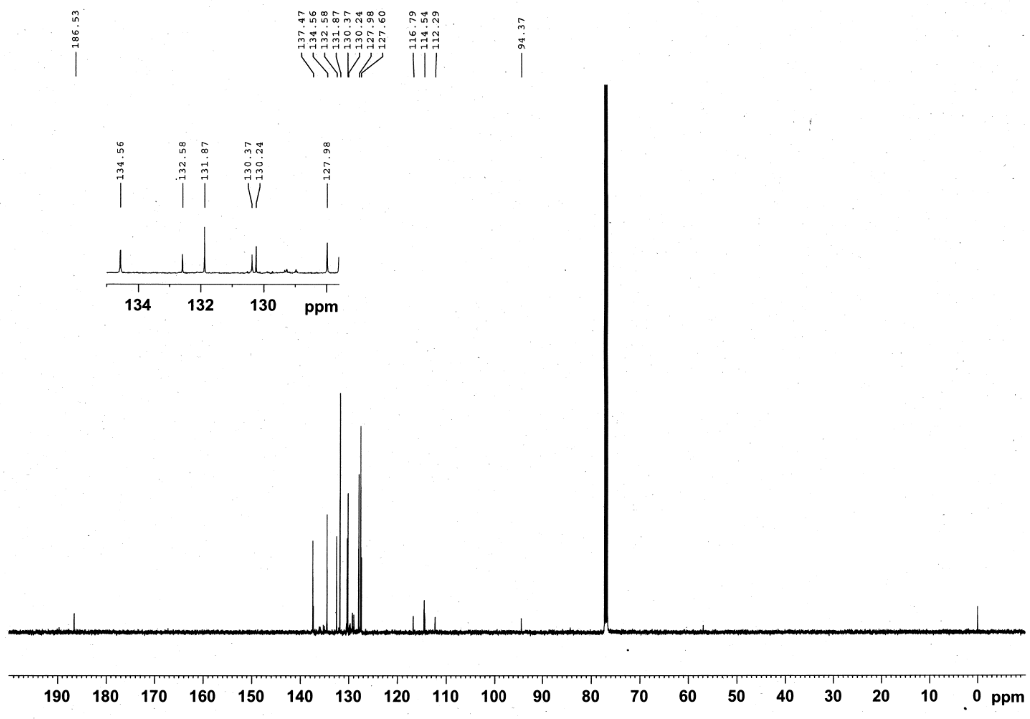

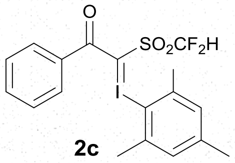

2c

^{19}F NMR (282 MHz, $CDCl_3$)

^{1}H NMR (300 MHz, $CDCl_3$)

^{13}C NMR (125 MHz, CD_3CN)

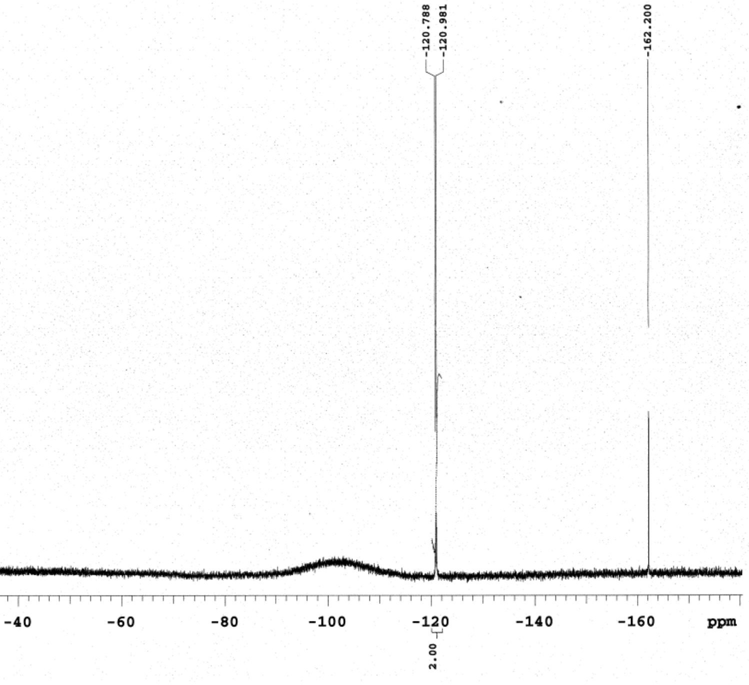

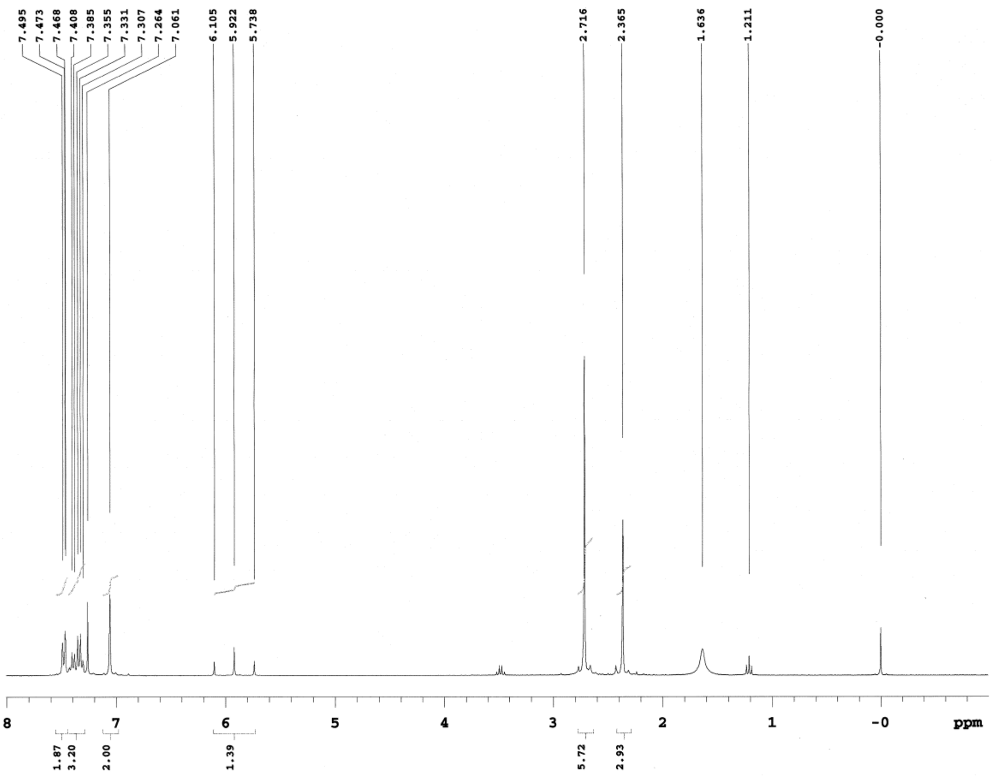
7.495
7.473
7.468
7.408
7.385
7.355
7.331
7.307
7.264
7.061
6.105
5.922
5.738
2.716
2.365
1.636
1.211
-0.000
1.87
3.20
2.00
1.39
5.72
2.93
ppm

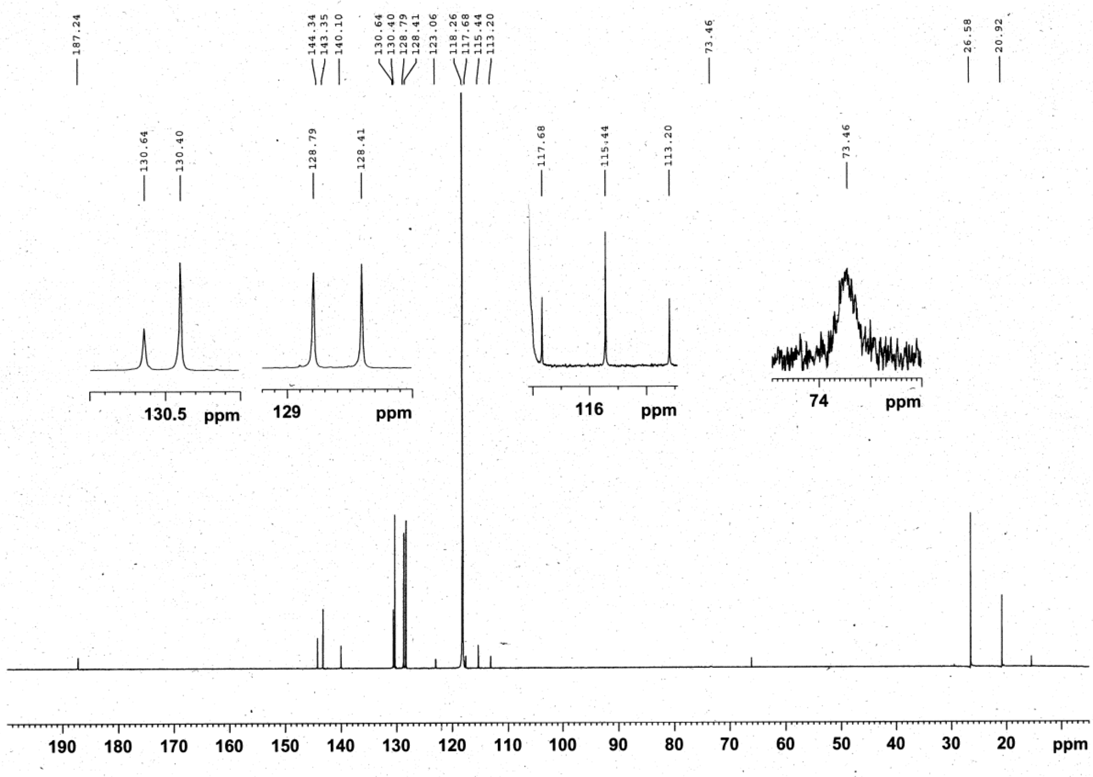
187.24
144.34
143.35
140.10
130.64
130.40
128.79
128.41
123.06
118.26
117.68
115.44
113.20
73.46
26.58
20.92
130.5
ppm
129
116
74
ppm

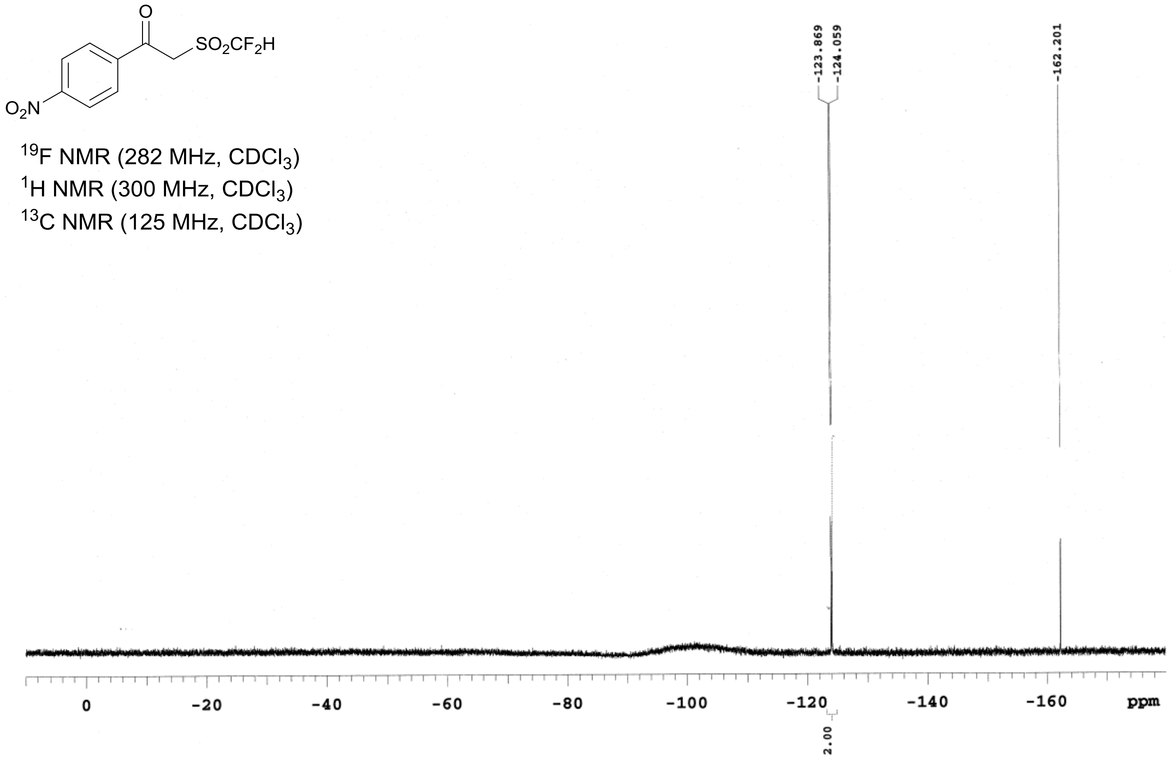
O
SO2CF2H
O2N
19F NMR (282 MHz, CDCl3)
1H NMR (300 MHz, CDCl3)
13C NMR (125 MHz, CDCl3)
-123.869
-124.059
-162.201
0
-20
-40
-60
-80
-100
-120
-140
-160
ppm
2.00

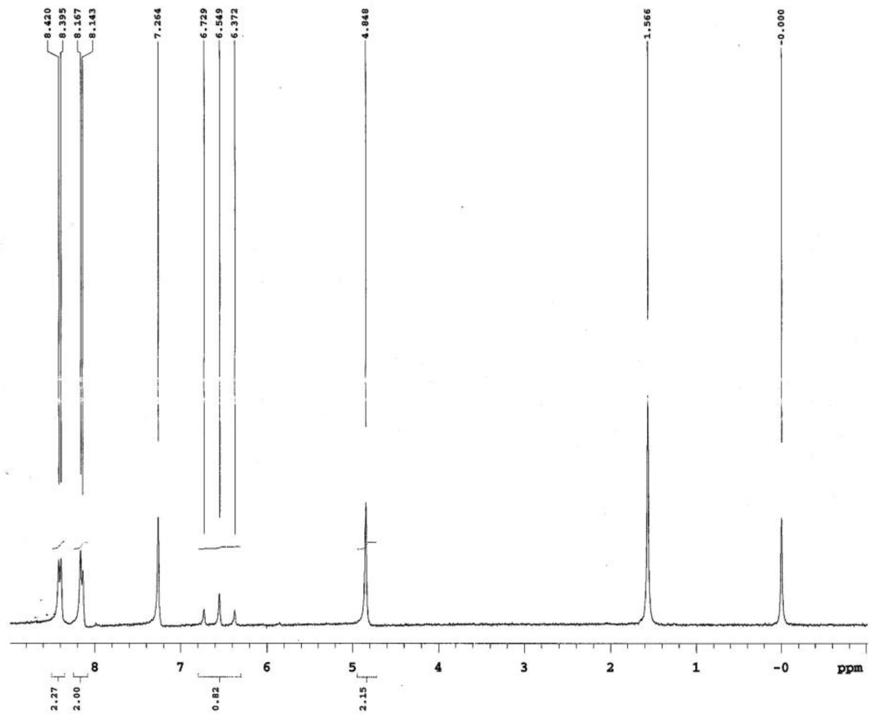
8.420
8.395
8.167
8.143
7.264
6.729
6.549
6.372
4.848
1.566
-0.000
8
7
6
5
4
3
2
1
-0
ppm
2.27
2.00
0.82
2.15

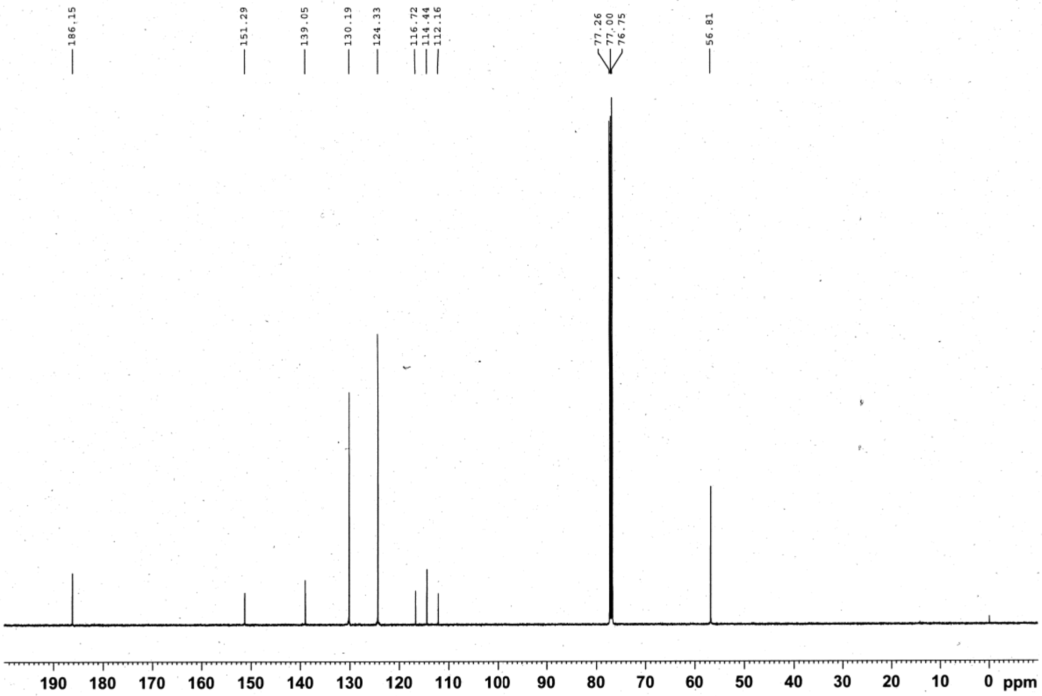
186.15
151.29
139.05
130.19
124.33
116.72
114.44
112.16
77.26
77.00
76.75
56.81
190
180
170
160
150
140
130
120
110
100
90
80
70
60
50
40
30
20
10
0
ppm

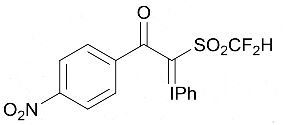

2b

^{19}F NMR (282 MHz, $(CD_3)_2CO$)
^{1}H NMR (300 MHz, $(CD_3)_2SO$)
^{13}C NMR (125 MHz, $(CD_3)_2SO$)

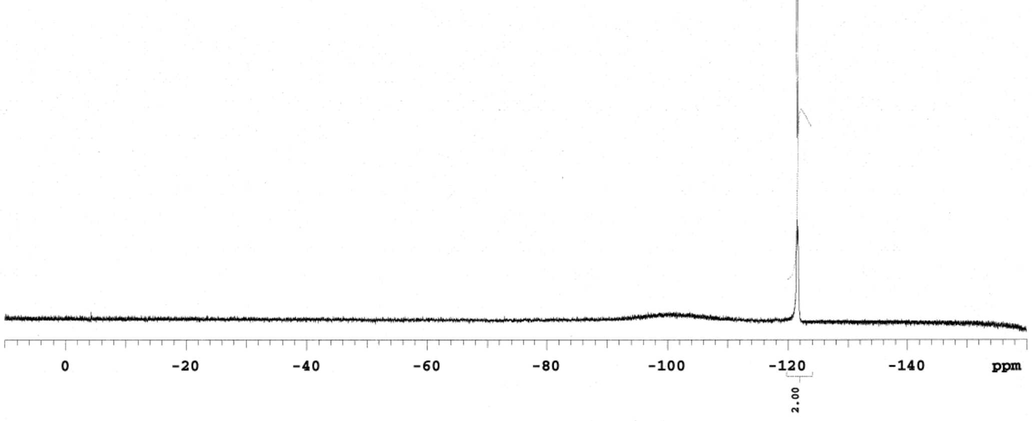

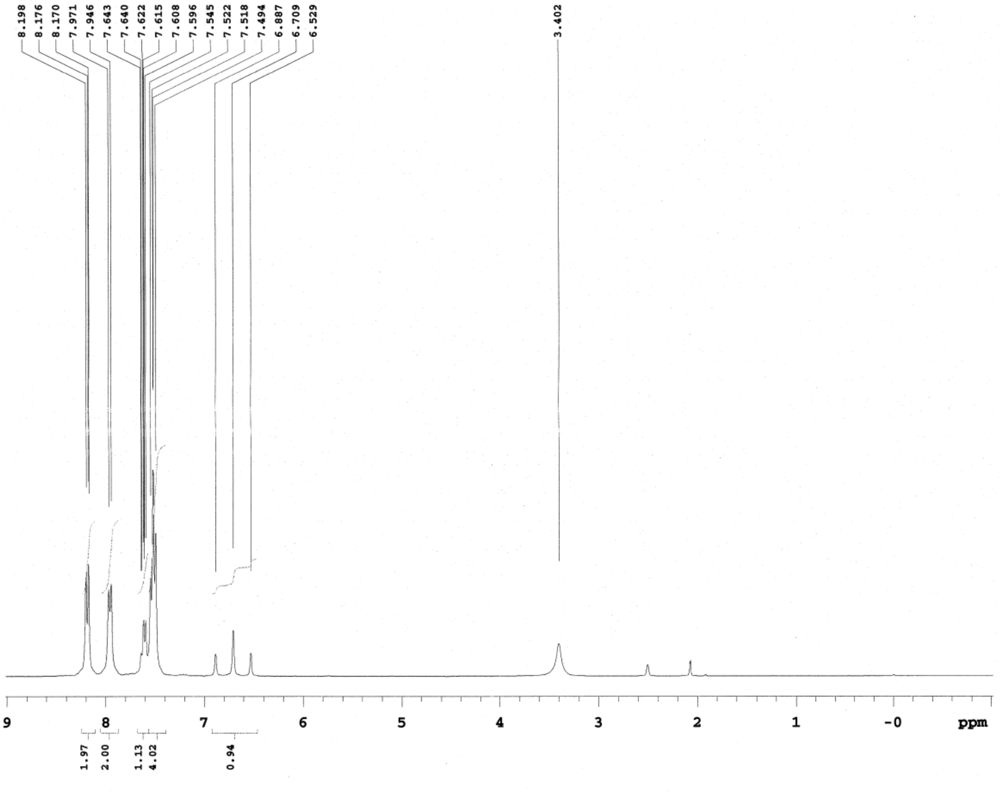

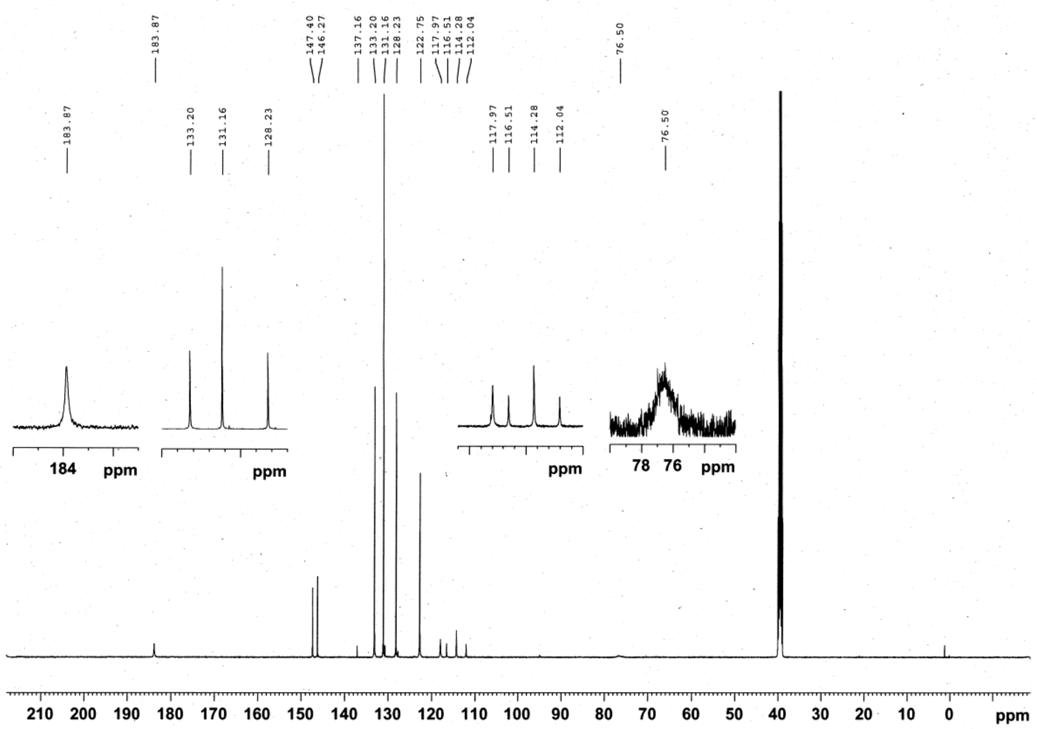

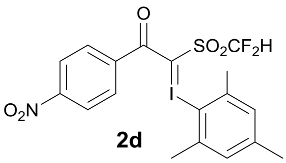

2d

^{19}F NMR (282 MHz, $CDCl_3$)
^{1}H NMR (300 MHz, $(CD_3)_2SO$)
^{13}C NMR (125 MHz, $(CD_3)_2SO$)

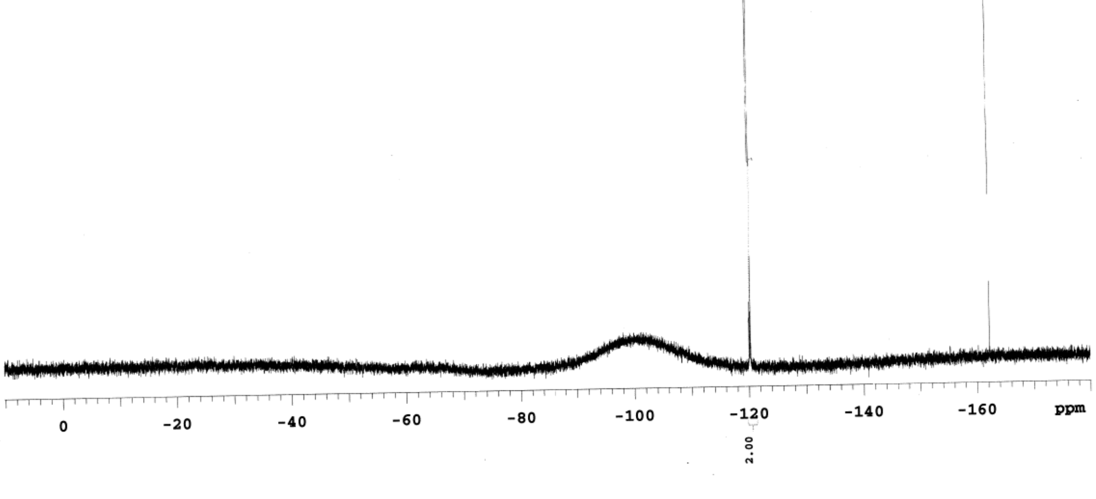

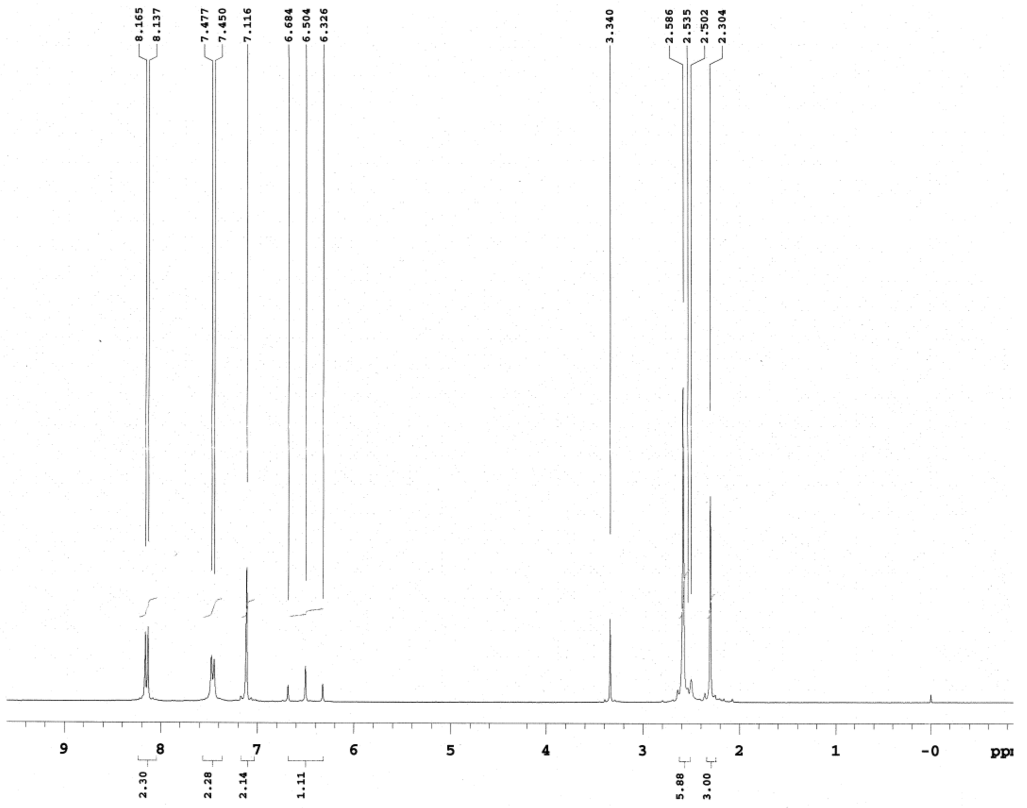
8.165
8.137
7.477
7.450
7.116
6.684
6.504
6.326
3.340
2.586
2.535
2.502
2.304
9 8 7 6 5 4 3 2 1 -0 ppm
2.30
2.28
2.14
1.11
5.88
3.00

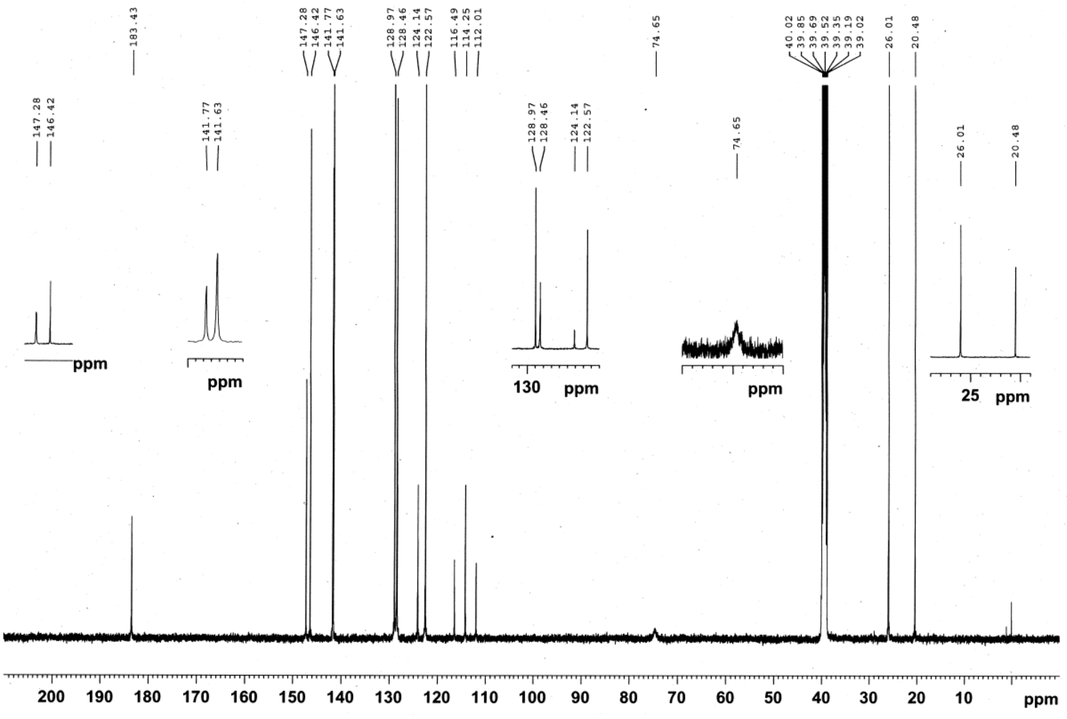
183.43
147.28
146.42
141.77
141.63
128.97
128.46
124.14
122.57
116.49
114.25
112.01
74.65
40.02
39.85
39.69
39.52
39.35
39.19
39.02
26.01
20.48
ppm
130 ppm
25 ppm
200 190 180 170 160 150 140 130 120 110 100 90 80 70 60 50 40 30 20 10 ppm

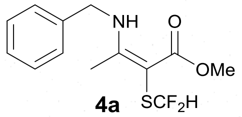

4a

^{19}F NMR (282 MHz, $CDCl_3$)
^{1}H NMR (300 MHz, $CDCl_3$)
^{13}C NMR (125 MHz, $CDCl_3$)

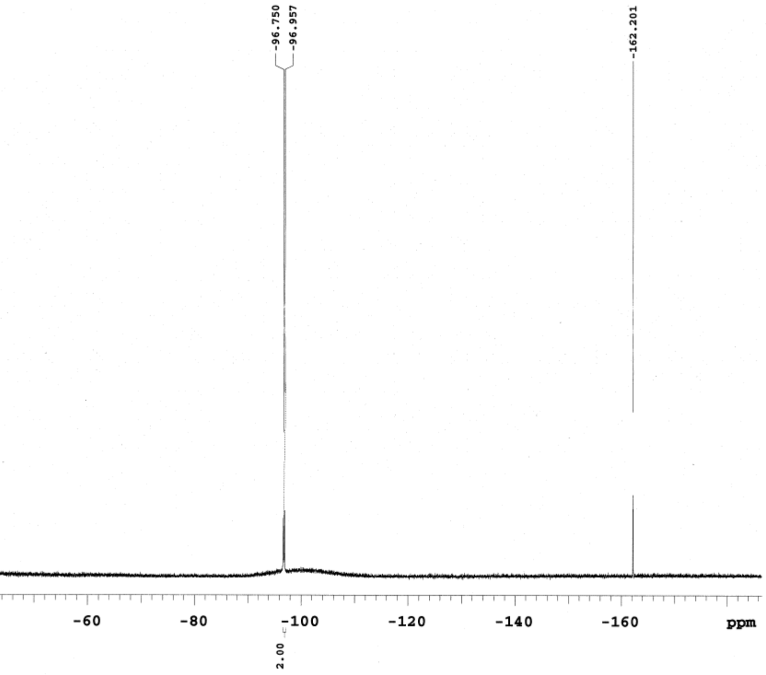

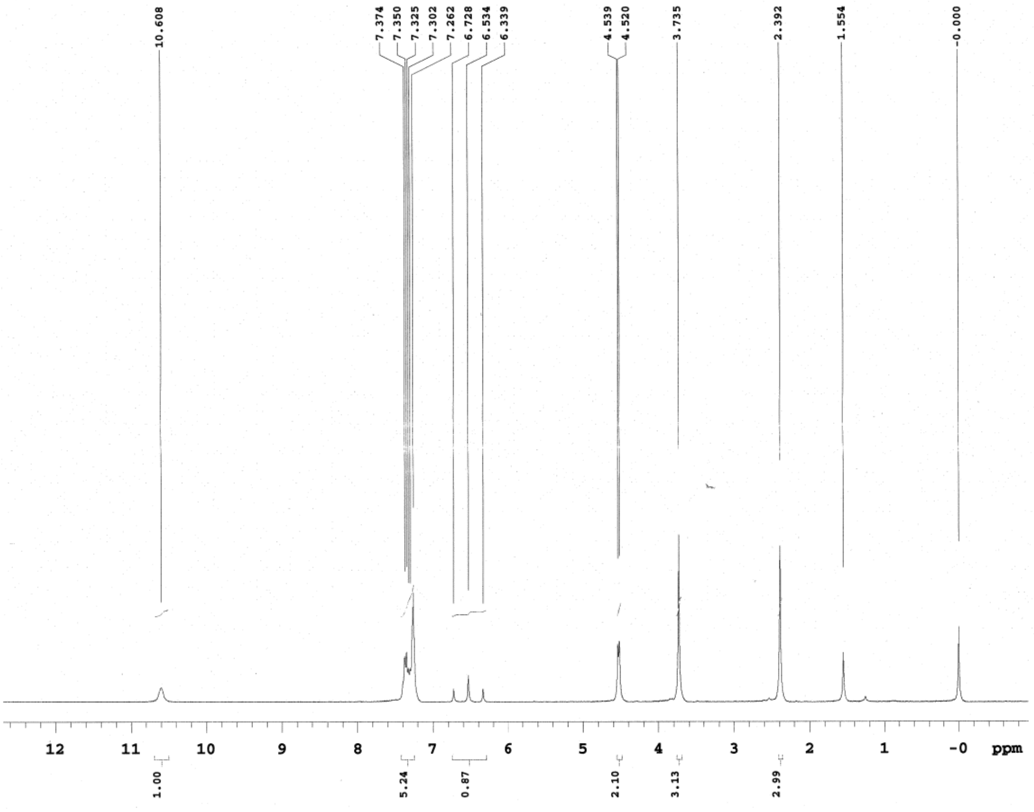

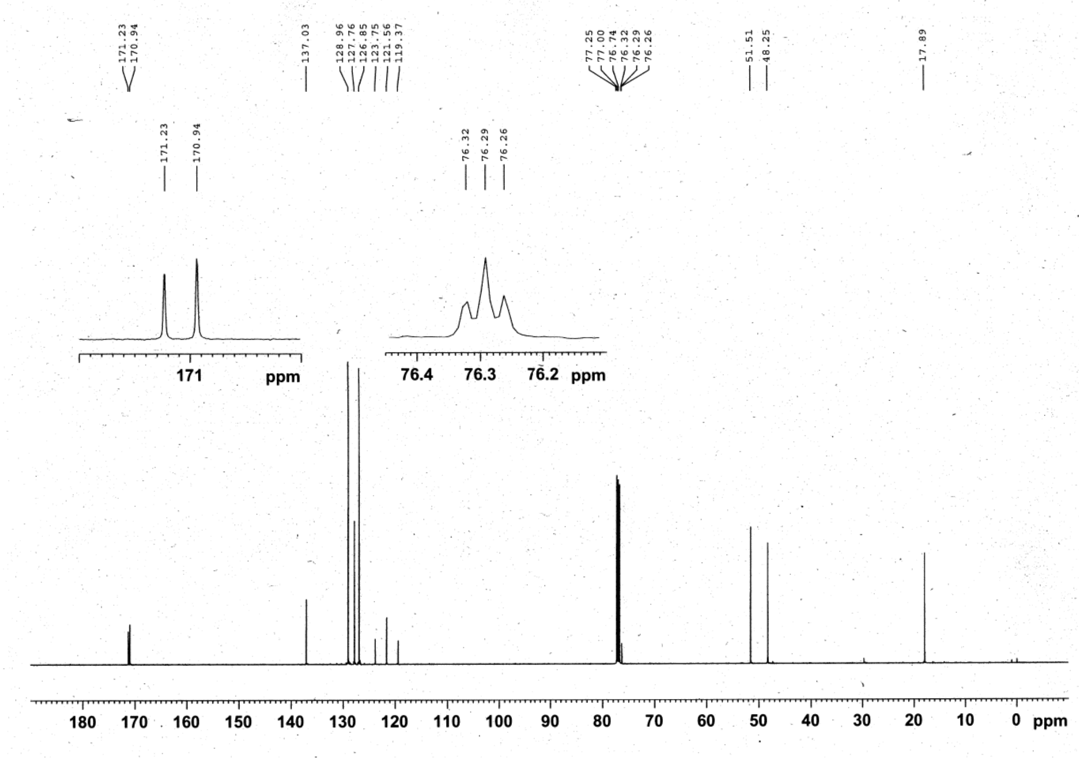

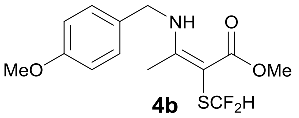

4b

^{19}F NMR (282 MHz, $CDCl_3$)
^{1}H NMR (300 MHz, $CDCl_3$)
^{13}C NMR (125 MHz, $CDCl_3$)

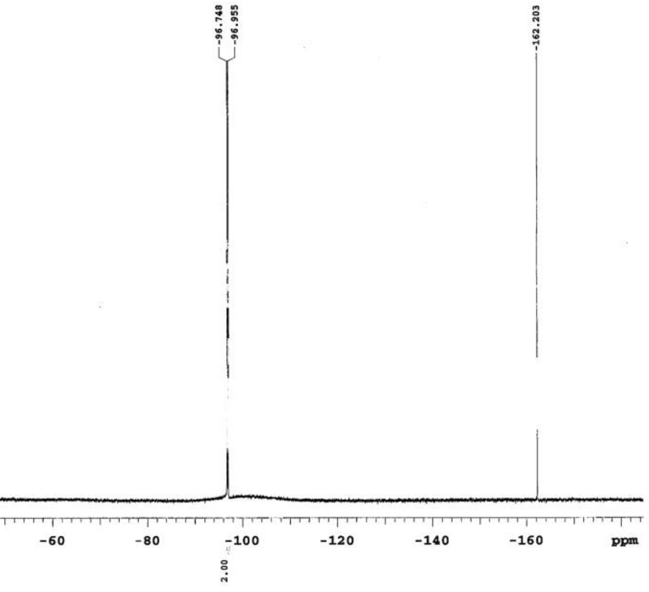

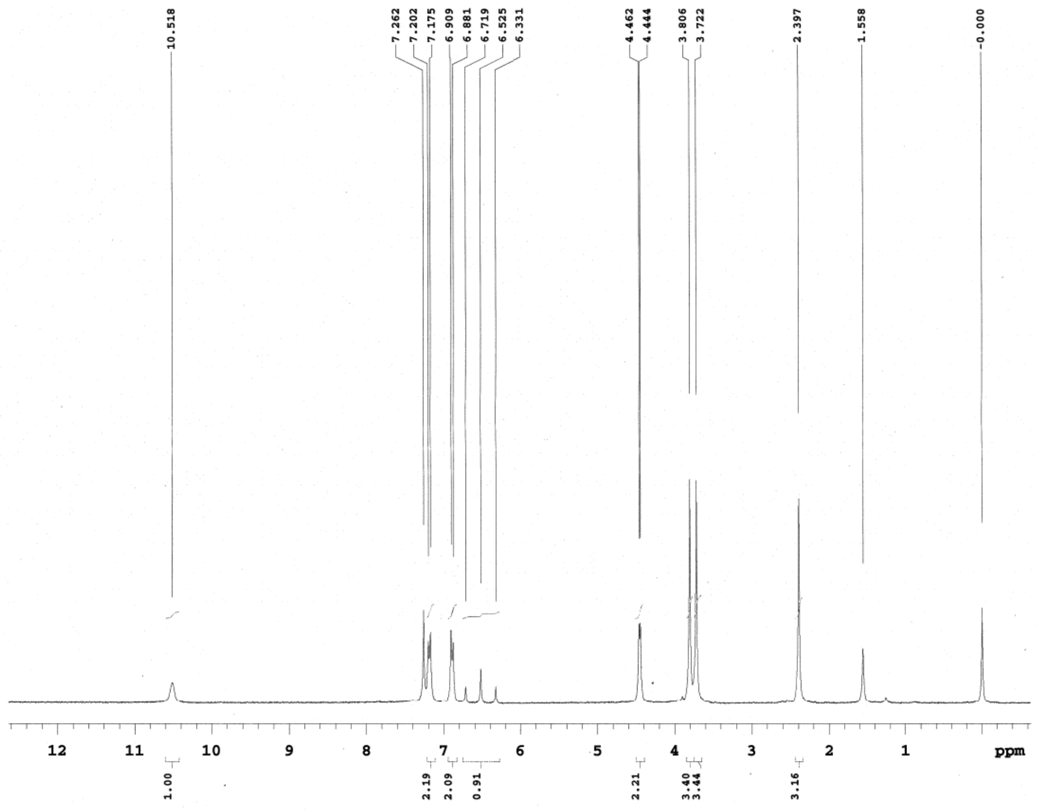
10.518
7.262
7.202
7.175
6.909
6.881
6.719
6.525
6.331
4.462
4.444
3.806
3.722
2.397
1.558
-0.000
12 11 10 9 8 7 6 5 4 3 2 1 ppm
1.00
2.19
2.09
0.91
2.21
3.40
3.44
3.16

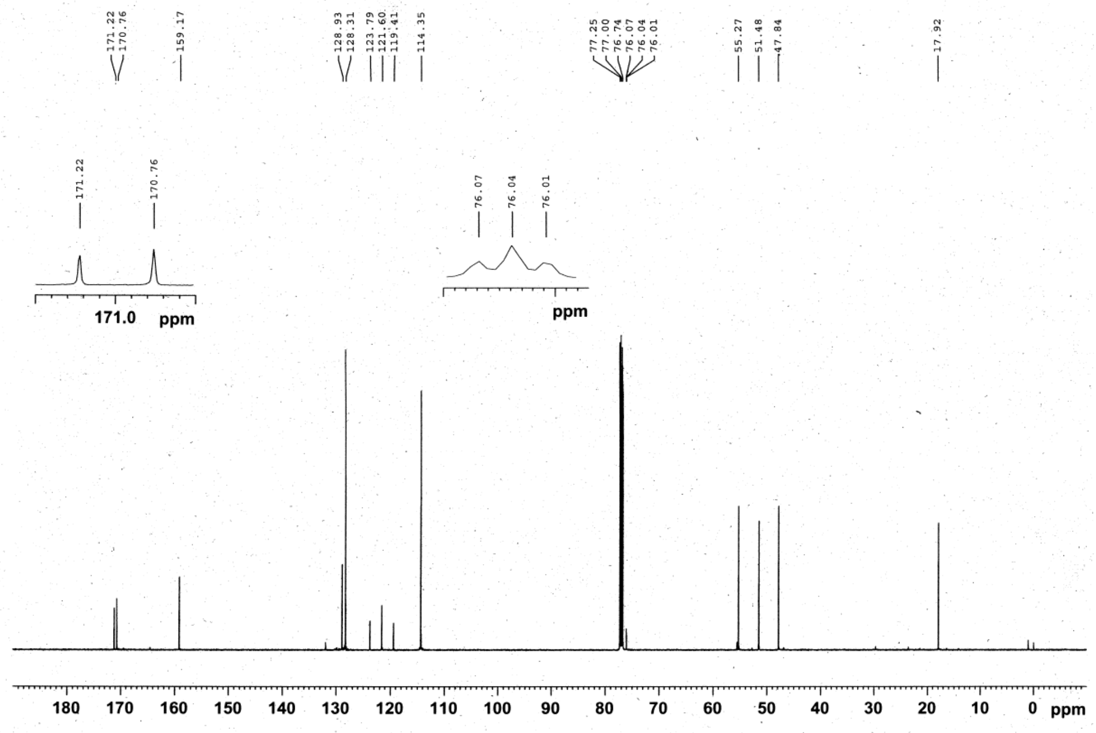
171.22
170.76
159.17
128.93
128.31
123.79
121.60
119.41
114.35
77.25
77.00
76.74
76.07
76.04
76.01
55.27
51.48
47.84
17.92
171.0 ppm
ppm
180 170 160 150 140 130 120 110 100 90 80 70 60 50 40 30 20 10 0 ppm

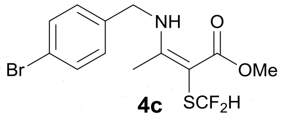

4c

^{19}F NMR (282 MHz, $CDCl_3$)
^{1}H NMR (300 MHz, $CDCl_3$)
^{13}C NMR (125 MHz, $CDCl_3$)

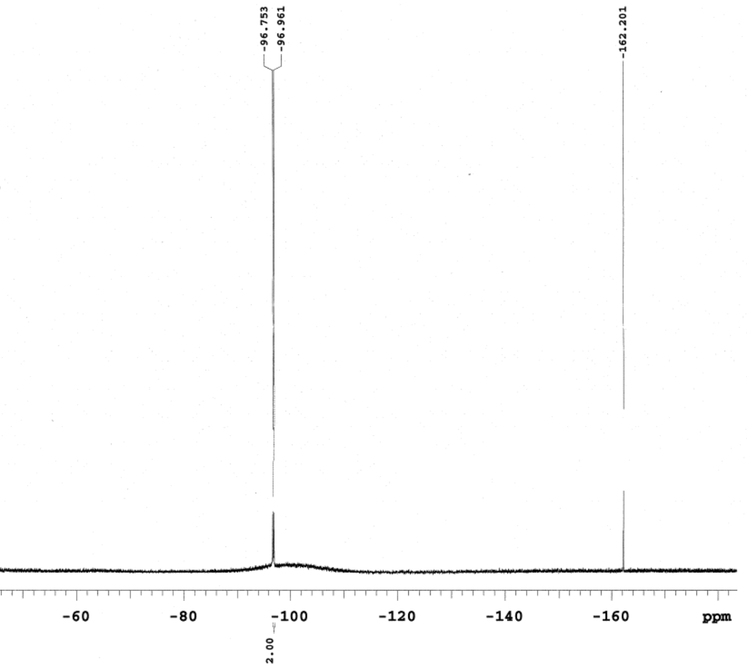

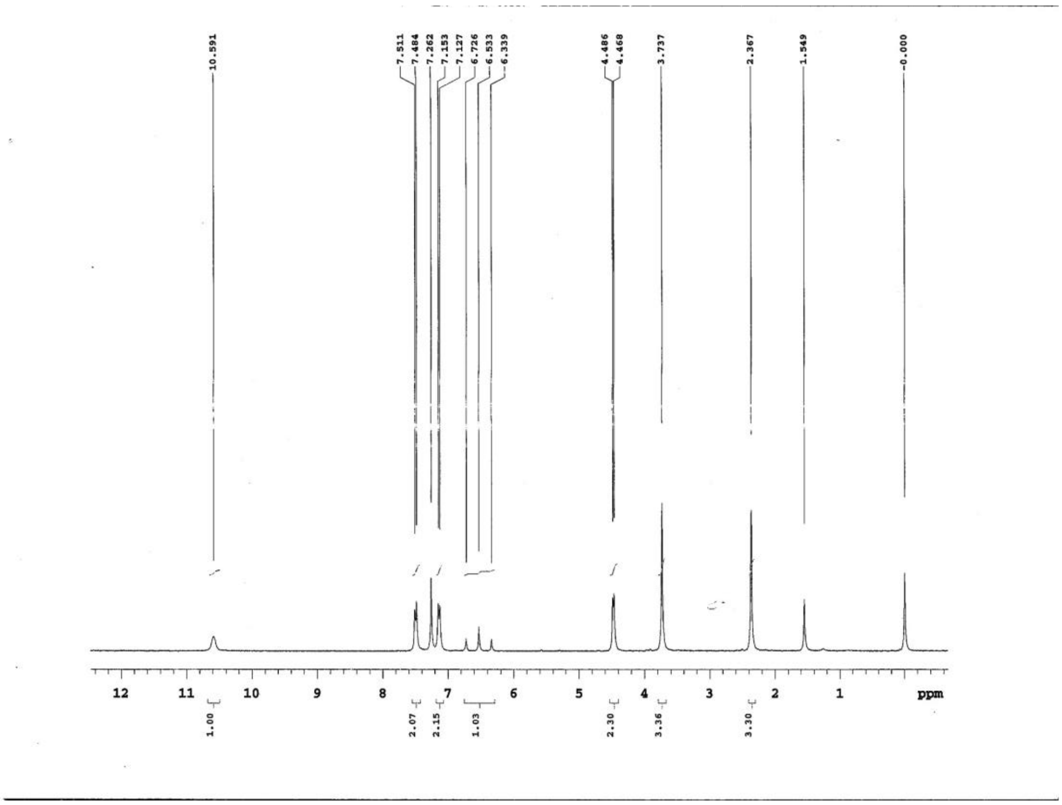

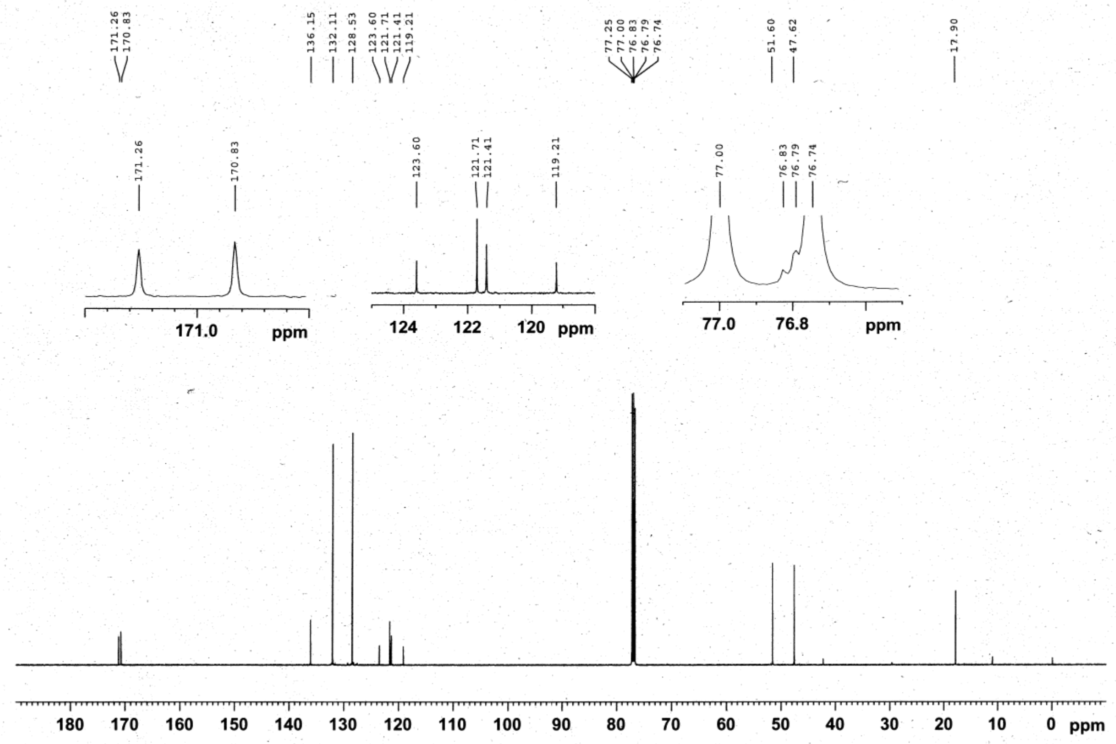

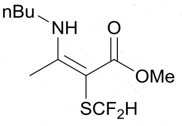

4d

^{19}F NMR (282 MHz, $CDCl_3$)

^{1}H NMR (300 MHz, $CDCl_3$)

^{13}C NMR (125 MHz, $CDCl_3$)

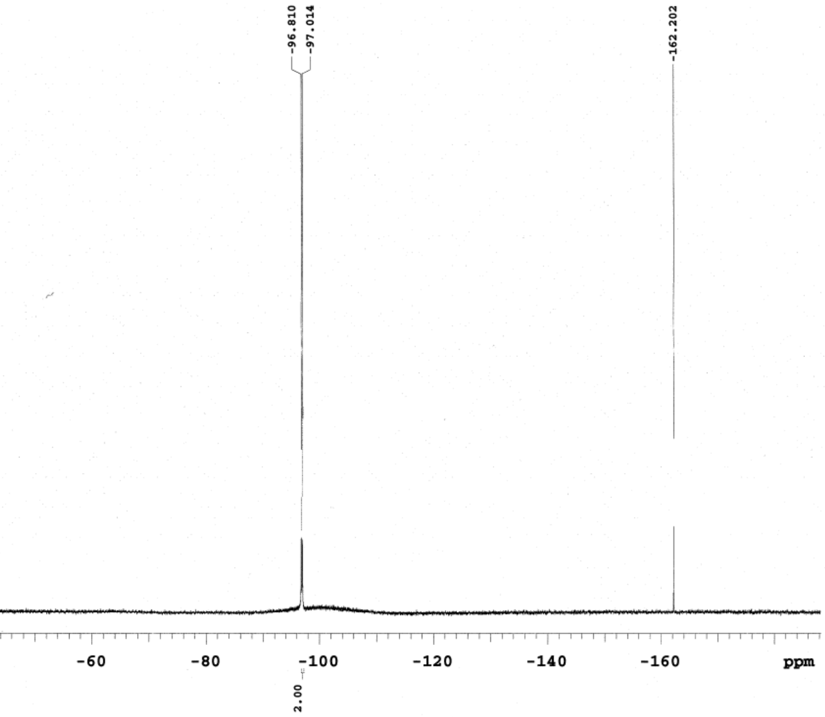

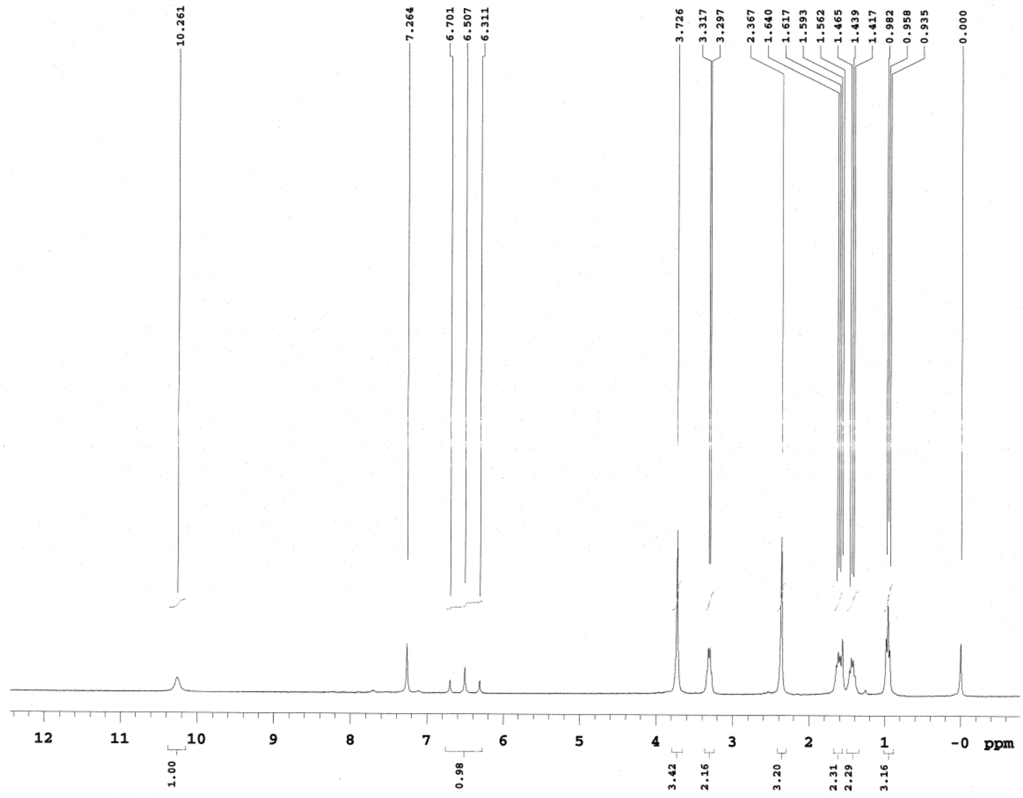

10.261
7.264
6.701
6.507
6.311
3.726
3.317
3.297
2.367
1.640
1.617
1.593
1.562
1.465
1.439
1.417
0.982
0.958
0.935
0.000
12 11 10 9 8 7 6 5 4 3 2 1 -0 ppm
1.00
0.98
3.42
2.16
3.20
2.31
2.29
3.16

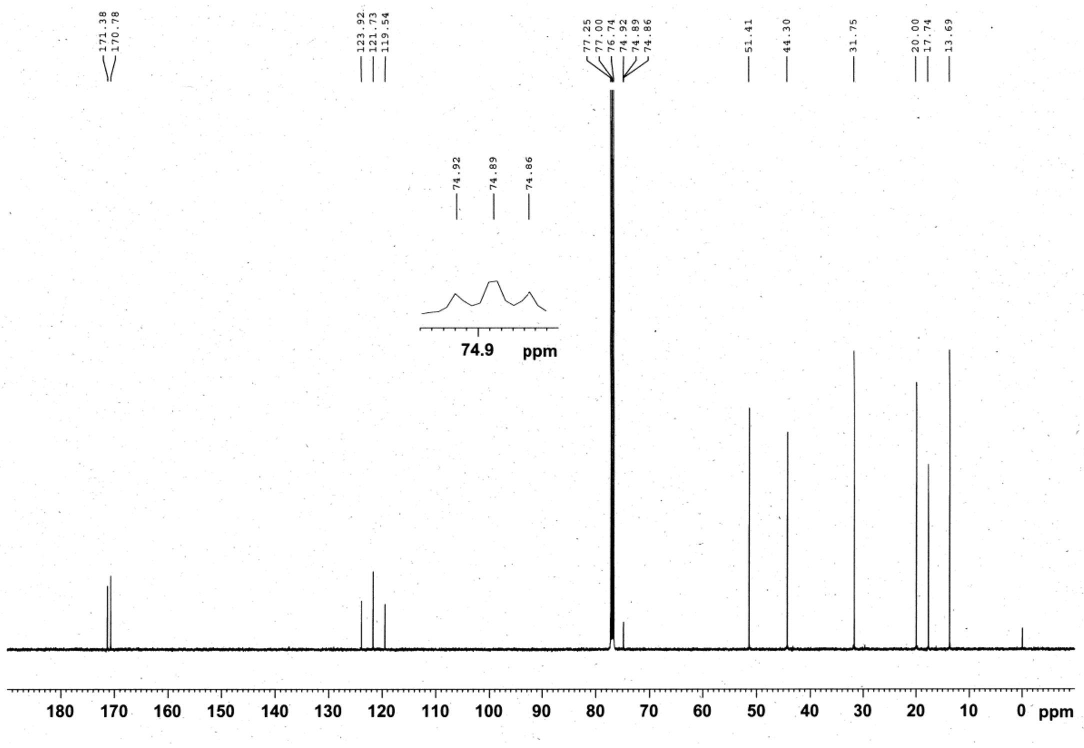

171.38
170.78
123.92
121.73
119.54
77.25
77.00
76.74
74.92
74.89
74.86
51.41
44.30
31.75
20.00
17.74
13.69
74.92
74.89
74.86
74.9 ppm
180 170 160 150 140 130 120 110 100 90 80 70 60 50 40 30 20 10 0 ppm

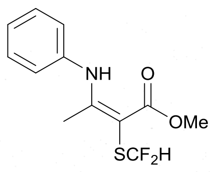

4e

^{19}F NMR (282 MHz, $CDCl_3$)
^{1}H NMR (300 MHz, $CDCl_3$)
^{13}C NMR (125 MHz, $CDCl_3$)

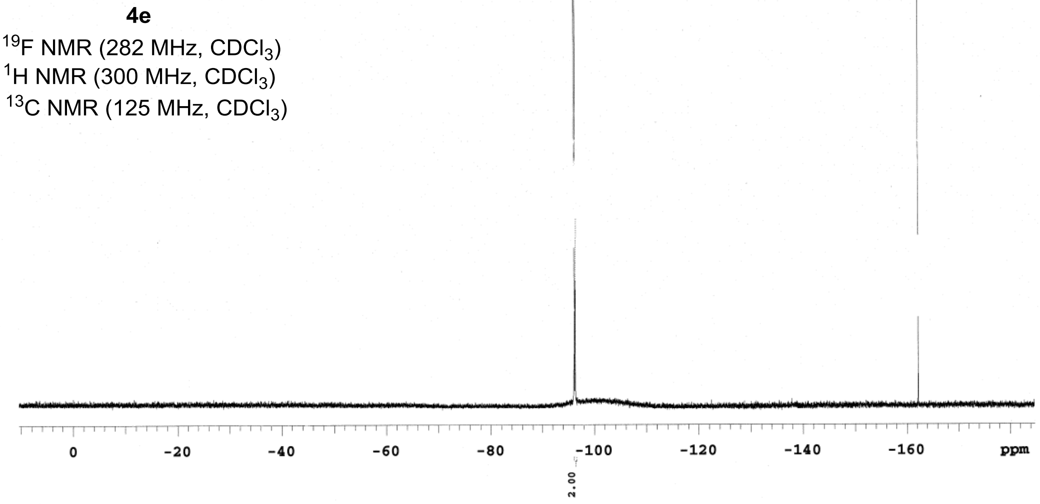

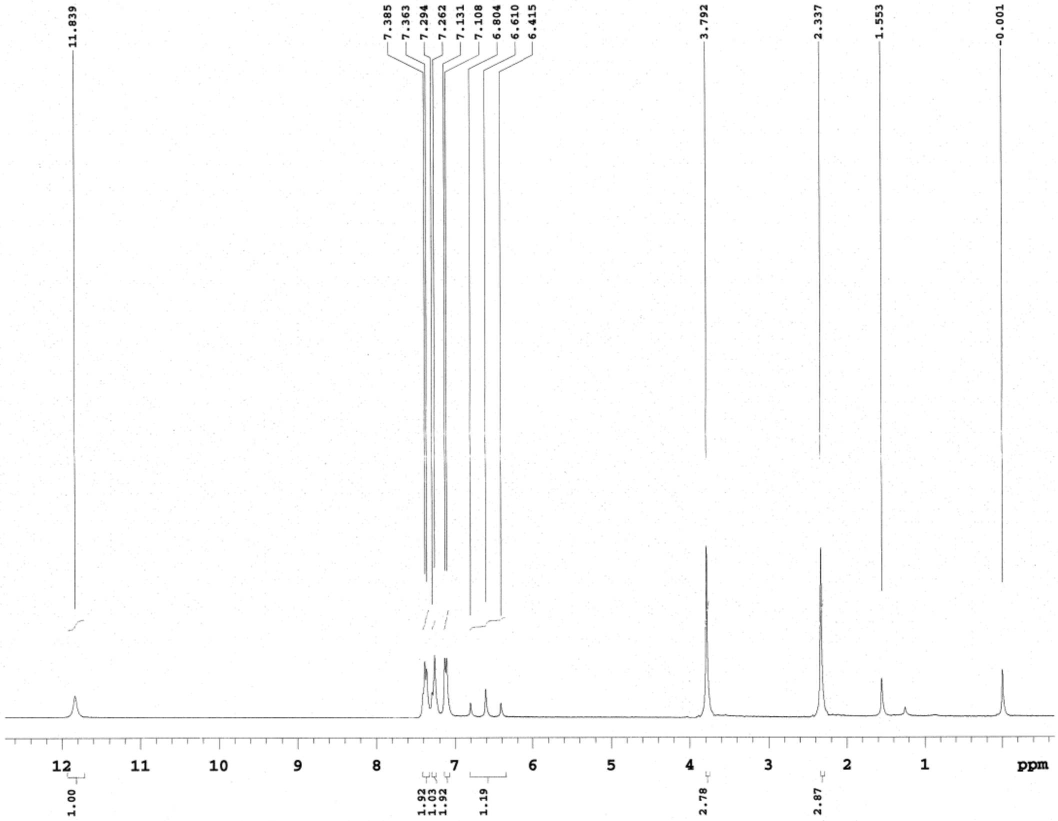

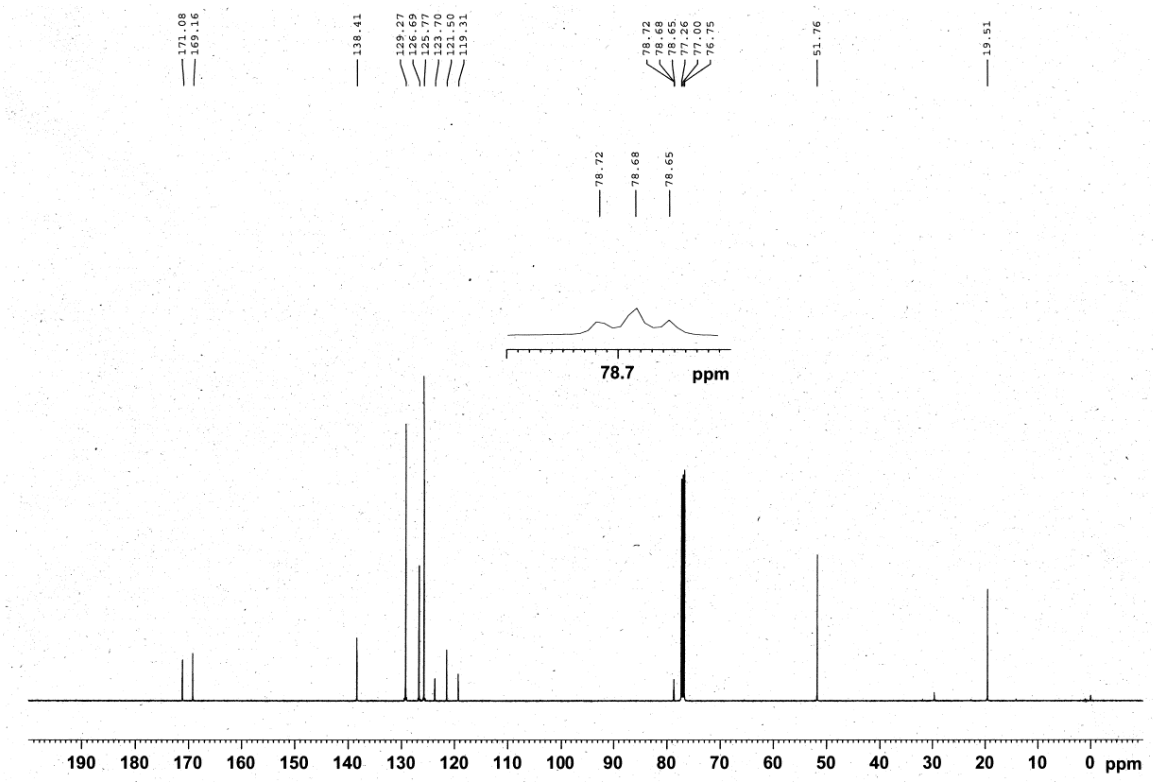

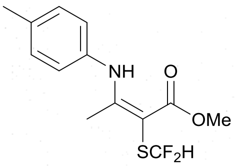

4f

^{19}F NMR (282 MHz, $CDCl_3$)
^{1}H NMR (300 MHz, $CDCl_3$)
^{13}C NMR (125 MHz, $CDCl_3$)

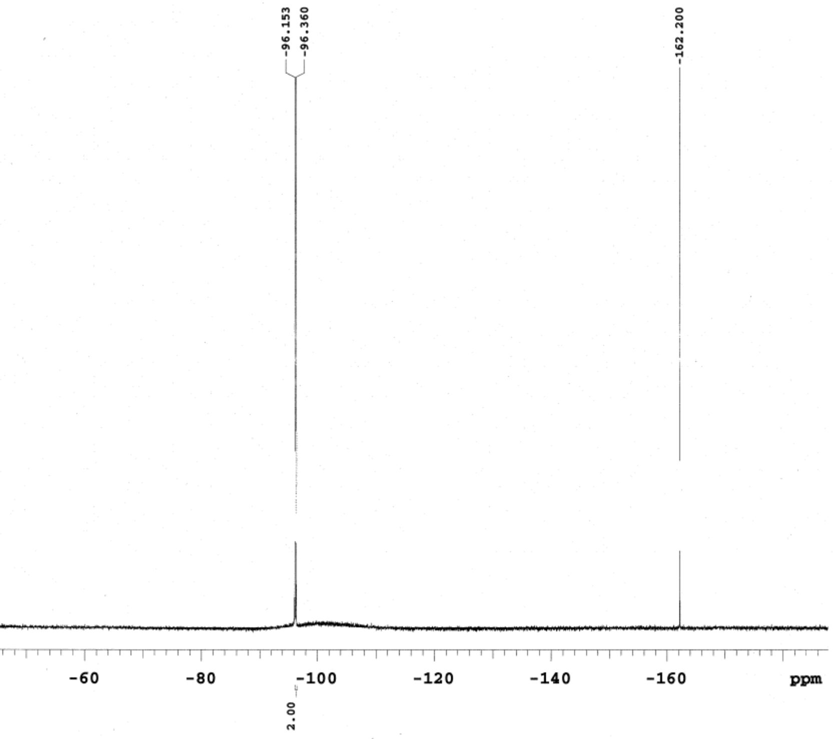

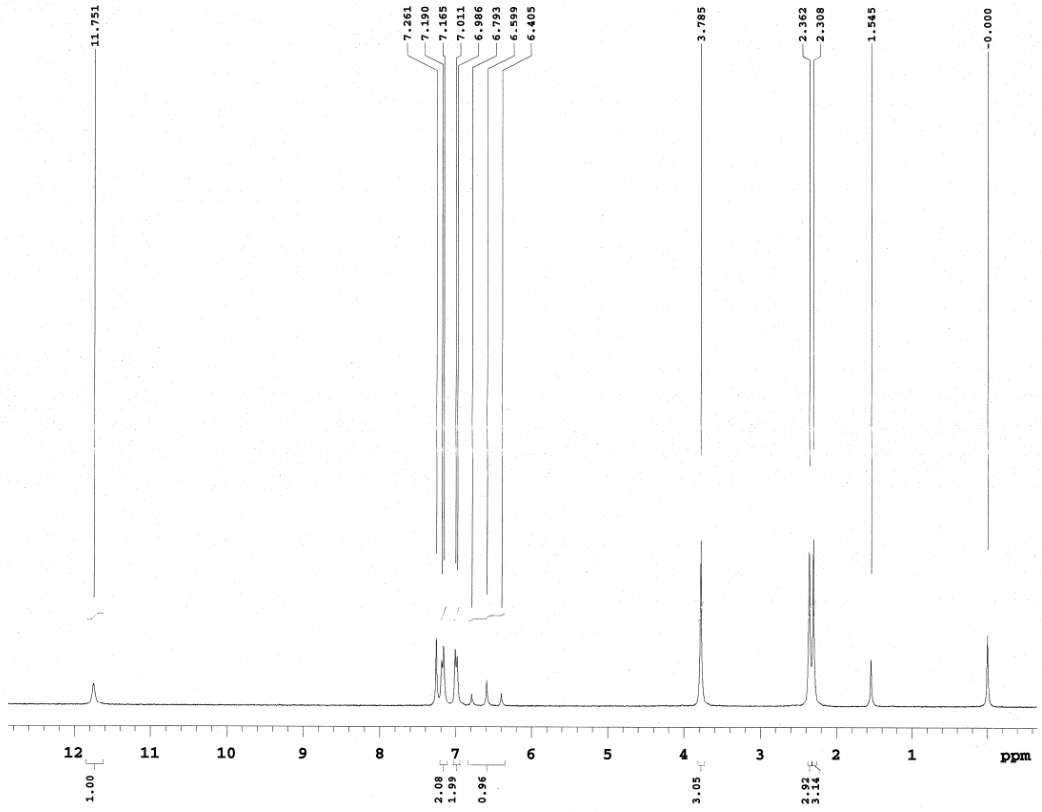
11.751
7.261
7.190
7.165
7.011
6.986
6.793
6.599
6.405
3.785
2.362
2.308
1.545
-0.000
12 11 10 9 8 7 6 5 4 3 2 1 ppm
1.00
2.08
1.99
0.96
3.05
2.92
3.14

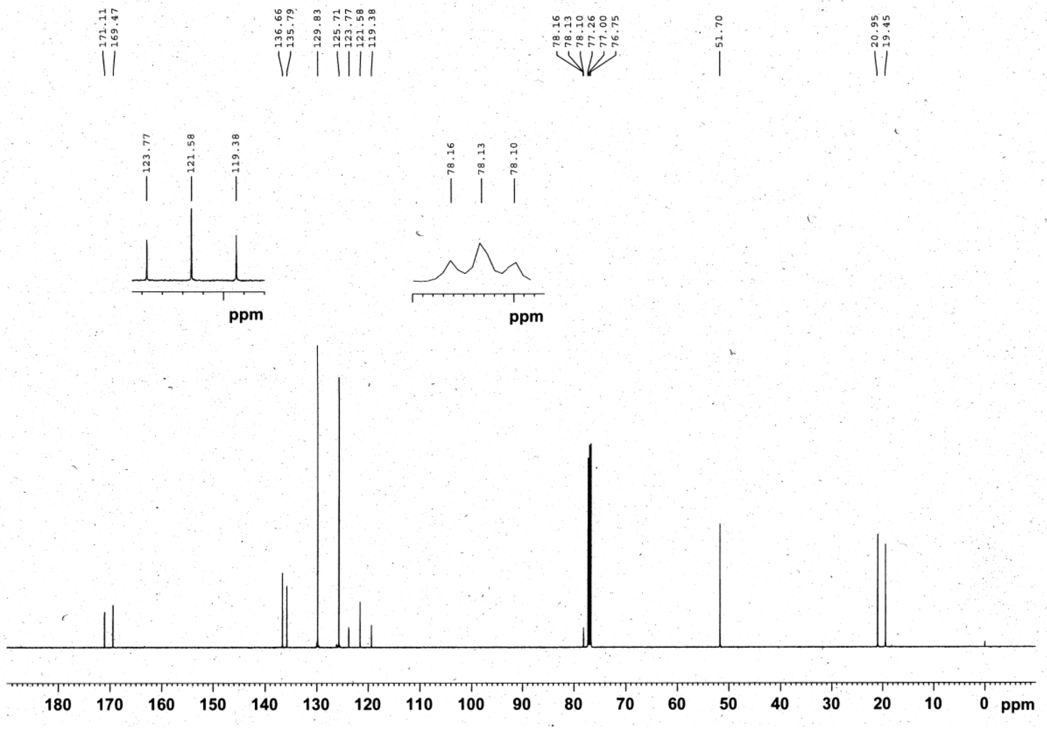
171.11
169.47
136.66
135.79
129.83
125.71
123.77
121.58
119.38
78.16
78.13
78.10
77.26
77.00
76.75
51.70
20.95
19.45
123.77
121.58
119.38
ppm
78.16
78.13
78.10
ppm
180 170 160 150 140 130 120 110 100 90 80 70 60 50 40 30 20 10 0 ppm

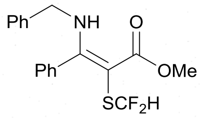

4g

^{19}F NMR (282 MHz, $CDCl_3$)
^{1}H NMR (300 MHz, $CDCl_3$)
^{13}C NMR (125 MHz, $CDCl_3$)

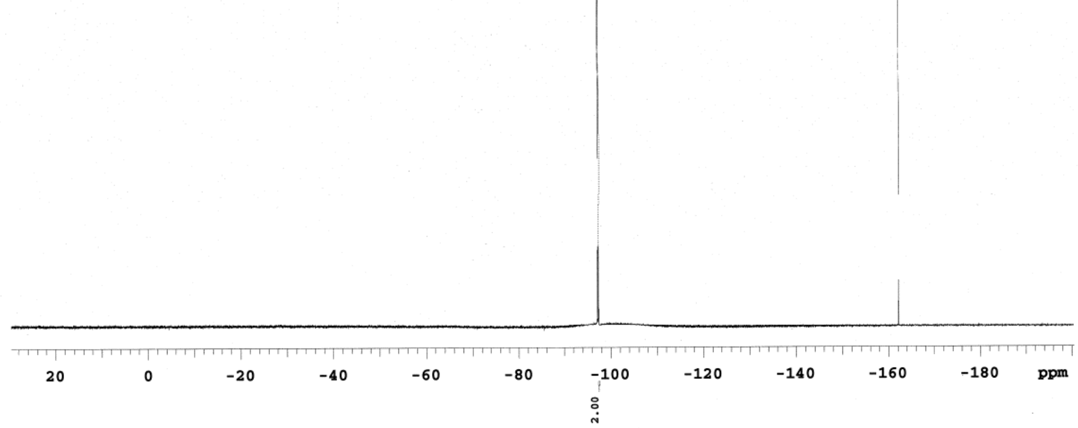

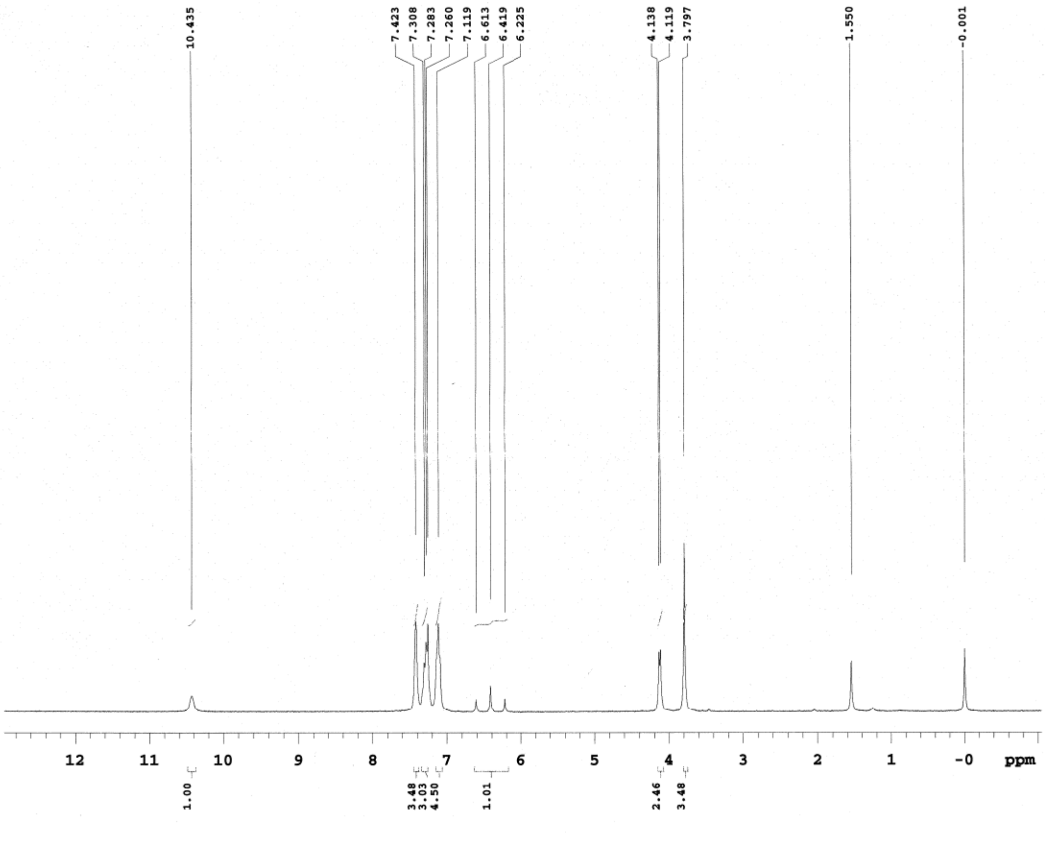

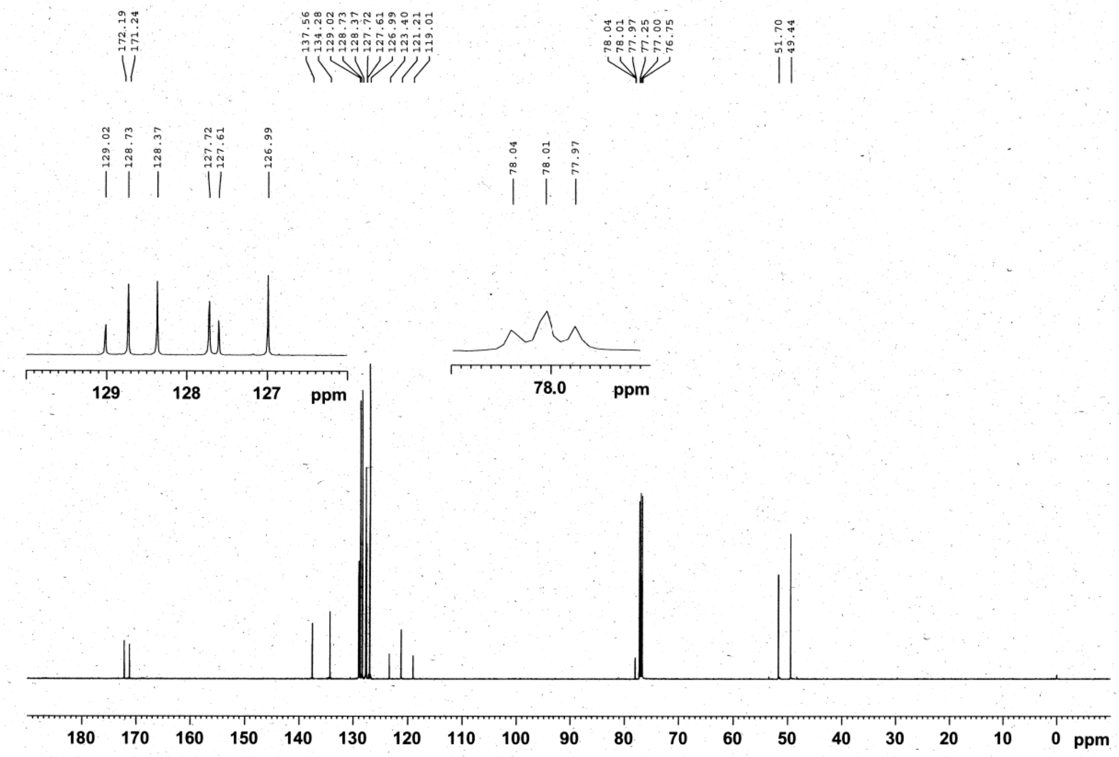

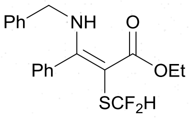

4h

^{19}F NMR (282 MHz, $CDCl_3$)
^{1}H NMR (300 MHz, $CDCl_3$)
^{13}C NMR (125 MHz, $CDCl_3$)

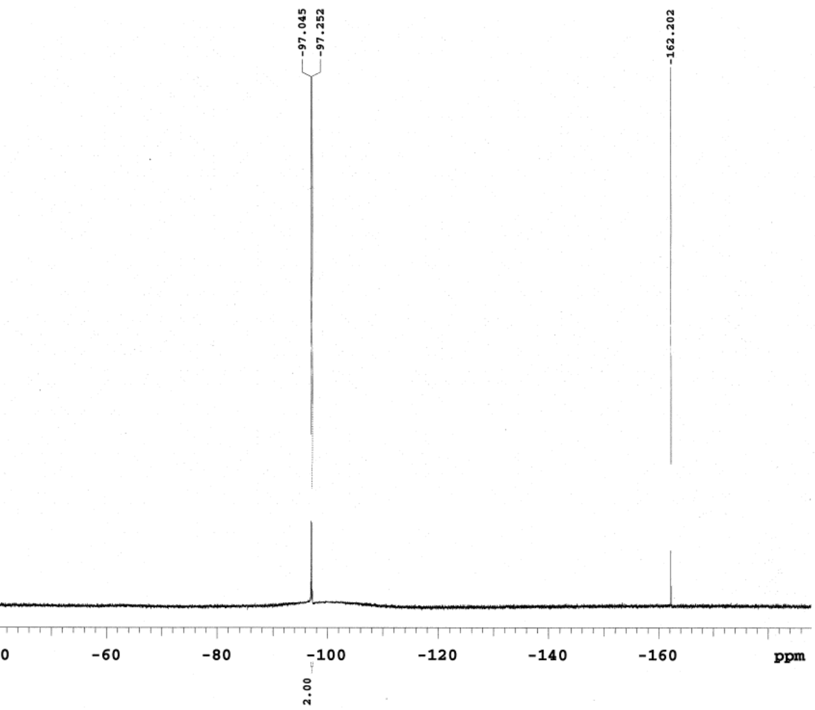

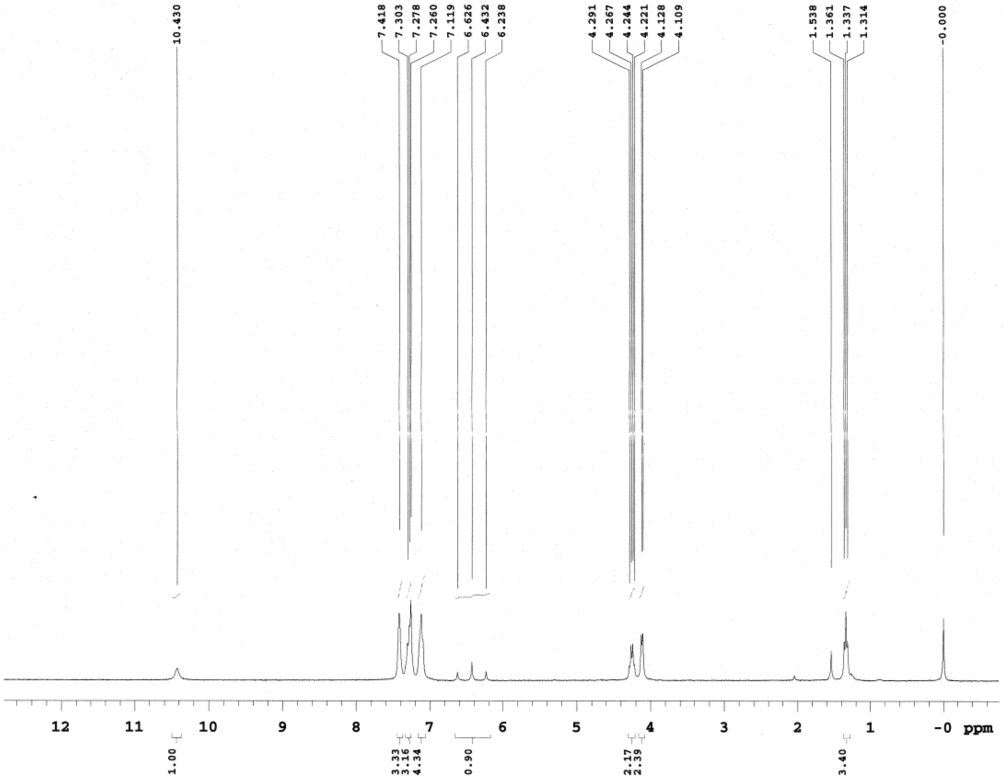
10.430
7.418
7.303
7.278
7.260
7.119
6.626
6.432
6.238
4.291
4.267
4.244
4.221
4.128
4.109
1.538
1.361
1.337
1.314
-0.000
1.00
3.33
3.16
4.34
0.90
2.17
2.39
3.40
ppm

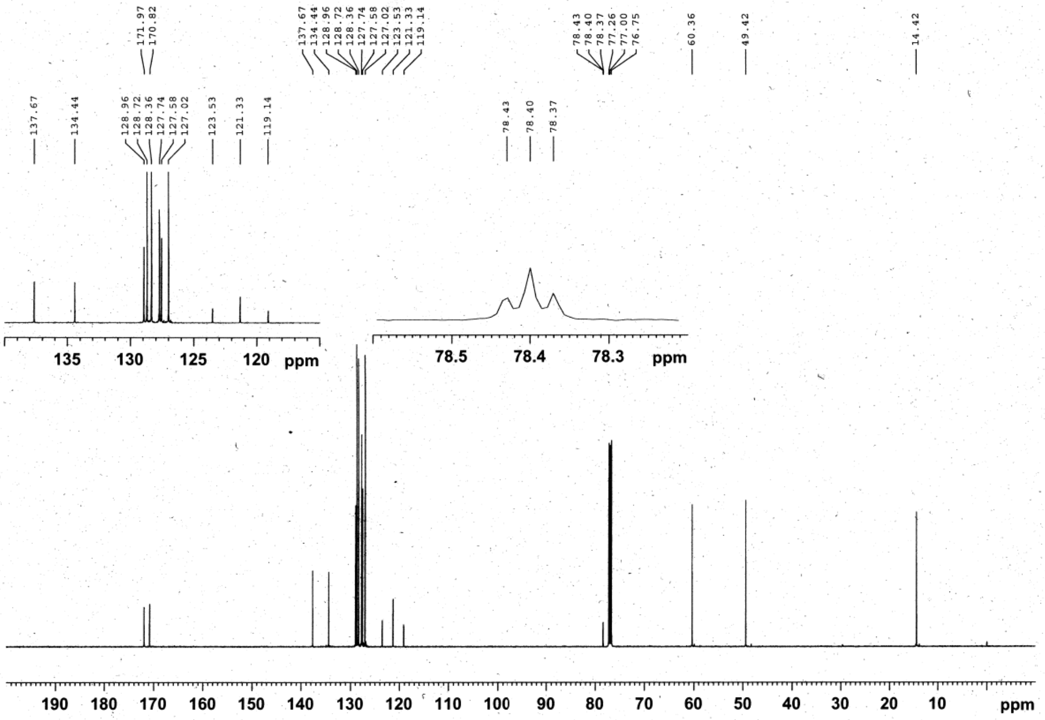
171.97
170.82
137.67
134.44
128.96
128.72
128.36
127.74
127.58
127.02
123.53
121.33
119.14
78.43
78.40
78.37
77.26
77.00
76.75
60.36
49.42
14.42
ppm

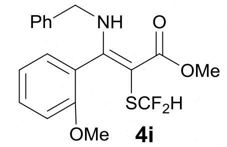

4i

^{19}F NMR (282 MHz, $CDCl_3$)
^{1}H NMR (300 MHz, $CDCl_3$)
^{13}C NMR (125 MHz, $CDCl_3$)

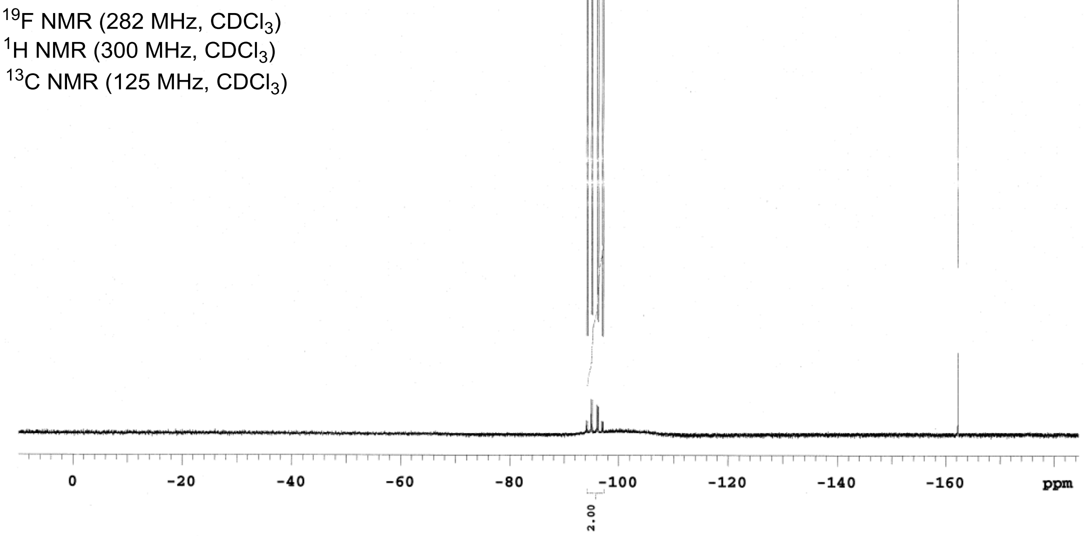

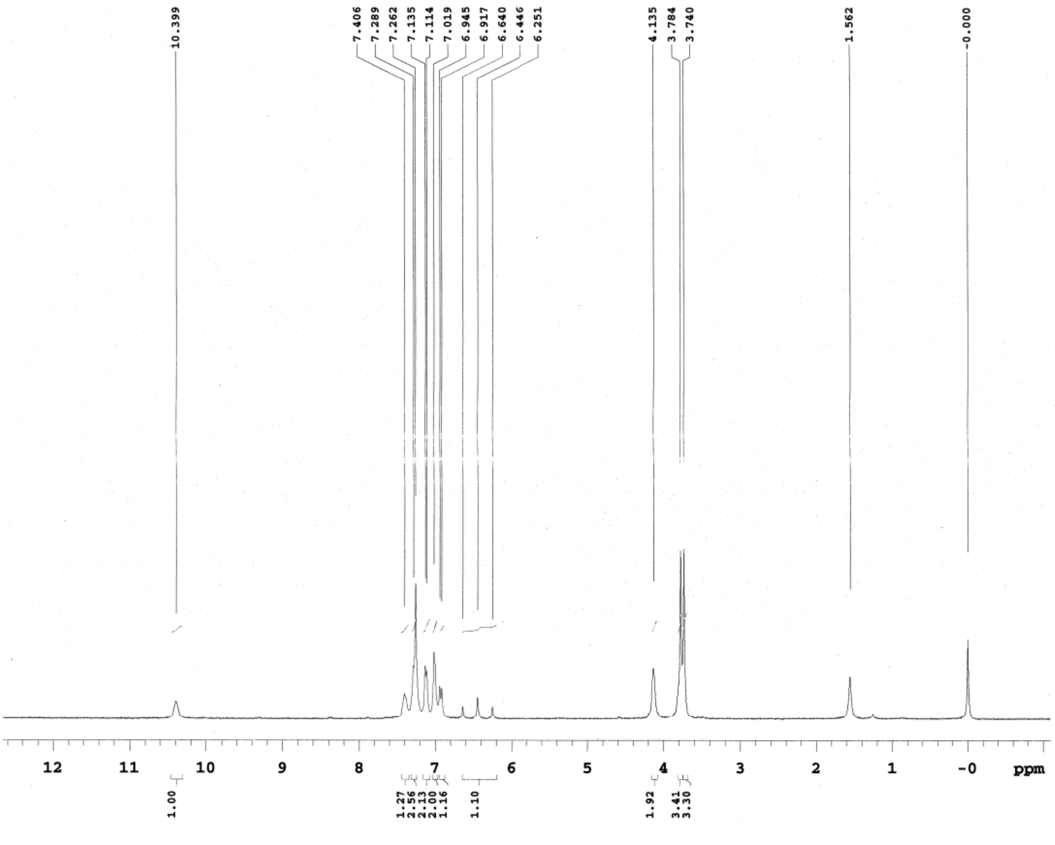

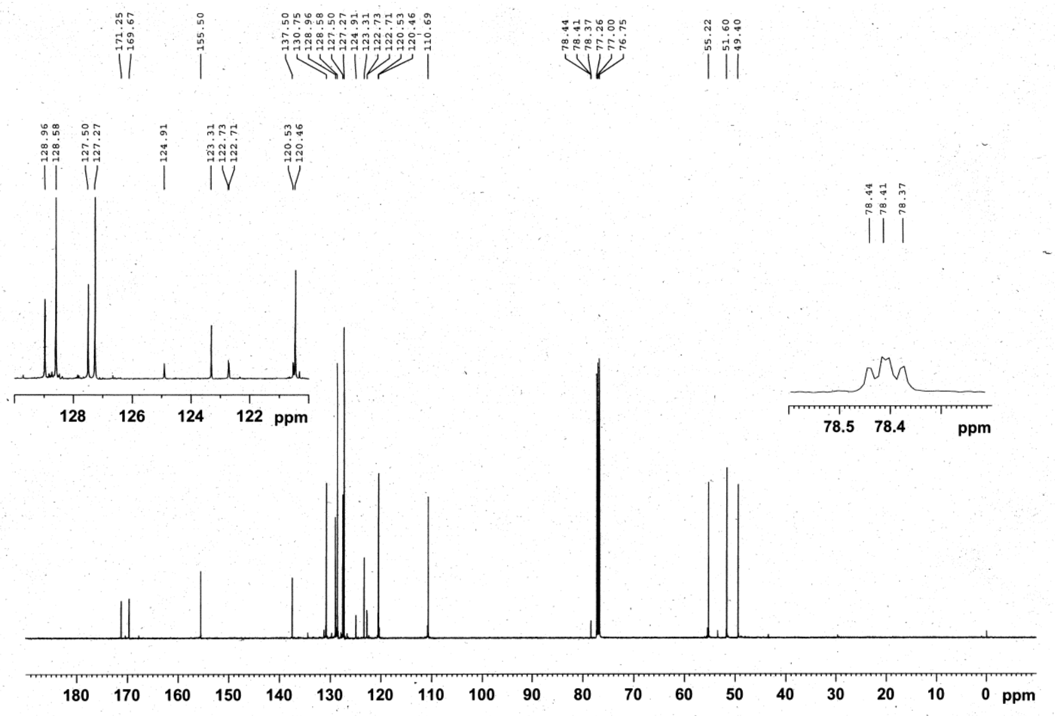

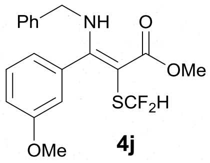

4j

^{19}F NMR (282 MHz, $CDCl_3$)
^{1}H NMR (300 MHz, $CDCl_3$)
^{13}C NMR (125 MHz, $CDCl_3$)

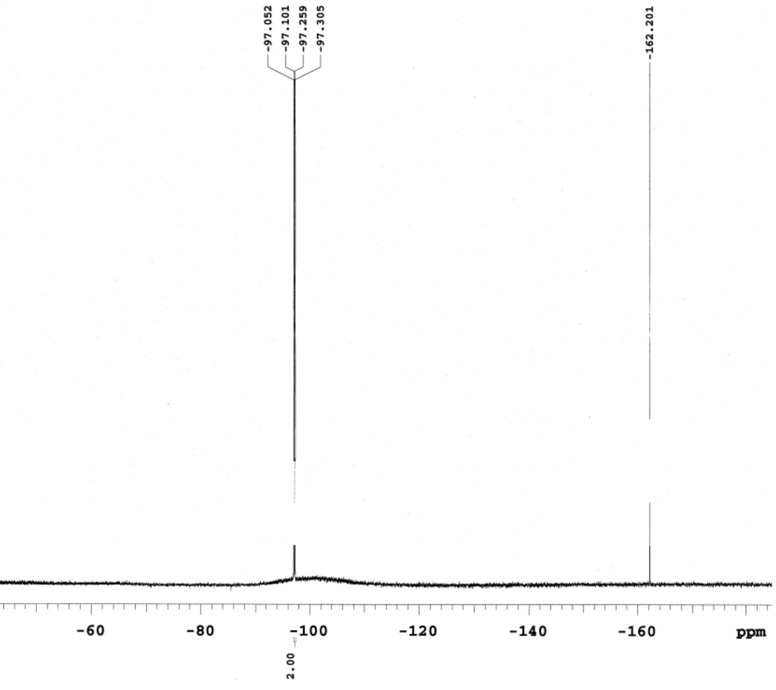

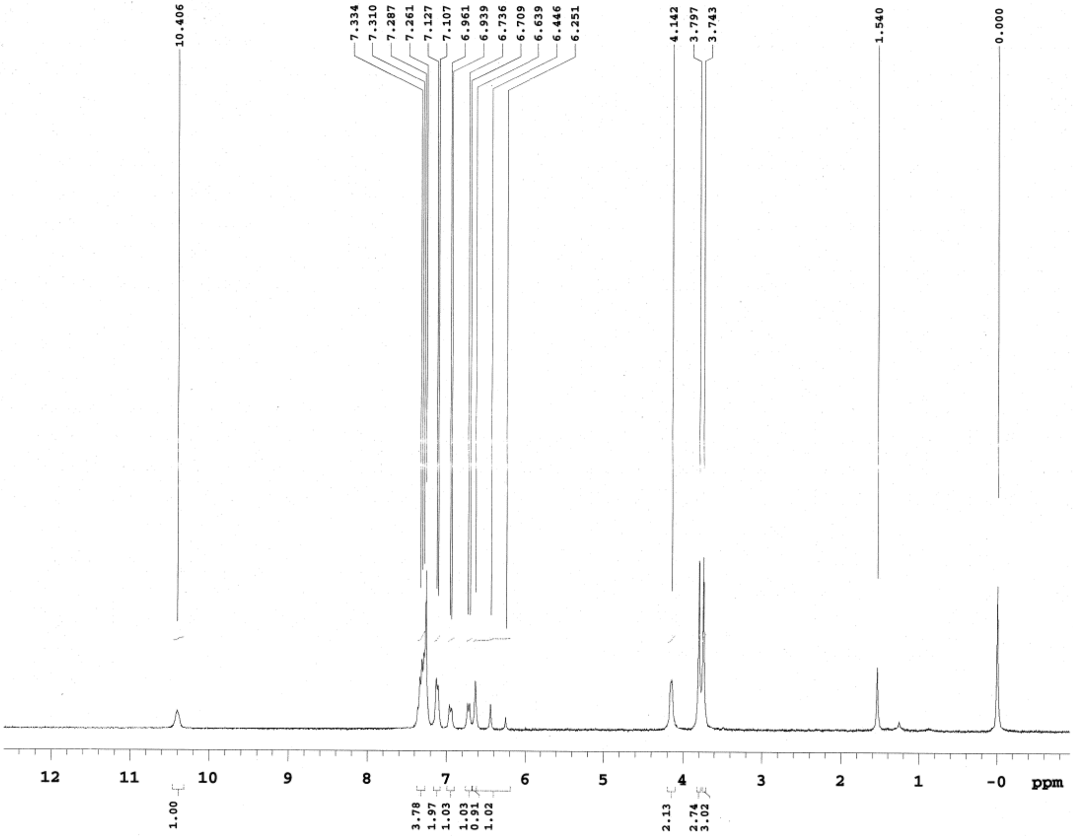
10.406
7.334 7.310 7.287 7.261 7.127 7.107 6.961 6.939 6.736 6.709 6.639 6.446 6.251
4.142 3.797 3.743
1.540
0.000
12 11 10 9 8 7 6 5 4 3 2 1 -0 ppm
1.00
3.78 1.97 1.03 1.03 0.91 1.02
2.13
2.74 3.02

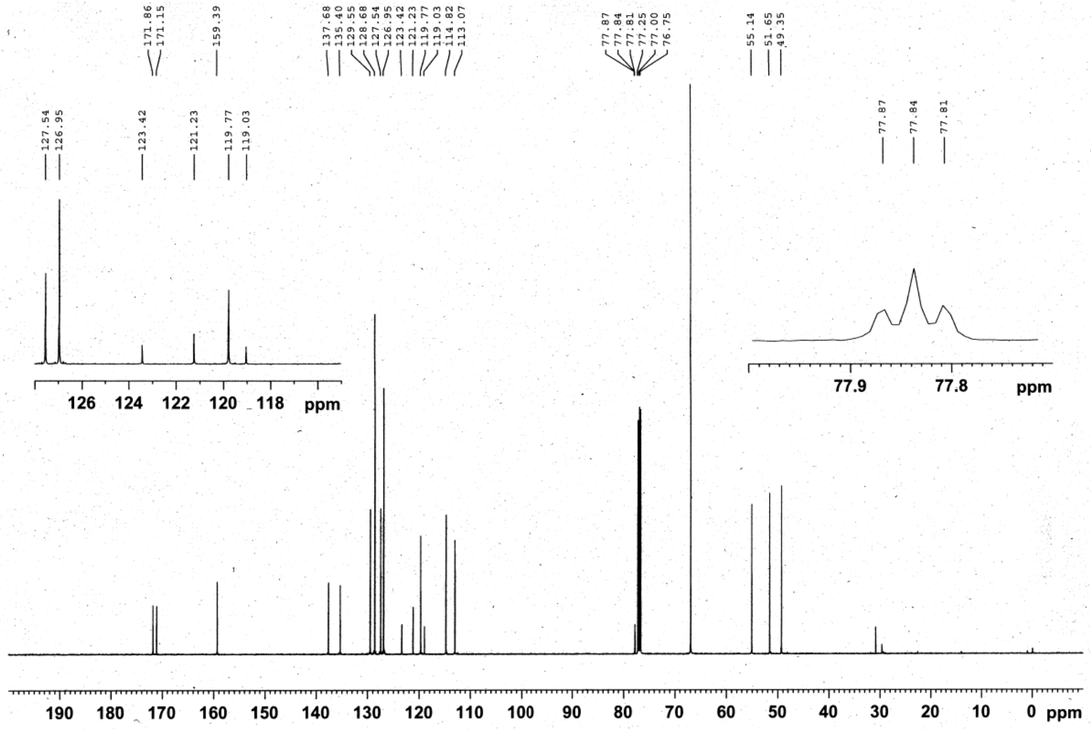
171.86 171.15
159.39
137.68 135.40 129.55 128.68 127.54 126.95 123.42 121.23 119.77 119.03 114.82 113.07
77.87 77.84 77.81 77.25 77.00 76.75
55.14 51.65 49.35
127.54 126.95 123.42 121.23 119.77 119.03
126 124 122 120 118 ppm
77.87 77.84 77.81
77.9 77.8 ppm
190 180 170 160 150 140 130 120 110 100 90 80 70 60 50 40 30 20 10 0 ppm

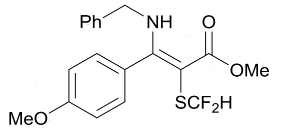

4k

^{19}F NMR (282 MHz, $CDCl_3$)
^{1}H NMR (300 MHz, $CDCl_3$)
^{13}C NMR (125 MHz, $CDCl_3$)

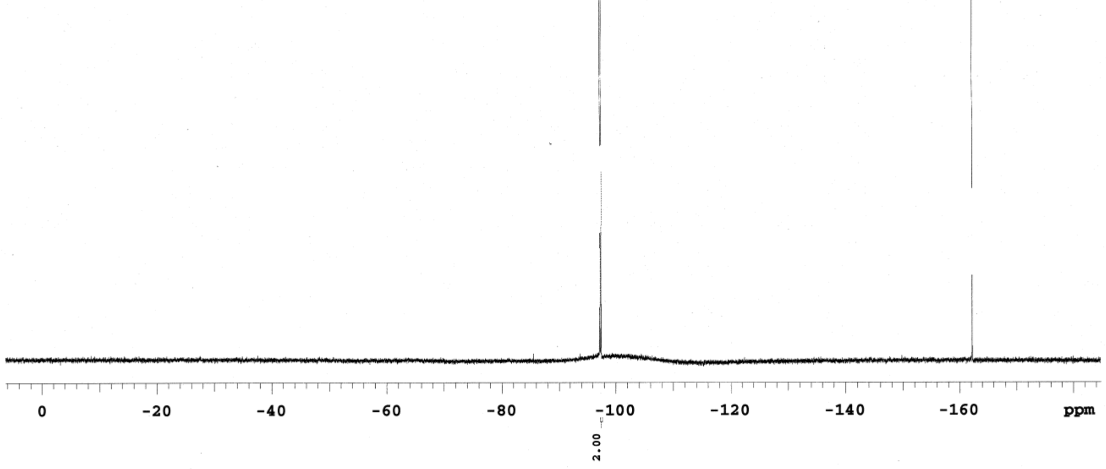

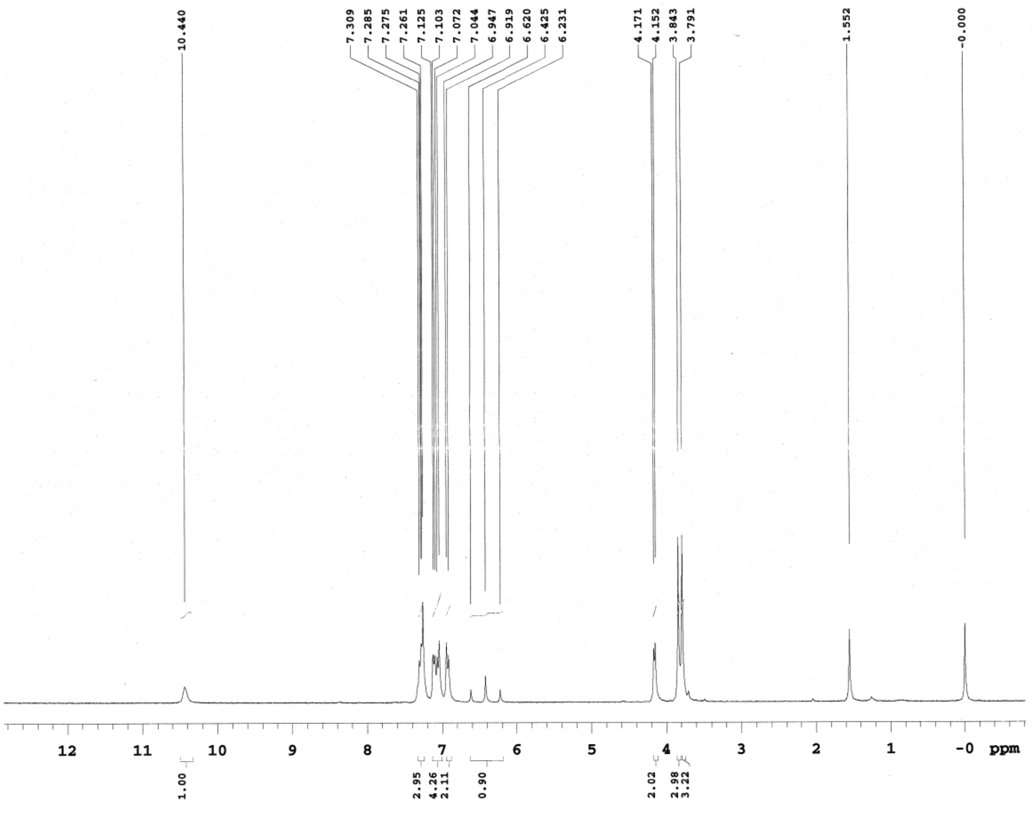

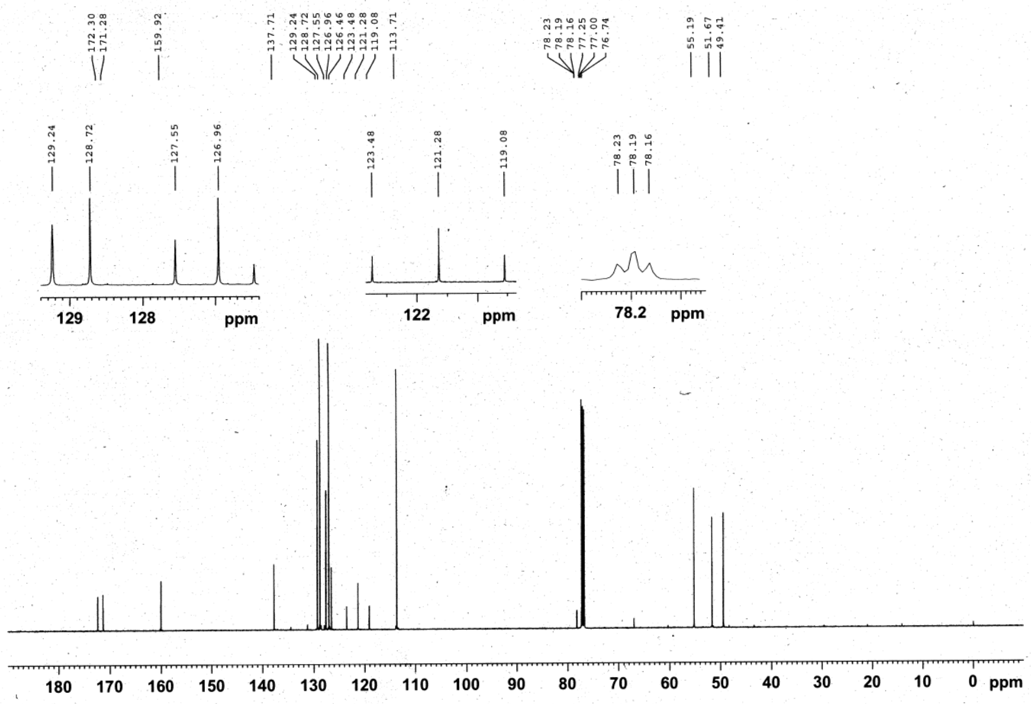

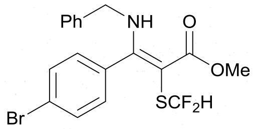

4l

^{19}F NMR (282 MHz, $CDCl_3$)
^{1}H NMR (300 MHz, $CDCl_3$)
^{13}C NMR (125 MHz, $CDCl_3$)

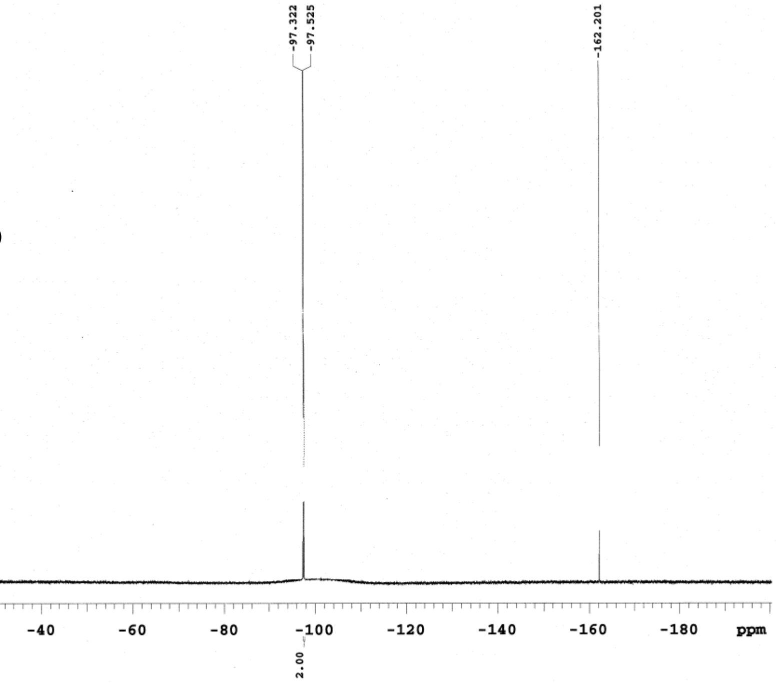

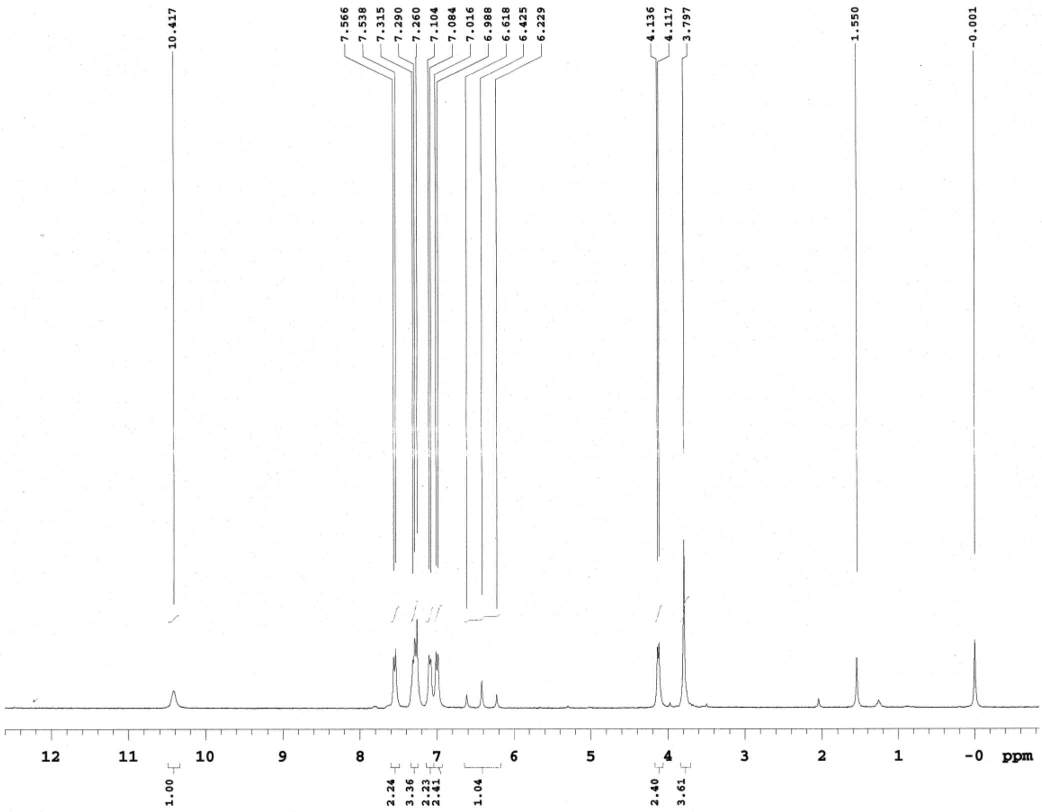
10.417
7.566
7.538
7.315
7.290
7.260
7.104
7.084
7.016
6.988
6.618
6.425
6.229
4.136
4.117
3.797
1.550
-0.001
12 11 10 9 8 7 6 5 4 3 2 1 -0 ppm
1.00
2.24
3.36
2.23
2.41
1.04
2.40
3.61

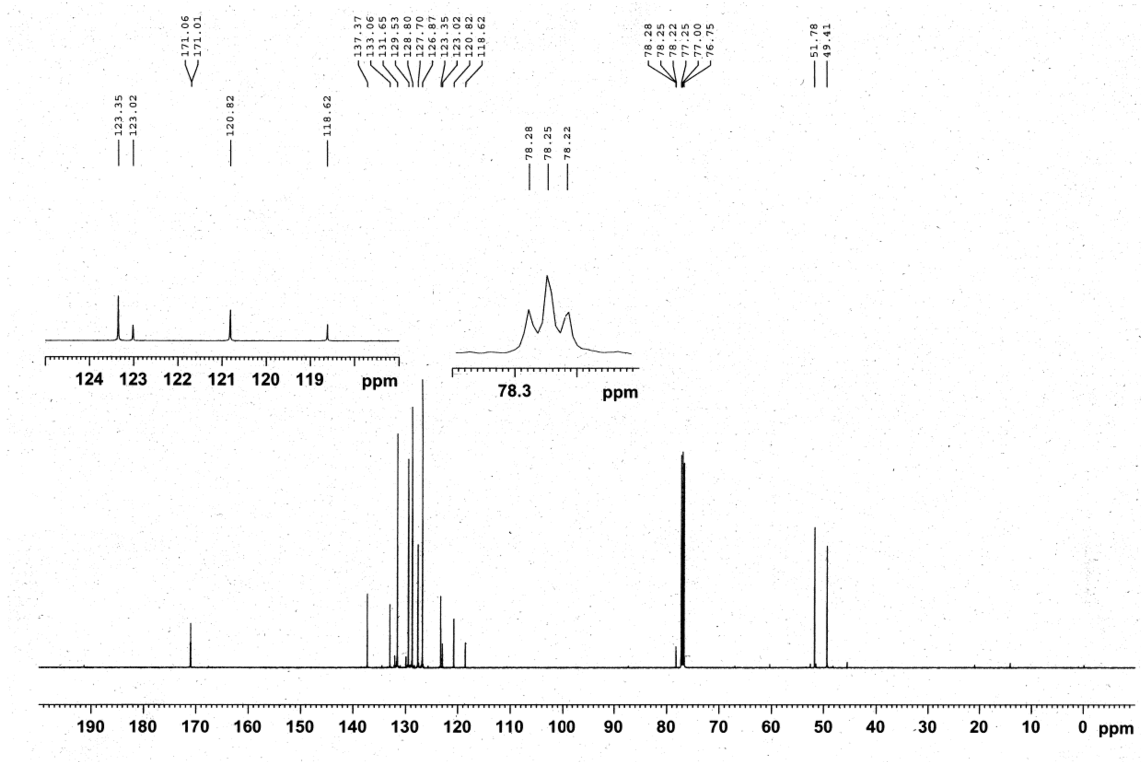
171.06
171.01
137.37
133.06
131.65
129.53
128.80
127.70
126.87
123.35
123.02
120.82
118.62
78.28
78.25
78.22
77.25
77.00
76.75
51.78
49.41
123.35
123.02
120.82
118.62
124 123 122 121 120 119 ppm
78.28
78.25
78.22
78.3 ppm
190 180 170 160 150 140 130 120 110 100 90 80 70 60 50 40 30 20 10 0 ppm

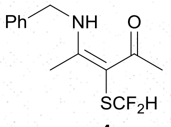

4m

^{19}F NMR (282 MHz, $CDCl_3$)

^{1}H NMR (300 MHz, $CDCl_3$)

^{13}C NMR (125 MHz, $CDCl_3$)

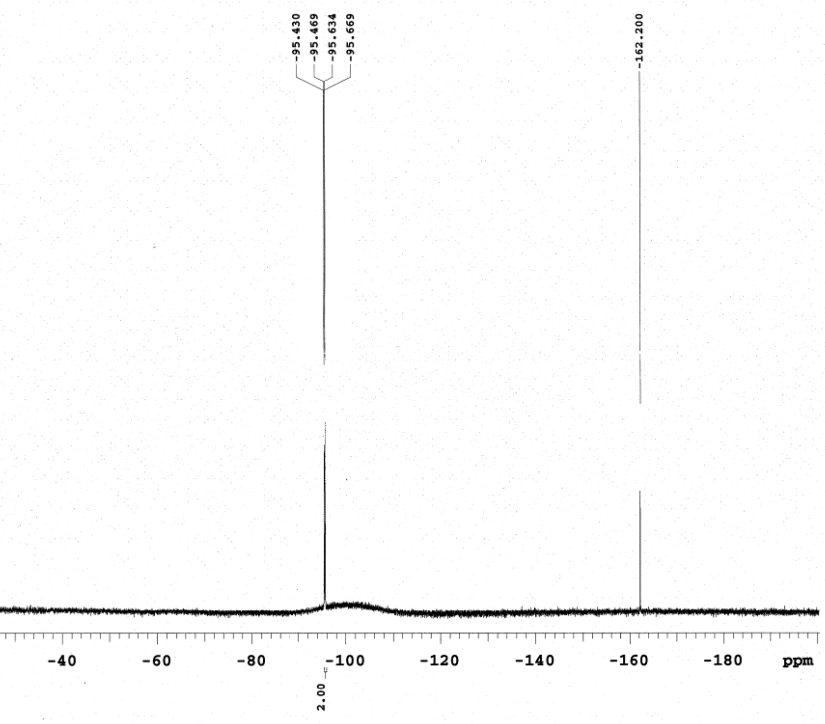

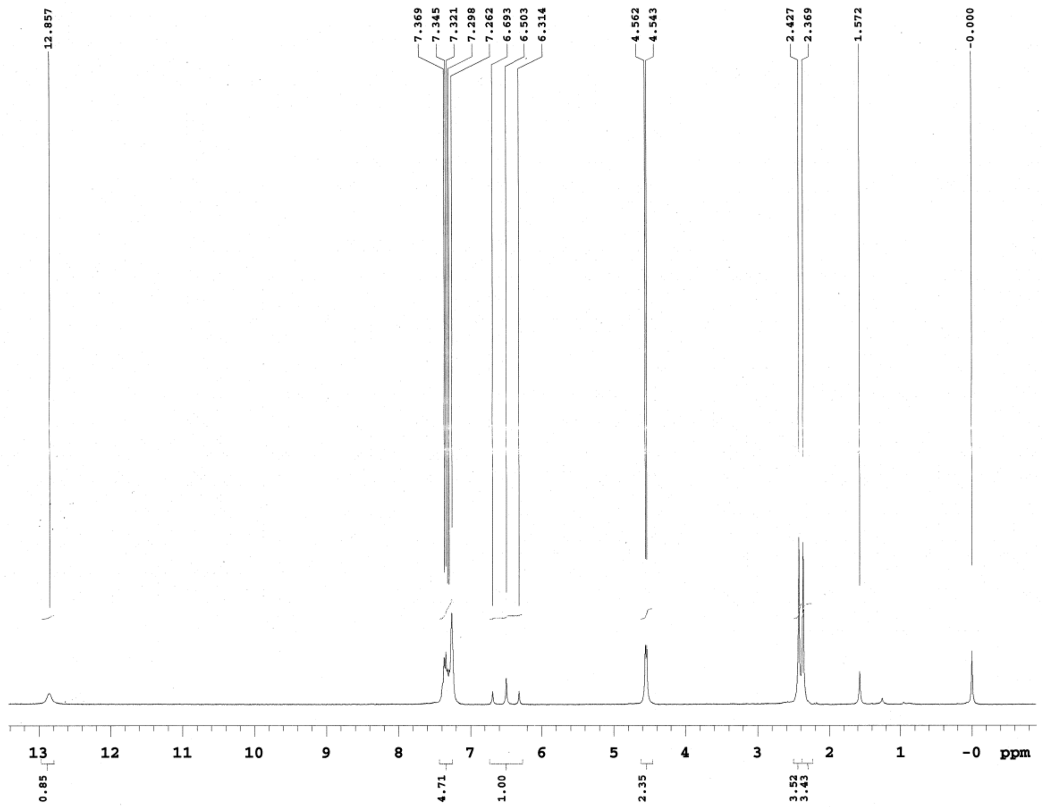

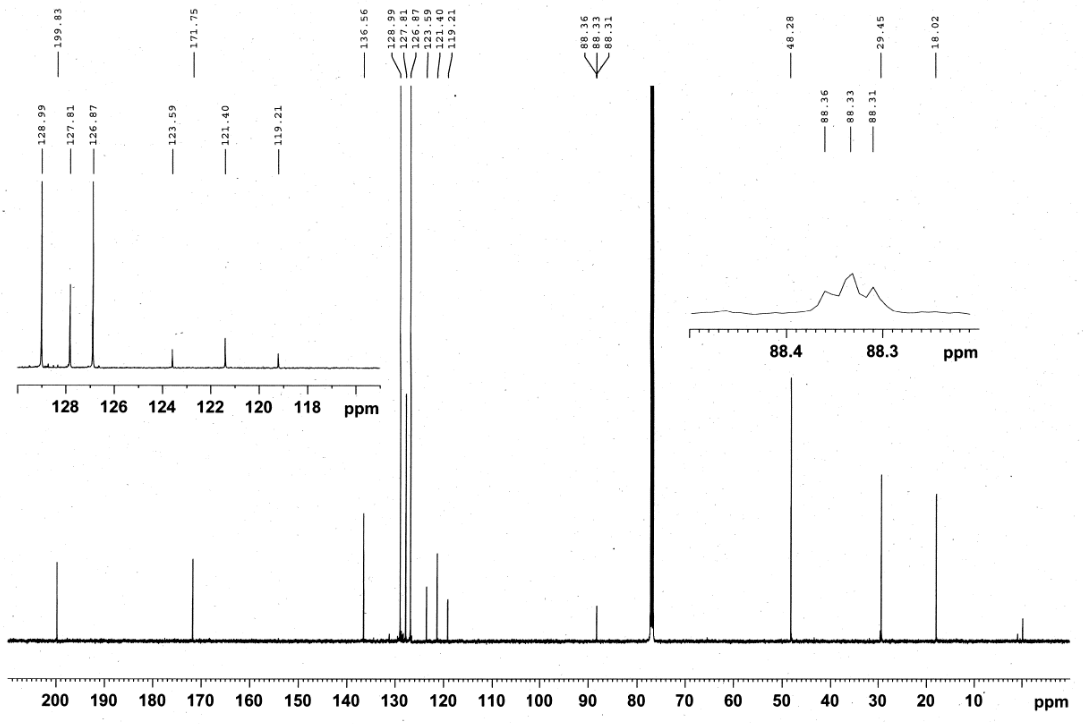

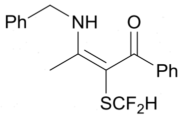

4n

^{19}F NMR (282 MHz, $CDCl_3$)
^{1}H NMR (300 MHz, $CDCl_3$)
^{13}C NMR (125 MHz, $CDCl_3$)

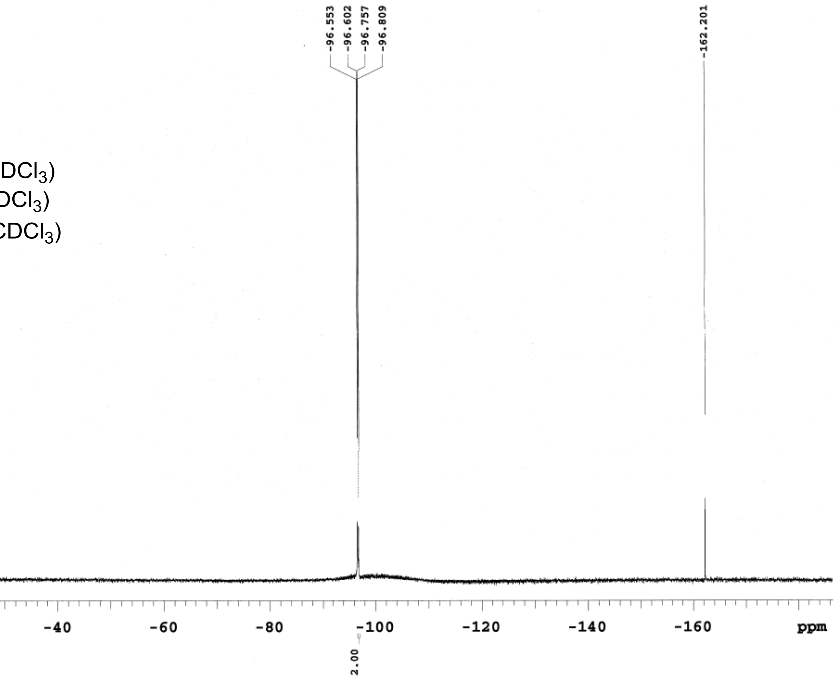

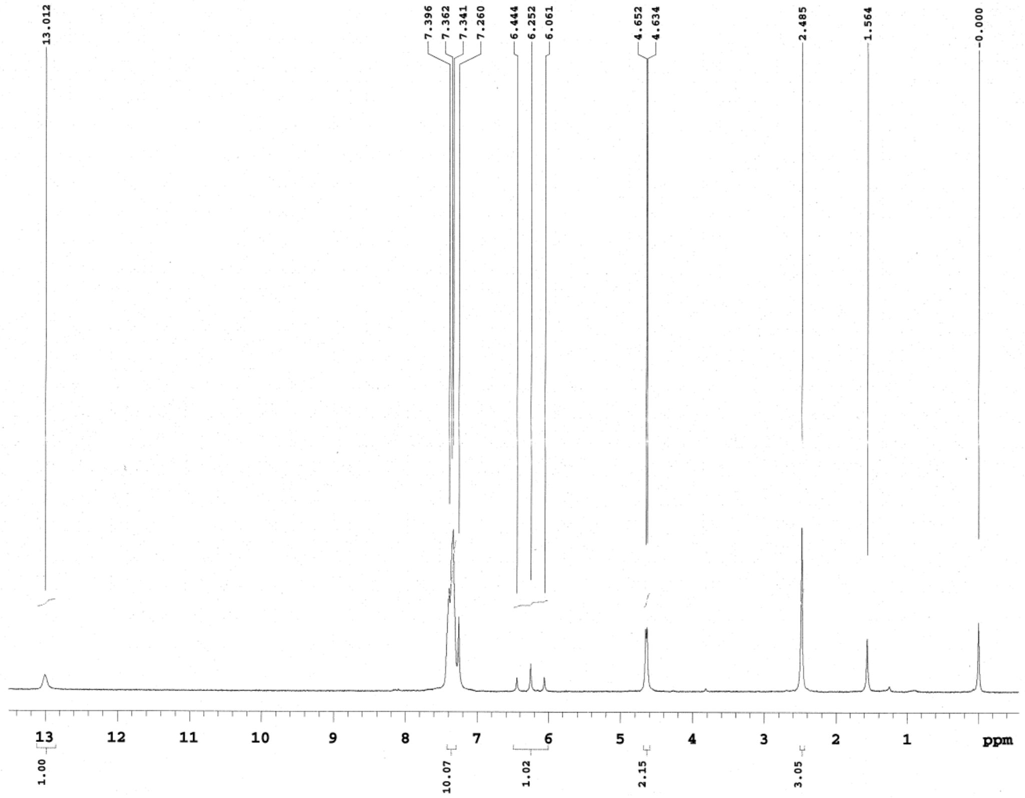
13.012
7.396
7.362
7.341
7.260
6.444
6.252
6.061
4.652
4.634
2.485
1.564
-0.000
ppm
1.00
10.07
1.02
2.15
3.05

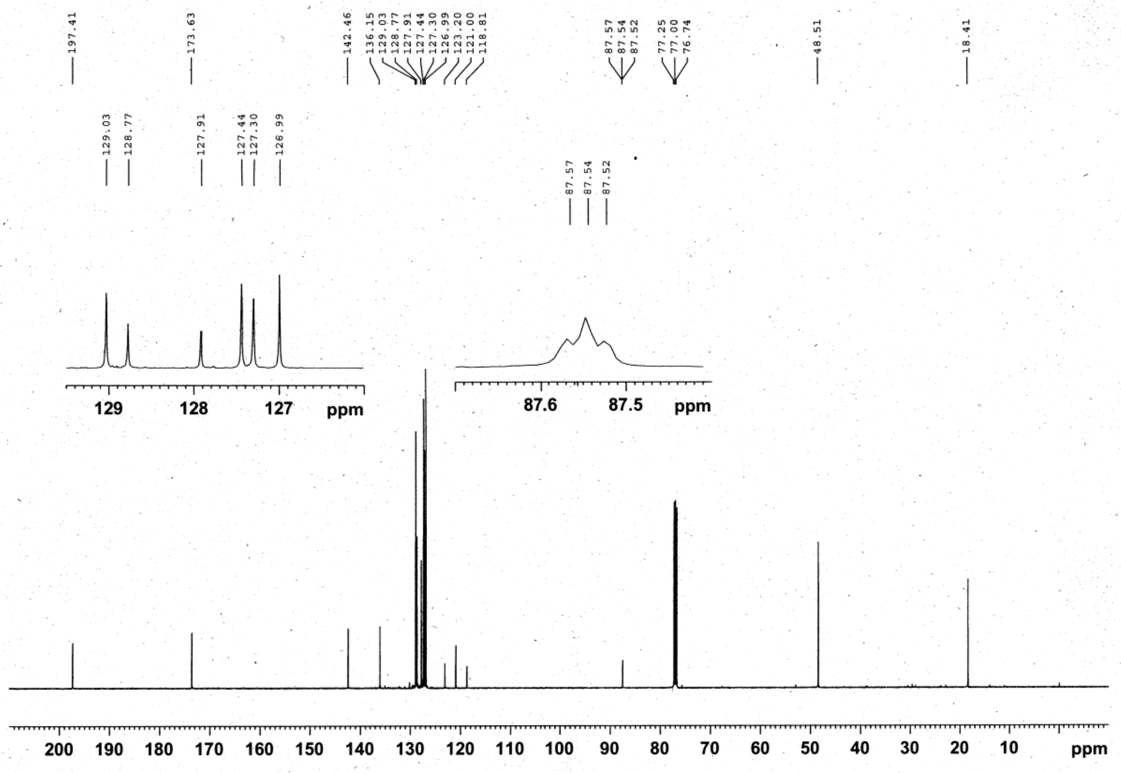
197.41
173.63
142.46
136.15
129.03
128.77
127.91
127.44
127.30
126.99
123.20
121.00
118.81
87.57
87.54
87.52
77.25
77.00
76.74
48.51
18.41
ppm

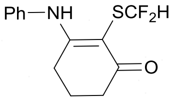

4o

^{19}F NMR (282 MHz, $CDCl_3$)
^{1}H NMR (300 MHz, $CDCl_3$)
^{13}C NMR (125 MHz, $CDCl_3$)

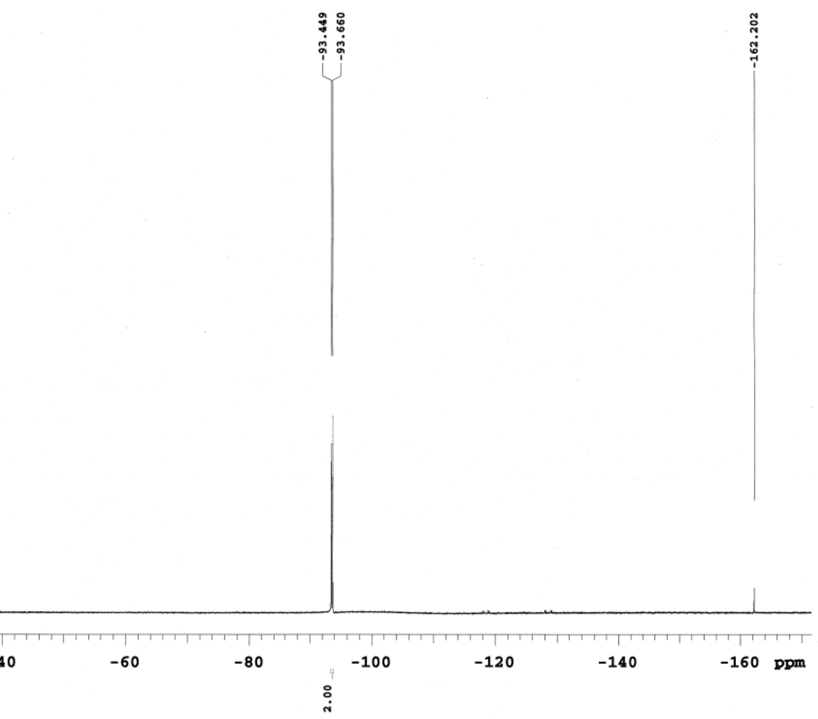

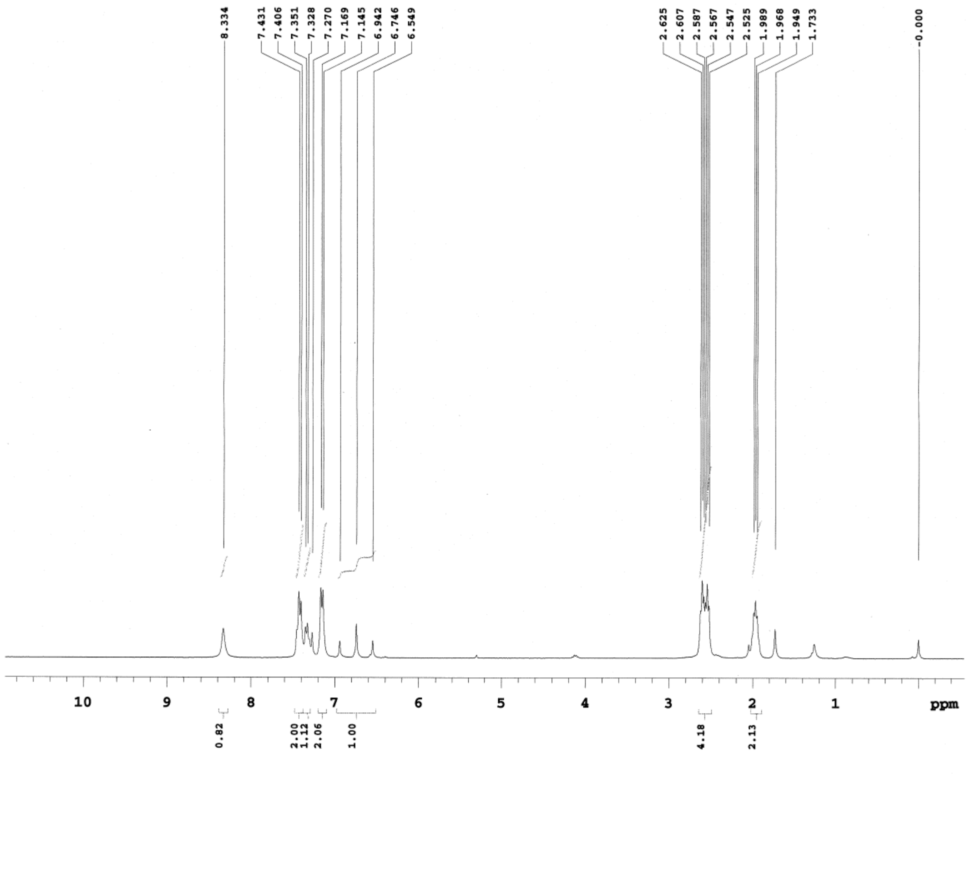

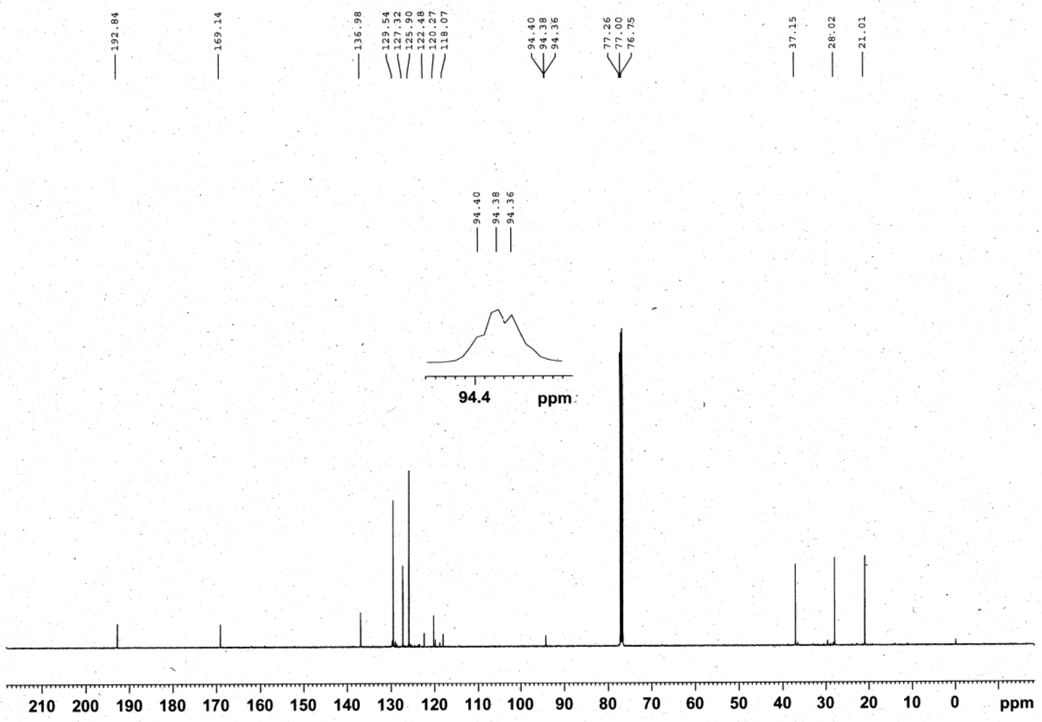

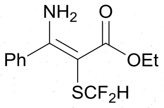

4p

^{19}F NMR (282 MHz, $CDCl_3$)

^{1}H NMR (300 MHz, $CDCl_3$)

^{13}C NMR (125 MHz, $CDCl_3$)

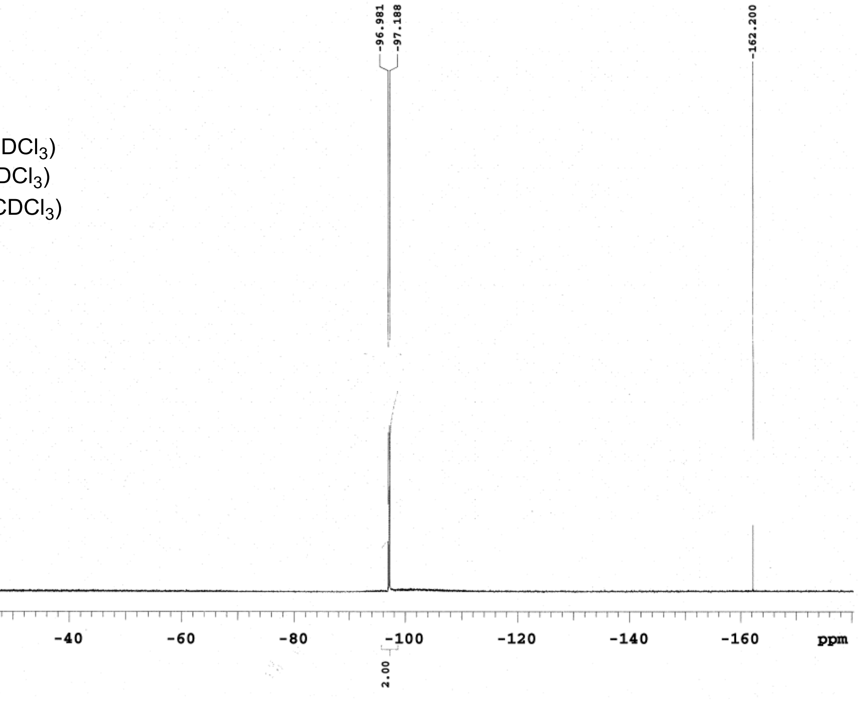

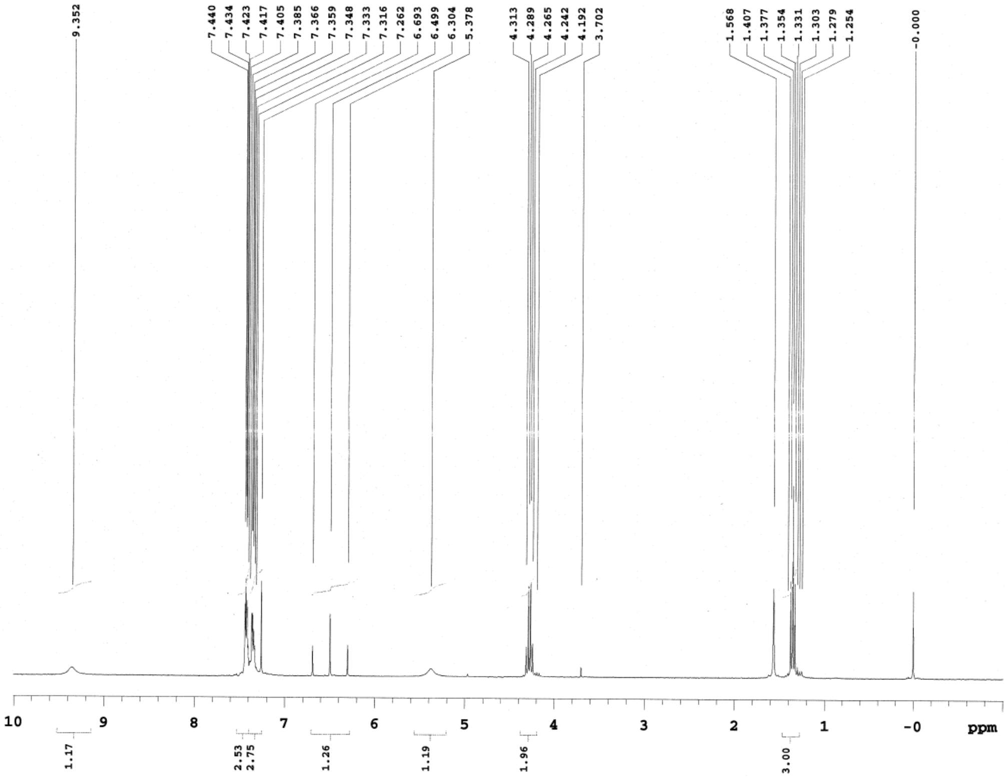
9.352
7.440
7.434
7.423
7.417
7.405
7.385
7.366
7.359
7.348
7.333
7.316
7.262
6.693
6.499
6.304
5.378
4.313
4.289
4.265
4.242
4.192
3.702
1.568
1.407
1.377
1.354
1.331
1.303
1.279
1.254
-0.000
10 9 8 7 6 5 4 3 2 1 -0 ppm
1.17
2.53
2.75
1.26
1.19
1.96
3.00

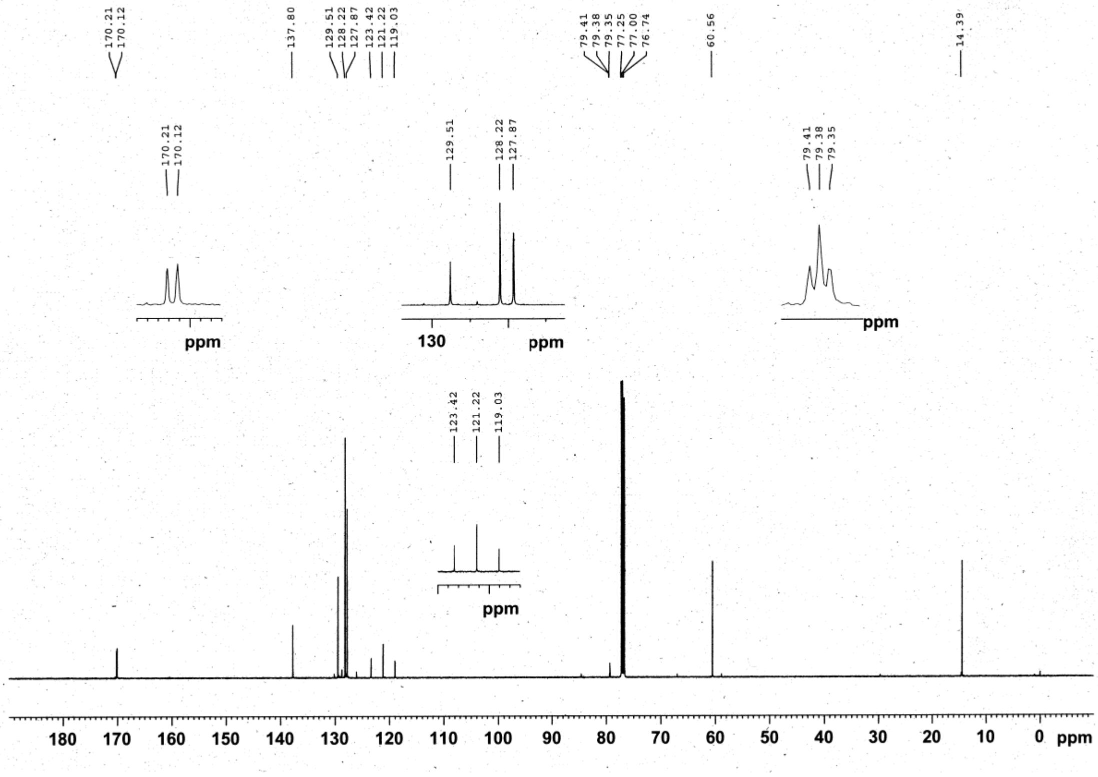
170.21
170.12
137.80
129.51
128.22
127.87
123.42
121.22
119.03
79.41
79.38
79.35
77.25
77.00
76.74
60.56
14.39
ppm
130
180 170 160 150 140 130 120 110 100 90 80 70 60 50 40 30 20 10 0 ppm

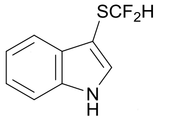

6a

^{19}F NMR (282 MHz, $CDCl_3$)

^{1}H NMR (300 MHz, $CDCl_3$)

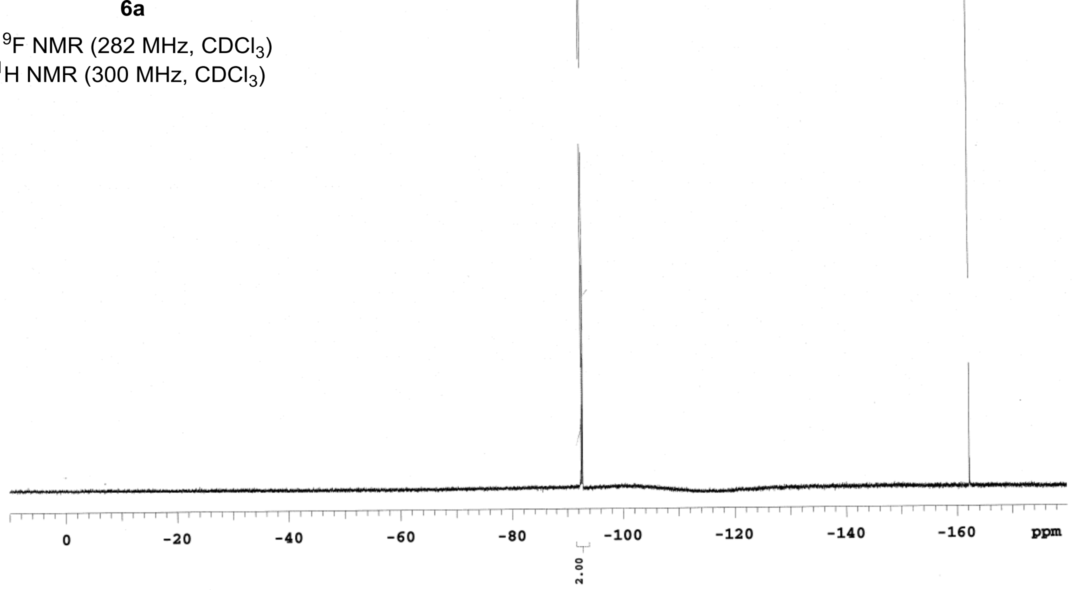

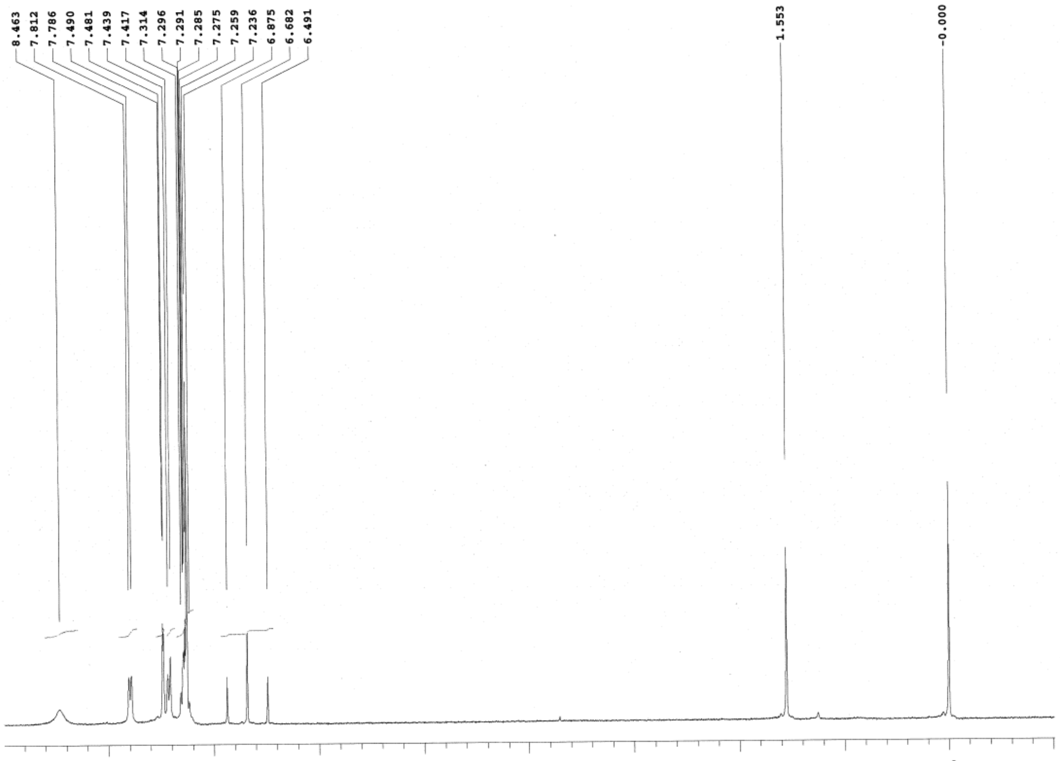

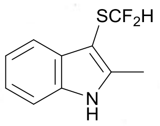

6b

^{19}F NMR (282 MHz, $CDCl_3$)
^{1}H NMR (300 MHz, $CDCl_3$)
^{13}C NMR (125 MHz, $CDCl_3$)

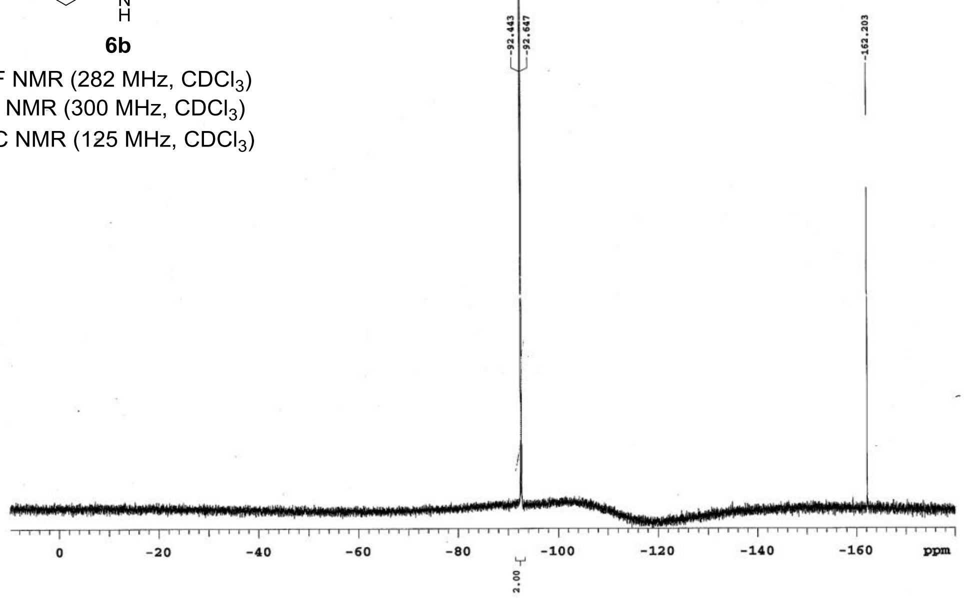

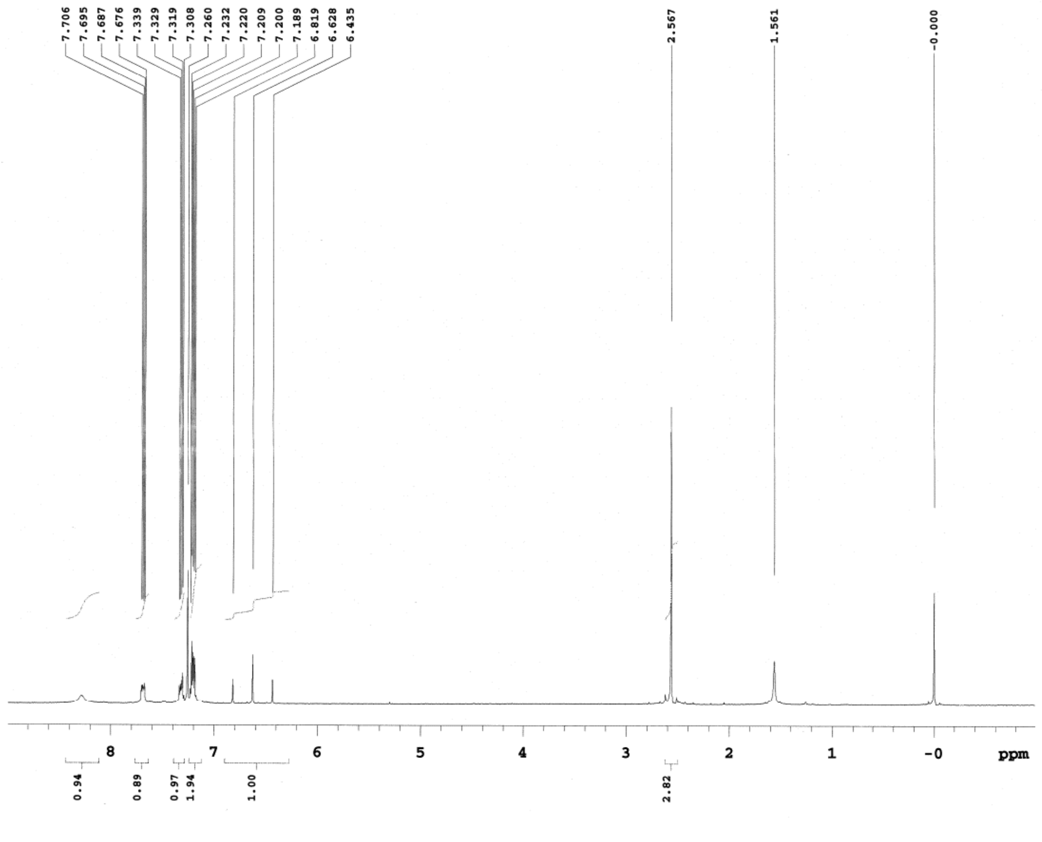

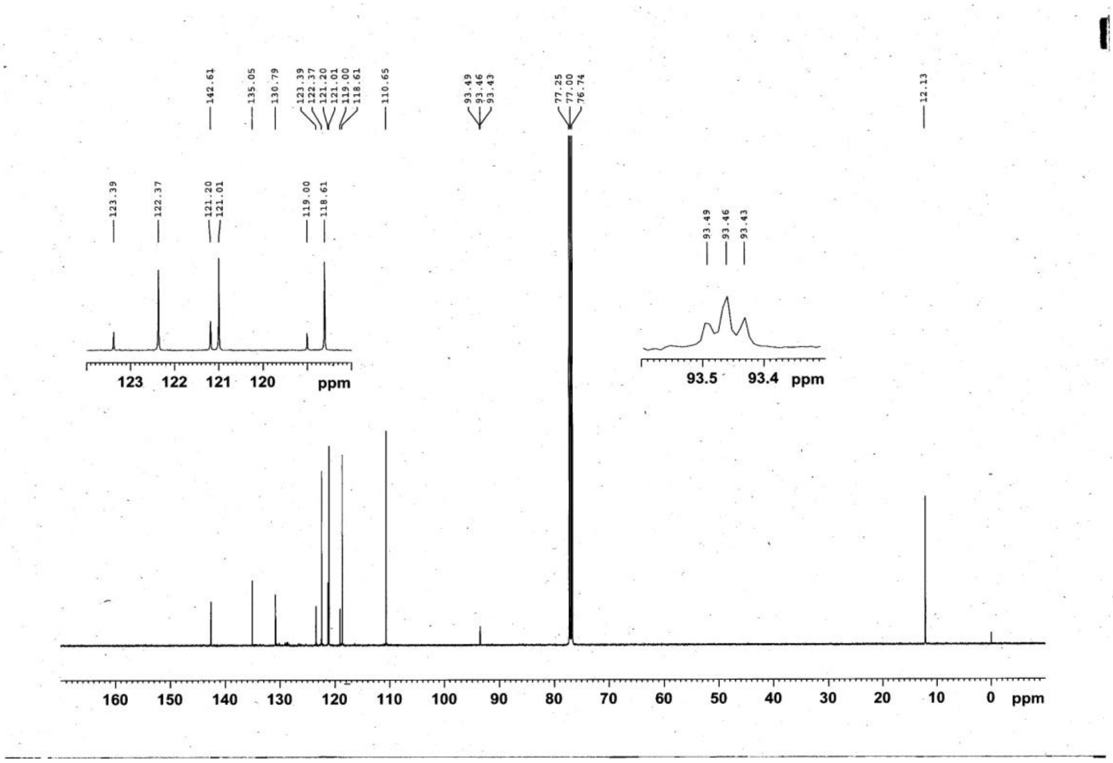

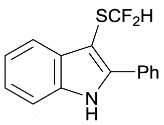

6c

^{19}F NMR (282 MHz, $CDCl_3$)

^{1}H NMR (300 MHz, $CDCl_3$)

^{13}C NMR (125 MHz, $CDCl_3$)

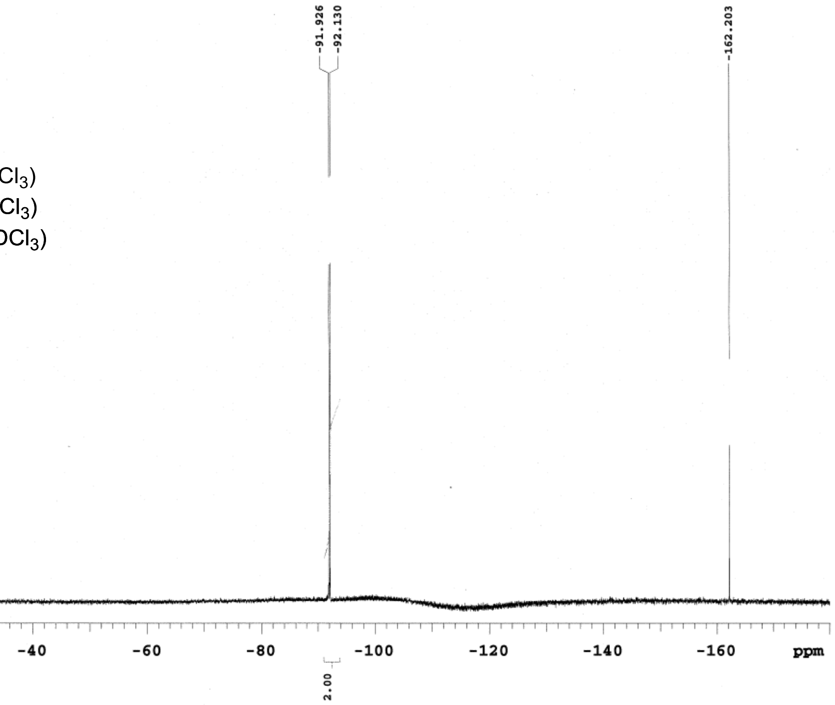

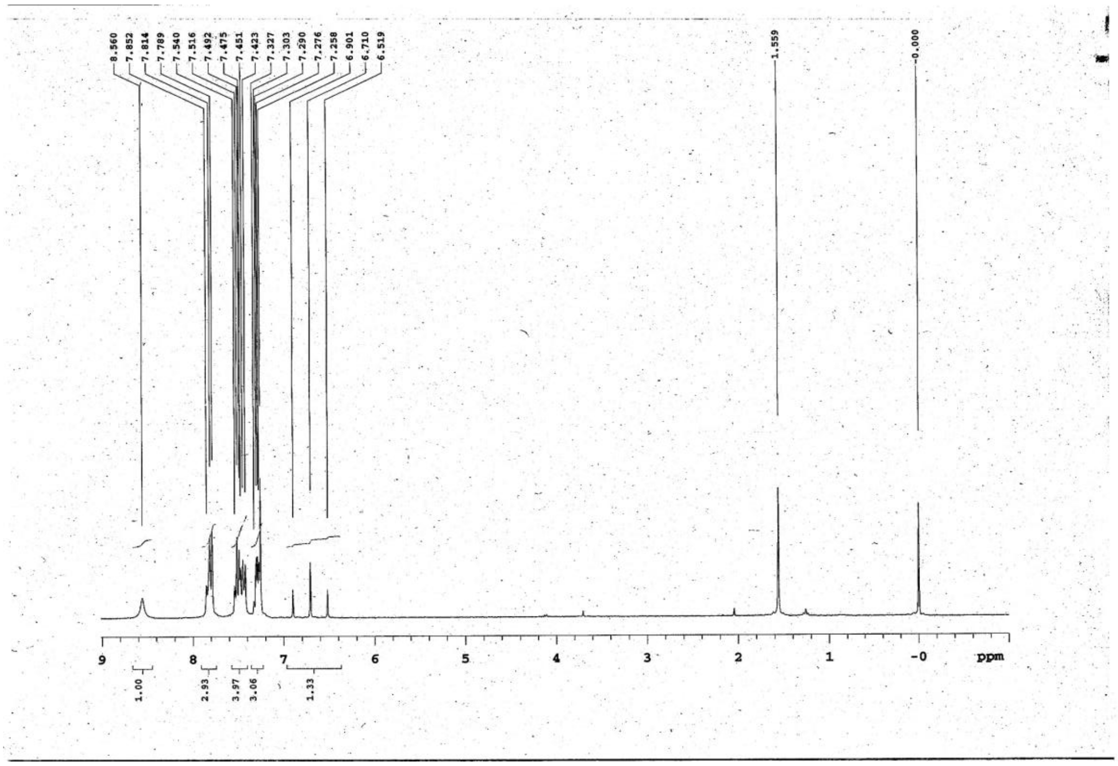
8.560
7.852
7.814
7.789
7.540
7.516
7.492
7.475
7.451
7.423
7.327
7.303
7.290
7.276
7.258
6.901
6.710
6.519
1.559
0.000
9
8
7
6
5
4
3
2
1
-0
ppm
1.00
2.93
3.97
3.06
1.33

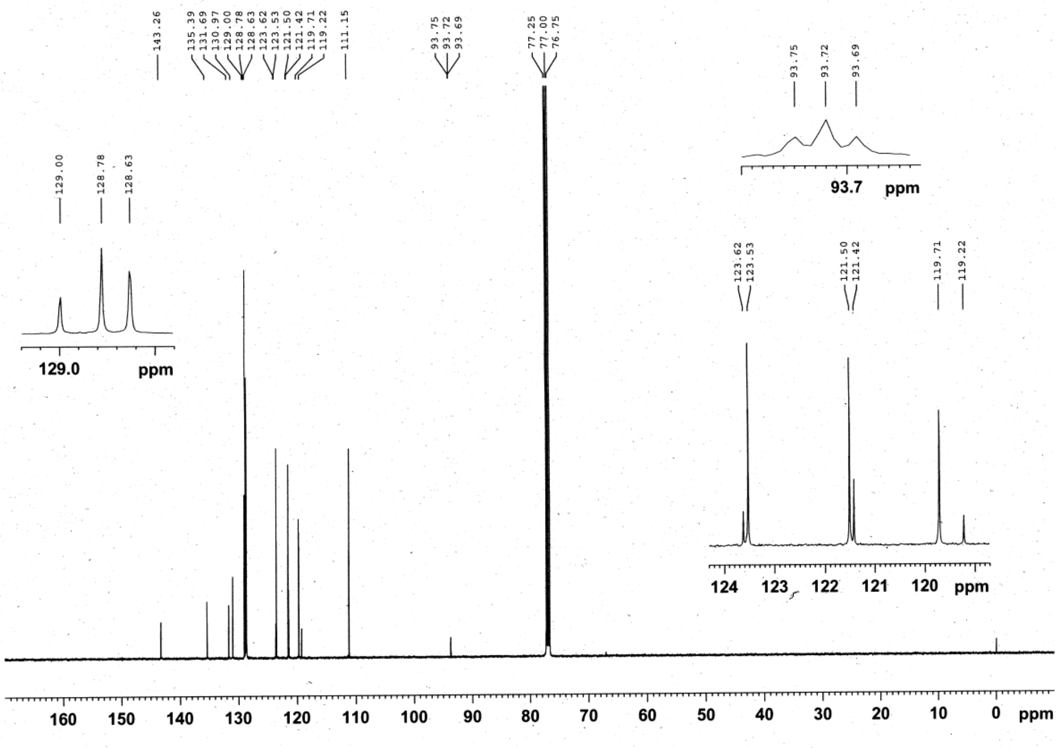
143.26
135.39
131.69
130.97
129.00
128.78
128.63
123.62
123.53
121.50
121.42
119.71
119.22
111.15
93.75
93.72
93.69
77.25
77.00
76.75
129.00
128.78
128.63
129.0
ppm
93.75
93.72
93.69
93.7
ppm
123.62
123.53
121.50
121.42
119.71
119.22
124
123
122
121
120
ppm
160
150
140
130
120
110
100
90
80
70
60
50
40
30
20
10
0
ppm

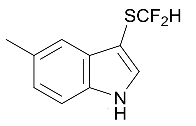

6d

^{19}F NMR (282 MHz, $CDCl_3$)
^{1}H NMR (300 MHz, $CDCl_3$)
^{13}C NMR (125 MHz, $CDCl_3$)

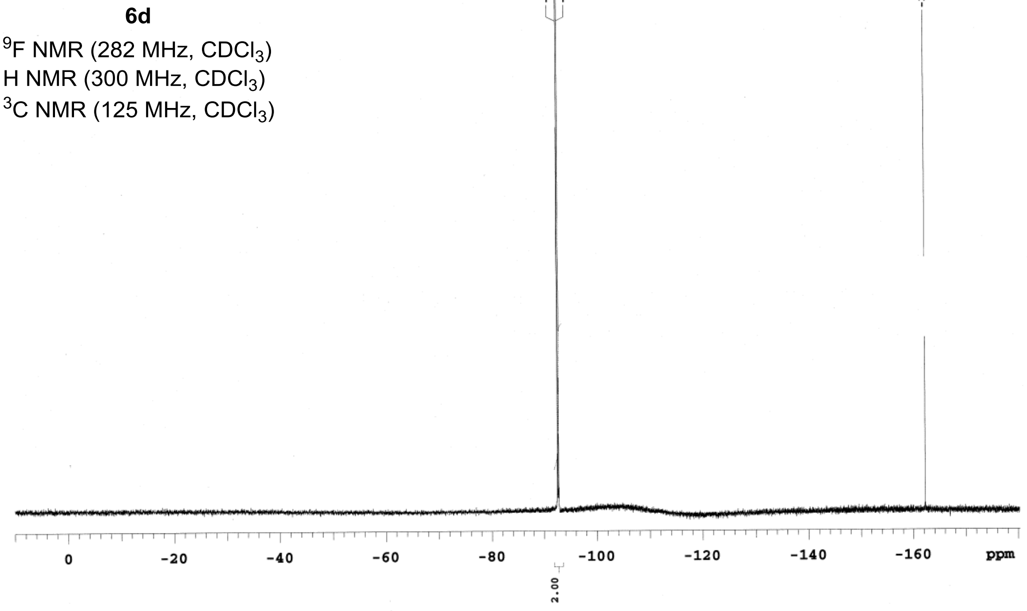

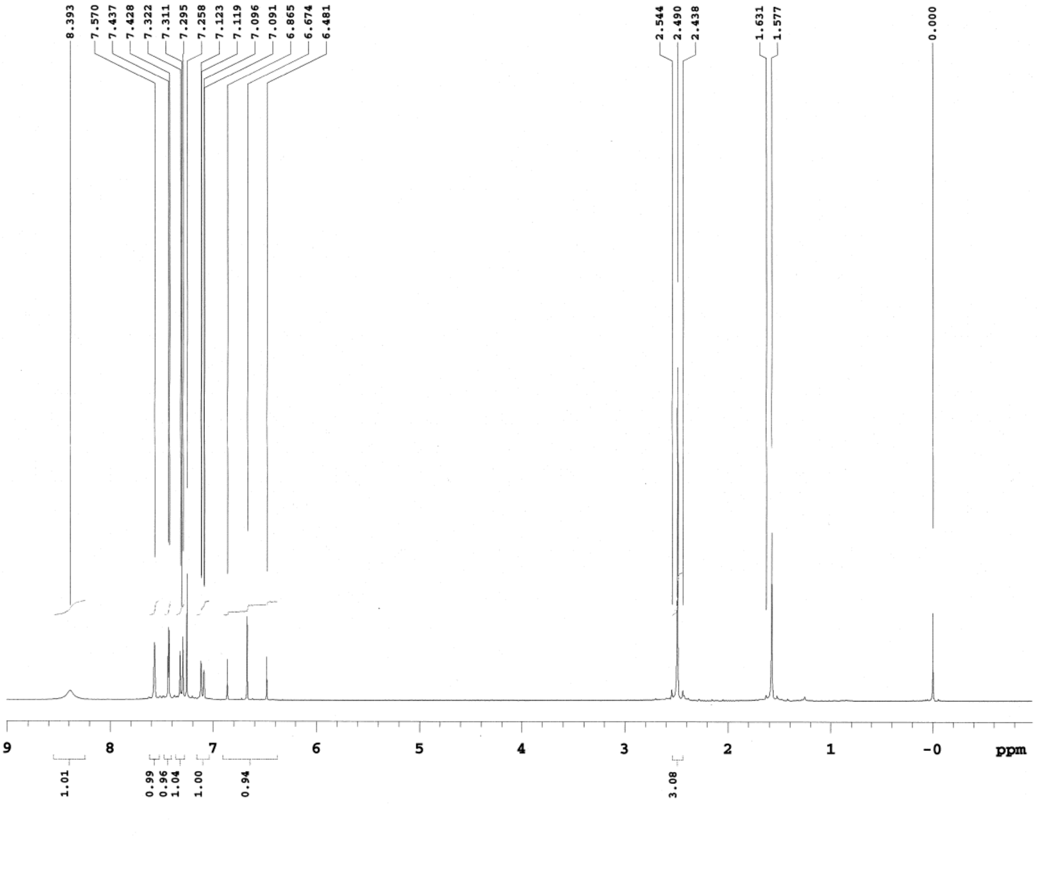

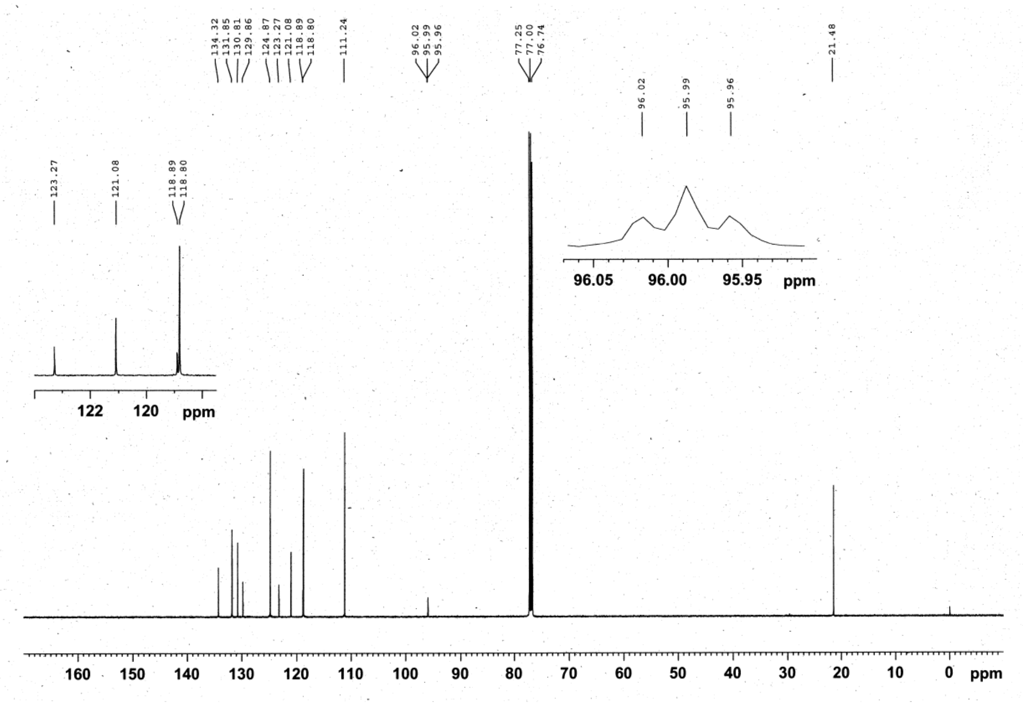

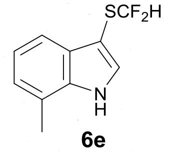

6e

^{19}F NMR (282 MHz, $CDCl_3$)
^{1}H NMR (300 MHz, $CDCl_3$)
^{13}C NMR (125 MHz, $CDCl_3$)

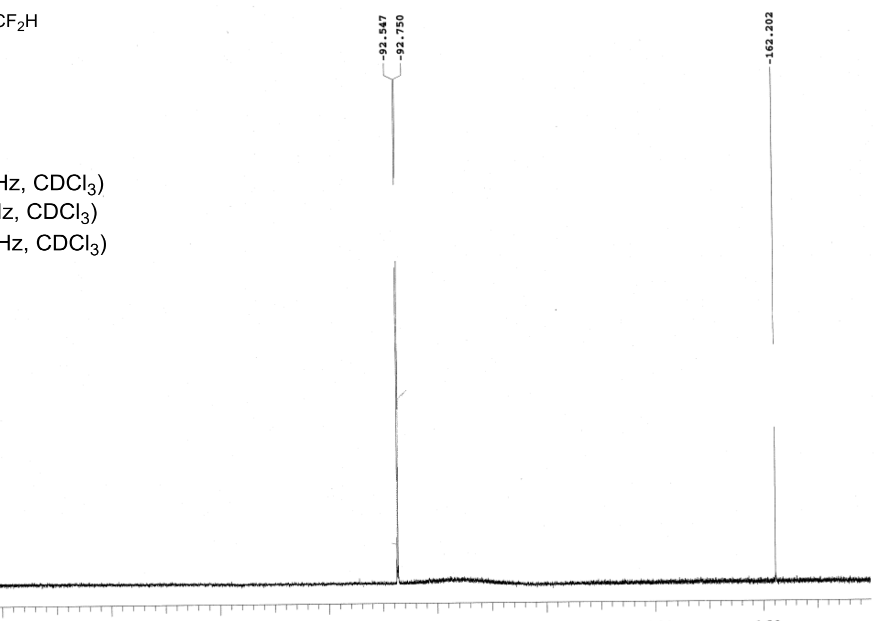

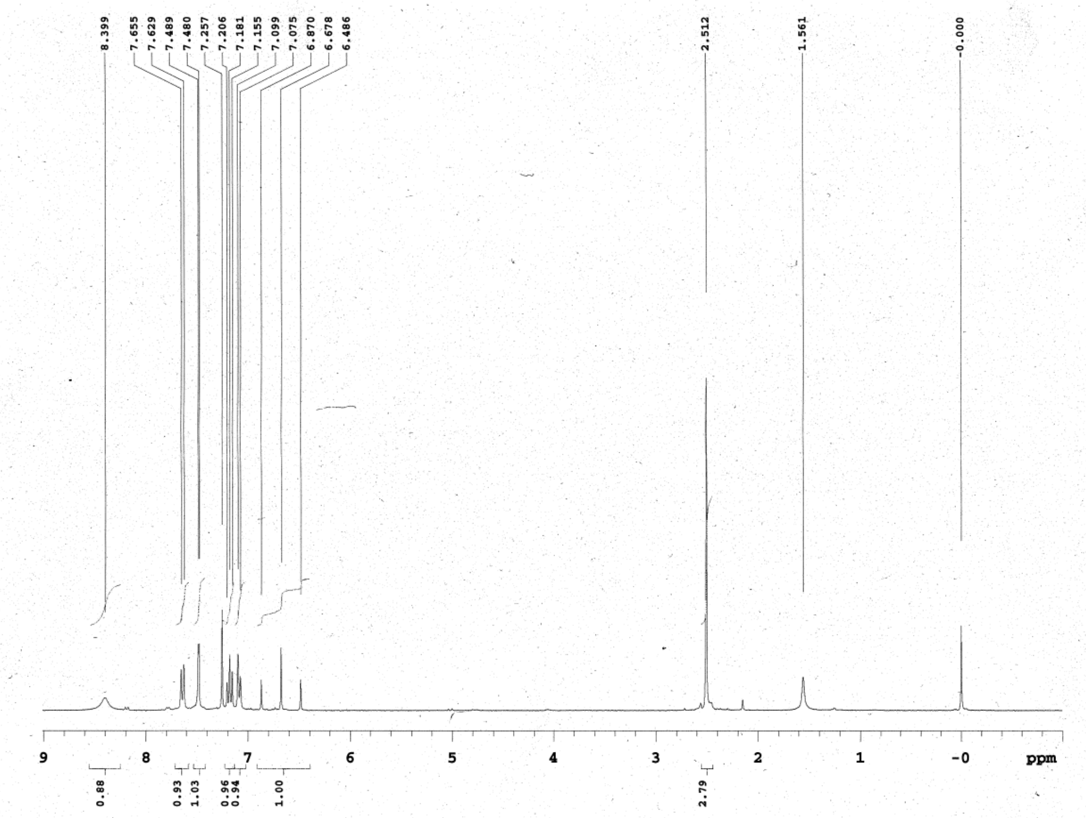
8.399
7.655
7.629
7.489
7.480
7.257
7.206
7.181
7.155
7.099
7.075
6.870
6.678
6.486
2.512
1.561
0.000
0.88
0.93
1.03
0.96
0.94
1.00
2.79
ppm

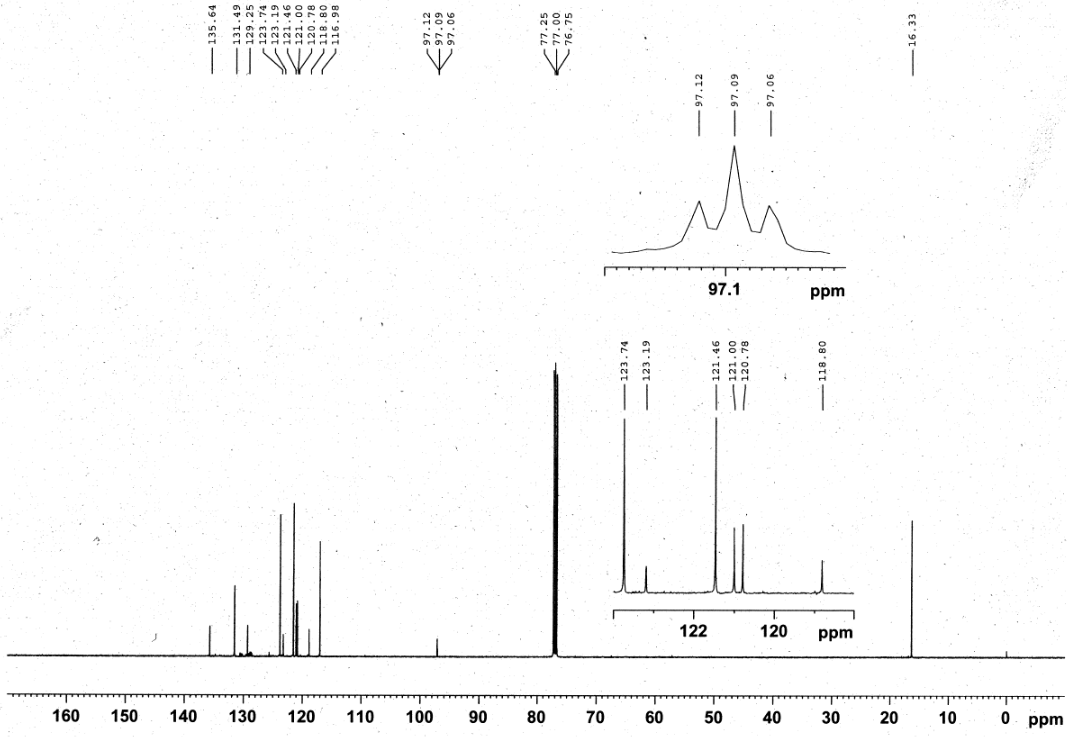
135.64
131.49
129.25
123.74
123.19
121.46
121.00
120.78
118.80
116.98
97.12
97.09
97.06
77.25
77.00
76.75
16.33
97.1
ppm
122
120
ppm
ppm

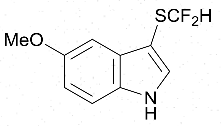

6f

^{19}F NMR (282 MHz, $CDCl_3$)
^{1}H NMR (300 MHz, $CDCl_3$)
^{13}C NMR (125 MHz, $CDCl_3$)

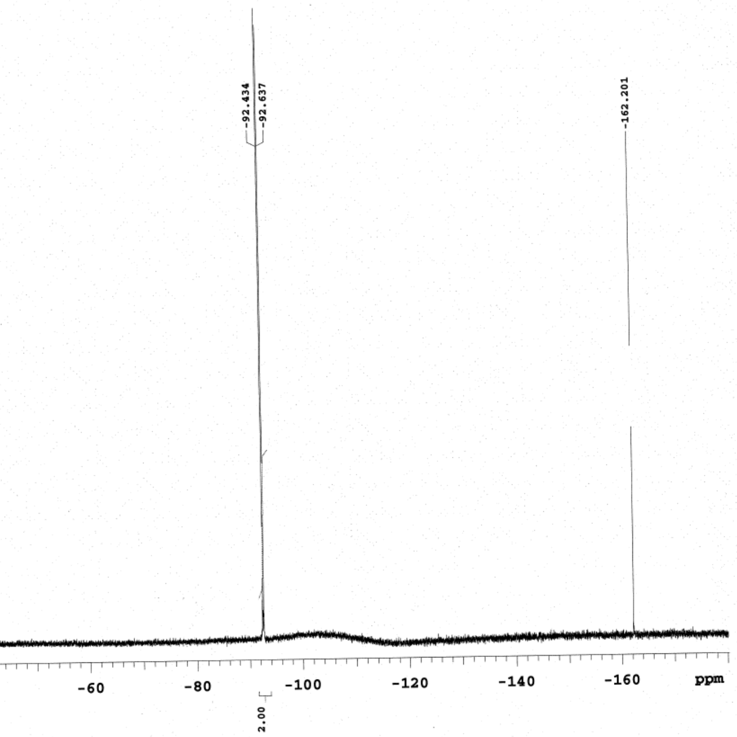

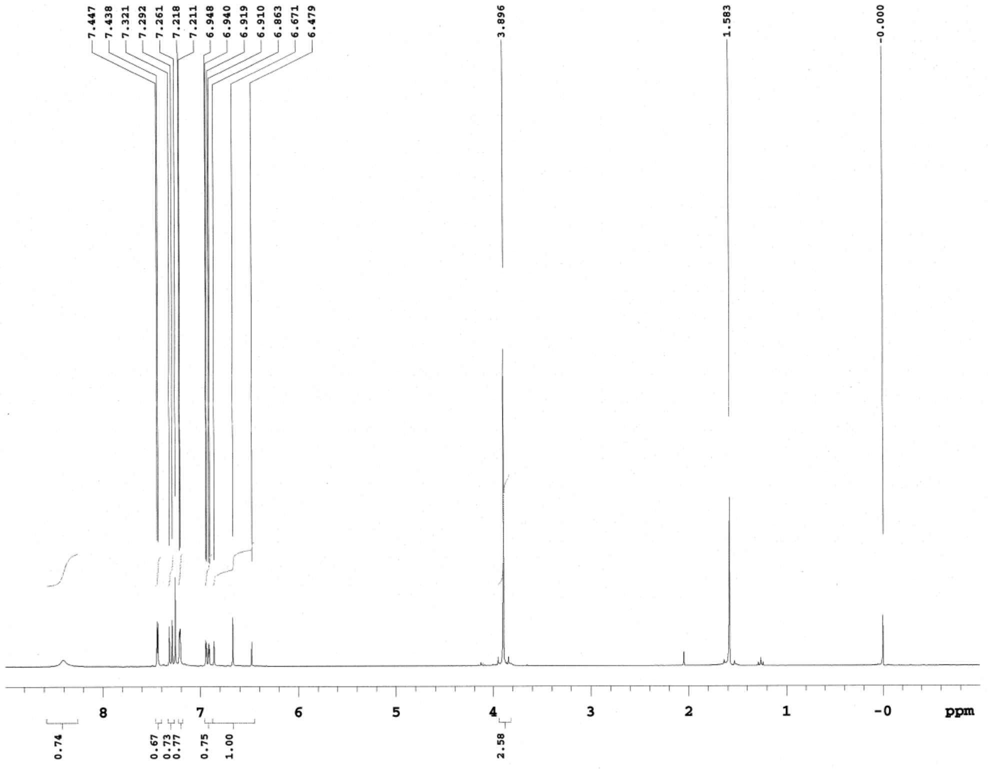

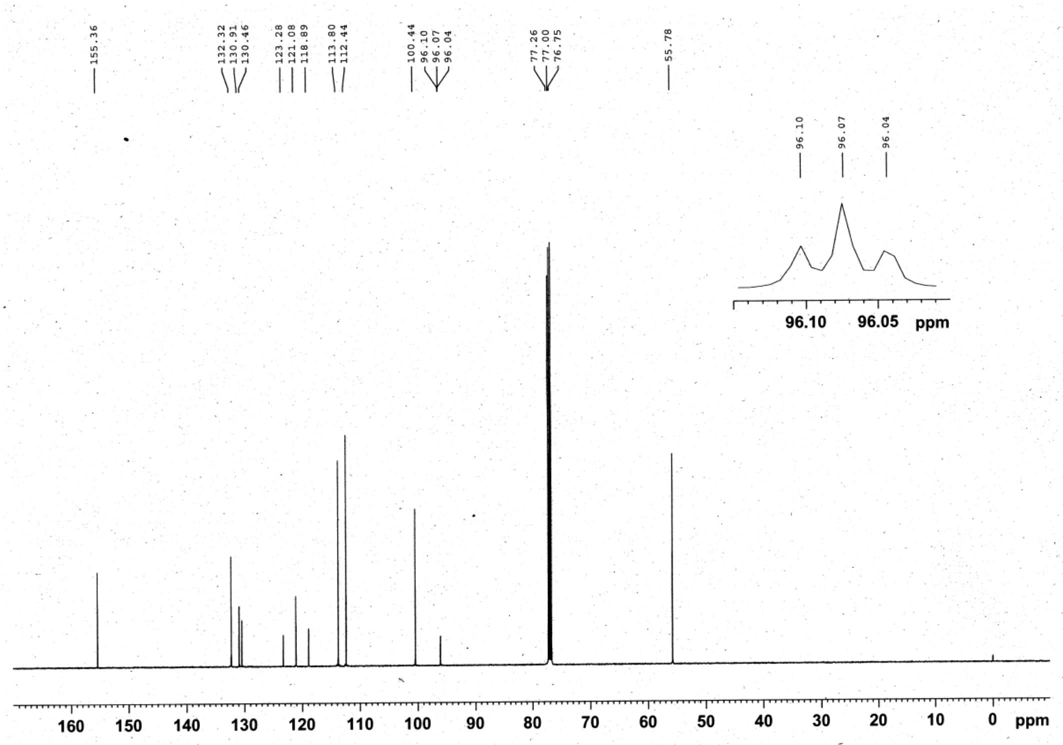

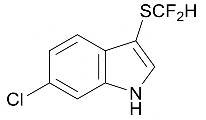

6g

^{19}F NMR (282 MHz, $CDCl_3$)
^{1}H NMR (300 MHz, $CDCl_3$)
^{13}C NMR (125 MHz, $CDCl_3$)

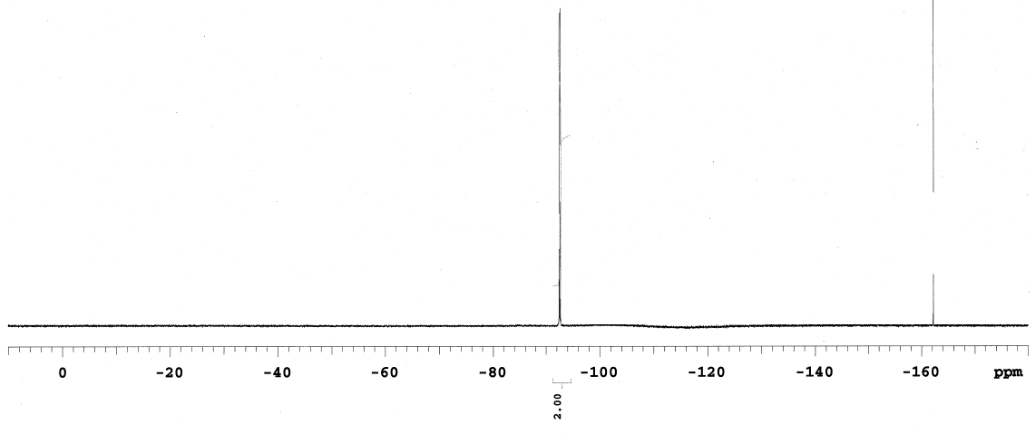

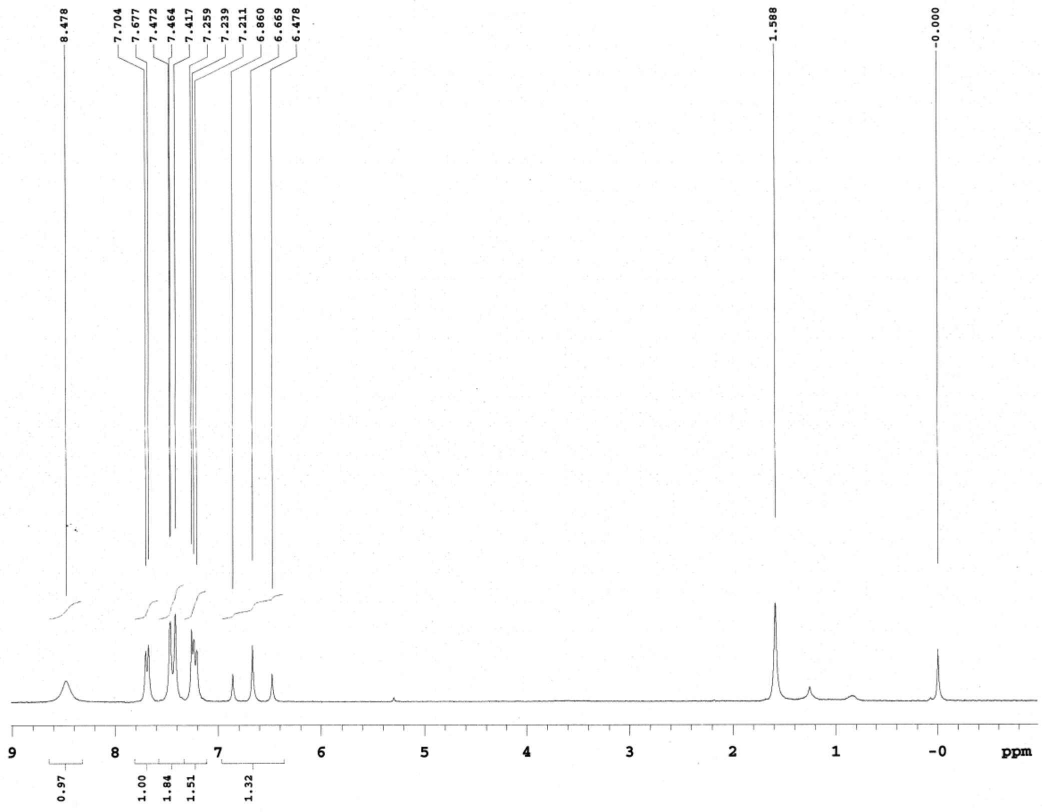

8.478
7.704
7.677
7.472
7.464
7.417
7.259
7.239
7.211
6.860
6.669
6.478
1.588
-0.000
0.97
1.00
1.84
1.51
1.32
ppm

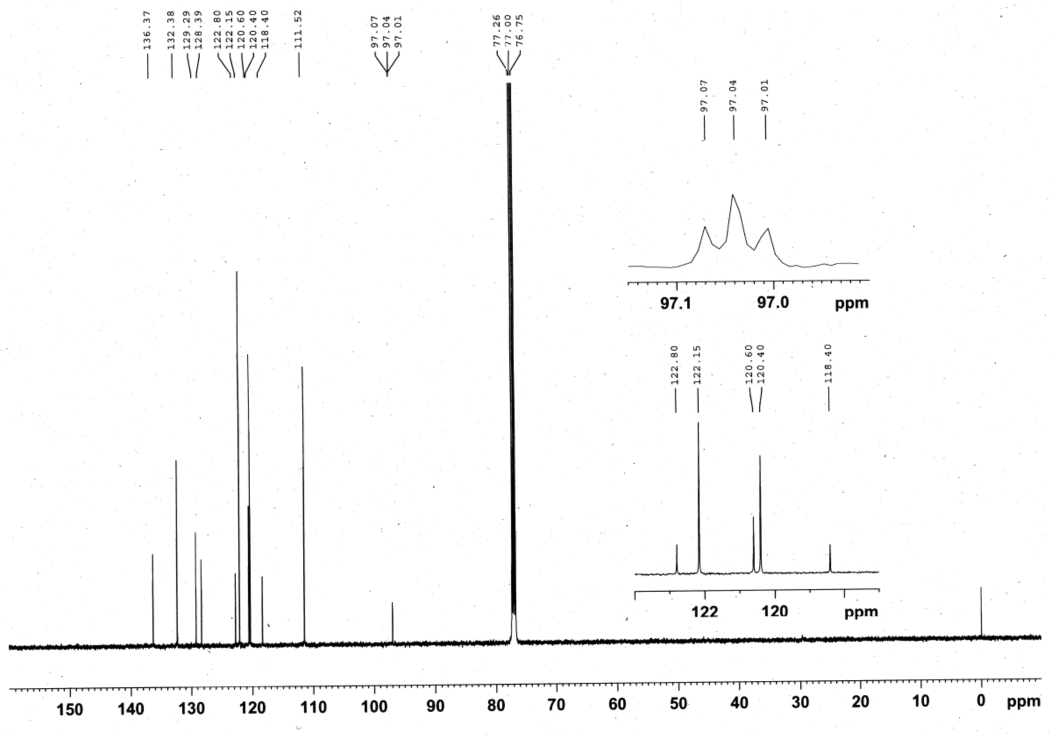

136.37
132.38
129.29
128.39
122.80
122.15
120.60
120.40
118.40
111.52
97.07
97.04
97.01
77.26
77.00
76.75
97.1
97.0
ppm
122
120
ppm

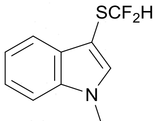

6h

^{19}F NMR (282 MHz, $CDCl_3$)
^{1}H NMR (300 MHz, $CDCl_3$)
^{13}C NMR (125 MHz, $CDCl_3$)

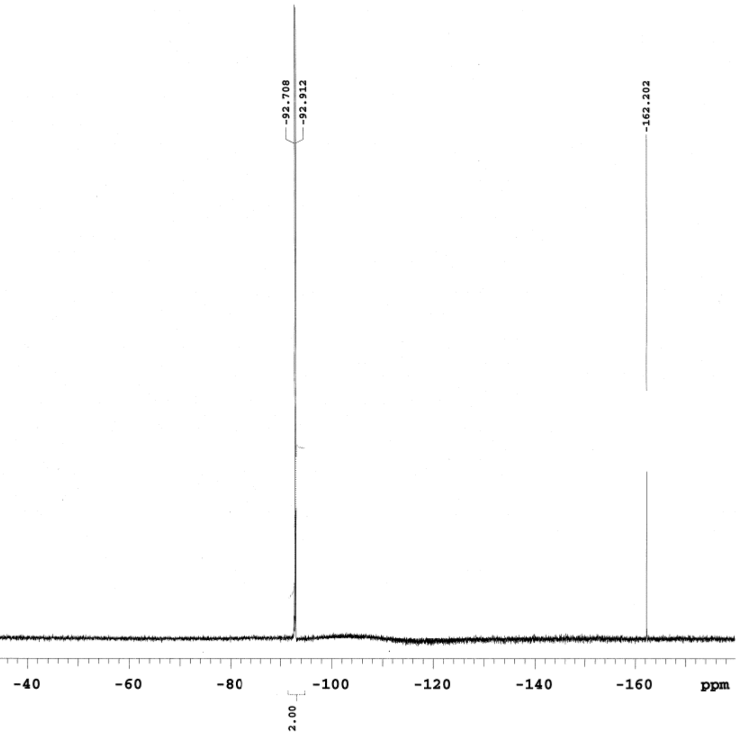

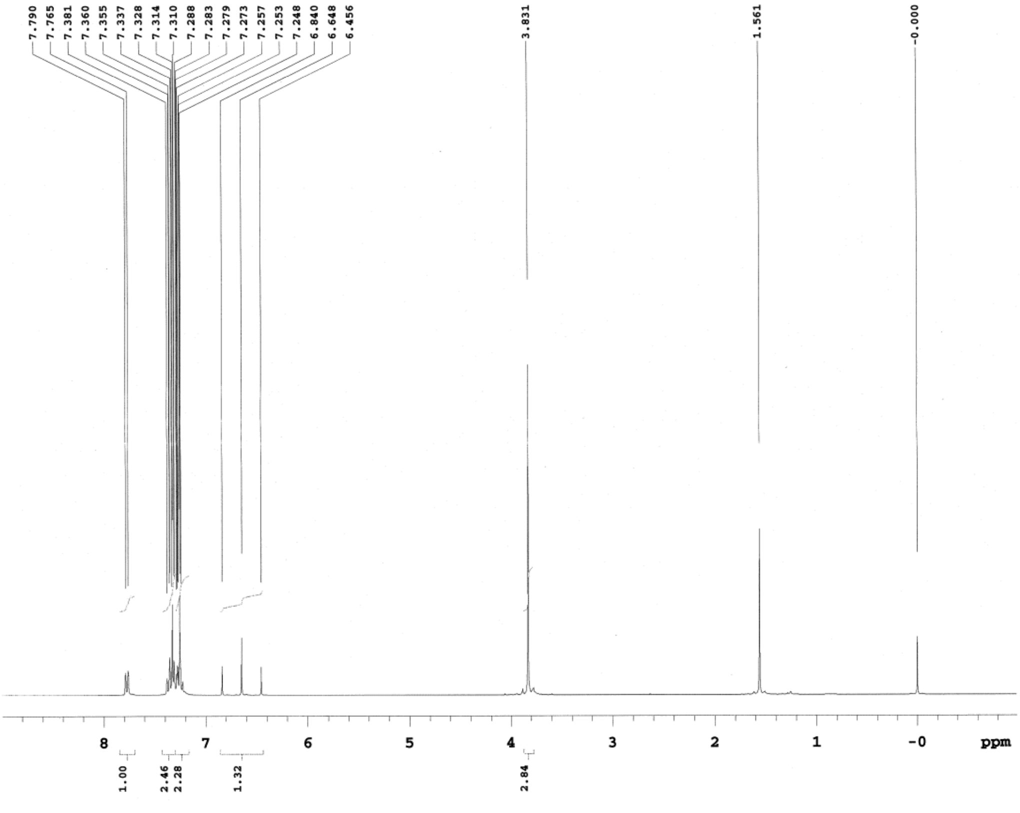

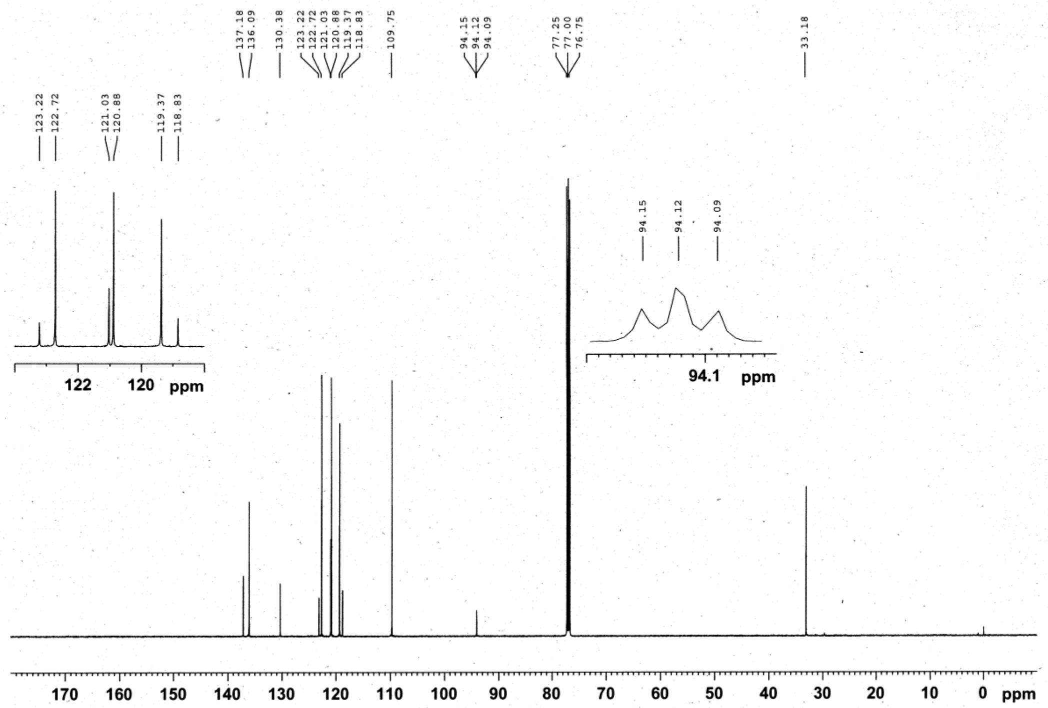
137.18
136.09
130.38
123.22
122.72
121.03
120.88
119.37
118.83
109.75
94.15
94.12
94.09
77.25
77.00
76.75
33.18
122
120
ppm
94.1
ppm
170 160 150 140 130 120 110 100 90 80 70 60 50 40 30 20 10 0 ppm

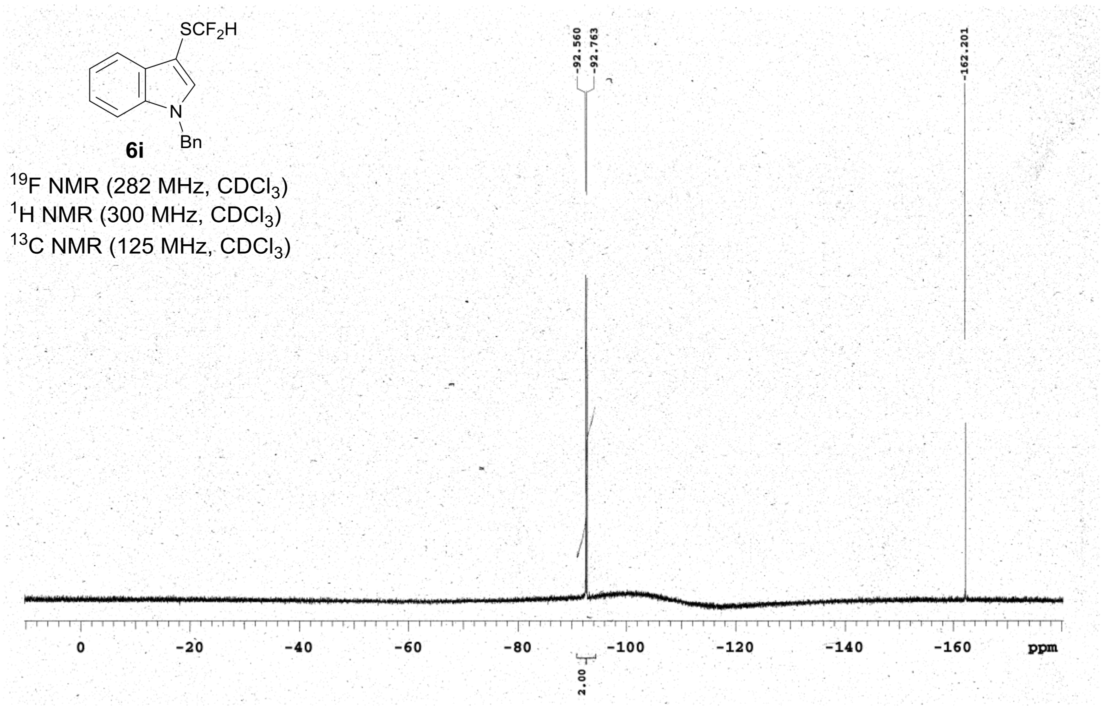
SCF2H
N
Bn
6i
19F NMR (282 MHz, CDCl3)
1H NMR (300 MHz, CDCl3)
13C NMR (125 MHz, CDCl3)
-92.560
-92.763
-162.201
0 -20 -40 -60 -80 -100 -120 -140 -160 ppm
2.00

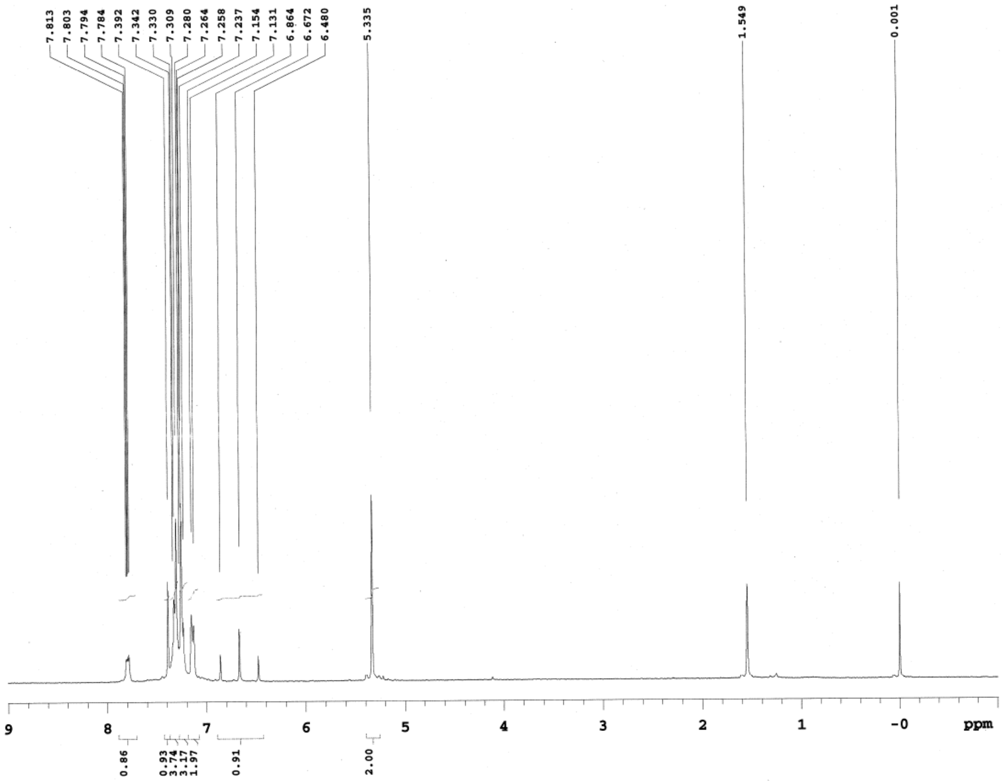
7.813
7.803
7.794
7.784
7.392
7.342
7.330
7.309
7.280
7.264
7.258
7.237
7.154
7.131
6.864
6.672
6.480
5.335
1.549
0.001
0.86
0.93
3.74
3.17
1.97
0.91
2.00
ppm

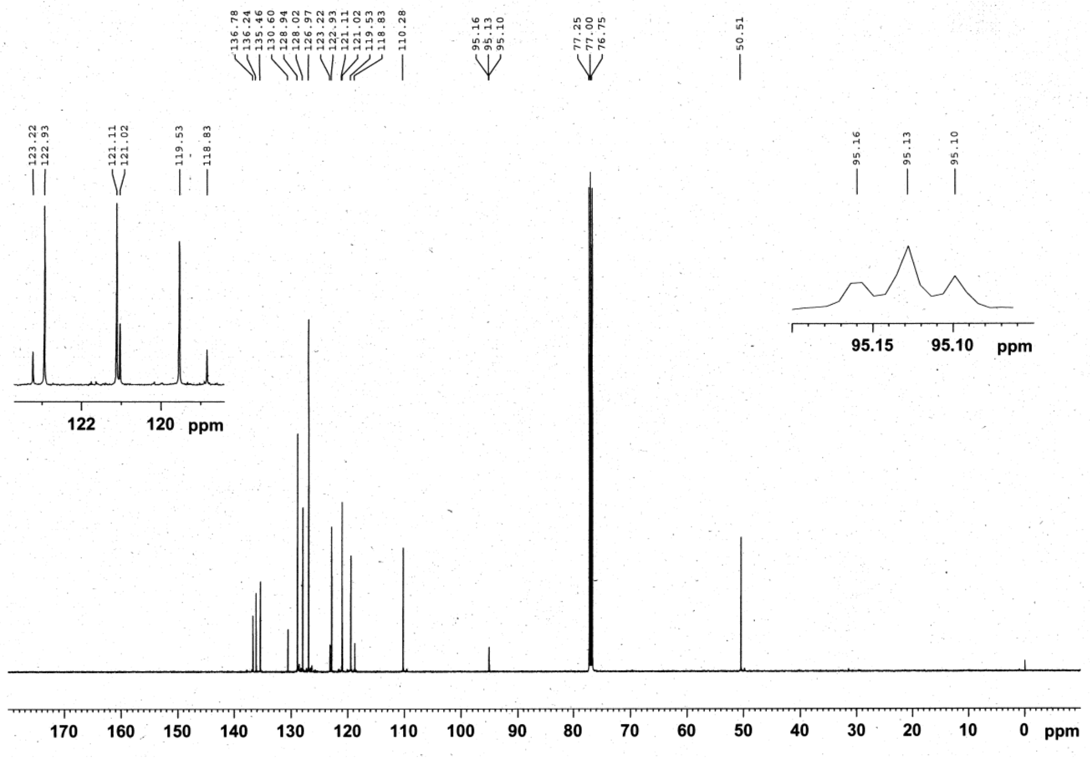
136.78
136.24
135.46
130.60
128.94
128.02
126.97
123.22
122.93
121.11
121.02
119.53
118.83
110.28
95.16
95.13
95.10
77.25
77.00
76.75
50.51
ppm

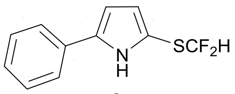

8a

^{19}F NMR (282 MHz, $CDCl_3$)
^{1}H NMR (300 MHz, $CDCl_3$)
^{13}C NMR (125 MHz, $CDCl_3$)

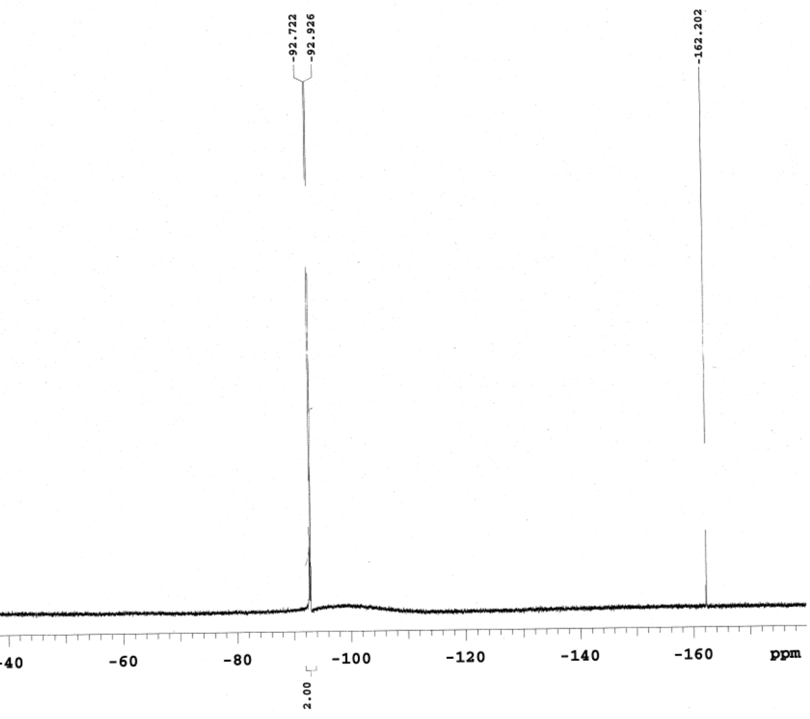

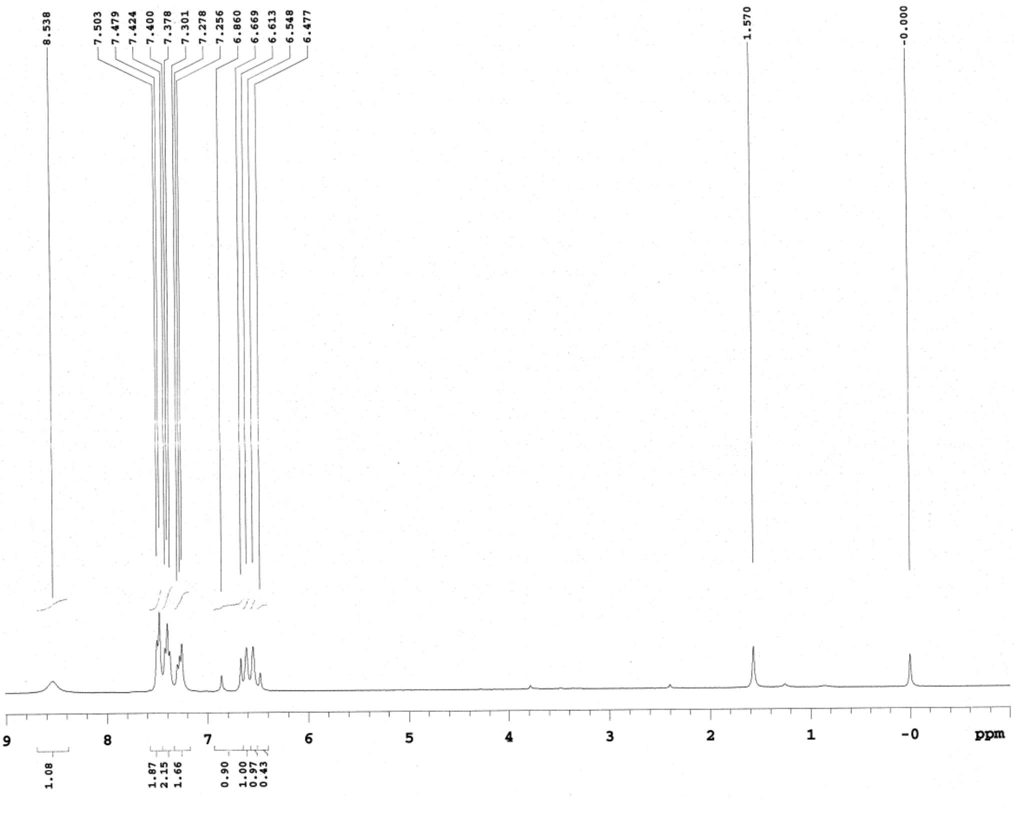

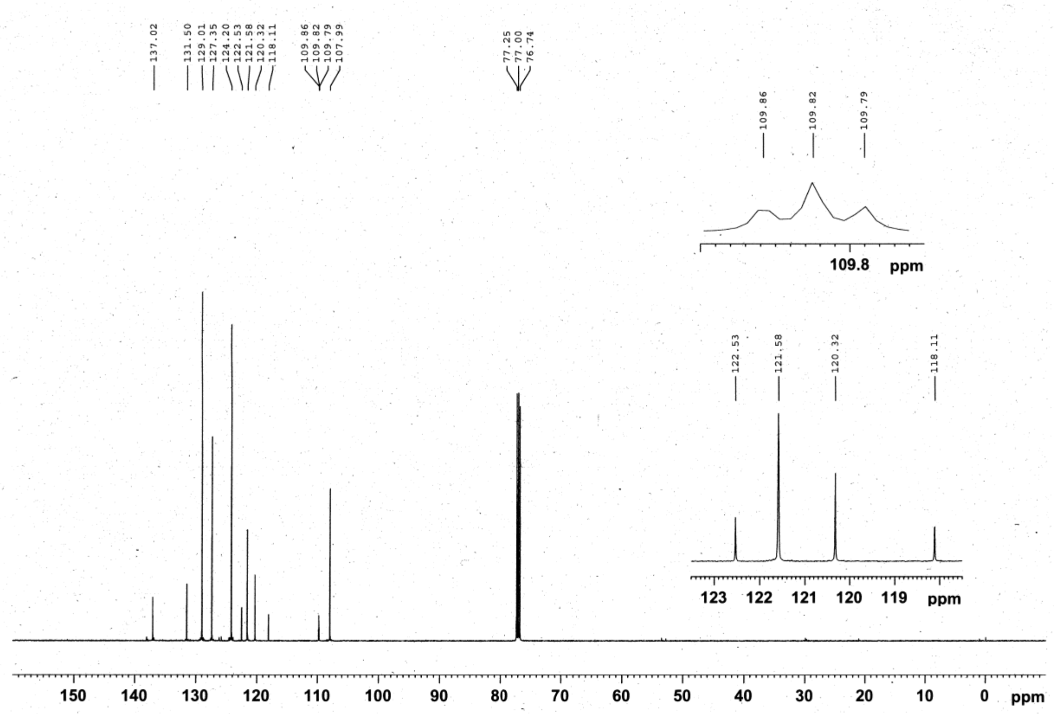

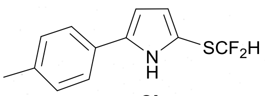

8b

^{19}F NMR (282 MHz, $CDCl_3$)
^{1}H NMR (300 MHz, $CDCl_3$)
^{13}C NMR (125 MHz, $CDCl_3$)

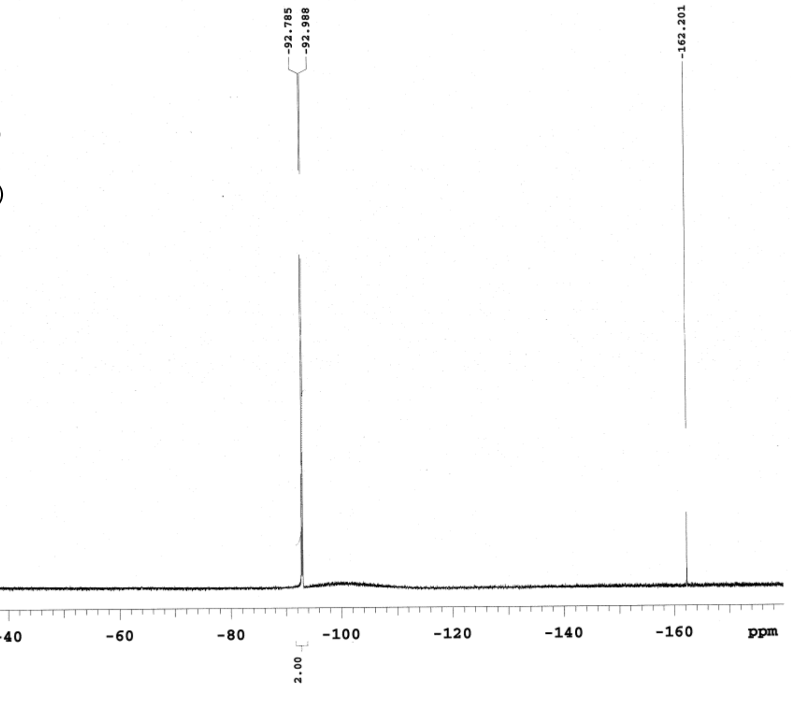

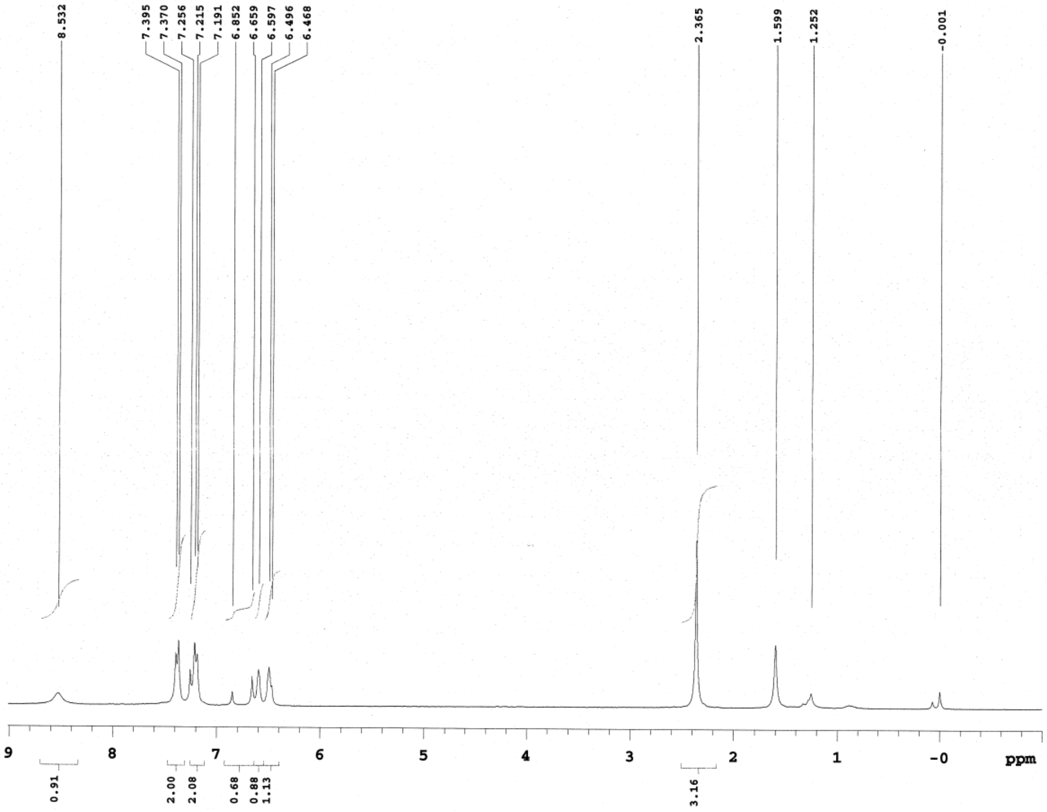
8.532
7.395
7.370
7.256
7.215
7.191
6.852
6.659
6.597
6.496
6.468
2.365
1.599
1.252
-0.001
0.91
2.00
2.08
0.68
0.88
1.13
3.16
ppm

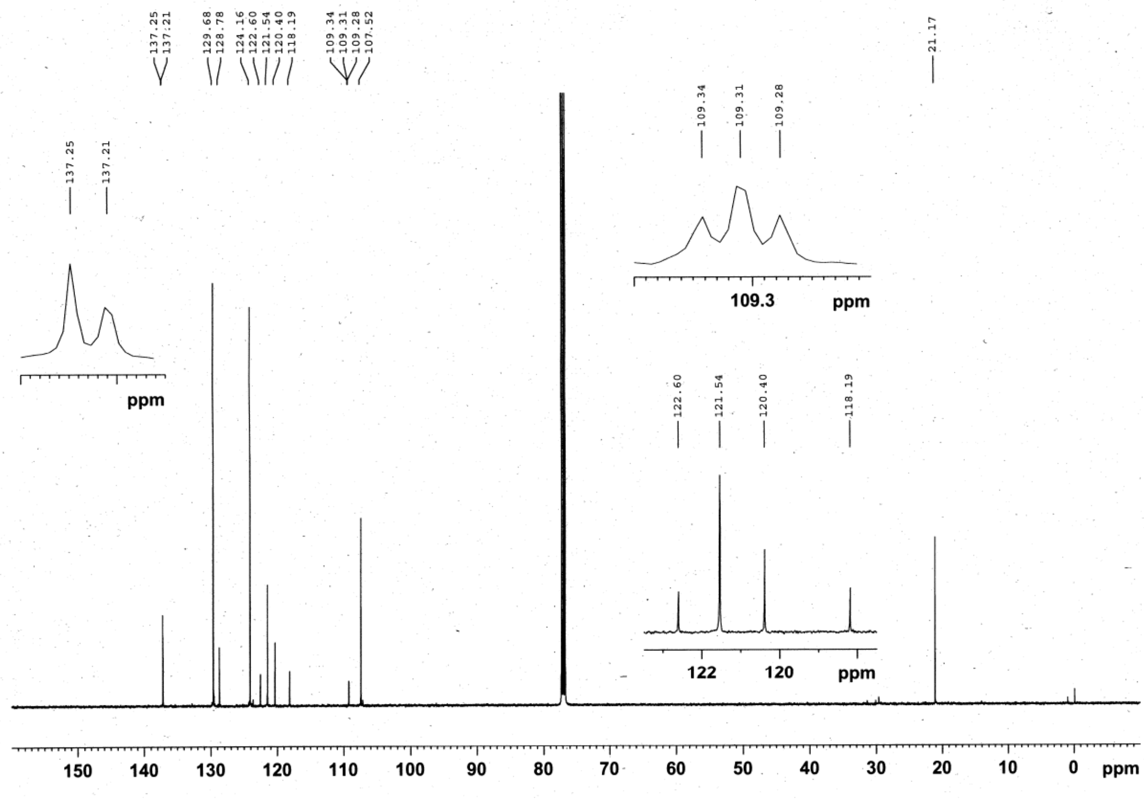
137.25
137.21
129.68
128.78
124.16
122.60
121.54
120.40
118.19
109.34
109.31
109.28
107.52
21.17
109.3
ppm

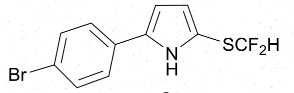

8c

^{19}F NMR (282 MHz, $CDCl_3$)
^{1}H NMR (300 MHz, $CDCl_3$)
^{13}C NMR (125 MHz, $CDCl_3$)

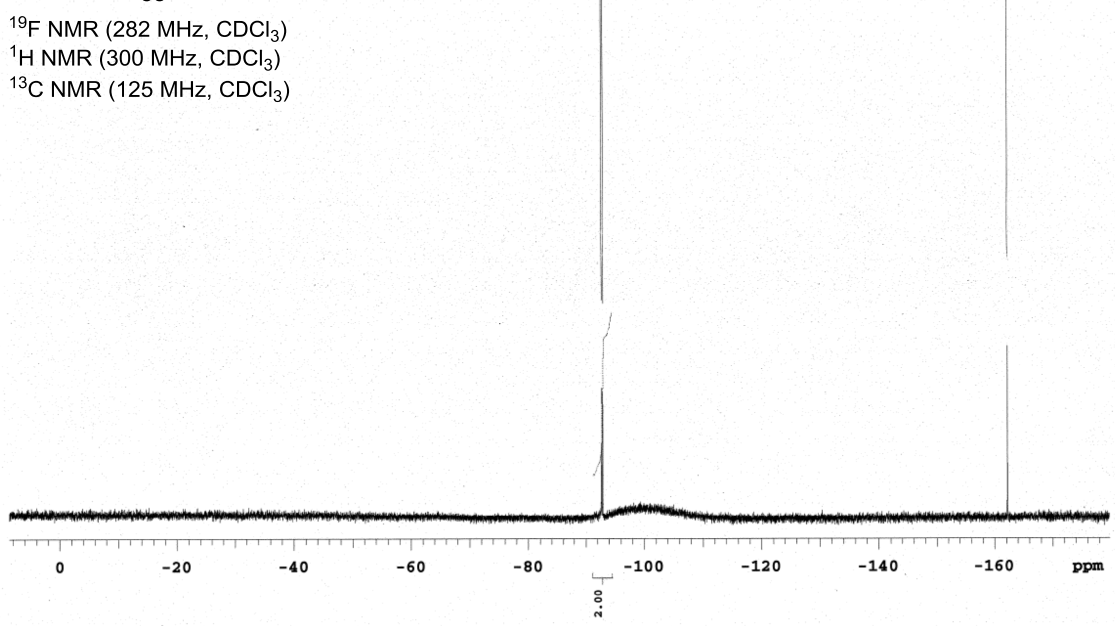

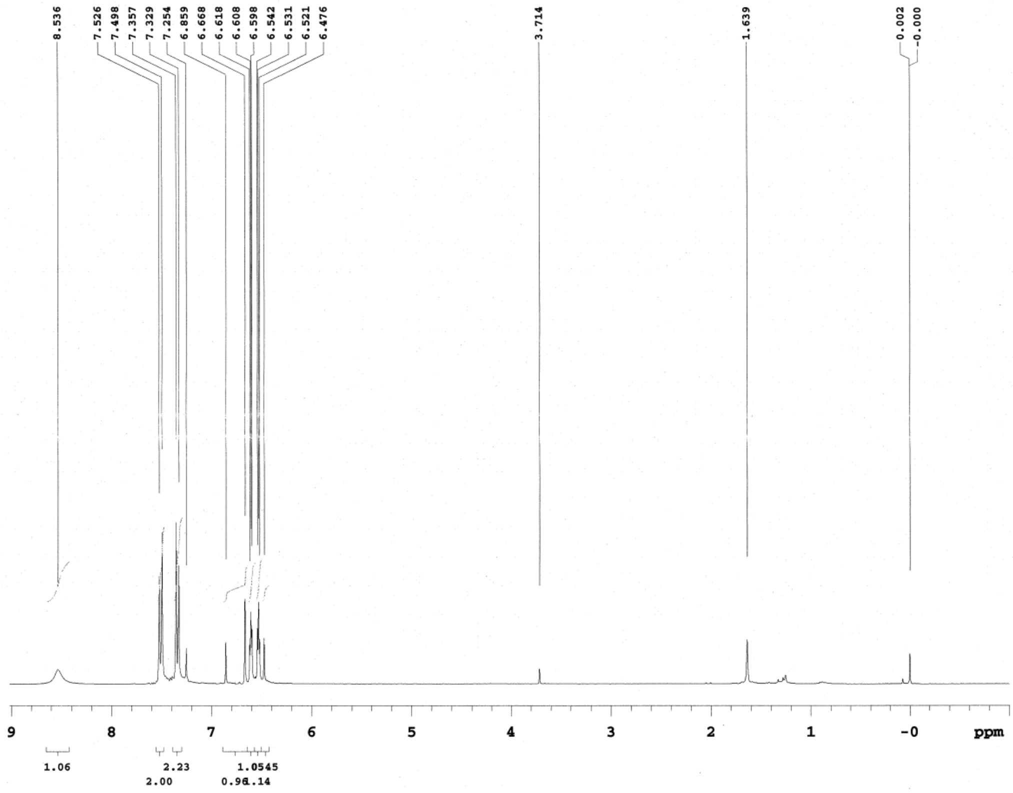

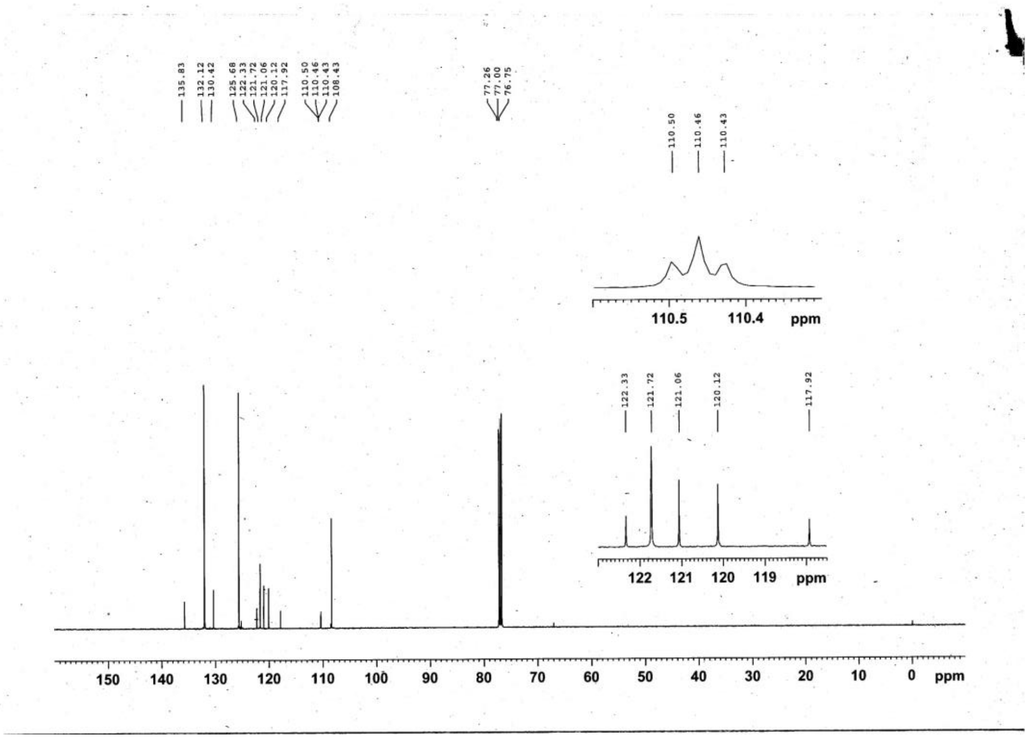

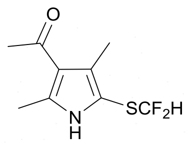

8d

^{19}F NMR (282 MHz, $CDCl_3$)

^{1}H NMR (300 MHz, $CDCl_3$)

^{13}C NMR (125 MHz, $CDCl_3$)

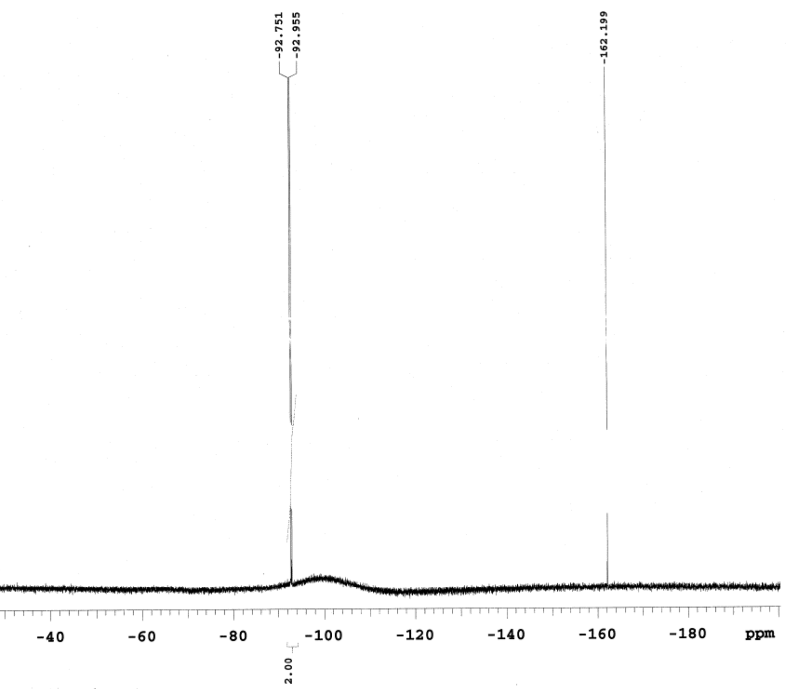

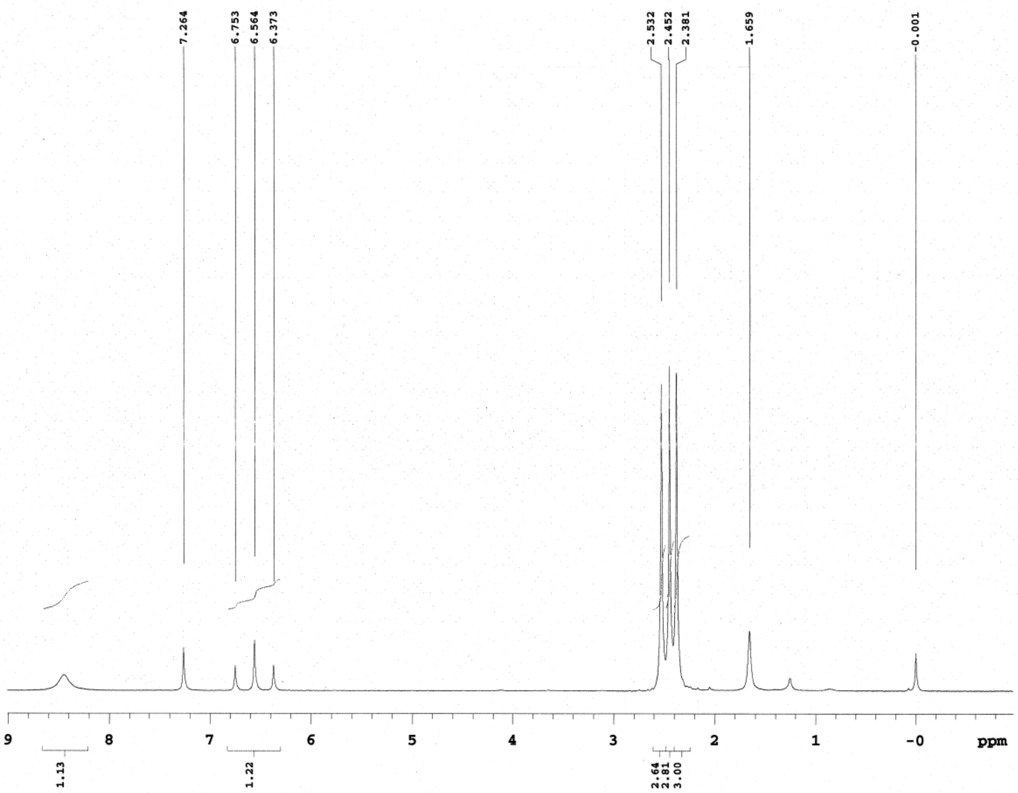
7.264
6.753
6.564
6.373
2.532
2.452
2.381
1.659
-0.001
9 8 7 6 5 4 3 2 1 -0 ppm
1.13
1.22
2.64
2.81
3.00

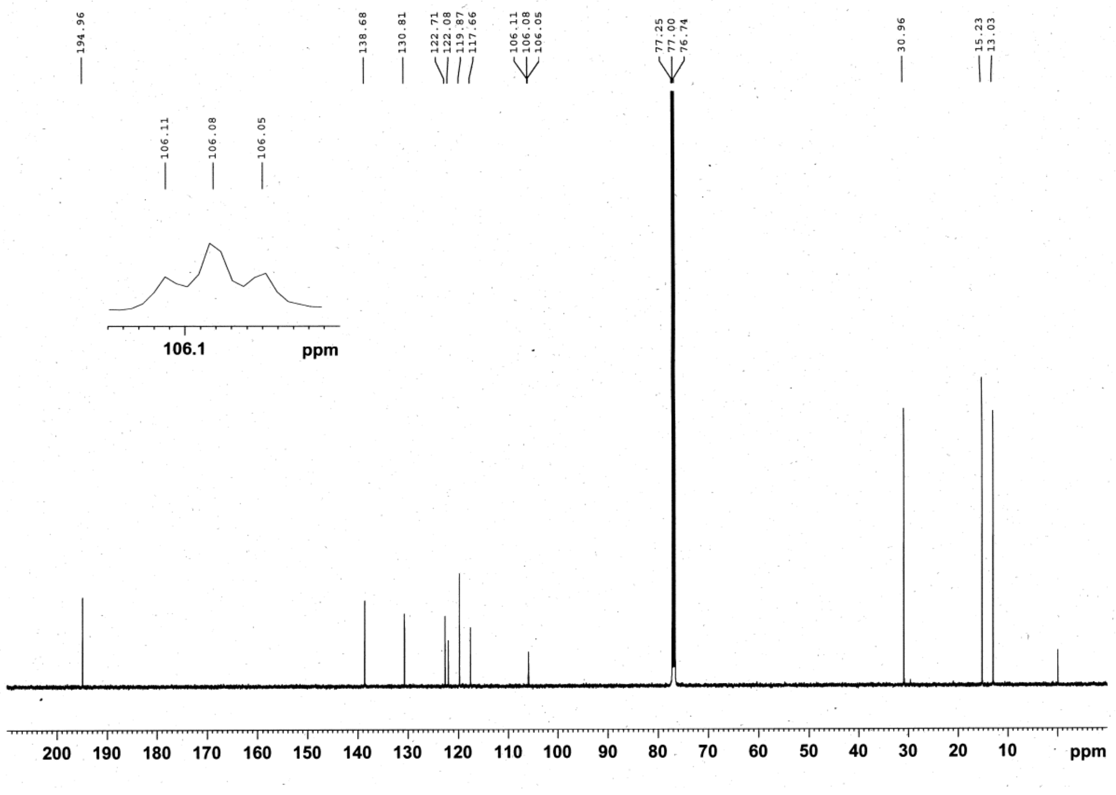
194.96
138.68
130.81
122.71
122.08
119.87
117.66
106.11
106.08
106.05
77.25
77.00
76.74
30.96
15.23
13.03
106.11
106.08
106.05
106.1
ppm
200 190 180 170 160 150 140 130 120 110 100 90 80 70 60 50 40 30 20 10 ppm

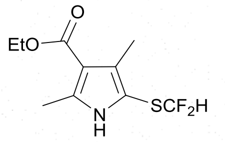

8e

^{19}F NMR (282 MHz, $CDCl_3$)

^{1}H NMR (300 MHz, $CDCl_3$)

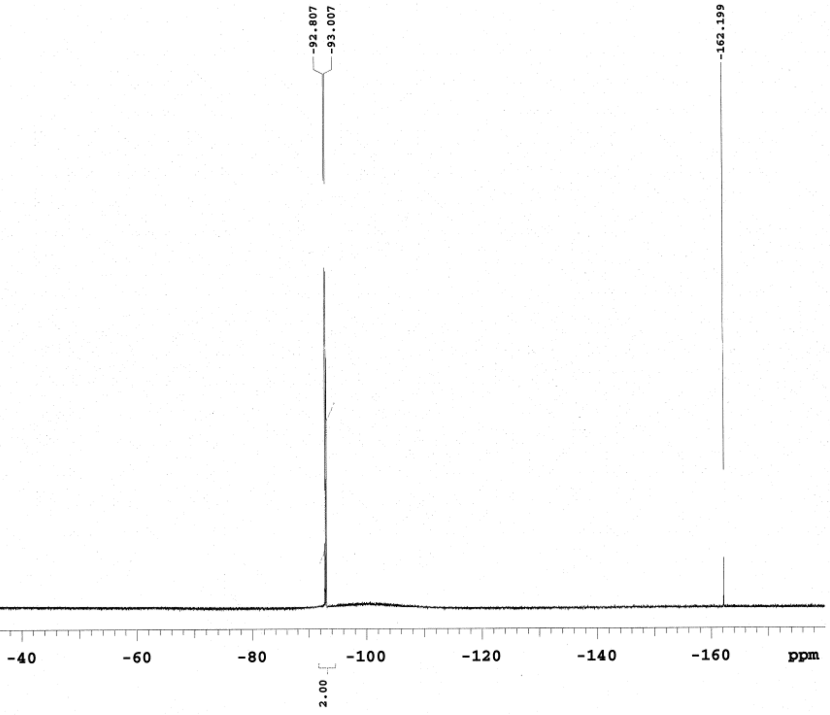

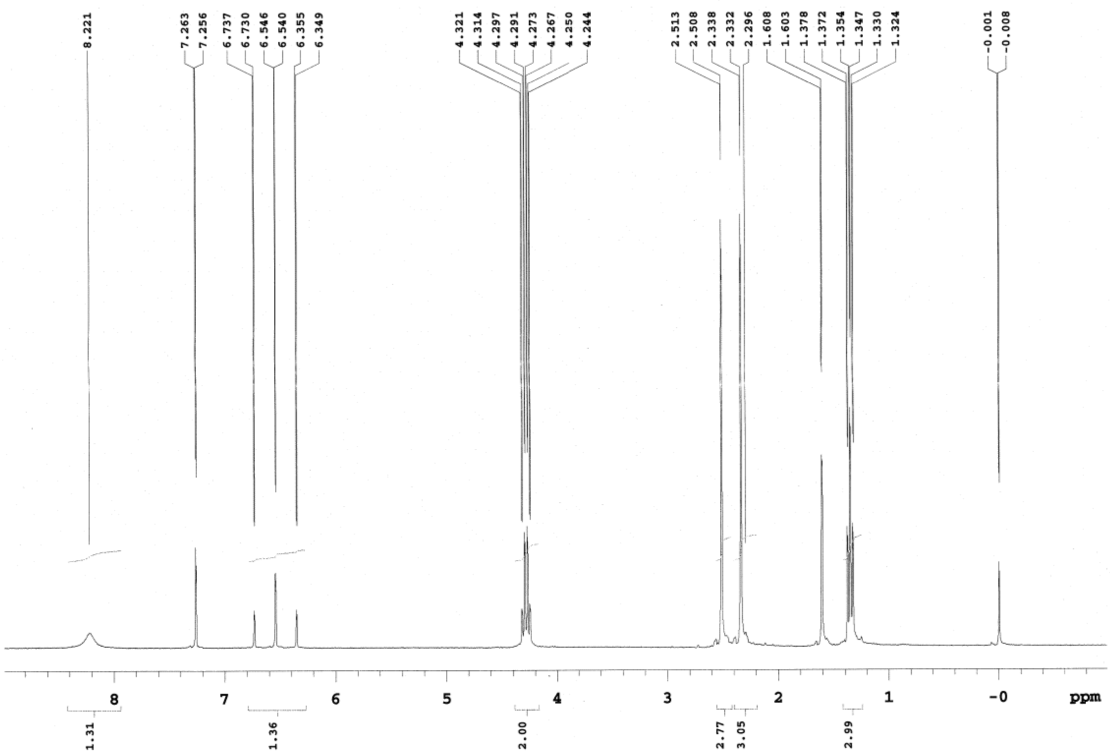

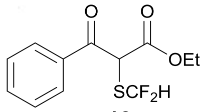

10p

^{19}F NMR (282 MHz, $CDCl_3$)
^{1}H NMR (300 MHz, $CDCl_3$)
^{13}C NMR (125 MHz, $CDCl_3$)

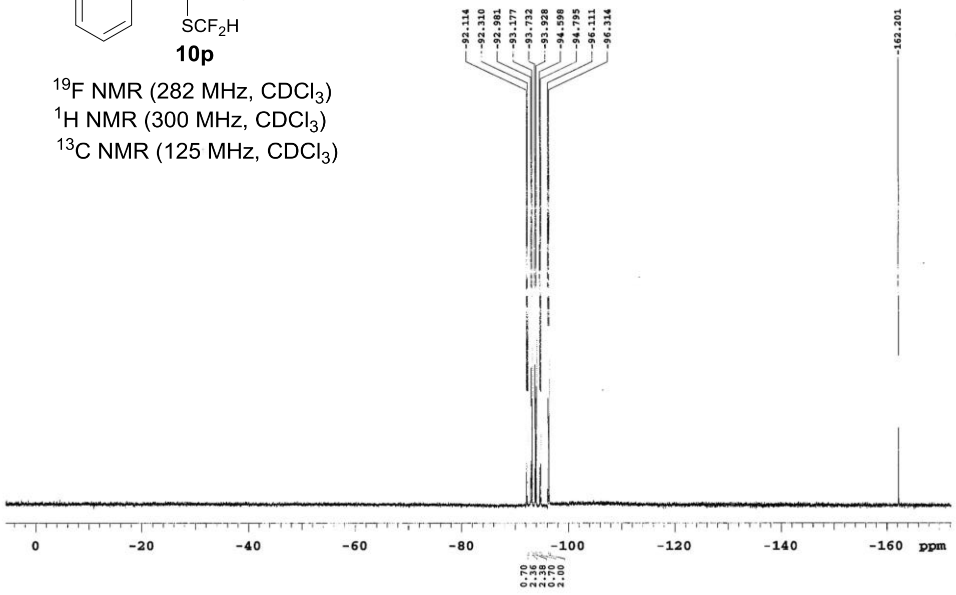

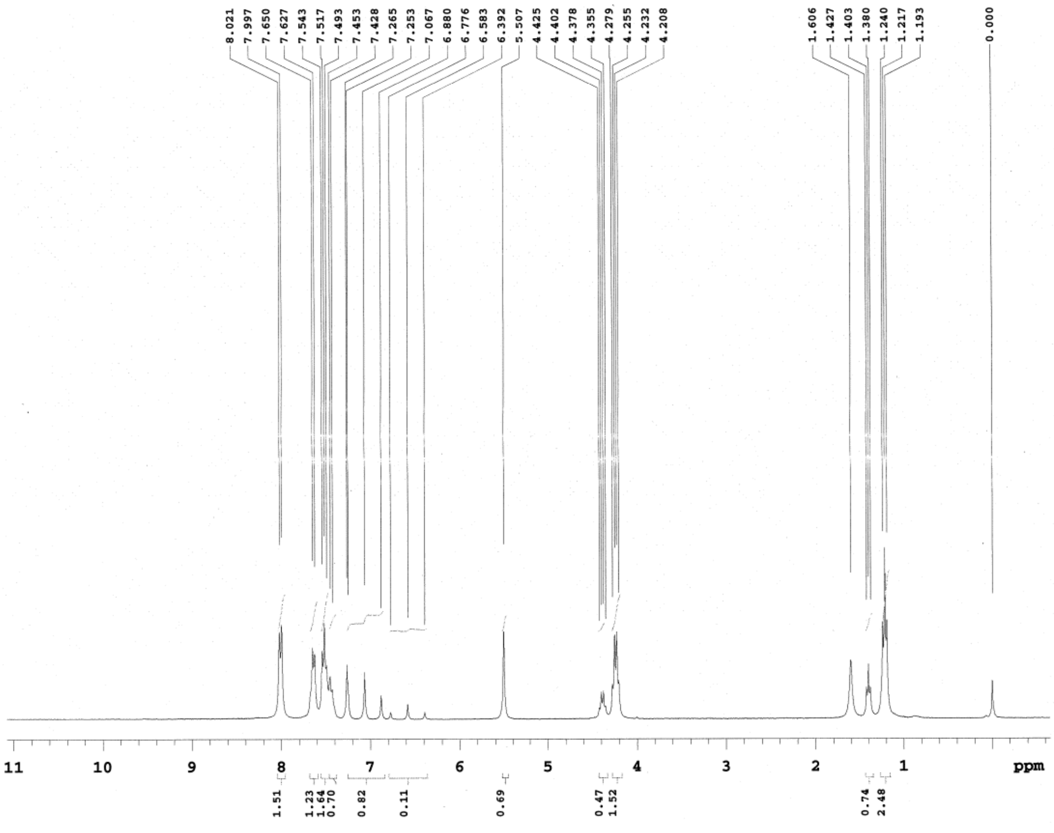

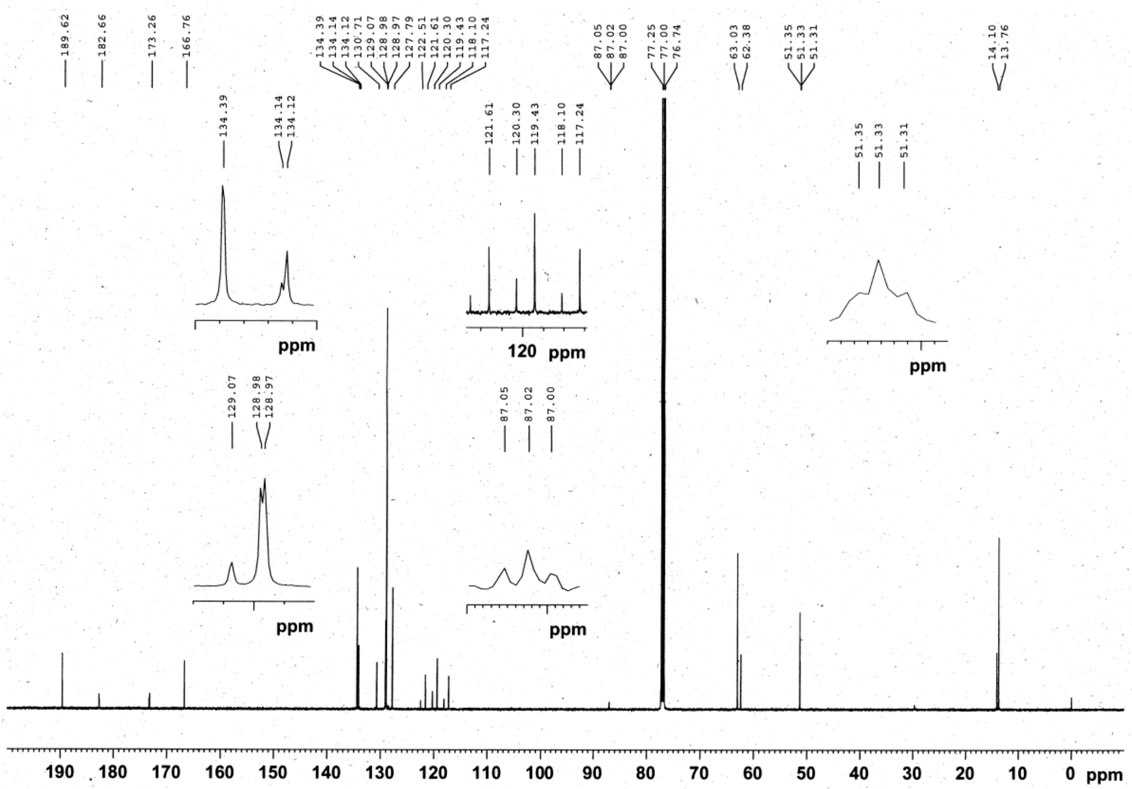

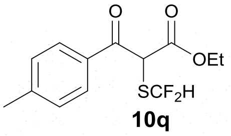

10q

^{19}F NMR (282 MHz, $CDCl_3$)
^{1}H NMR (300 MHz, $CDCl_3$)
^{13}C NMR (125 MHz, $CDCl_3$)

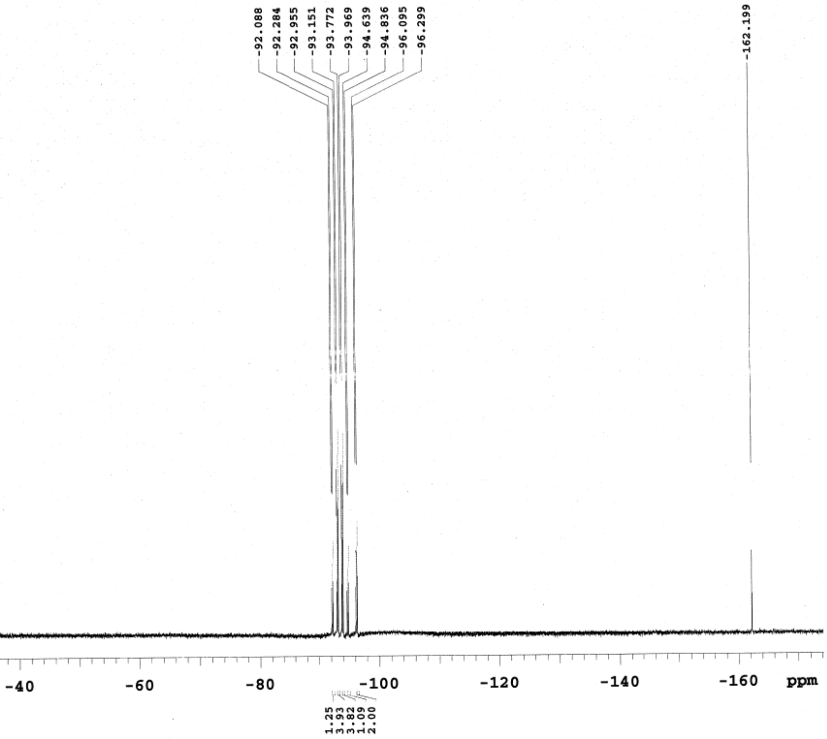

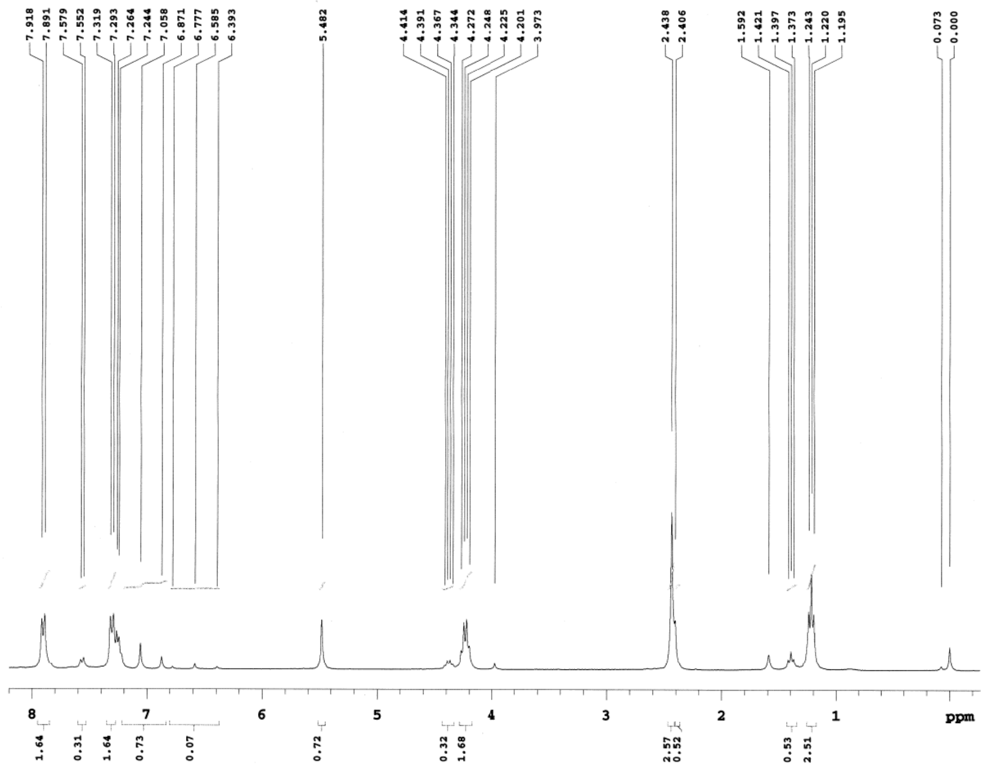
7.918
7.891
7.579
7.552
7.319
7.293
7.264
7.244
7.058
6.871
6.777
6.585
6.393
5.482
4.414
4.391
4.367
4.344
4.272
4.248
4.225
4.201
3.973
2.438
2.406
1.592
1.421
1.397
1.373
1.243
1.220
1.195
0.073
0.000
8 7 6 5 4 3 2 1 ppm
1.64
0.31
1.64
0.73
0.07
0.72
0.32
1.68
2.57
0.52
0.53
2.51

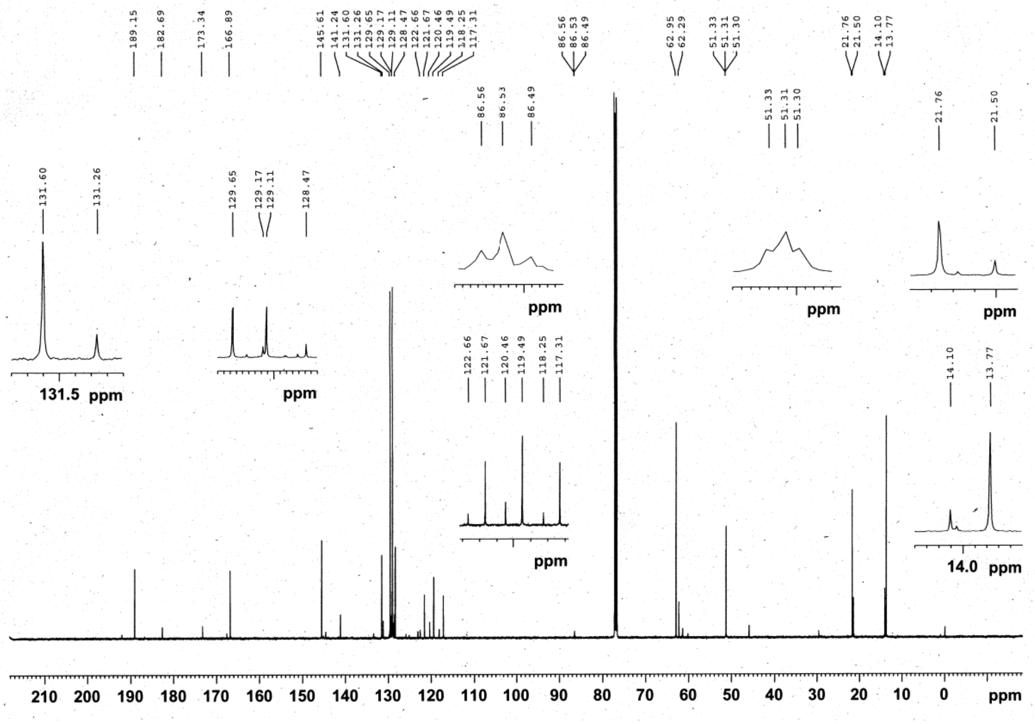
189.15
182.69
173.34
166.89
145.61
141.24
131.60
131.26
129.65
129.17
129.11
128.47
122.66
121.67
120.46
119.49
118.25
117.31
86.56
86.53
86.49
62.95
62.29
51.33
51.31
51.30
21.76
21.50
14.10
13.77
131.5 ppm
ppm
14.0 ppm
210 200 190 180 170 160 150 140 130 120 110 100 90 80 70 60 50 40 30 20 10 0 ppm

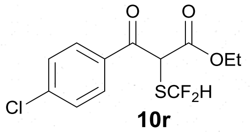

10r

^{19}F NMR (282 MHz, $CDCl_3$)

^{1}H NMR (300 MHz, $CDCl_3$)

^{13}C NMR (125 MHz, $CDCl_3$)

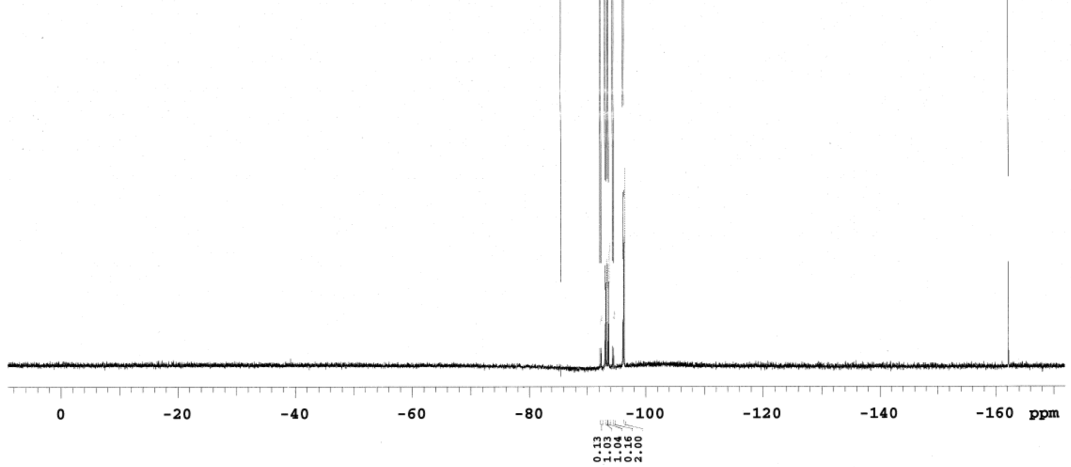

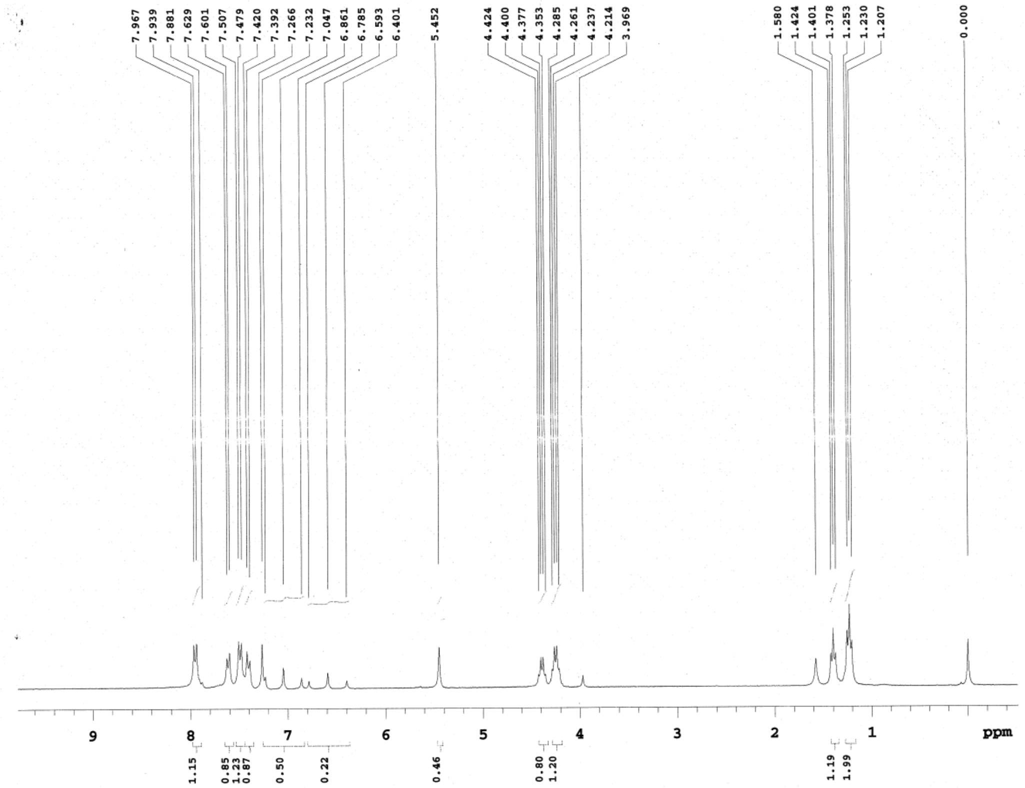

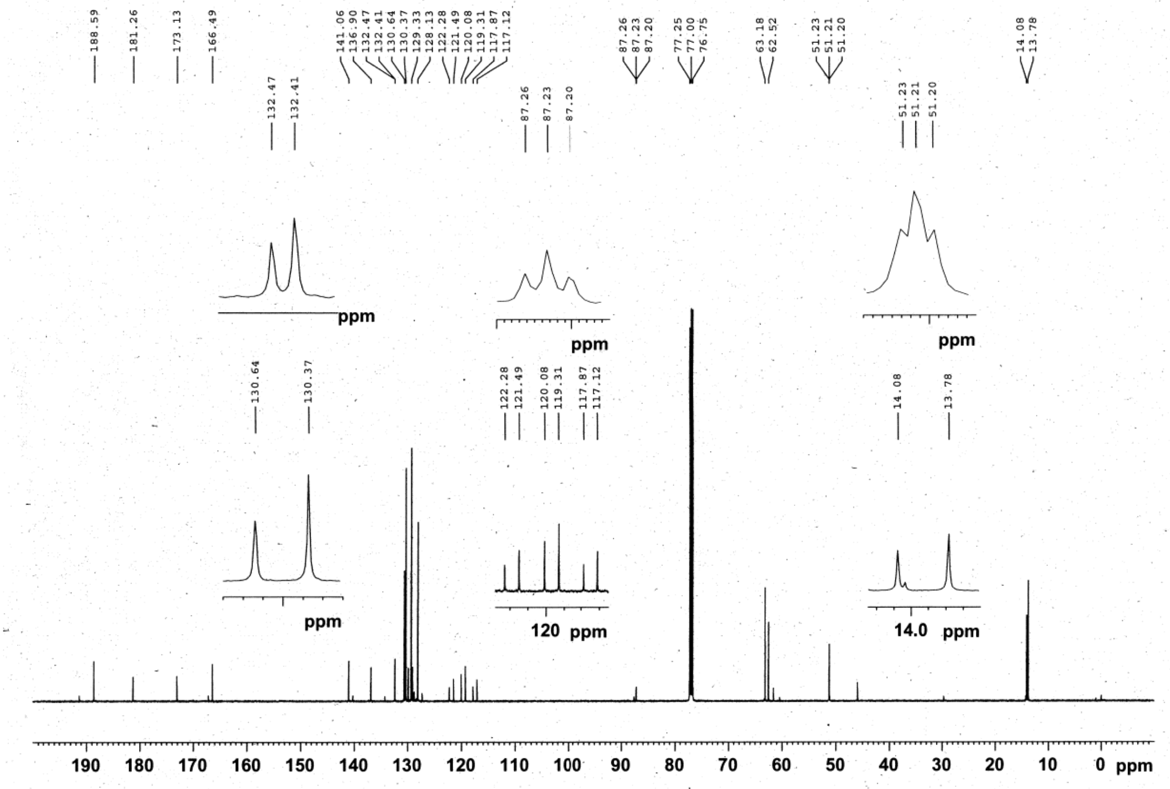

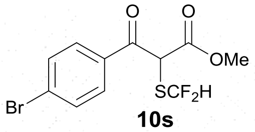

10s

^{19}F NMR (282 MHz, $CDCl_3$)

^{1}H NMR (300 MHz, $CDCl_3$)

^{13}C NMR (125 MHz, $CDCl_3$)

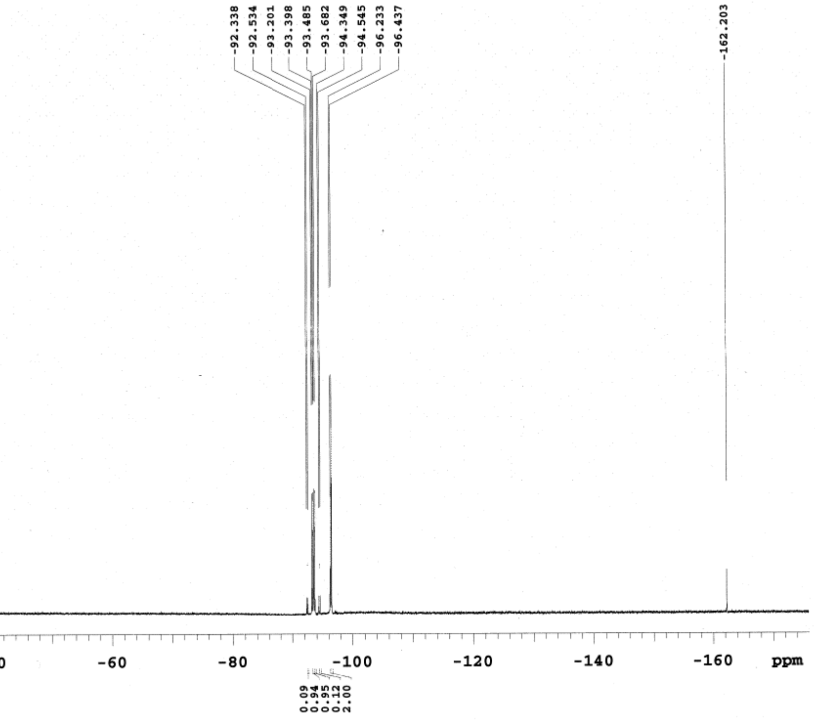

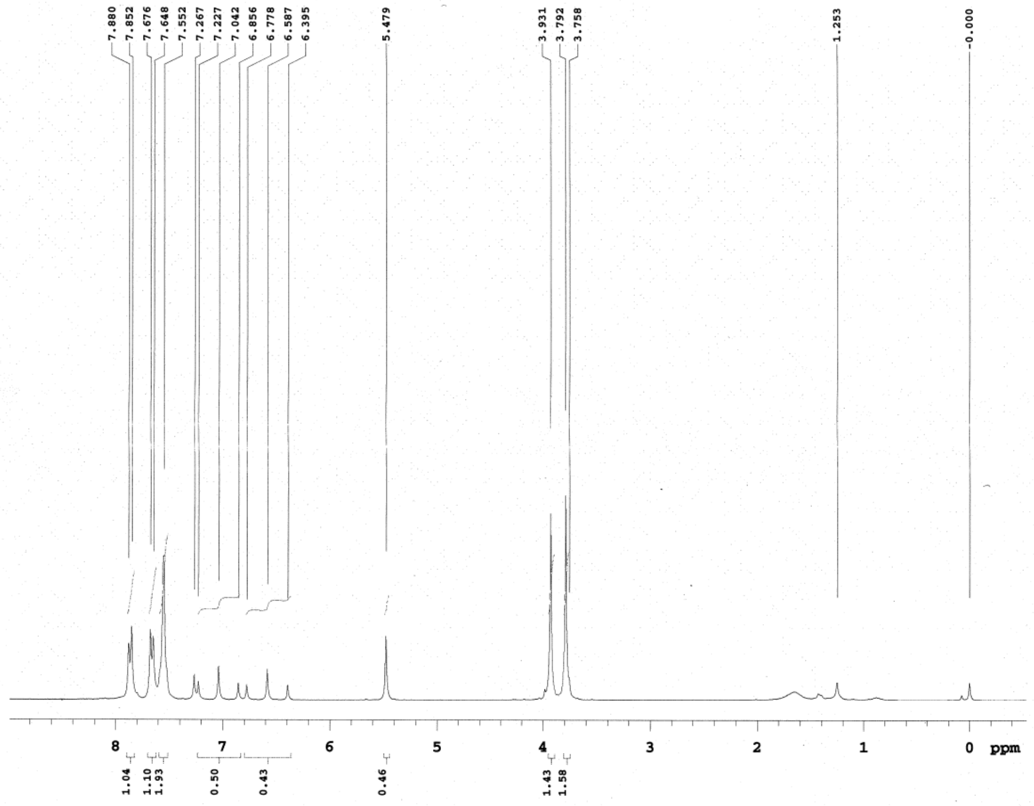
7.880
7.852
7.676
7.648
7.552
7.267
7.227
7.042
6.856
6.778
6.587
6.395
5.479
3.931
3.792
3.758
1.253
-0.000
8 7 6 5 4 3 2 1 0 ppm
1.04
1.10
1.93
0.50
0.43
0.46
1.43
1.58

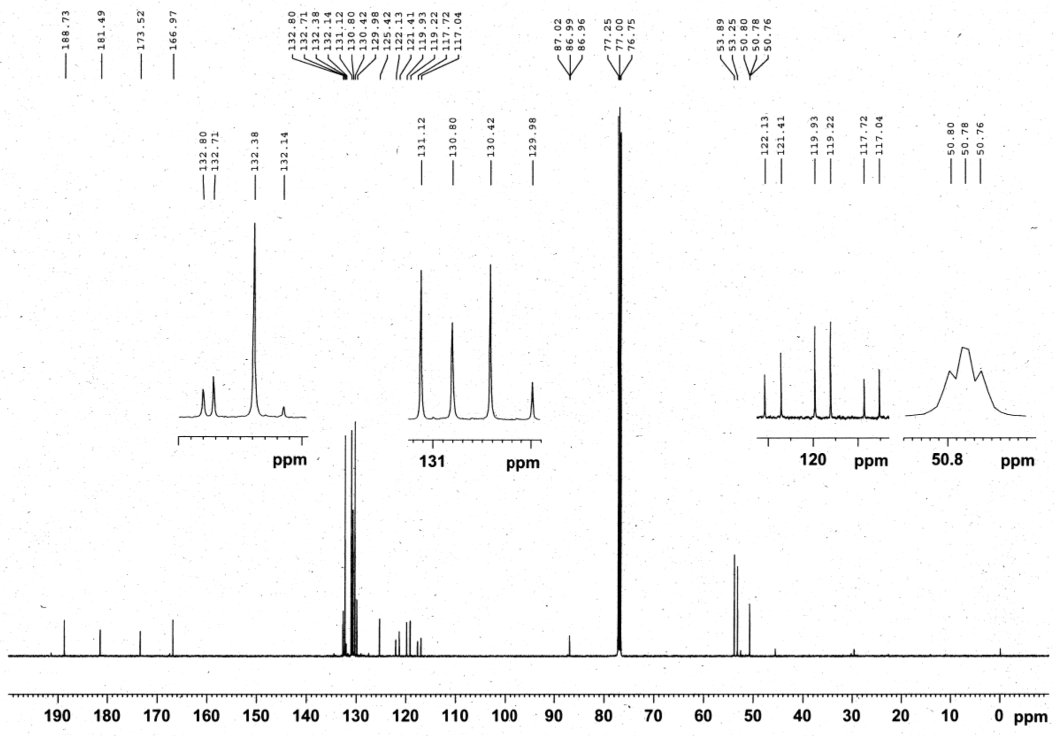
188.73
181.49
173.52
166.97
132.80
132.71
132.38
132.14
131.12
130.80
130.42
129.98
125.42
122.13
121.41
119.93
119.22
117.72
117.04
87.02
86.99
86.96
77.25
77.00
76.75
53.89
53.25
50.80
50.78
50.76
ppm
131
ppm
120
ppm
50.8
ppm
190 180 170 160 150 140 130 120 110 100 90 80 70 60 50 40 30 20 10 0 ppm

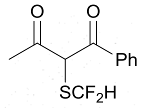

10t

^{19}F NMR (282 MHz, $CDCl_3$)
^{1}H NMR (300 MHz, $CDCl_3$)
^{13}C NMR (125 MHz, $CDCl_3$)

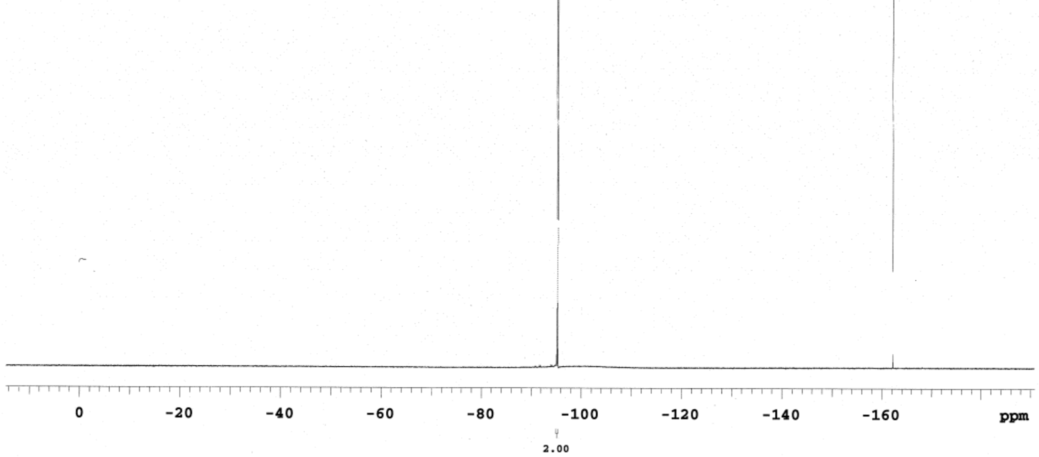

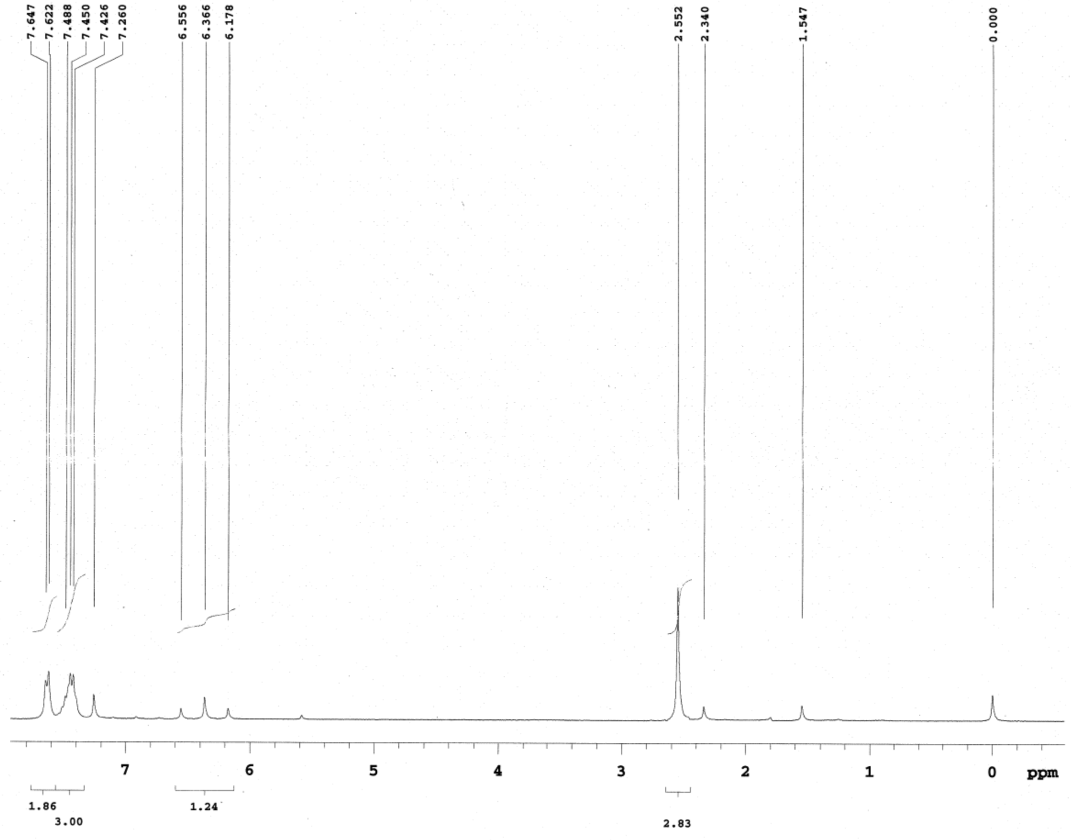

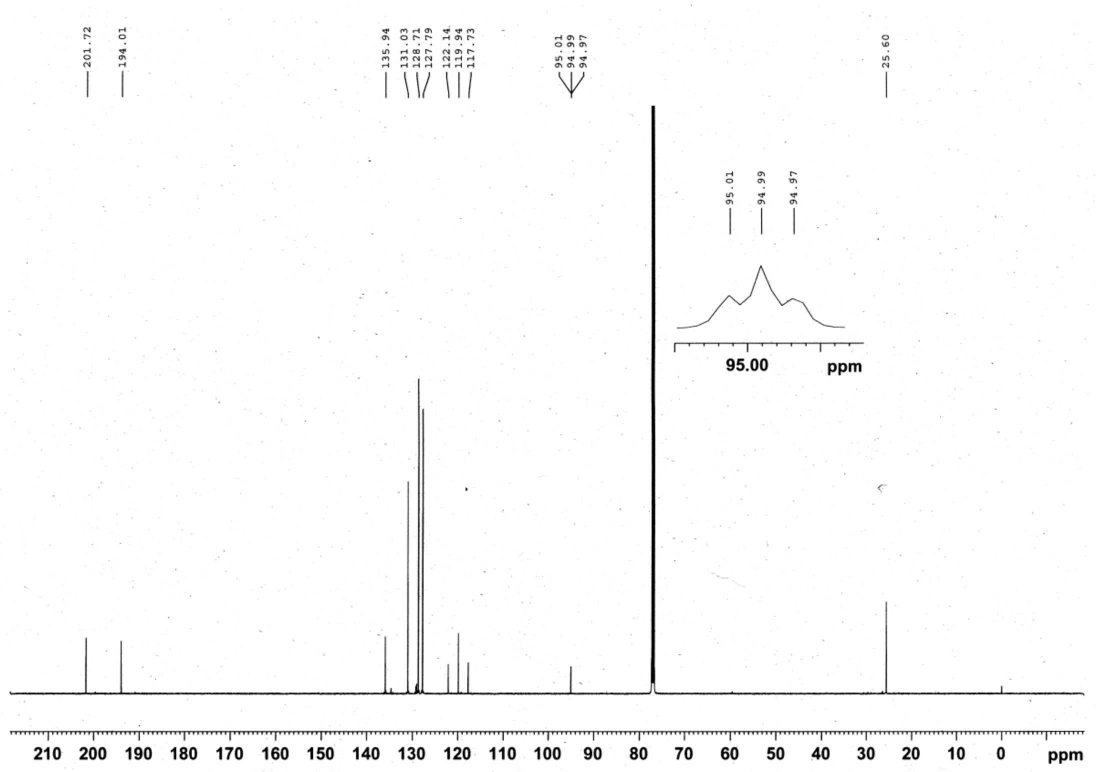

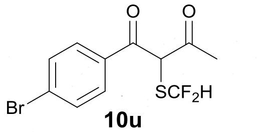

10u

^{19}F NMR (282 MHz, $CDCl_3$)
^{1}H NMR (300 MHz, $CDCl_3$)
^{13}C NMR (125 MHz, $CDCl_3$)

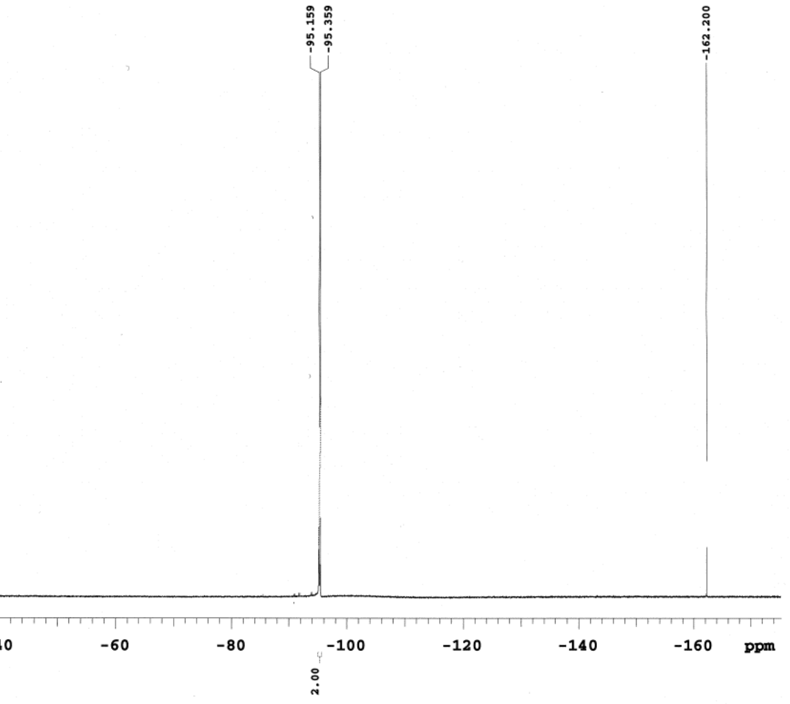

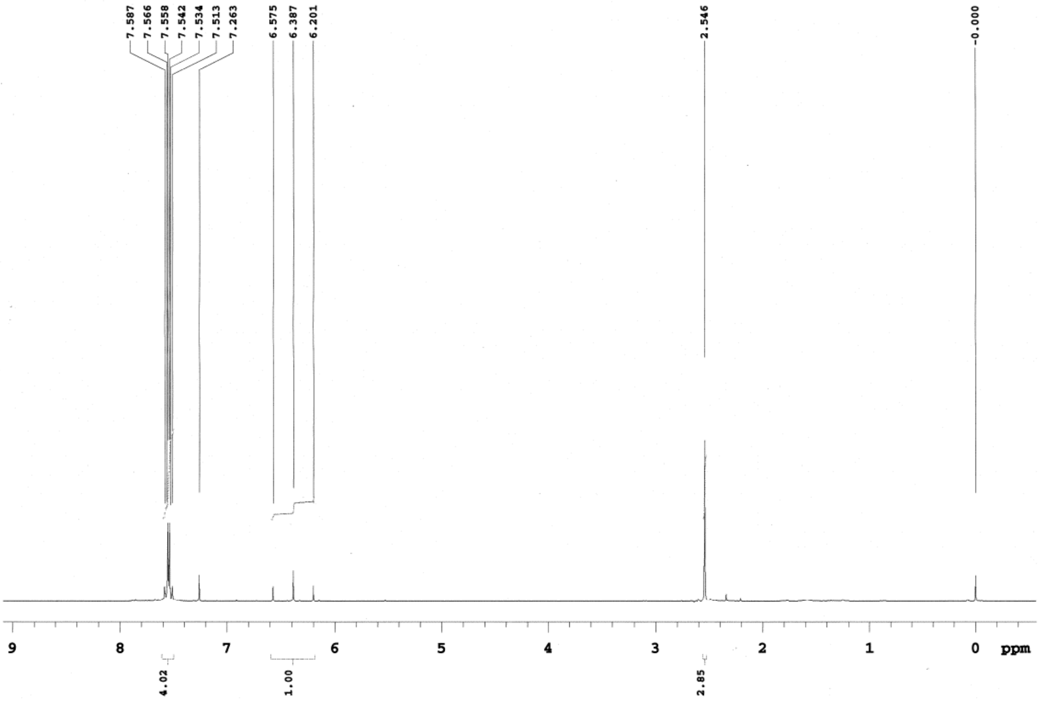
7.587
7.566
7.558
7.542
7.534
7.513
7.263
6.575
6.387
6.201
2.546
-0.000
9 8 7 6 5 4 3 2 1 0 ppm
4.02
1.00
2.85

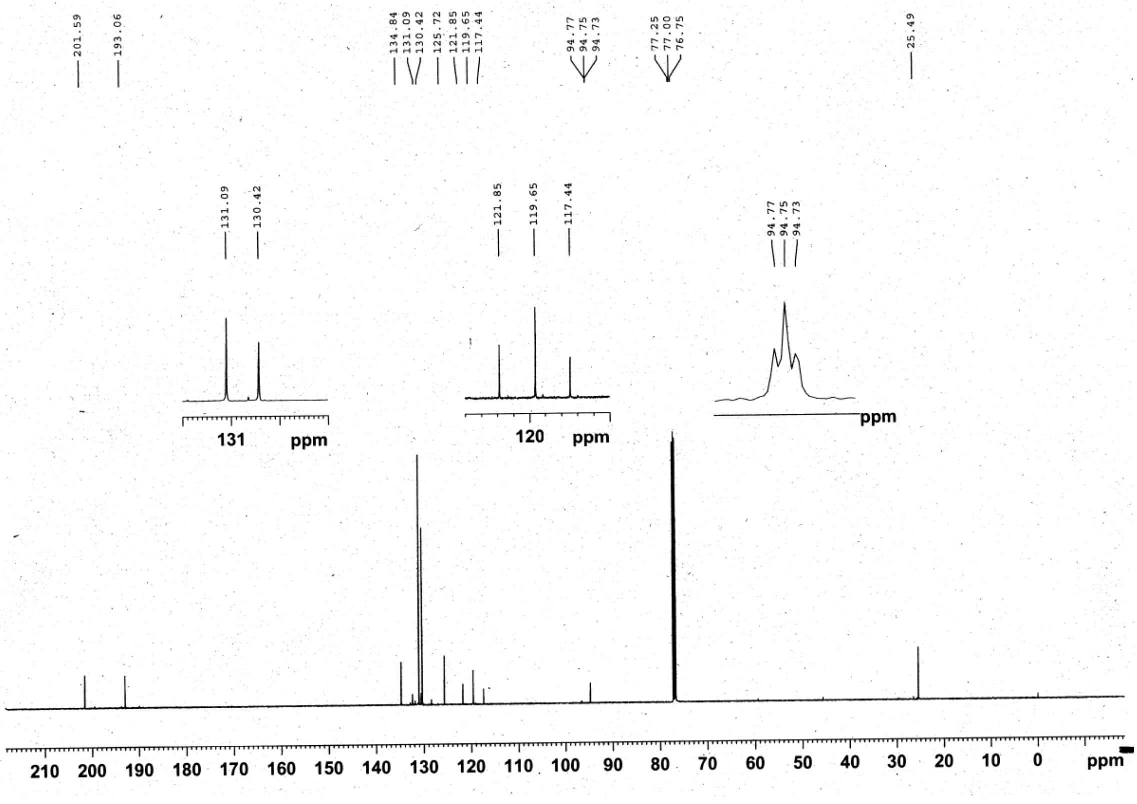
201.59
193.06
134.84
131.09
130.42
125.72
121.85
119.65
117.44
94.77
94.75
94.73
77.25
77.00
76.75
25.49
131.09
130.42
131 ppm
121.85
119.65
117.44
120 ppm
94.77
94.75
94.73
ppm
210 200 190 180 170 160 150 140 130 120 110 100 90 80 70 60 50 40 30 20 10 0 ppm

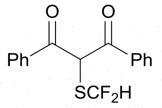

10v

^{19}F NMR (282 MHz, $CDCl_3$)
^{1}H NMR (300 MHz, $CDCl_3$)
^{13}C NMR (125 MHz, $CDCl_3$)

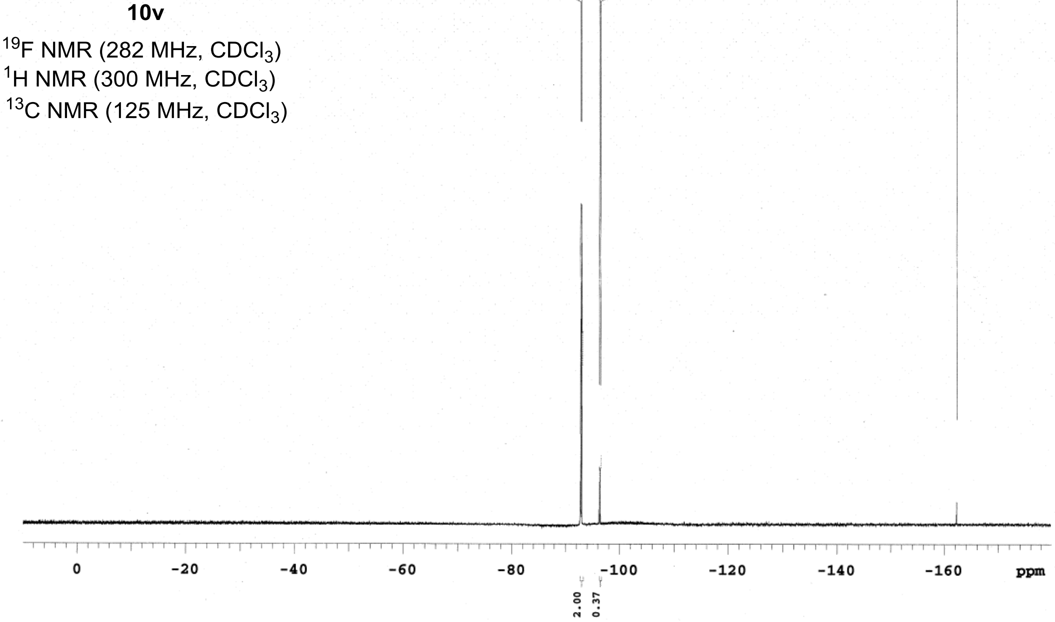

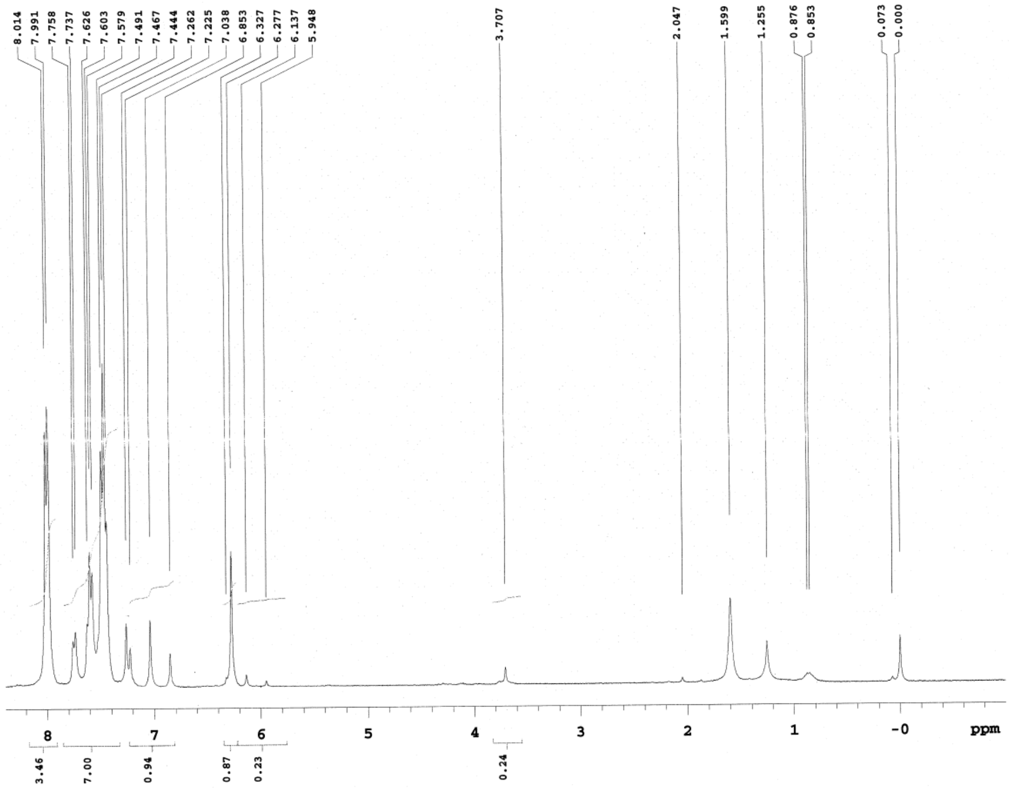

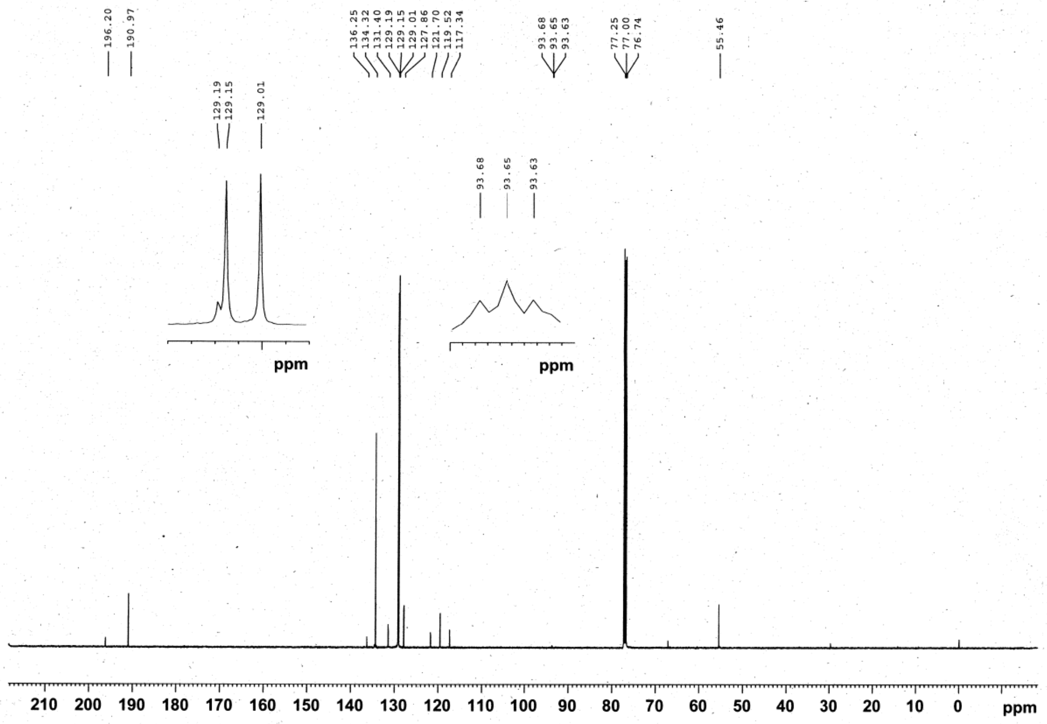

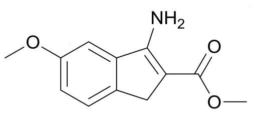

3x

^{1}H NMR (300 MHz, $CDCl_3$)

^{13}C NMR (125 MHz, $CDCl_3$)

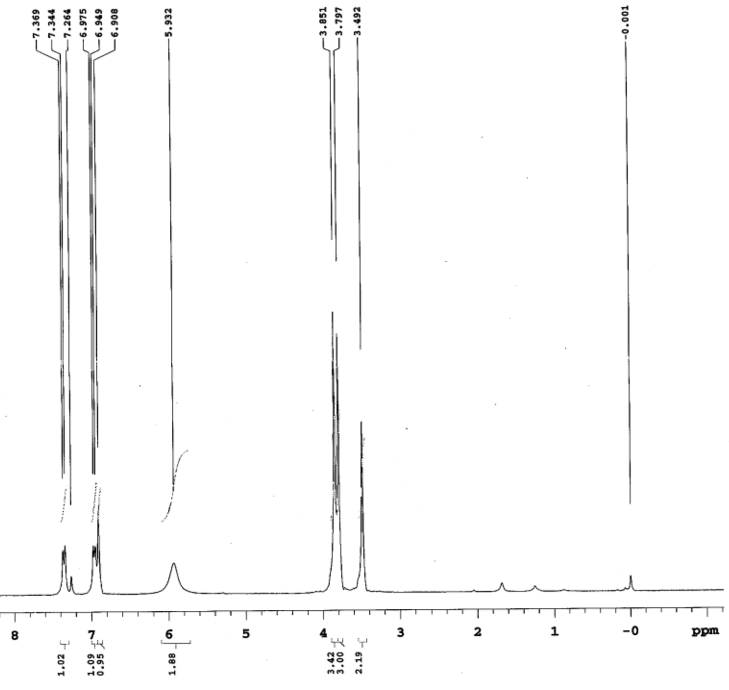

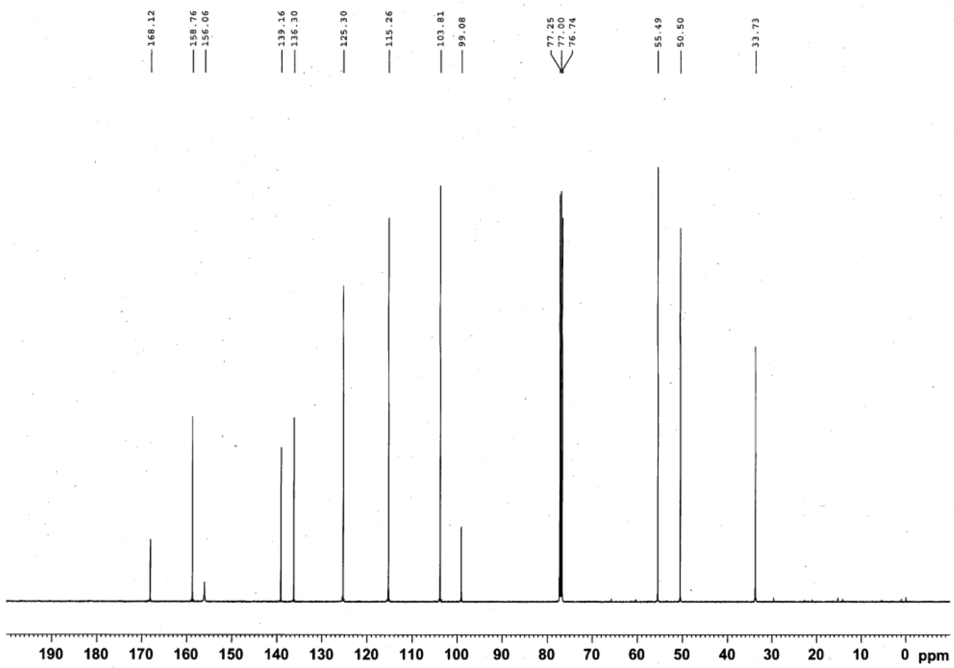

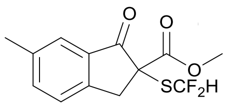

10w

^{19}F NMR (282 MHz, $CDCl_3$)
^{1}H NMR (300 MHz, $CDCl_3$)

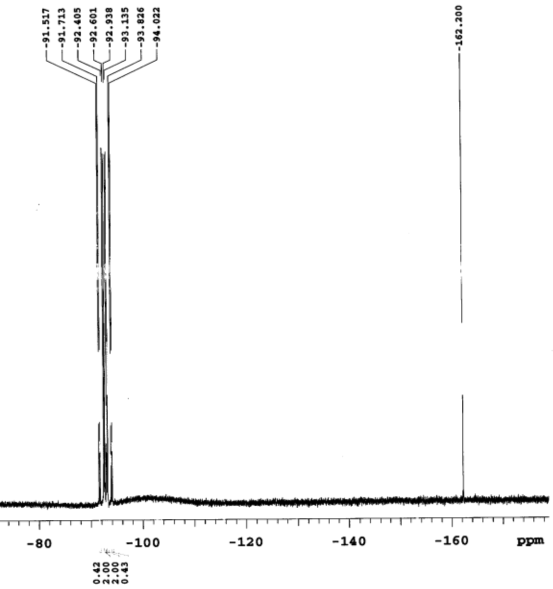

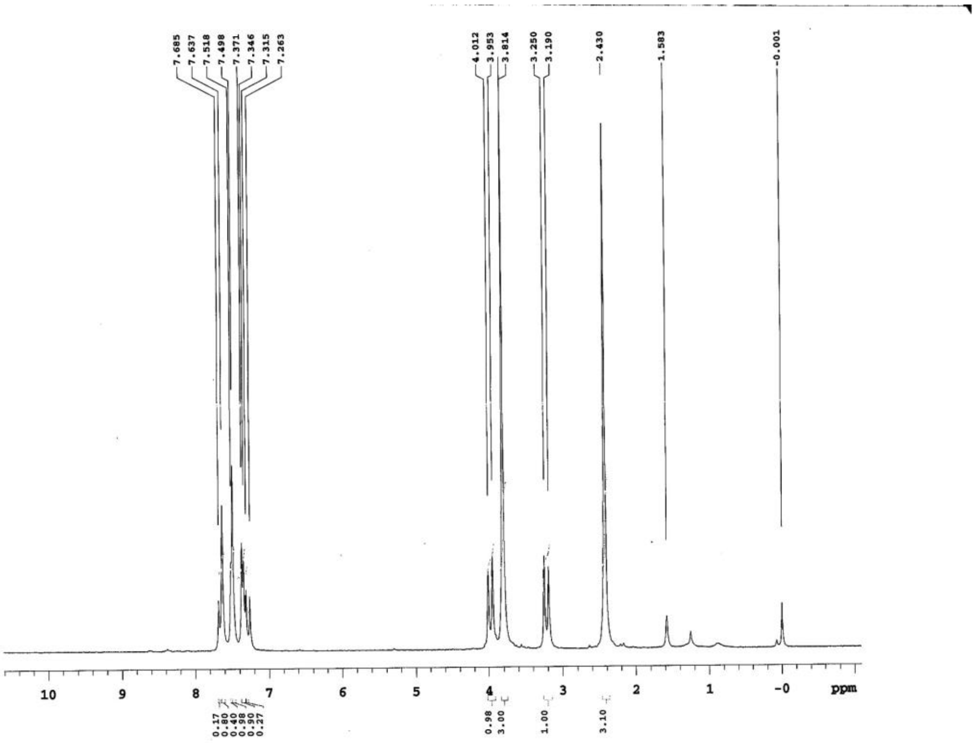

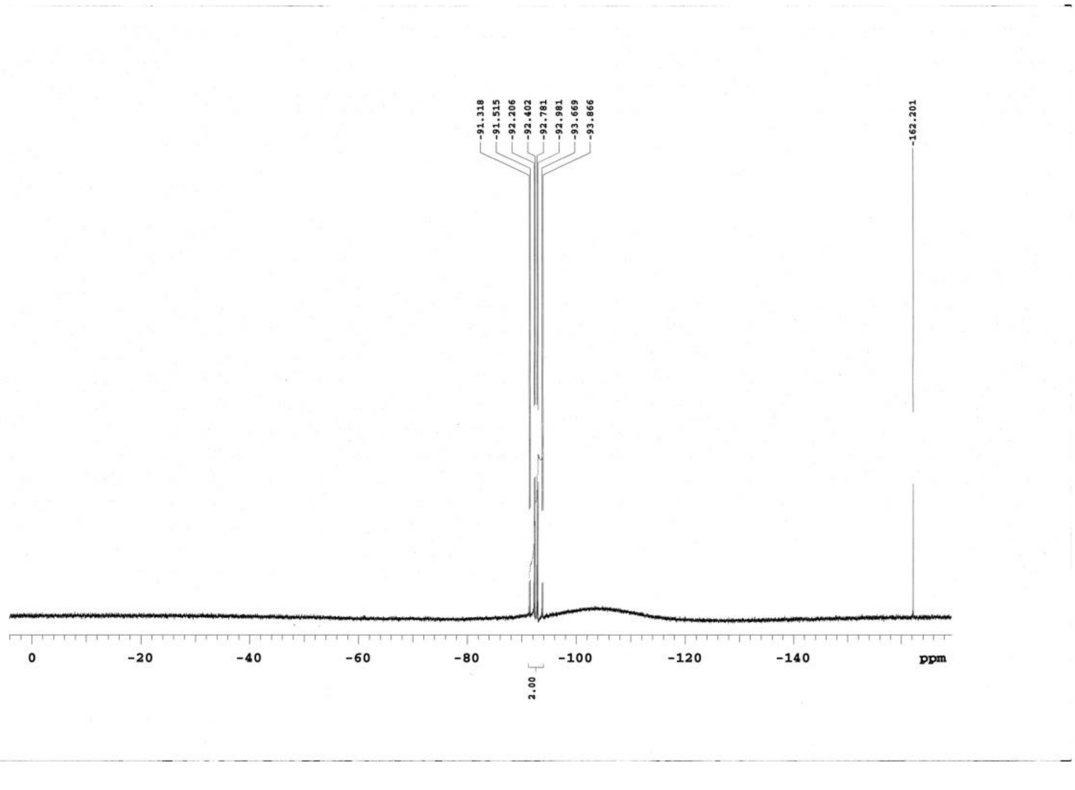

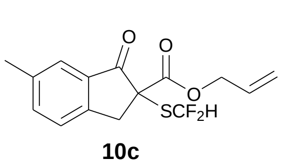

10c

^{19}F NMR (282 MHz, $CDCl_3$)

^{1}H NMR (300 MHz, $CDCl_3$)

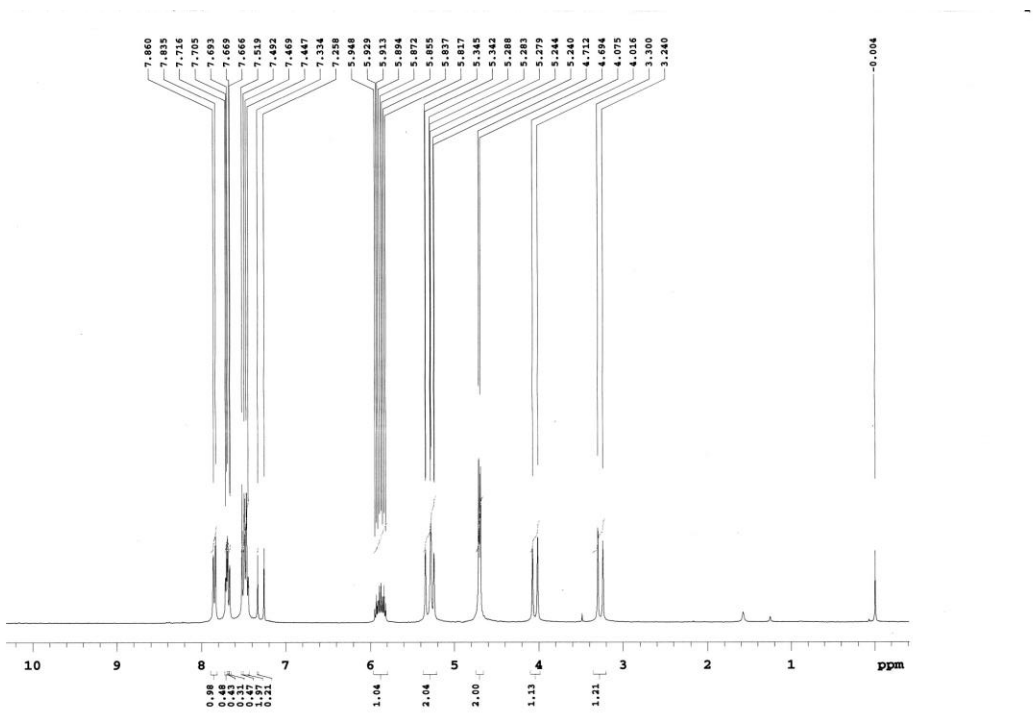
7.860
7.835
7.716
7.705
7.693
7.669
7.666
7.519
7.492
7.469
7.447
7.334
7.258
5.948
5.929
5.913
5.894
5.872
5.855
5.837
5.817
5.345
5.342
5.288
5.283
5.279
5.244
5.240
4.712
4.694
4.075
4.016
3.300
3.240
-0.004
10 9 8 7 6 5 4 3 2 1 ppm
0.98
0.48
0.43
0.31
0.47
1.97
0.21
1.04
2.04
2.00
1.13
1.21

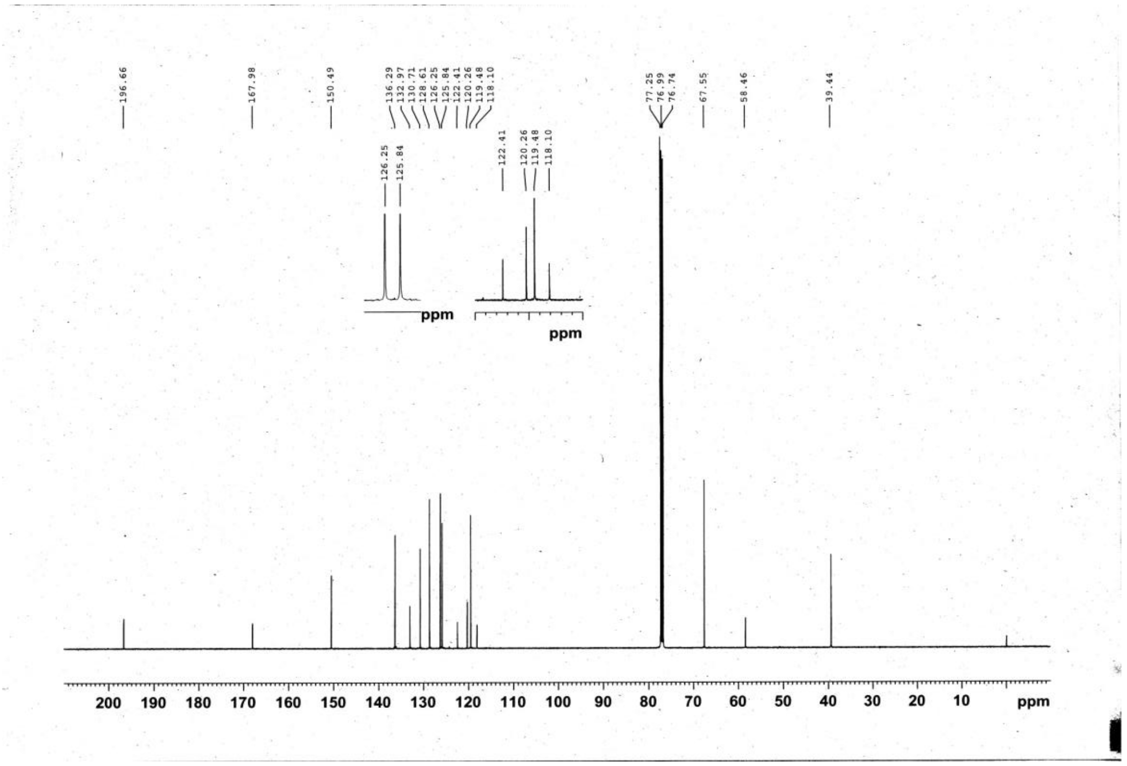
196.66
167.98
150.49
136.29
132.97
130.71
128.61
126.25
125.84
122.41
120.26
119.48
118.10
77.25
76.99
76.74
67.55
58.46
39.44
126.25
125.84
ppm
122.41
120.26
119.48
118.10
ppm
200 190 180 170 160 150 140 130 120 110 100 90 80 70 60 50 40 30 20 10 ppm

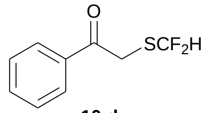

10 d

^{19}F NMR (282 MHz, $CDCl_3$)
^{1}H NMR (300 MHz, $CDCl_3$)
^{13}C NMR (125 MHz, $CDCl_3$)

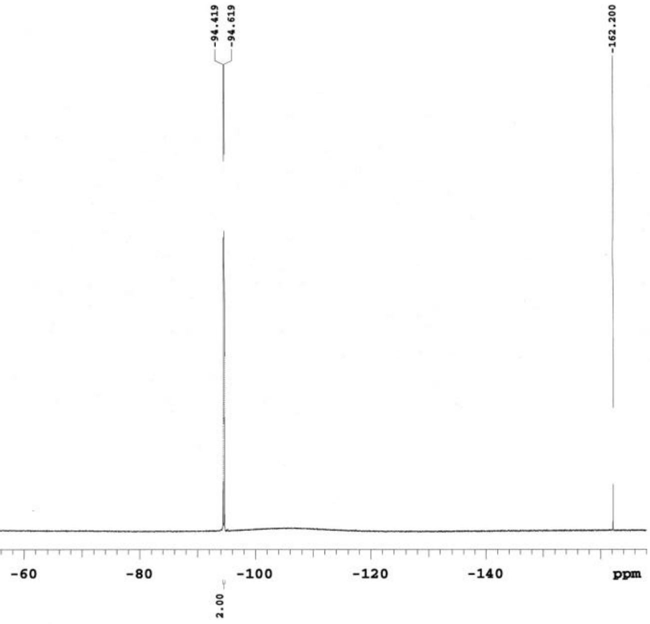

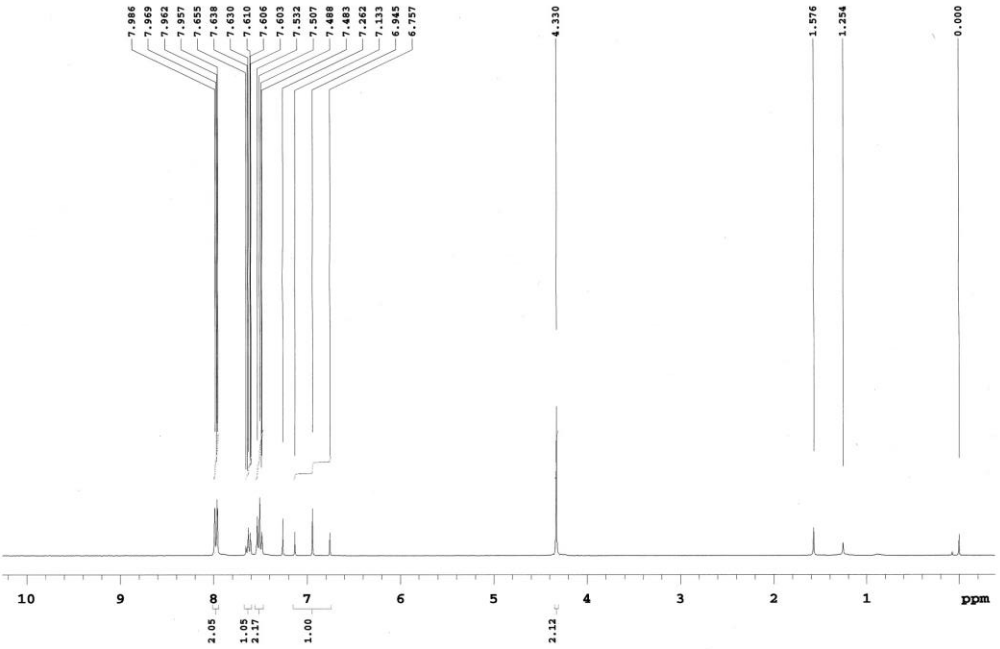

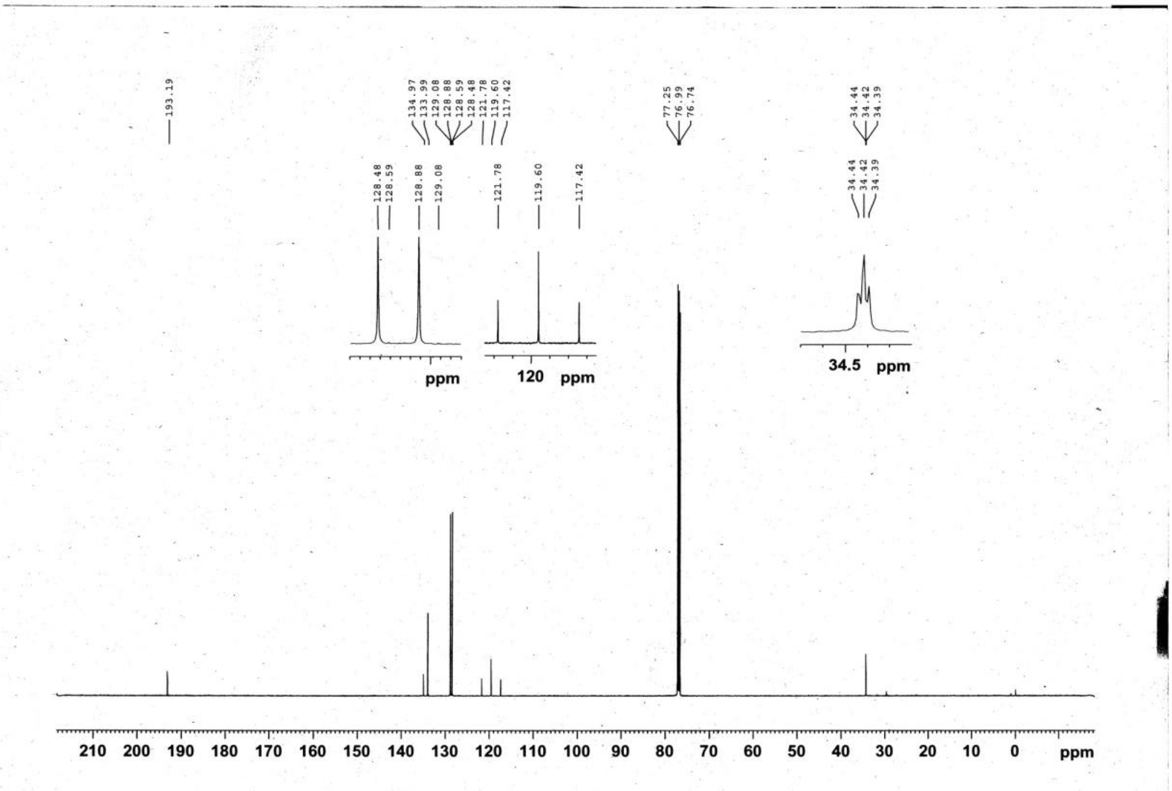

193.19
134.97
133.99
129.08
128.88
128.59
128.48
121.78
119.60
117.42
77.25
76.99
76.74
34.44
34.42
34.39
128.48
128.59
128.88
129.08
ppm
121.78
119.60
117.42
120 ppm
34.44
34.42
34.39
34.5 ppm
210 200 190 180 170 160 150 140 130 120 110 100 90 80 70 60 50 40 30 20 10 0 ppm

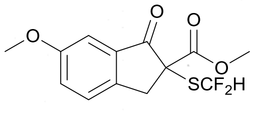

10x

^{19}F NMR (282 MHz, $CDCl_3$)
^{1}H NMR (300 MHz, $CDCl_3$)
^{13}C NMR (125 MHz, $CDCl_3$)

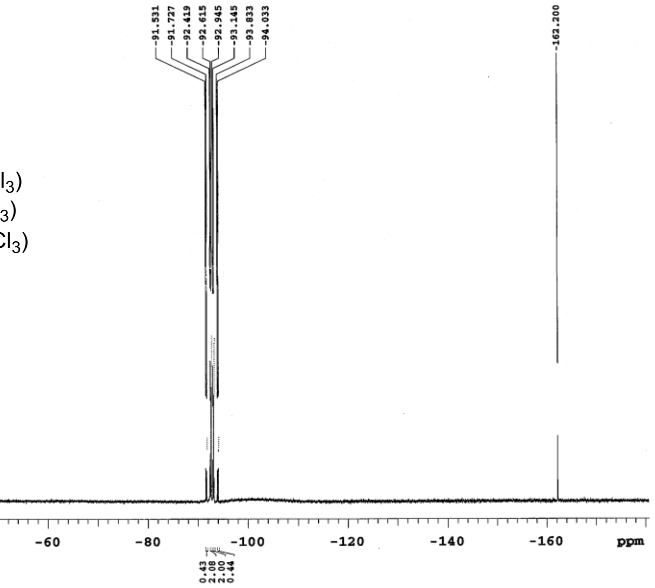

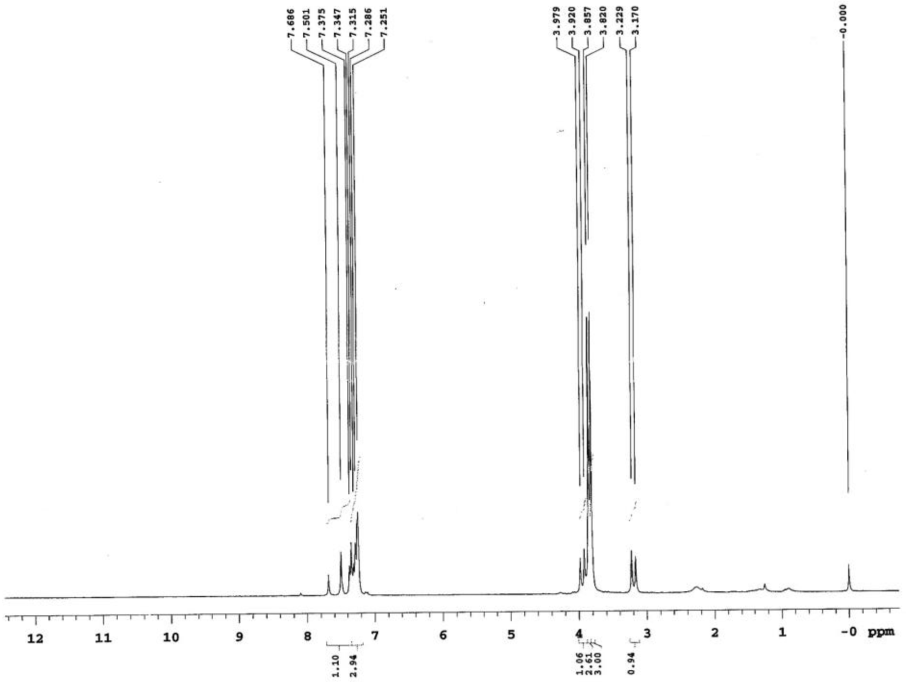

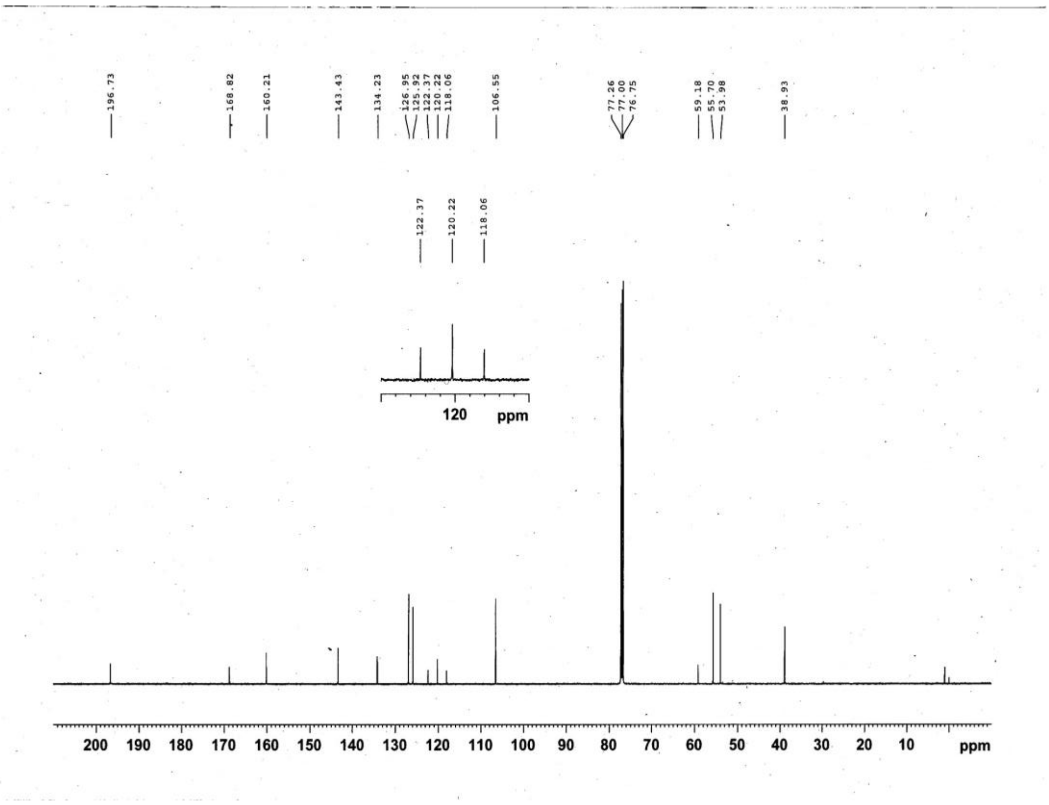

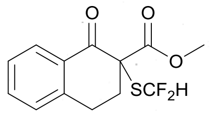

10y

^{19}F NMR (282 MHz, $CDCl_3$)
^{1}H NMR (300 MHz, $CDCl_3$)
^{13}C NMR (125 MHz, $CDCl_3$)

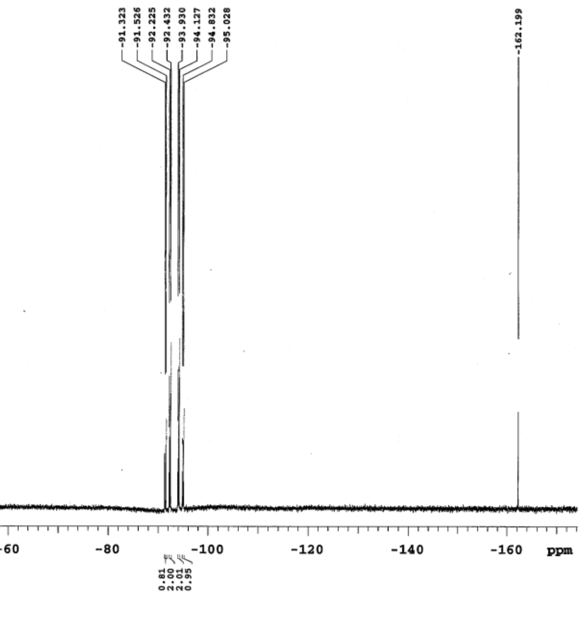

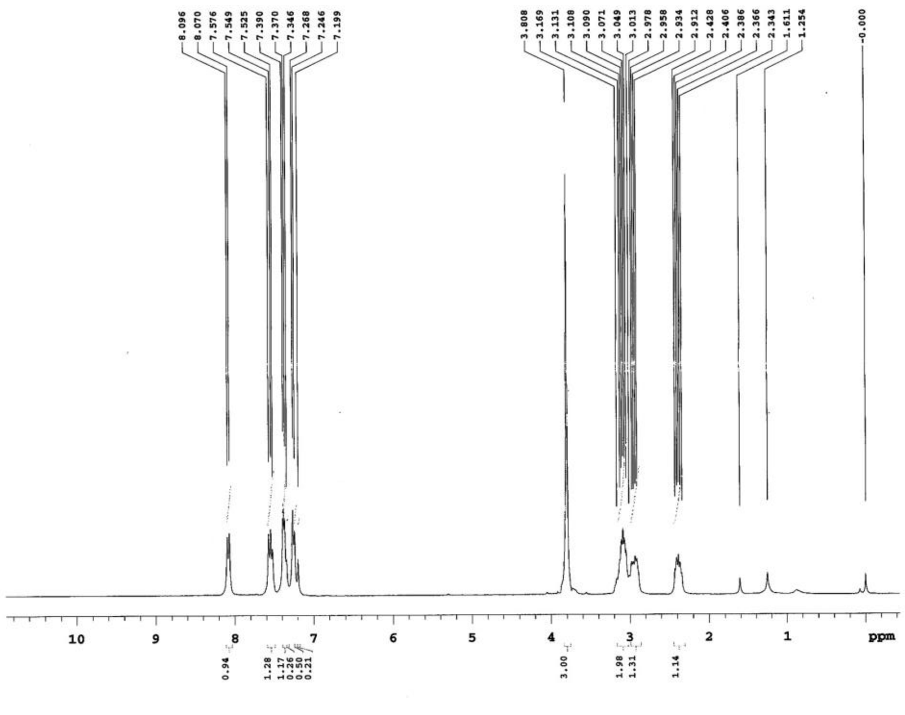
8.096
8.070
7.576
7.549
7.525
7.390
7.370
7.346
7.268
7.246
7.199
3.808
3.169
3.131
3.108
3.090
3.071
3.049
3.013
2.978
2.958
2.934
2.912
2.428
2.406
2.386
2.366
2.343
1.611
1.254
0.000
10 9 8 7 6 5 4 3 2 1 ppm
0.94
1.28
1.17
0.26
0.50
0.21
3.00
1.98
1.31
1.14

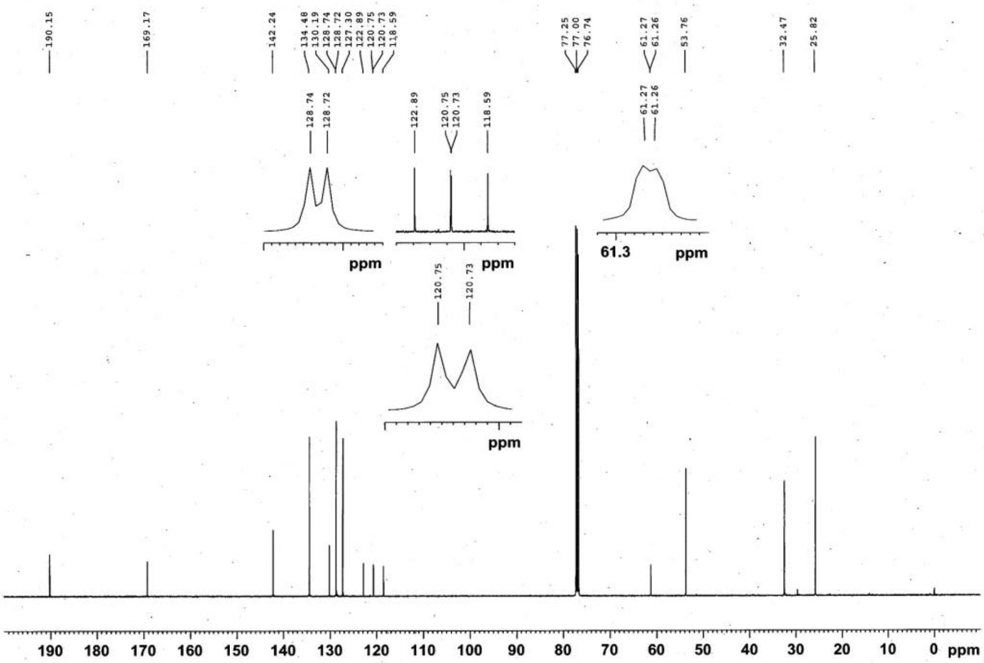
190.15
169.17
142.24
134.48
130.19
128.74
128.72
127.30
122.89
120.75
120.73
118.59
77.25
77.00
76.74
61.27
61.26
53.76
32.47
25.82
128.74
128.72
ppm
122.89
120.75
120.73
118.59
ppm
61.27
61.26
61.3
ppm
120.75
120.73
ppm
190 180 170 160 150 140 130 120 110 100 90 80 70 60 50 40 30 20 10 0 ppm

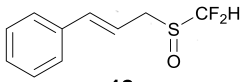

12a

^{19}F NMR (282 MHz, $CDCl_3$)
^{1}H NMR (300 MHz, $CDCl_3$)
^{13}C NMR (125 MHz, $CDCl_3$)

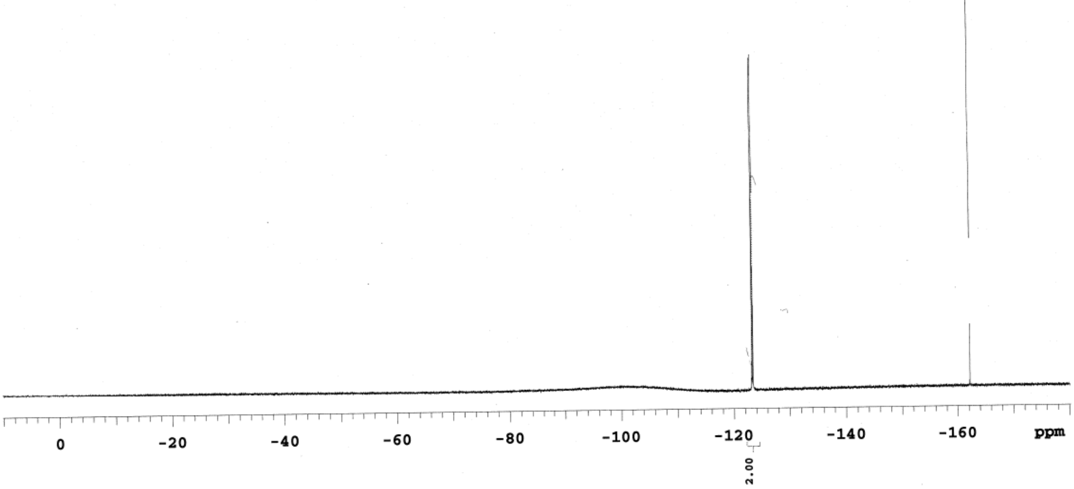

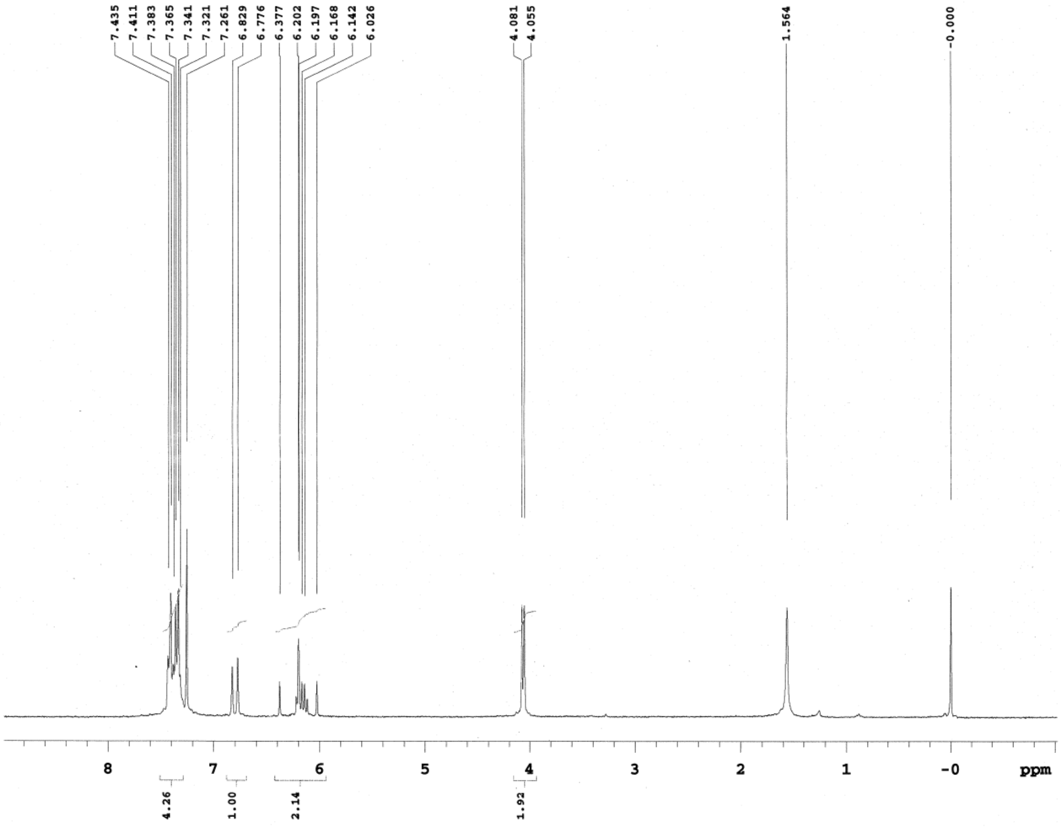

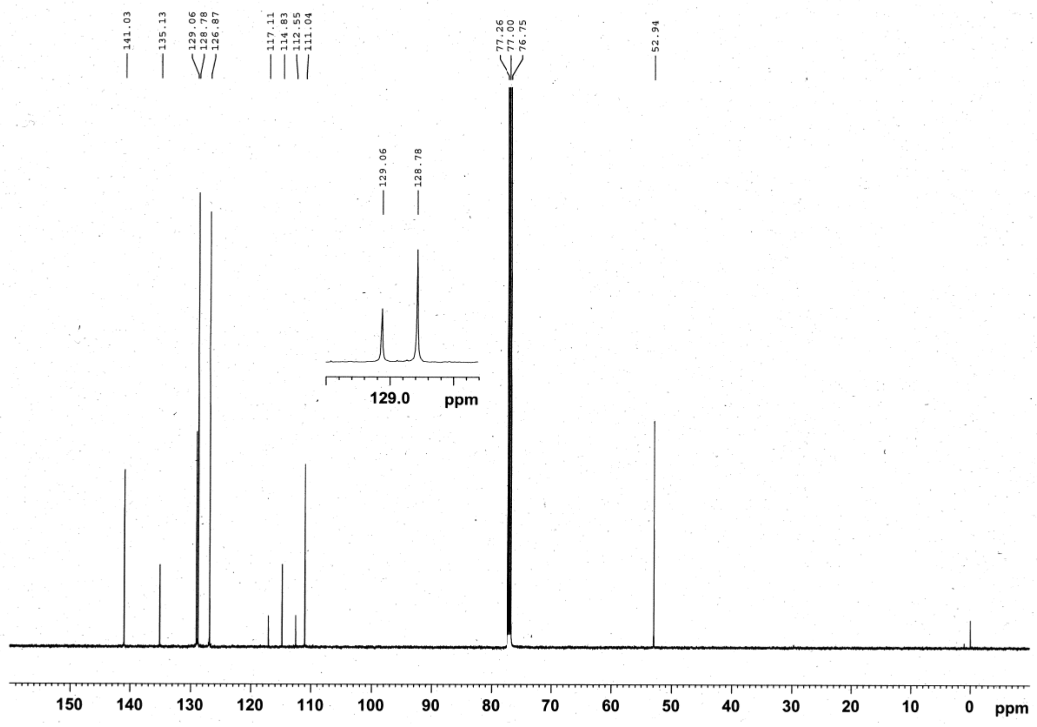

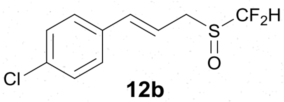

12b

^{19}F NMR (282 MHz, $CDCl_3$)
^{1}H NMR (300 MHz, $CDCl_3$)
^{13}C NMR (125 MHz, $CDCl_3$)

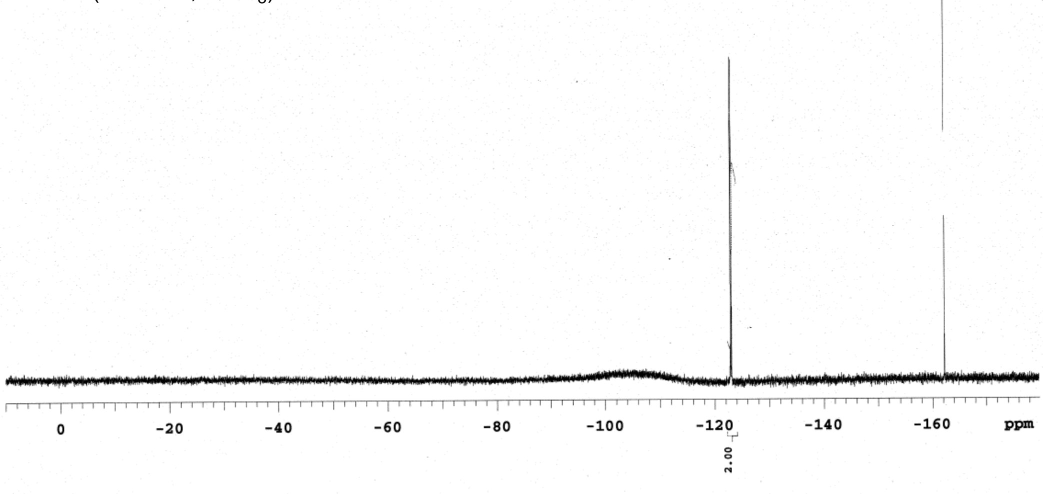

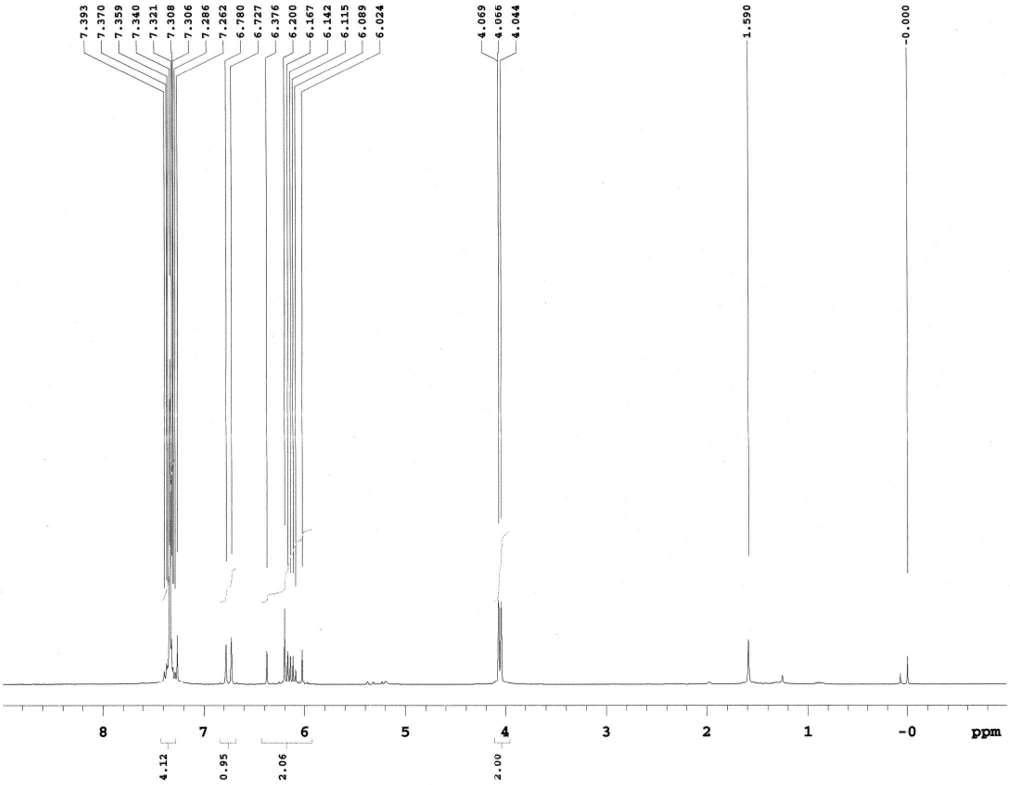
7.393
7.370
7.359
7.340
7.321
7.308
7.306
7.286
7.262
6.780
6.727
6.376
6.200
6.167
6.142
6.115
6.089
6.024
4.069
4.066
4.044
1.590
-0.000
8
7
6
5
4
3
2
1
-0
ppm
4.12
0.95
2.06
2.00

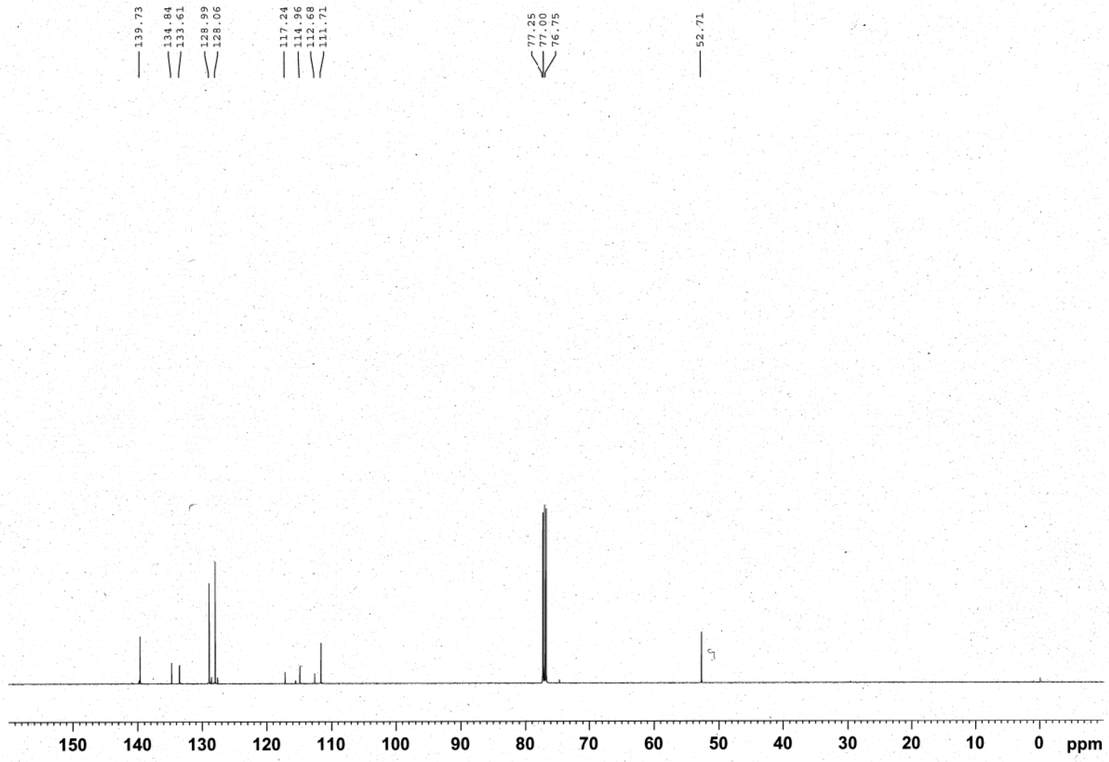
139.73
134.84
133.61
128.99
128.06
117.24
114.96
112.68
111.71
77.25
77.00
76.75
52.71
150
140
130
120
110
100
90
80
70
60
50
40
30
20
10
0
ppm

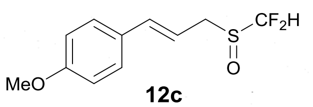

12c

^{19}F NMR (282 MHz, $CDCl_3$)
^{1}H NMR (300 MHz, $CDCl_3$)
^{13}C NMR (125 MHz, $CDCl_3$)

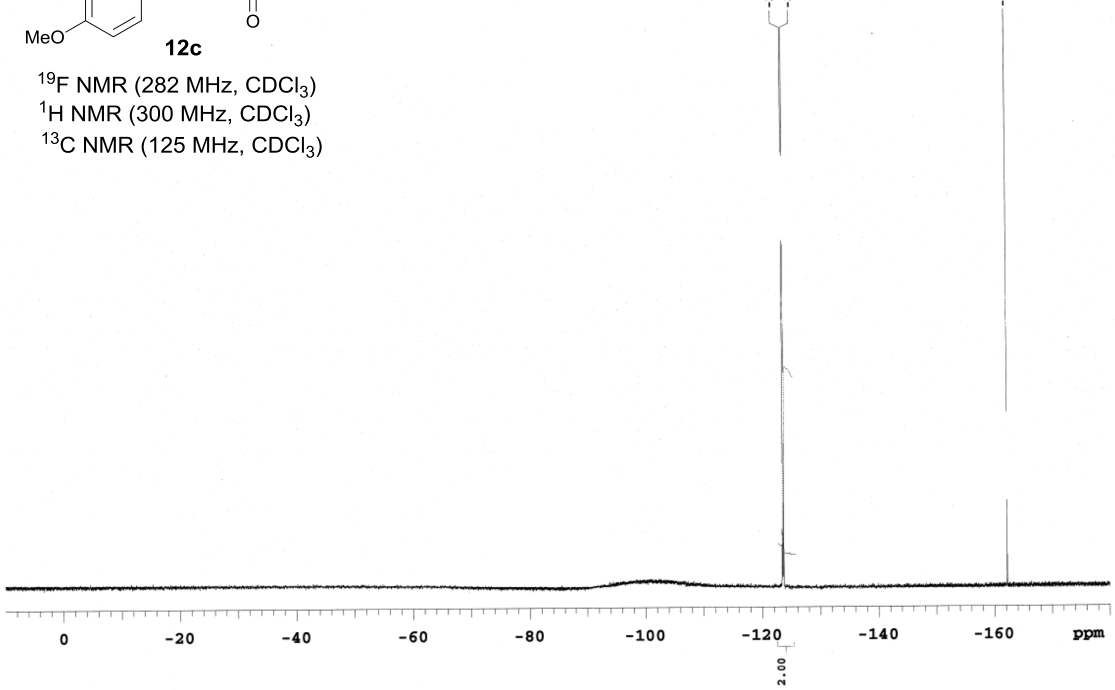

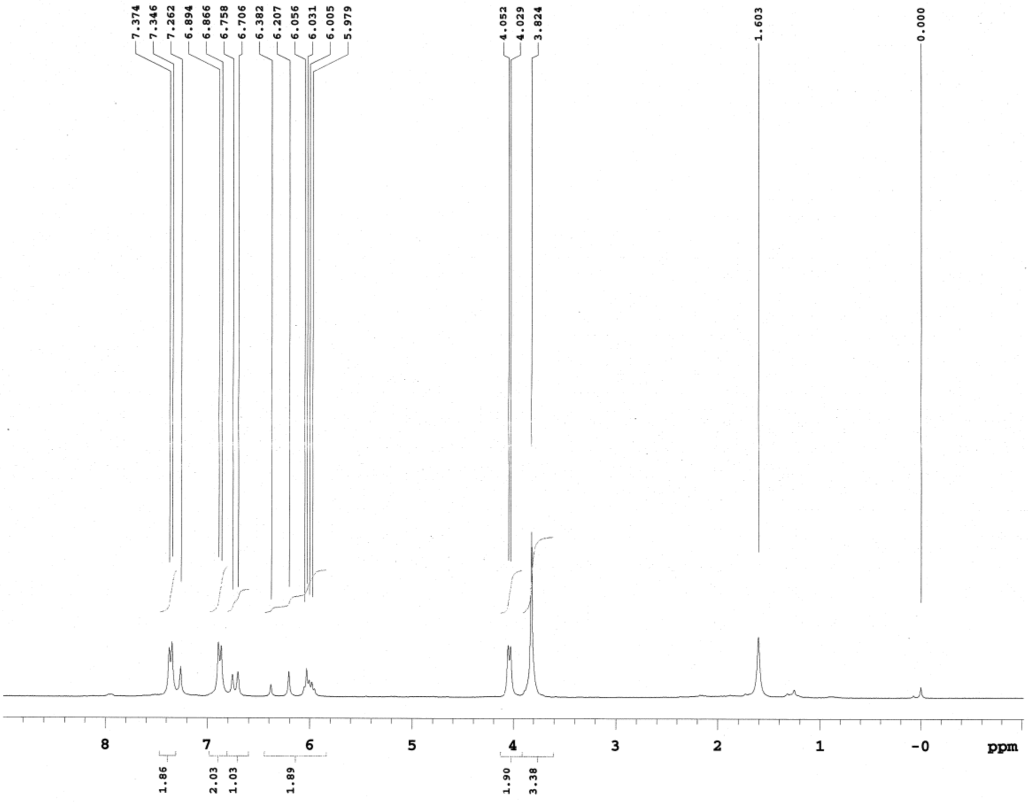

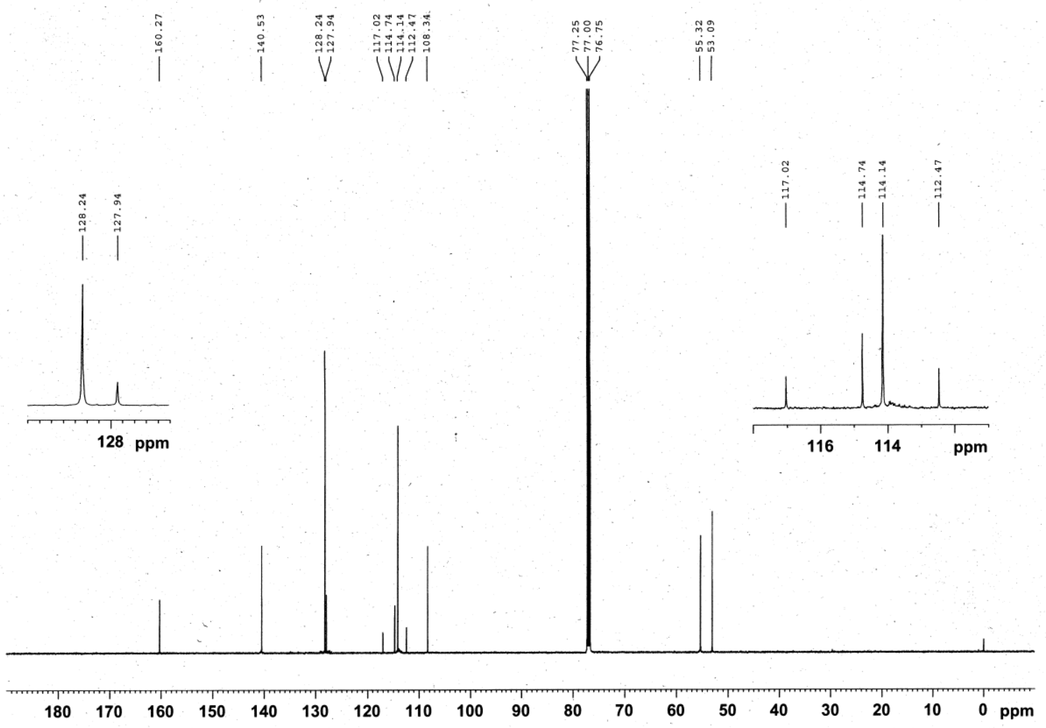

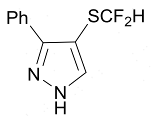

13

^{19}F NMR (282 MHz, $CDCl_3$)

^{1}H NMR (300 MHz, $CDCl_3$)

^{13}C NMR (125 MHz, $CDCl_3$)

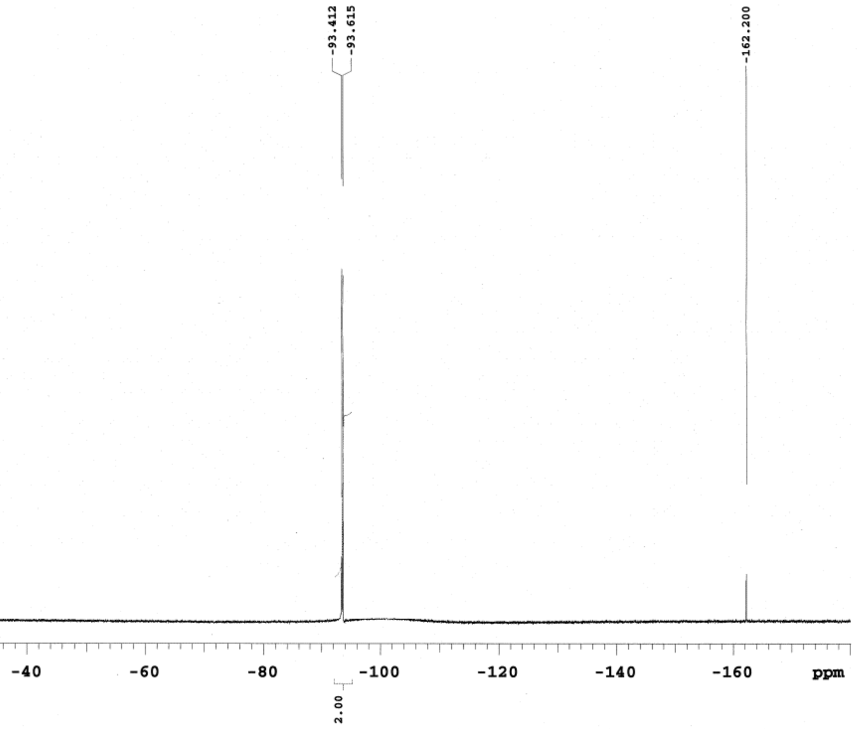

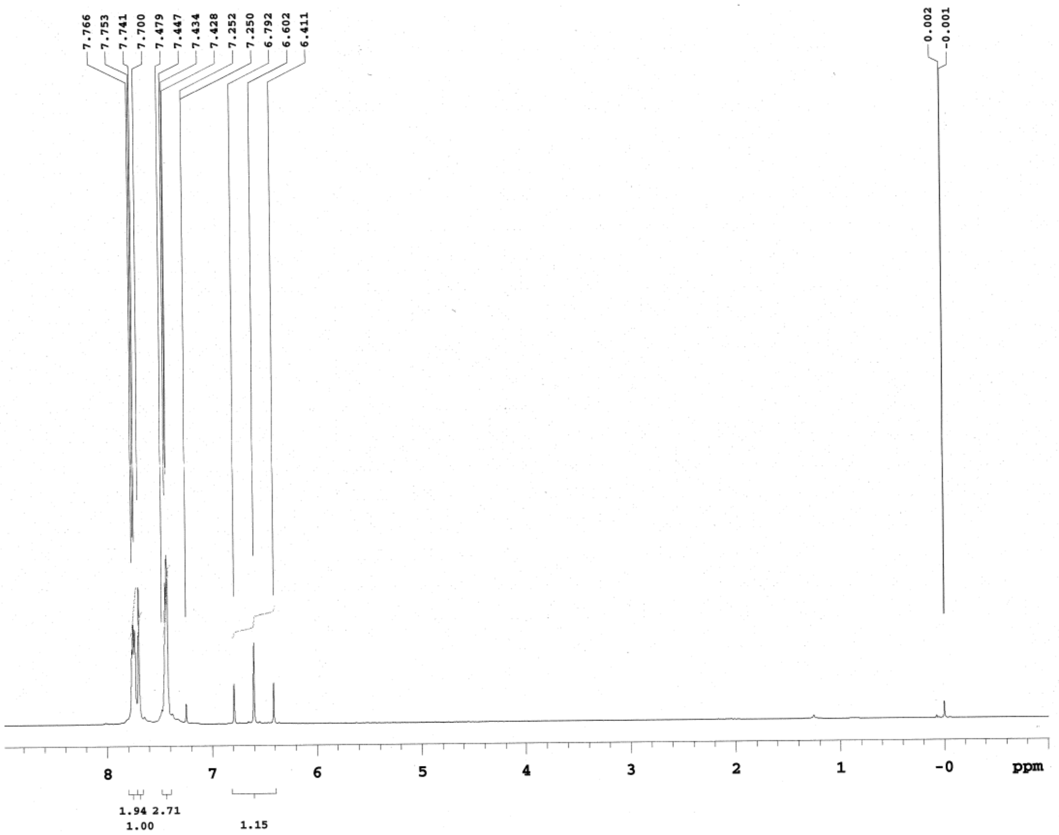
7.766
7.753
7.741
7.700
7.479
7.447
7.434
7.428
7.252
7.250
6.792
6.602
6.411
0.002
-0.001
8
7
6
5
4
3
2
1
-0
ppm
1.94
2.71
1.00
1.15

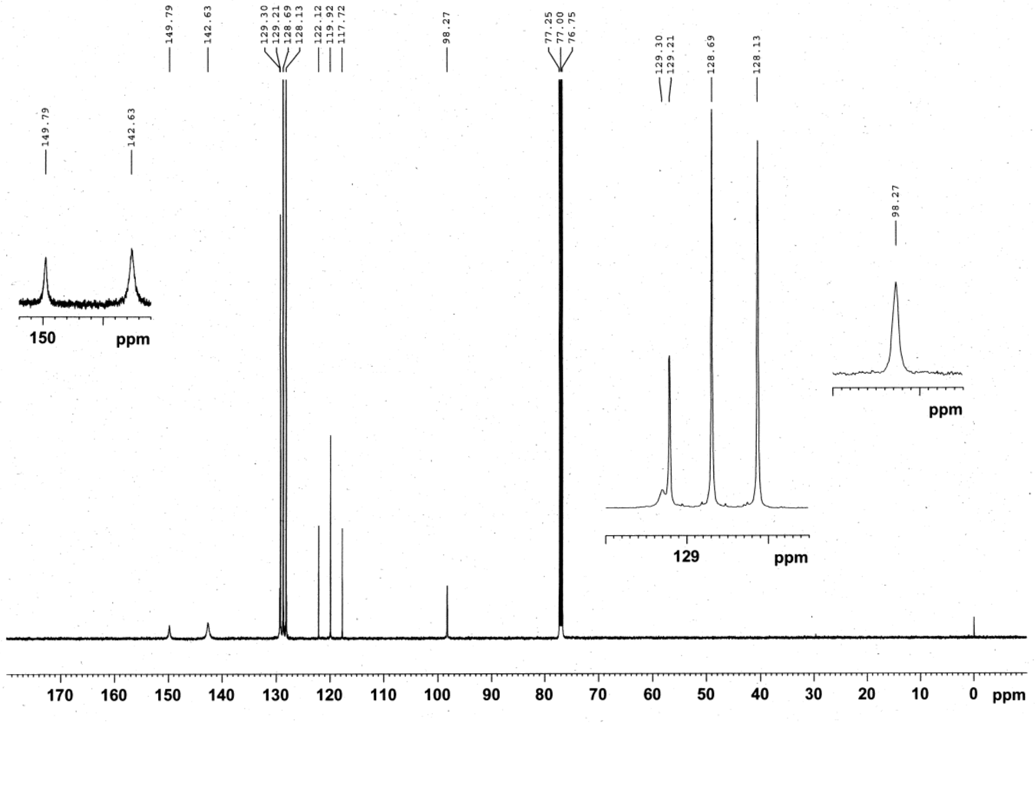
149.79
142.63
129.30
129.21
128.69
128.13
122.12
119.92
117.72
98.27
77.25
77.00
76.75
150
ppm
129
ppm
ppm
170
160
150
140
130
120
110
100
90
80
70
60
50
40
30
20
10
0
ppm

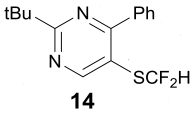

14

^{19}F NMR (282 MHz, $CDCl_3$)
^{1}H NMR (300 MHz, $CDCl_3$)
^{13}C NMR (125 MHz, $CDCl_3$)

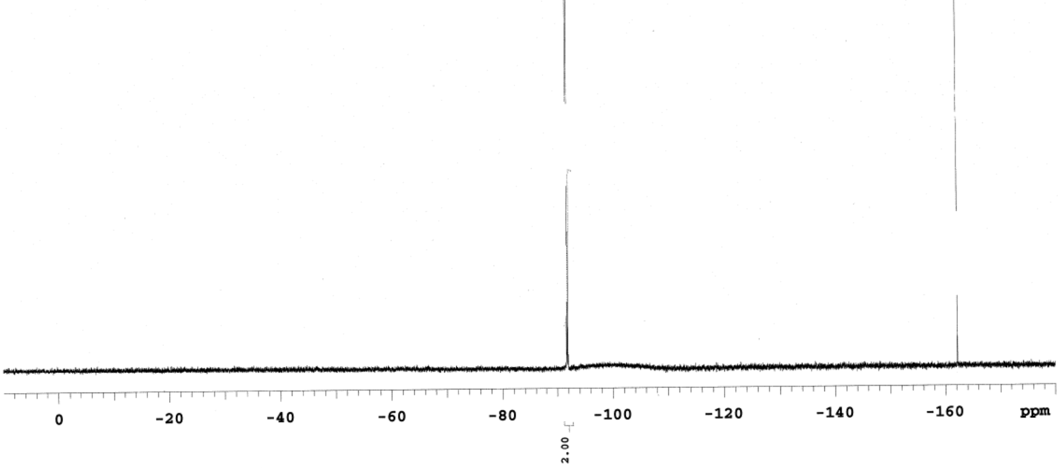

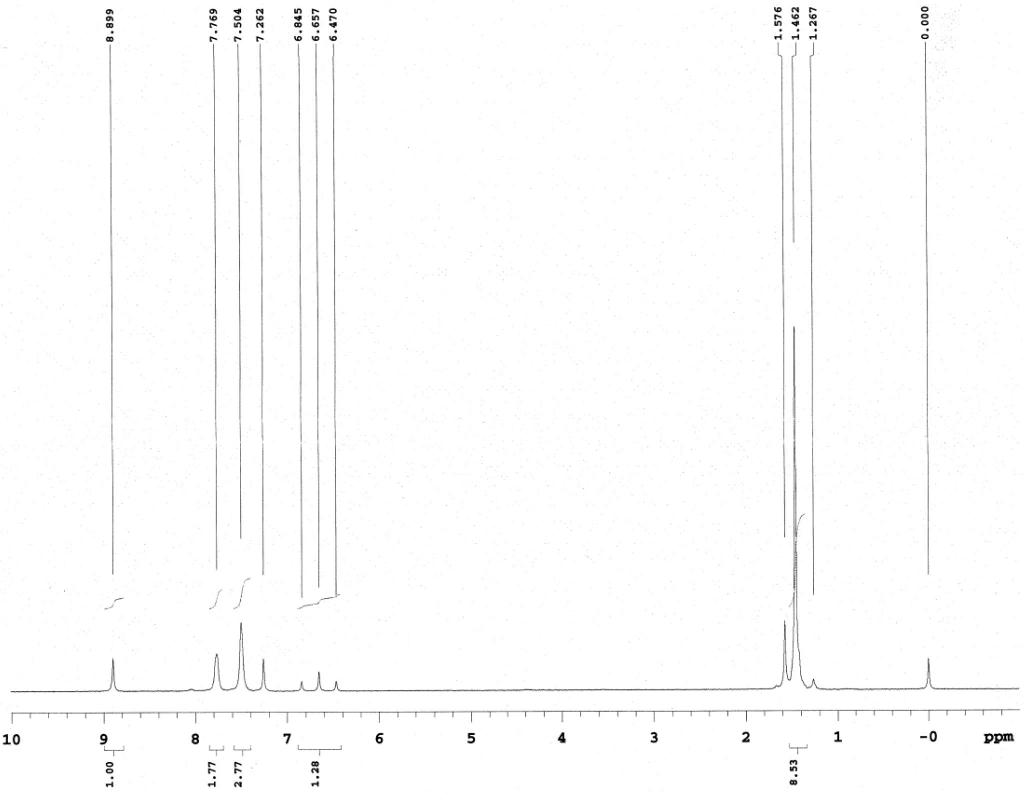

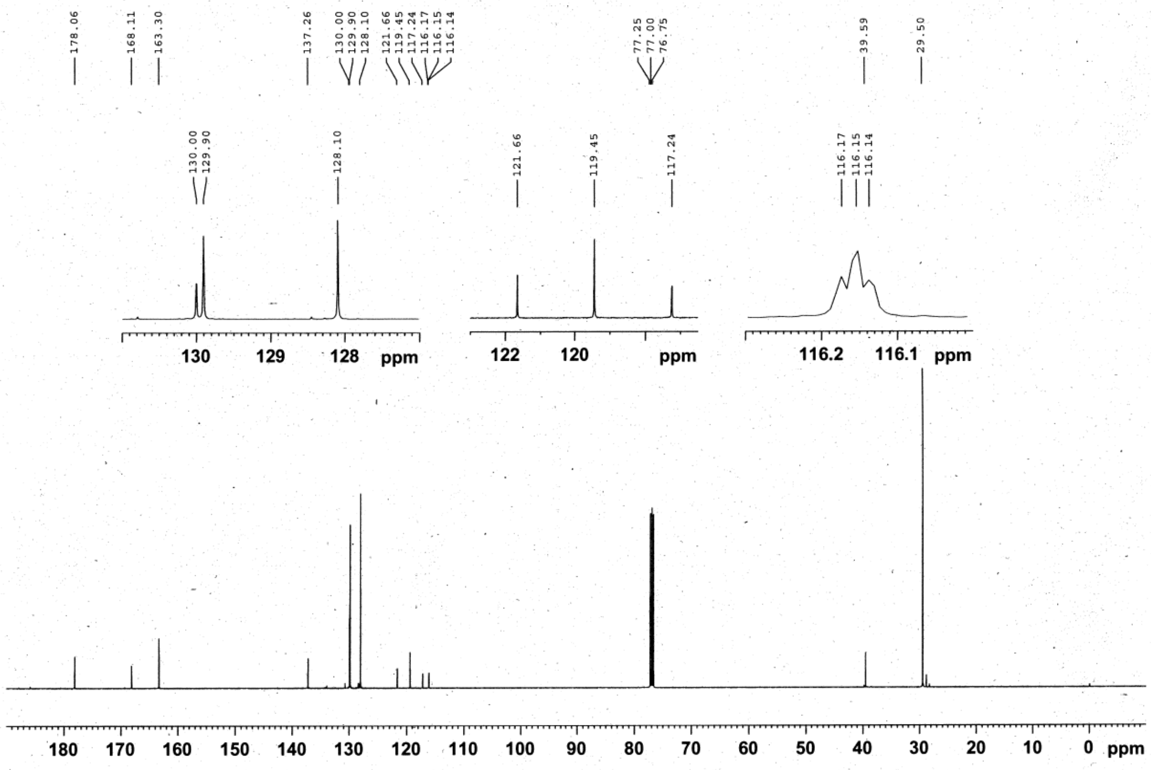

178.06
168.11
163.30
137.26
130.00
129.90
128.10
121.66
119.45
117.24
116.17
116.15
116.14
77.25
77.00
76.75
39.59
29.50
130
129
128
ppm
122
120
ppm
116.2
116.1
ppm
180
170
160
150
140
130
120
110
100
90
80
70
60
50
40
30
20
10
0
ppm
